# Triphenylene Diazocines: Butterfly-Type Rigid Photoswitches with Annulated Aromatic Ring Systems and Increased Switching Amplitude

**DOI:** 10.1021/acs.orglett.5c01398

**Published:** 2025-06-02

**Authors:** Artjom Businski, Daniel Hugenbusch, Thuy C. Ta, Ramina Tayaran, Lara Unterriker, Jan-Simon von Glasenapp, Christian Näther, Rainer Herges

**Affiliations:** † Otto Diels Institute of Organic Chemistry, 9179 Kiel University, Otto-Hahn-Platz 4, 24118 Kiel, Germany; # Institute of Inorganic Chemistry, 9179 Kiel University, Max-Eyth-Str. 2, 24118 Kiel, Germany

## Abstract

Azobenzenes are arguably the most frequently used photoswitches, but systems in which the benzene units are replaced by larger π systems are rare. Azonaphthalene is known, but the next higher homologue azoanthracene already undergoes irreversible intramolecular cycloaddition during photoisomerization. Such side reactions are not possible with rigid diazocines. In the present work, we have succeeded in integrating triphenylene groups into diazocines, making them the azo switches with the largest aromatic, annulated ring systems published to date. Both the unsubstituted system and several derivatives are easy to access. The extension of the π systems results in a larger molecular switching amplitude compared to parent diazocine, which should lead to better force transmission to the environment in material applications. The switching wavelengths are shifted bathochromically into the visible range, although the photostationary equilibria are decreased. Potential applications include switchable liquid crystals, mechanophores, photoactuators, and many other responsive materials. Particularly noteworthy is the 4-fold substitution of the system, which allows incorporation into larger systems, e.g. as multifunctional cross-linkers in polymers or as building blocks in COFs and MOFs.

## Graphical abstract

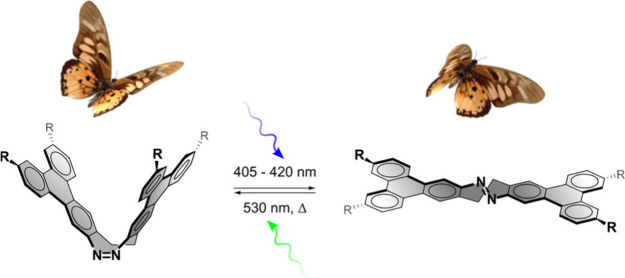

Diazocines (DA) are bridged azo based photoswitches and exhibit particular properties with significant advantages over the azobenzene parent system.^1,2^ Diazocines have an inverted stability, i.e. the *Z* isomer is more stable than the *E* form, the switching wavelengths are bathochromically shifted and the structures are rigid, with no free rotation of the phenyl groups, resulting in very high quantum yields for photoswitching.^1–3^ Especially the rigid structure potentially allows larger, extended π systems to be integrated into the switchable system. The largest aryl units that have been realized in extended azo switches are azonaphthalenes.^4,5^ Although the next higher homologue, azoanthracene could be theoretically studied and synthesized, upon exposure to light it undergoes an irreversible, intramolecular [4 + 4] cycloaddition besides *E* → *Z* isomerization.^6,7^ In the present work, we show, that much larger π systems can be incorporated into diazocines without causing side reactions during photoisomerization. Our triphenylene diazocines (TPD) are easily accessible and exhibit switching wavelengths in the visible range (405–420 nm, 530 nm, Figure 1) and long-term stability with no measurable fatigue over 15 switching cycles (Figure 2). The extension of the “aromatic lever” during *Z* → *E* isomerization also increases the molecular movement amplitude Δ. In the extended TPD the amplitude Δ is 1.73 times larger (Δ_TPD_ = 4.5 Å) than in the parent diazocine system (Δ_DA_ = 2.6 Å).^8^ Moreover, the four substituents are at maximum distance from each other and point in different spatial directions. In the *Z* configuration they form a distorted square and in the *E* form a distorted tetrahedron. Therefore, the implementation of TPDs has further advantages when used as switchable building blocks in framework and network materials such as photoswitchable polymers,^9–11^ MOFs^12,13^ and COFs.^14,15^ Further potential applications are photoresponsive cages and helicates.^16–20^ Moreover, the tub-shaped conformation of the extended aromatic systems with a central 8-membered ring such as our TPD is an ideal structural unit to favor the formation of columnar liquid crystals. Similar structures have been used to build photoresponsive liquid crystals leading to a number of applications such as photoswitchable adhesives.^21,22^

**Figure 1:**
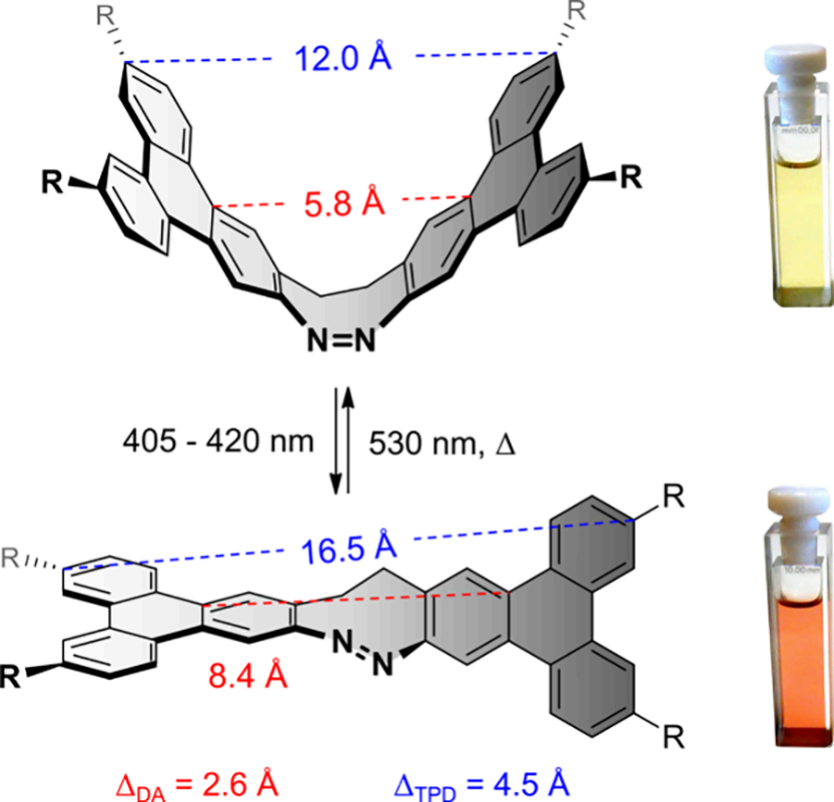
Light-driven reversible *Z* ⇄ *E* isomerization of TPD.^8^

**Figure 2:**
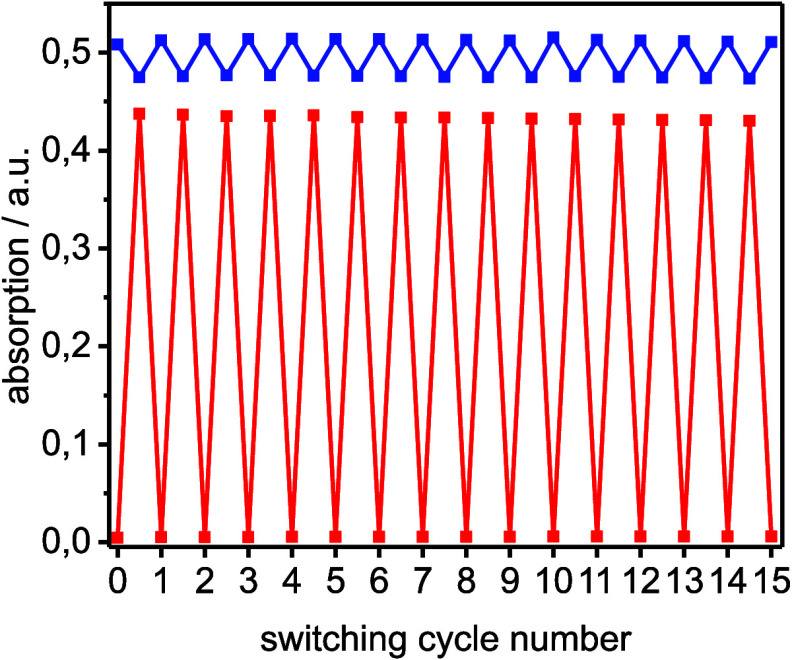
Absorption of TPD **18** at λ = 421 nm (blue) and λ = 507 nm (red) after alternating irradiation at 420 and 530 nm.

The key reaction used for the synthesis of the triphenylene units is a multiple Stille cross-coupling reaction between tetrabromo diazocine **5** and stannafluorenes **13**–**17**.^23^ Diazocine **5** was synthesized in four steps from dibromo toluene **1** by nitration, oxidative dimerization,^24^ reduction and azocyclization^25^ (Scheme 1). First, the aryl bromide **1** underwent a nitration reaction yielding 61% of the nitro compound **2**. The consecutive addition of KO*t*Bu and bromine,^24^ lead to an inseparable mixture, consisting of the desired dimer **3a** and the byproduct **3b** (*E*/*Z*, see section S 2.1 in the Supporting Information). However, after reduction the resulting amine **4** could be purified and isolated in 85% yield. The subsequent azocyclization with *m*CPBA,^25^ yielded the tetrabromo diazocine **5** that was directly obtained by filtration from the reaction mixture due to its very low solubility. It should be noted that the desired diazocine **5** precipitated from the reaction solution alongside with the corresponding azoxy compound. Separation of the product mixture was possible but tedious. Moreover, the azoxy compound could not be cleanly reduced to the diazocine **5**. Therefore, it was more advantageous at this point to continue working with the mixture of azo and azoxy compound, as purification was easier at the next synthetic stage. The syntheses of diazocine **5** and its precursors **2** – **4** were carried out on a multigram scale. The second component of the Stille coupling,^23^ the stannafluorenes, were synthesized analogously to known procedures for the parent and methoxy system **17** and **16**.^23,26^ Starting from the dihydroxy biphenyl **6**,^27^ Williamson ether synthesis using methyl iodide, potassium carbonate and acetone, converted both hydroxyl groups into methyl ethers in 99% yield (Scheme 2). Subsequent bromination with NBS in acetonitrile of the dimethoxy biphenyl **7** provided dibromo biphenyl **8** in 83% yield.^28^ Cleavage of the methoxy groups with boron tribromide in DCM lead to the formation of the brominated hydroxy compound **9** in 98% yield.^23^ Conversion of the hydroxy groups by silylation,^29^ iridium-catalyzed vinylation^30^ and Williamson ether synthesis^23^ resulted in differently functionalized compounds **10** – **12** in up to 99% yield. Stannylation and formation of the fluorene structure was accomplished by bromo-lithium exchange with *n*-butyllithium and reaction with dimethyltin dichloride.^23,26^ While the silylated compound **13** was obtained as a pure product through precipitation in 62% yield, the vinylated as well as alkylated derivatives **14** – **15** could not be isolated in high purity due to their instability (see section S 2.3). The syntheses within Scheme 2 were carried out on a multigram scale. Further Stille coupling reactions^23^ between tetrabromo diazocine **5** and different substituted stannafluorenes **13** – **17** led to the corresponding TPDs **18** – **22** in yields between 10 – 81% (Scheme 3). Especially TPD **18** is of great interest, since this compound was isolated in high purity on a multigram scale. TPD **18** is a stable compound and a good starting point for further derivatization through one-pot rapid cleavage of the silyl groups using TBAF in THF,^29^ followed by *in situ* treatment with different electrophilic reagents. Using this method, TPDs **23** – **31** were obtained up to 92% yield.

**Scheme 1:**
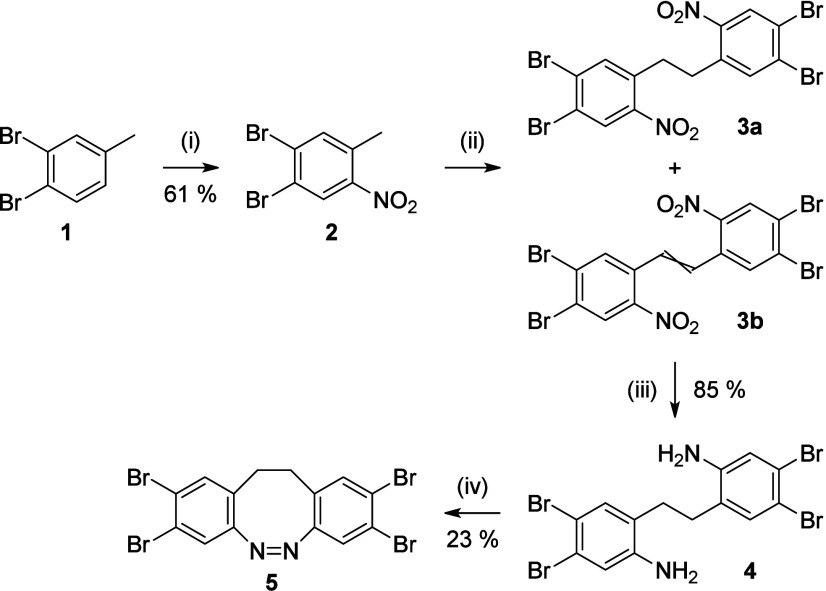
Synthesis of Tetrabromo Diazocine **5** ^a^

a Reaction conditions: (i) fum. HNO_3_, DCM, 2 h, −10 °C; (ii) (1) KO*t*Bu, THF, 30 s, −10 °C, (2) Br_2_, 10 min, −10 °C; (iii) SnCl_2_·2H_2_O, AcOEt, 18 h, 80 °C; (iv) *m*CPBA, DCM/AcOH (3:1), 24 h, rt.

**Scheme 2:**
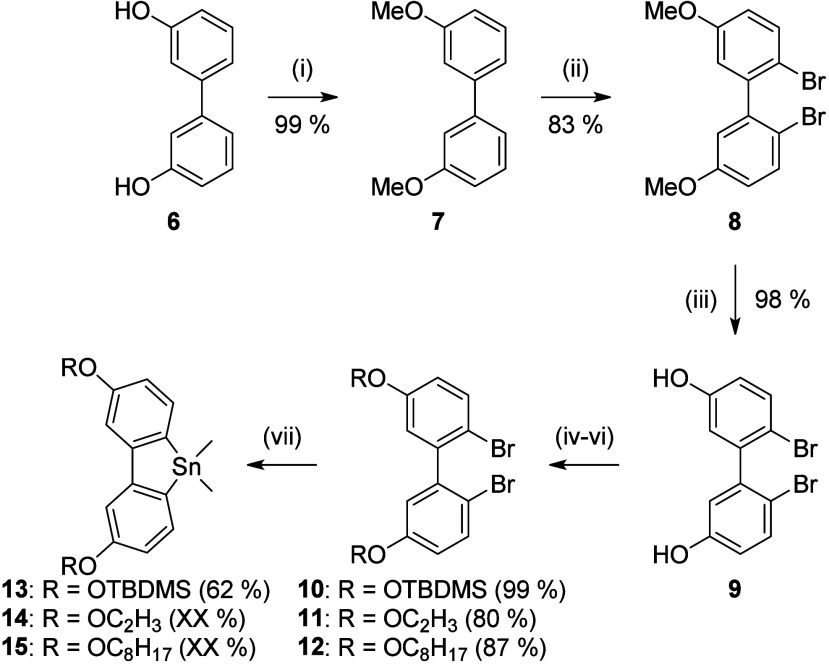
Synthesis of Stannafluorenes **13**–**15** ^a^

a Reaction conditions: (i) MeI, K_2_CO_3_, acetone, 18 h, 70 °C; (ii) NBS, MeCN, 18 h,–10 °C → rt; (iii) BBr_3_, DCM, 18 h, −78 °C → rt; (iv, **10**) TBDMS-Cl, imidazole, DCM, 15 h, rt; (v, **11**) [Ir­(cod)­Cl]_2_, vinyl acetate, Na_2_CO_3_, toluene, 24 h, 100 °C; (vi, **12**) C_8_H_17_OTs, Cs_2_CO_3_, acetone, 17 h, 70 °C; (vii) (1) *n*-BuLi, Et_2_O, 3 h, −78 °C. (2) Me_2_SnCl_2_, Et_2_O, 15 h, −78 °C → rt.

**Scheme 3:**
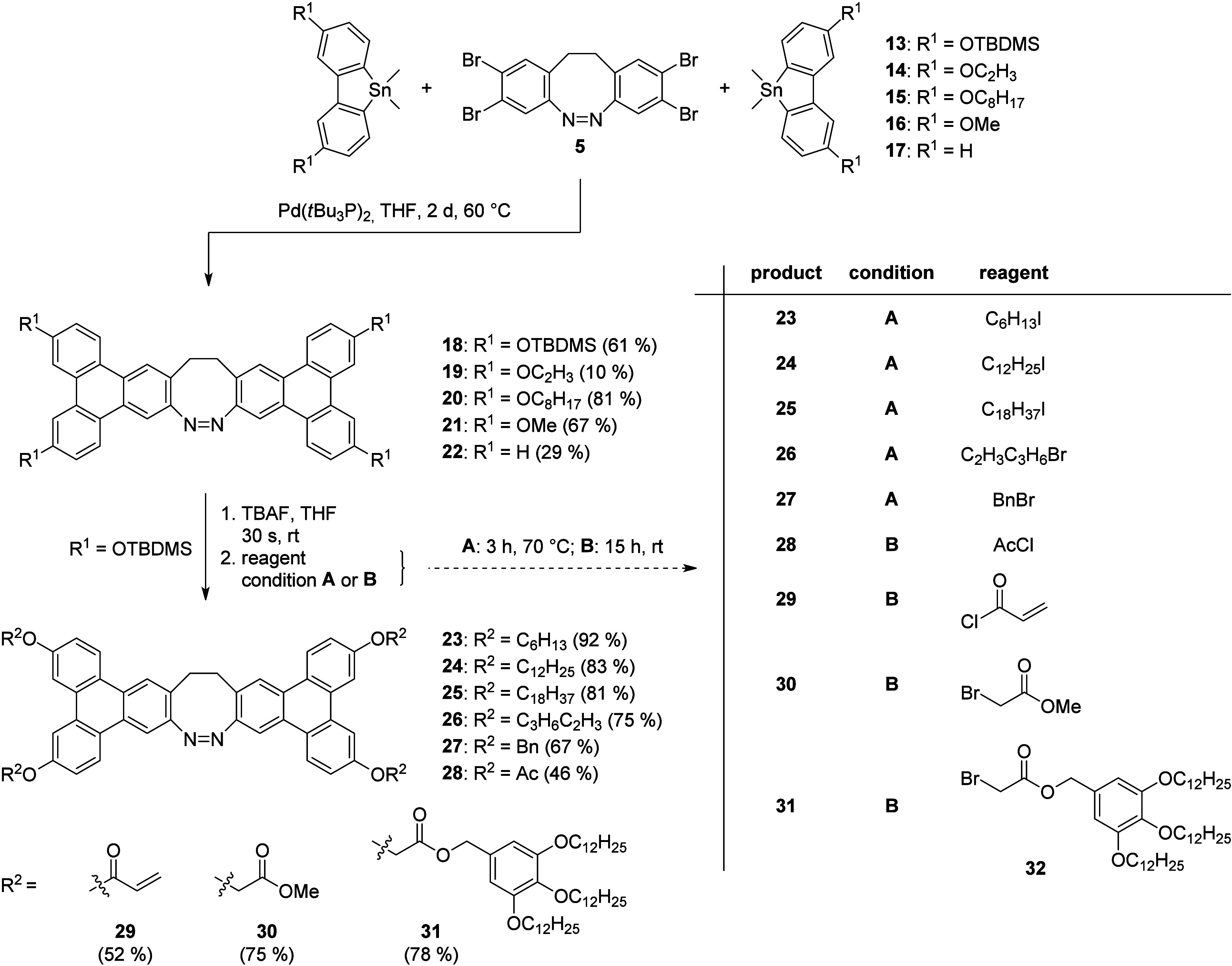
Synthesis of Bistriphenylene Diazocines **18**–**31**

TPDs **21** and **22** showed almost complete insolubility due to π···π stacking of the triphenylene units, which made characterization through NMR and UV/vis spectroscopy difficult (see sections S 2.4, S 3, and S 4). Compared to the parent diazocine,^1^ maxima of absorption bands of TPDs are bathochromically shifted and appear within a range of λ_max_ (*Z*) = 405 – 410 nm and λ_max_ (*E*) = 504–507 nm (Table 1). Conversion to the photostationary state was performed with light at λ = 405 and 420 nm, depending on the type of substituent and its electronic effect. It is noteworthy, that for the ester functionalized TPDs **28** and **29** the switching efficiency of the *Z* → *E* conversion is higher and the thermal half-life shorter, compared to the remaining ether functionalized TPDs **18**–**20**, **23**–**27**, **30**, and **31**. Due to the extended π system of the triphenylene units, the ππ* transition is shifted to longer wavelengths and overlaps with the nπ* band of the *Z* isomer. This is probably the reason, why TPDs show lower switching efficiencies than the parent diazocine itself.^1^ This overlap is less pronounced in the ester substituted TPDs **28** and **29** as compared to the ether functionalized TPDs **18**–**20**, **23, 27**, **30**, and **31**, which leads to higher switching efficiencies of TPDs **28** and **29** (Figure 3). None of the TPDs exhibit fluorescence which is probably due to quenching of the emission by the azo group.

**Figure 3:**
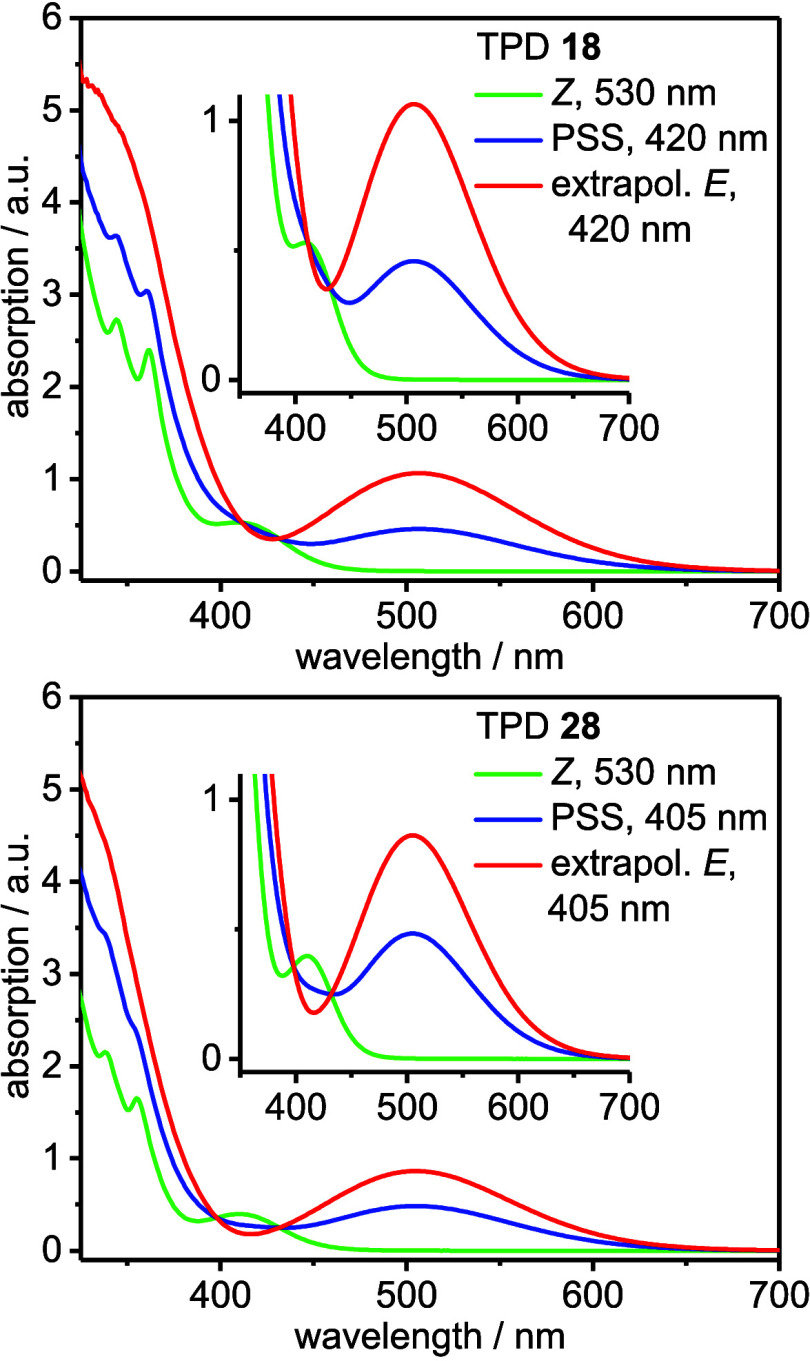
UV/vis absorption spectra of TPDs **18** and **28** (THF, 300 μM, 25 °C).

**Table 1:** Photophysical Properties of Diazocines **5** and **18** −**31**

	compound														
	**5** ^a^ ^,^ ^d^	**18** ^a^ ^,^ ^d^	**19** ^a^ ^,^ ^d^	**20** ^a^ ^,^ ^d^	**21** ^c^ ^,^ ^e^	**22** ^c^ ^,^ ^e^	**23** ^a^ ^,^ ^d^	**24** ^a^ ^,^ ^d^	**25** ^a^ ^,^ ^d^	**26** ^a^ ^,^ ^d^	**27** ^b^ ^,^ ^e^	**28** ^a^ ^,^ ^d^	**29** ^a^ ^,^ ^d^	**30** ^b^ ^,^ ^e^	**31** ^a^ ^,^ ^d^
λ_max_ (*Z*)/nm	405	410	409	410	410	405	410	410	410	410	410	410	410	410	407
λ_max_ (*E*)/nm	492	507	505	507	505	504	507	507	507	507	506	505	504	506	504
*k*/10^–4^ s^–1^	0.881	1.56	1.59	1.42	0.944	1.06	1.25	1.39	1.40	1.49	0.993	1.74	1.65	0.972	1.57
*t*_1/2_/min	131	74.2	72.9	81.3	122	109	92.7	83.0	82.4	77.5	116	66.1	69.9	119	73.6
Γ_*Z*→*E*_/%	81^f^	43^h^	44^h^	39^h^	40^h^	53^g^	41^h^	42^h^	41^h^	41^h^	39^h^	57^g^	56^g^	38^h^	45^h^
Γ_*E*→*Z*_/%^i^	>99	>99	>99	>99	>99	>99	>99	>99	>99	>99	>99	>99	>99	>99	>99

a UV/vis spectra were measured in THF (25 °C, 300 μM).

b DMSO (25 °C, 300 μM).

c DMSO (25 °C, 50 μM).

d NMR spectra for the determination of the conversion rates Γ were measured in THF-*d*_*8*_ (0 °C).

e NMR spectra for the determination of the conversion rates Γ were measured in DMSO-*d*_*6*_ (25 °C).

f Wavelength used for *Z* → *E* conversion (Γ_*Z*→*E*_): 385 nm.

g Wavelength used for *Z* → *E* conversion (Γ_*Z*→*E*_): 405 nm.

h Wavelength used for *Z* → *E* conversion (Γ_*Z*→*E*_): 420 nm.

i Wavelength used for *E* → *Z* conversion (Γ_*E*→*Z*_): 530 nm.

TPDs **18**, **21** and **22** were characterized by SC-XRD (see the Supporting Information). In all three compounds the TPDs are in the *Z* configuration and show a butterfly like structure (Figure 4). In the isostructural compounds **21** and **22** the molecules are arranged in columns, in which the top of each molecule fits perfectly into the aperture of a neighboring molecule (Figure 4, left). Within each column the molecules always point in the same direction, proving the noncentrosymmetry of this structure. Each triphenylene unit is parallel to neighboring units, which is indicative for π···π stacking interactions. Between the columns no pronounced interactions are observed. Finally, the columns are arranged in layers (see the Supporting Information). In TPD **18** the molecules are also arranged into columns, but these columns are built up of dimer-like units, in which each half of a molecule fits perfectly into the aperture of a neighboring molecule (Figure 4, right). Within these units the H atoms of the *tert*-butyl groups points toward the 6-membered rings, indicative for C–H···π interactions. (see the Supporting Information, Figure S118). In contrast to **18** and **22**, in the entire crystal structure there are no signs for π···π stacking interactions and between neighboring columns only weak van-der-Waals interactions seem to dominate. The observation, that no strong intermolecular interactions between the columns are observed, might be responsible for the fact, that these crystals are extremely soft, that some of them are bent and that they easily can be deformed with a preparation needle (see the Supporting Information).

**Figure 4:**
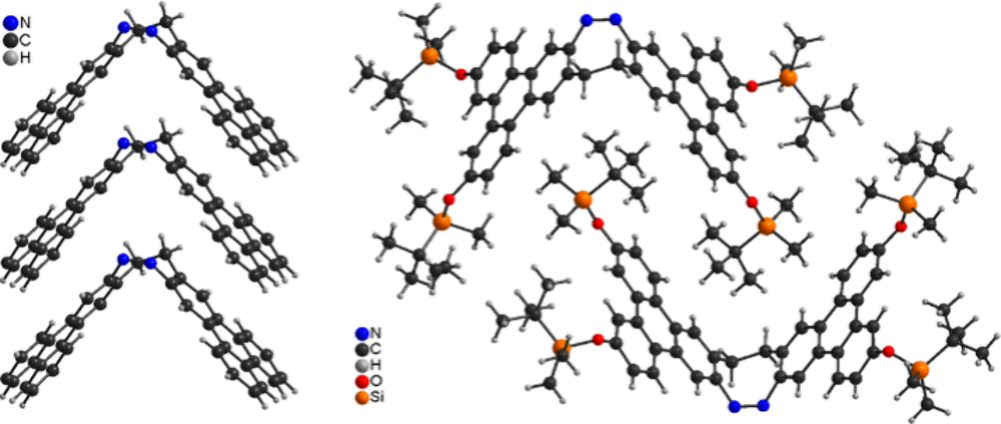
Left: View onto one column in the crystal structure of TPD **21** and **22** with **22** as representative. Right: View along a column in TPD **18**, showing the arrangement of two neighboring molecules.

With the TPDs, we have developed a simple synthetic approach to azo-based photoswitches (diazocines) including the largest π systems (triphenylene units) to date. The stable, silyl-protected compound **18** is an ideal starting point for the preparation of further tetrasubstituted systems. The thermodynamically stable *Z* form has a rigid tub-shaped conformation, which changes into the stretched, twisted *E* form when exposed to light of 405 or 420 nm. Upon irradiation with 530 nm, reisomerization to the original form takes place. The four substituents on the triphenylene units change from a coplanar arrangement (distorted square) in the *Z* configuration to that of a distorted tetrahedron in the *E* isomer. The isomerization is reversible and shows no fatigue even after 15 switching cycles. The amplitude of the molecular movement is increased by a factor of approximately 1.7 compared to parent diazocines due to the extended lever and the rigid structure. The exceptional structural features and properties are suggestive of applications, that are not readily accessible with conventional azobenzenes and diazocines. We see potential in the design of photoresponsive, porous scaffold materials, polymeric actuators and, in particular, photoswitchable, liquid crystalline systems.

## Supplementary Material



## Data Availability

The data underlying this study are available in the published article and its Supporting Information.

## Notes

Experimental methods, syntheses, and characterization, NMR spectra, photophysical characterization, and X-ray single crystal structure analysis (PDF)
