# Supplementary material for: Triphenylene Diazocines: Butterfly-Type Rigid Photoswitches with Annulated Aromatic Ring Systems and Increased Switching Amplitude
Source: Org Lett. 2025 Jun 2;28(1):1–6. doi: 10.1021/acs.orglett.5c01398 (PMC12797320; doi:10.1021/acs.orglett.5c01398)
Supplement: Supplementary file 1 [file ol5c01398_si_001.pdf]

Supporting Information

Triphenylene Diazocines: Butterfly-Type Rigid Photoswitches with Annulated Aromatic Ring Systems and Increased Switching Amplitude

Artjom Businski,† Daniel Hugenbusch,† Thuy C. Ta,† Ramina Tayaran,† Lara Unterriker,† Jan-Simon von Glasenapp,† Christian Näther,# Rainer Herges*†

†Otto Diels Institute of Organic Chemistry, Kiel University, Otto-Hahn-Platz 4, 24118 Kiel, Germany

#Institute of Inorganic Chemistry, Kiel University, Max-Eyth-Str. 2, 24118 Kiel, Germany

*Correspondence: rherges@oc.uni-kiel.de

Table of Contents

S 1 Experimental methods 1

S 2 Syntheses and characterization 2
- S 2.1 Synthesis of tetrabromo diazocine **5** 2
- S 2.2 Synthesis of compound **32** 5
- S 2.3 Synthesis of stannafluorenes **13 – 17** 6
- S 2.4 Synthesis of triphenylene diazocines **18 – 31** 13

S 3 NMR spectra 23

S 4 Photophysical characterization 51
- S 4.1 UV/vis absorption spectra and thermal half-lives 51
- S 4.2 Photostationary states 67

S 5 X-ray single crystal structure analysis 83

S 6 References 90

S 1 Experimental methods

General information

Unless otherwise stated, the syntheses were carried out at room temperature, under atmospheric pressure and atmospheric air. For reactions that require heating, a silicon oil bath at the given temperatures was used. The water used was softened by an ion exchanger. All chemicals, whose synthesis is not exactly described were purchased commercially and used without any modification. 4,5-Dibromotoluene **1** was purchased from BLD Pharmatech GmbH.

NMR spectroscopy

Bruker Avance Neo 500 (^{1}H NMR: 500.1 MHz, ^{13}C NMR: 125.8 MHz) and Bruker Avance 600 (^{1}H NMR: 600.1 MHz, ^{13}C NMR: 150.9 MHz).

Unless otherwise stated, the NMR spectra were recorded at 298 K. Two-dimensional NMR spectra (COSY, HSQC, HMBC) were additionally measured. For the chemical shift (δ in ppm), the signal of the solvent was used as a reference. For the multiplicities of the ^{1}H NMR signals the following abbreviations were used: s (singlet), d (doublet), t (triplet), m (multiplet) and m_c (multiplet centered). Deuterated solvents were purchased from Eurisotop®.

**solvent**	**degree of deuteration / %**	**^{1}H NMR signal / ppm[1]**	**^{13}C NMR signal / ppm[1]**
CD_2Cl_2	99.80	5.32	53.84
$CDCl_3$	99.80	7.26	77.16
DMSO-d_6	99.80	2.50	39.52
THF-d_8	99.50	3.58, 1.72	67.21, 25.31

Mass spectrometry

Joel AccuTOF 4G using electron ionization (EI) at an ionization energy of 70 eV, Thermo Scientific Q Exactive Plus using electrospray ionization (ESI) and Bruker Autoflex Speed LRF using matrix assisted laser desorption ionization (MALDI).

IR spectroscopy

Perkin Elmer Spectrum100 using a Specac MKII Golden Gate Single Reflection Diamond ATR A531-G system. For the intensities of the absorption bands the following abbreviations were used: s (strong), m (medium), w (weak).

UV/vis spectroscopy

Shimadzu UV-2600i using a Lauda thermostat and Hellma high performance quartz glass cuvettes (optical path length of 10 mm, volume of 3500 µL).

Elemental analysis

Vario MICRO cube elemental analyzer from Elementar Analysensysteme GmbH.

Melting point determination

Büchi M-560 using melting point tubes. Melting points were only determined for crystalline products.

Thin layer chromatography

Macherey-Nagel silica gel coated Xtra SIL G/UV254 ALUGRAM® plates using a CAMAG UV lamp at wavelengths of 254 nm and 366 nm.

Automatic flash column chromatography

Biotage® Isolera™ One 3.0 using Interchim puriFlash® cartridges (PF-30SIHP-F0040, PF-30SIHP-F0025, PF-30SIHP-F0012).

Light sources

Custom-made light sources with wavelengths of 365 nm, 385 nm, 405 nm, 420 nm, 435 nm, 450 nm and 530 nm (Sahlmann Photochemical Solutions).

S 2 Syntheses and characterization

S 2.1 Synthesis of tetrabromo diazocine 5

2-Nitro-4,5-dibromotoluene (2)

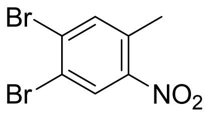

2

Under nitrogen atmosphere 4,5-dibromotoluene (**1**, 10.0 g, 40.3 mmol) was dissolved in dry dichloromethane (50 mL) and slowly added at -10 °C within 1 h to fuming nitric acid (19.1 g, 303 mmol). After complete addition, the reaction mixture was stirred at -10 °C for 1 h and then quenched with ice. The phases were separated, the aqueous phase was extracted three times with dichloromethane (50 mL each), the combined organic phases were dried over magnesium sulfate, filtered off, and the solvent was removed in vacuo. The crude product was recrystallized from ethanol. The product was obtained as a pale-yellow crystalline solid.

Yield: 7.25 g (24.6 mmol, 61 %).

^{1}H NMR (600 MHz, $CDCl_3$): δ = 8.24 (s, 1H), 7.65 (s, 1H), 2.55 (s, 3H) ppm.

^{13}C NMR (151 MHz, $CDCl_3$): δ = 148.0*, 137.4, 134.2, 130.7, 129.6, 122.7, 20.2 ppm. *The signal was detected by HMBC (see Figure S3).

HRMS (EI) *m/z*: $[M]^{\cdot+}$ Calcd for $^{12}C_7{}^{1}H_5{}^{79}Br_2{}^{14}N_1{}^{16}O_2$ 292.8687; Found 292.8686. Calcd for $^{12}C_7{}^{1}H_5{}^{79}Br_1{}^{81}Br_1{}^{14}N_1{}^{16}O_2$ 294.8667; Found 294.8666. Calcd for $^{12}C_7{}^{1}H_5{}^{81}Br_2{}^{14}N_1{}^{16}O_2$ 296.8646; Found 296.8646.

IR (ATR): $\tilde{\nu}$ = 3036 (w), 2976 (w), 1595 (m), 1548 (m), 1514 (s), 1446 (s), 1380 (m), 1088 (m), 1035 (m), 893 (s), 822 (s), 782 (w), 754 (s) cm^{-1}.

m.p.: 89 °C.

1,2-Bis(2-nitro-4,5-dibromophenyl)ethane (3a) + (*E,Z*)-1,2-Bis(2-nitro-4,5-dibromo- phenyl)ethene (3b)

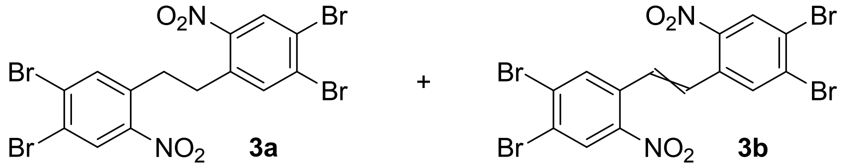

3a **3b**

Under nitrogen atmosphere 2-nitro-4,5-dibromotoluene (**2**, 3.32 g, 11.3 mmol) was dissolved in dry tetrahydrofuran (100 mL) and cooled to -10 °C. Potassium *tert*-butoxide (1.65 g, 14.7 mmol) was added in one portion, the reaction mixture was stirred for 30 s whereupon bromine (1.81 g, 11.3 mmol) was added. After complete addition, the reaction mixture was stirred at -10 °C for 10 min and then quenched with ice. The precipitate was filtered off, washed with the smallest possible amount of ice-cold dichloromethane and dried in vacuo. The crude product was obtained as an orange solid in which approx. 83 % of the desired product **3a** and approx. 17 % of a (*E,Z*)-stilbene derivative as the by-product **3b** were present (control via ^{1}H NMR). The inseparable crude product was obtained as an orange amorphous solid and used for the following reaction without further purification.

Yield: crude product: 2.23 g

main product **3a**: 1.85 g (3.15 mmol, 56 %).

by-product **3b**: 379 mg (647 µmol, 11 %).

Characterization of the main product **3a** by NMR spectroscopy:

^{1}H NMR (500 MHz, THF-d_8): δ = 8.32 (s, 2H), 7.82 (s, 2H), 3.20 (s, 4H) ppm.

^{13}C NMR (126 MHz, THF-d_8): δ = 149.5*, 137.9, 137.2, 131.1, 130.7, 124.0, 33.6 ppm.

*The signal was detected by HMBC (see Figure S5).

It was not possible to obtain high resolution mass spectra of compounds **3a** and **3b**, as the molecular peaks and their isotope patterns overlap due to the very small difference in molecular weight of both compounds. Both **3a** and **3b** are formed side by side during the dimerization of the monomer **2** and cannot be separated by column chromatography. Compounds **3a** and **3b** react upon treatment with tin dichloride dihydrate to form mainly the reduced desired product **4**, which can be characterized and isolated by column chromatography in pure form. Therefore, it is not necessary and practical to separate and characterize **3a** and **3b**, but rather to analyze the main product **4** at the next stage.

1,2-Bis(2-amino-4,5-dibromophenyl)ethane (4)

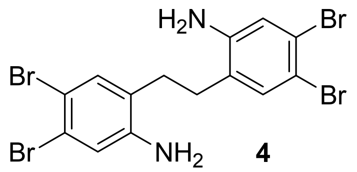

4

A crude mixture consisting of 1,2-bis(2-nitro-4,5-dibromophenyl)ethane (**3a**, 5.00 g, 8.51 mmol), (*E*,*Z*)-1,2-bis(2-nitro-4,5-dibromophenyl)ethene (**3b**, 1.02 g, 1.74 mmol)

and tin dichloride dihydrate (19.2 g, 85.1 mmol) were suspended in ethyl acetate (500 mL) and stirred under reflux for 18 h. The reaction mixture was cooled to room temperature and neutralized with a sodium hydroxide solution (15 wt%). The precipitate was filtered off, and the phases were separated. The aqueous phase was extracted three times with ethyl acetate (100 mL each), the combined organic phases were dried over magnesium sulfate, filtered off, and the solvent was removed in vacuo. The crude product was purified by column chromatography on silica gel (cyclohexane/ethyl acetate, 9:1 → 1:1). After removal of the solvent in vacuo the solid was additionally washed with the smallest possible amount of ice-cold ethyl acetate. The product was obtained as a pale-orange amorphous solid.

Yield: 3.81 g (7.22 mmol, 85 %).*

*Yield in relation to the amount of substance of **3a**.

^{1}H NMR (600 MHz, DMSO-*d*$_6$): δ = 7.32 (s, 2H), 6.97 (s, 2H), 5.41 (s, 4H), 2.57 (s, 4H) ppm.

^{13}C NMR (151 MHz, DMSO-*d*$_6$): δ = 147.4, 133.2, 126.6, 120.8, 118.1, 107.8, 28.3 ppm.

HRMS (EI) *m*/*z*: $[M]^{\cdot+}$ Calcd for $^{12}C_{14}{}^{1}H_{12}{}^{79}Br_4{}^{14}N_2$ 523.7734; Found 523.7739.

IR (ATR): $\tilde{\nu}$ = 3474 (m), 3387 (m), 2870 (w), 1614 (s), 1552 (m), 1473 (s), 1109 (m), 906 (s), 886 (s) cm^{-1}.

2,3,8,9-Tetrabromo-11,12-dihydrodibenzo[*c,g*][1,2]diazocin (5)

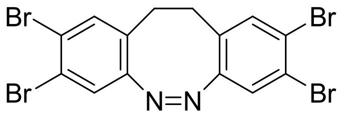

5

1,2-Bis(2-amino-4,5-dibromophenyl)ethane (**4**, 5.00 g, 9.47 mmol) was suspended in a mixture consisting of dichloromethane and glacial acetic acid (3:1, 237 mL) and stirred at room temperature. A solution of *meta*-chloroperoxybenzoic acid (3.27 g, 18.9 mmol) in glacial acetic acid (60 mL) was added dropwise over a period of 24 h using a syringe pump. The precipitate was filtered off, which was spectroscopically assigned to an inseperable mixture consisting of the desired tetrabromo diazocine **5** and the corresponding azoxy derivative. The filtrate was left untouched until further precipitate formed. The precipitate was filtered off, dried in vacuo and spectroscopically assigend to the desired product **5** in pure form. The product was obtained as a yellow amorphous solid.

Yield: 1.14 g (2.18 mmol, 23 %).

^{1}H NMR (600 MHz, THF-*d*$_8$): δ = 7.45 (s, 2H), 7.24 (s, 2H), 2.85 – 2.81 (m, 4H) ppm.

^{13}C NMR (151 MHz, THF-*d*$_8$): δ = 156.2, 135.7, 130.4, 125.1, 123.8, 123.4, 31.3 ppm.

HRMS (EI) *m*/*z*: $[M]^{\cdot+}$ Calcd for $^{12}C_{14}{}^{1}H_{8}{}^{79}Br_4{}^{14}N_2$ 519.7421; Found 519.7423.

IR (ATR): $\tilde{\nu}$ = 2928 (w), 2225 (w), 2191 (w), 1615 (w), 1573 (w), 1518 (w), 1455 (s), 1111 (s), 885 (s), 877 (s), 853 (m), 832 (m) cm^{-1}.

S 2.2 Synthesis of compound 32

3,4,5-Tris(dodecyloxy)benzyl-2-bromoacetate (32)

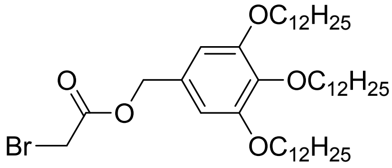

32

Under nitrogen atmosphere a solution of 2-bromoacetyl bromide (735 mg, 3.64 mmol) in dry dichloromethane (10 mL) was slowly added at -10 °C to 3,4,5-tris(dodecyloxy)benzyl alcohol (**33**, 2.00 g, 3.03 mmol), triethylamine (368 mg, 3.64 mmol) and 4-dimethylaminopyridine (37.0 mg, 303 µmol), dissolved in dry dichloromethane (20 mL). After complete addition, the reaction mixture was slowly allowed to warm up to room temperature and stirred over night. Water (100 mL) was added to the reaction mixture and the phases were separated. The aqueous phase was extracted three times with dichloromethane (50 mL each), the combined organic phases were dried over magnesium sulfate, filtered off, and the solvent was removed in vacuo. The crude product was purified by column chromatography on silica gel (*n*-pentane/dichloromethane, 9:1 → 1:1). The product was obtained as a colorless oil, which precipitated as a colorless amorphous solid.

Yield: 1.61 g (2.06 mmol, 68 %).

^{1}H NMR (500 MHz, $CDCl_3$): δ = 6.55 (s, 2H), 5.10 (s, 2H), 3.98 – 3.93 (m, 6H), 3.87 (s, 2H), 1.82 – 1.76 (m, 4H), 1.75 – 1.70 (m, 2H), 1.49 – 1.43 (m, 6H), 1.34 – 1.26 (m, 48H), 0.89 – 0.87 (m, 9H) ppm.

^{13}C NMR (121 MHz, $CDCl_3$): δ = 167.2, 153.4, 138.6, 130.0, 107.2, 73.6, 69.3, 68.4, 32.1, 30.5, 29.91, 29.85, 29.80, 29.76, 29.6, 29.5, 26.27, 26.25, 26.1, 22.9, 14.3 ppm. The number of ^{13}C NMR signals does not equal the number of non-symmetry equivalent carbon atoms probably because of superposition of signals.

HRMS (EI) *m/z*: $[M]^{\cdot+}$ Calcd for $^{12}C_{45}{}^{1}H_{81}{}^{79}Br_1{}^{16}O_5$ 780.5267; Found 780.5263. Calcd for $^{12}C_{45}{}^{1}H_{81}{}^{81}Br_1{}^{81}Br_1{}^{16}O_5$ 782.5247; Found: 782.5255.

IR (ATR): $\tilde{\nu}$ = 2916 (s), 2849 (s), 1732 (m), 1592 (m), 1509 (m), 1467 (m), 1440 (m), 1372 (w), 1276 (s), 1119 (s), 1031 (w), 884 (w), 750 (w), 721 (m), 688 (w), 627 (w) $cm^{-1.}$

3,4,5-Tris(dodecyloxy)benzyl alcohol (33)

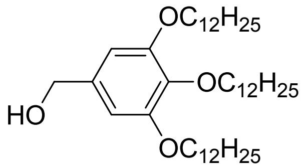

33

Synthesis and characterisation according to literature[2].

S 2.3 Synthesis of stannafluorenes 13 – 17

1,1'-Biphenyl-3,3'-diol (6)

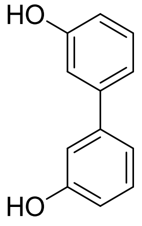

6

Synthesis and characterisation according to literature[3].

9,9-Dimethyl-9-stannafluorene (17)

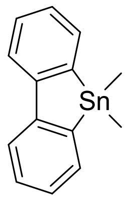

17

Synthesis and characterisation according to literature[4].

1,1'-Biphenyl-3,3'-dimethoxy (7)

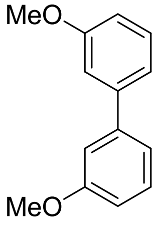

7

1,1'-Biphenyl-3,3'-diol (**6**, 500 mg, 2.69 mmol), iodomethane (1.15 g, 8.07 mmol) and potassium carbonate (2.23 g, 16.1 mmol) were placed in acetone (50 mL) and stirred at 70 °C for 15 h. Iodomethane (2.28 g, 17.3 mmol) was then added again and the reaction mixture was stirred at 70 °C for 6 h. The suspension was filtered, and the solvent was removed in vacuo. The crude product was purified by column chromatography on silica gel (cyclohexane/ethyl acetate, 9:1 → 7:3). The product was obtained as a colorless amorphous solid.

Yield: 570 mg (2.66 mmol, 99 %).

^{1}H NMR (500 MHz, $CDCl_3$): δ = 7.37 – 7.34 (m, 2H), 7.18 (ddd, J = 7.7 Hz, 1.7 Hz, 1.0 Hz, 2H), 7.13 (dd, J = 2.4 Hz, 1.8 Hz, 2H), 6.91 (ddd, J = 8.2 Hz, 2.6 Hz, 0.9 Hz, 2H), 3.87 (s, 6H) ppm.

^{13}**C NMR** (126 MHz, $CDCl_3$): δ = 160.0, 142.8, 129.9, 119.9, 113.1, 113.0, 55.5 ppm.

HRMS (EI) *m/z*: $[M]^{\cdot+}$ Calcd for $^{12}C_{14}{}^{1}H_{14}{}^{16}O_2$ 214.0994; Found: 214.0994.

IR (ATR): $\tilde{\nu}$ = 2960 (w), 2835 (w), 1583 (s), 1485 (s), 1462 (s), 1444 (s), 1413 (s), 1324 (s), 1157 (s), 1089 (m), 857 (s), 806 (s), 796 (s), 776 (s), 706 (m) cm^{-1}.

1,1'-Biphenyl-2,2'-dibromo-5,5'-dimethoxy (8)

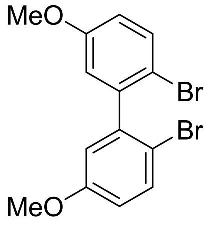

8

Under nitrogen atmosphere *N*-bromosuccinimide (12.8 g, 71.9 mmol) was dissolved in dry acetonitrile (100 mL) and slowly added within 1 h at -10 °C to 1,1'-biphenyl-3,3'-dimethoxy (7.70 g, 35.9 mmol), dissolved in dry acetonitrile (50 mL). After complete addition, the reaction mixture was slowly allowed to warm up to room temperature and stirred over night. The reaction mixture was quenched with ice, the precipitated solid was filtered off, dried in vacuo and recrystallized from ethanol. The product was obtained as a colorless crystalline solid.

Yield: 11.1 g (29.8 mmol, 83 %).

^{1}**H NMR** (500 MHz, $CDCl_3$): δ = 7.54 – 7.52 (m, 2H), 6.84 – 6.81 (m, 2H), 6.80 – 6.79 (m, 2H), 3.81 (s, 6H) ppm.

^{13}**C NMR** (126 MHz, $CDCl_3$): δ = 158.7, 142.9, 133.3, 116.4, 115.6, 113.9, 55.7 ppm.

HRMS (EI) *m/z*: $[M]^{\cdot+}$ Calcd for $^{12}C_{14}{}^{1}H_{12}{}^{79}Br_2{}^{16}O_2$ 369.9204; Found 369.9203. Calcd for $^{12}C_{14}{}^{1}H_{12}{}^{79}Br_1{}^{81}Br_1{}^{16}O_2$ 371.9184; Found 371.9183. Calcd for $^{12}C_{14}{}^{1}H_{12}{}^{81}Br_2{}^{16}O_2$ 373.9163; Found 373.9165.

IR (ATR): $\tilde{\nu}$ = 2957 (w), 2909 (w), 1593 (m), 1482 (m), 1458 (s), 1435 (m), 1420 (s), 1299 (m), 1139 (m), 1057 (w), 888 (m), 871 (s), 806 (s) cm^{-1}.

m.p.: 132 °C.

1,1‘-Biphenyl-2,2‘-dibromo-5,5‘-diol (9)

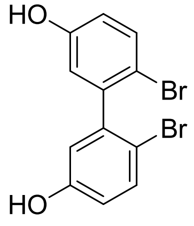

9

Under nitrogen atmosphere boron tribromide (1.0 M in dichloromethane, 25.3 g, 101 mmol) was slowly added within 1 h at -78 °C to 1,1’-biphenyl-2,2’-dibromo-5,5’-dimethoxy (12.5 g, 33.6 mmol), dissolved in dry dichloromethane (300 mL). After complete addition, the reaction mixture was slowly allowed to warm up to room temperature and stirred over night. The reaction mixture was quenched with ice, the precipitated solid was filtered off and dissolved in ethyl acetate (200 mL). The organic phase was washed three times with saturated sodium chloride solution (100 mL each), dried over magnesium sulfate, filtered off, and the solvent was removed in vacuo. The product was obtained as a colorless amorphous solid.

Yield: 11.3 g (32.8 mmol, 98 %).

^{1}H NMR (500 MHz, DMSO-d_6): δ = 9.83 (s, 2H), 7.44 (d, J = 8.7 Hz, 2H), 6.74 (dd, J = 8.7 Hz, 2.9 Hz, 2H), 6.61 (d, J = 2.9 Hz, 2H) ppm.

^{13}C NMR (126 MHz, DMSO-d_6): δ = 156.6, 142.3, 133.0, 117.6, 117.0, 110.9 ppm.

HRMS (EI) *m*/*z*: $[M]^{\cdot+}$ Calcd for $^{12}C_{12}{}^{1}H_{8}{}^{79}Br_{2}{}^{16}O_{2}$ 341.8891; Found 341.8890. Calcd for $^{12}C_{12}{}^{1}H_{8}{}^{79}Br_{1}{}^{81}Br_{1}{}^{16}O_{2}$ 343.8871; Found 343.8871. Calcd for $^{12}C_{12}{}^{1}H_{8}{}^{81}Br_{2}{}^{16}O_{2}$ 345.8850; Found 345.8852.

IR (ATR): $\tilde{\nu}$ = 3177 (br), 1588 (w), 1493 (w), 1449 (s), 1039 (m), 857 (s), 809 (s), 722 (w), 713 (s) cm^{-1}.

1,1’-Biphenyl-2,2’-dibromo-5,5’-di(*tert*-butyldimethylsilyloxy) (10)

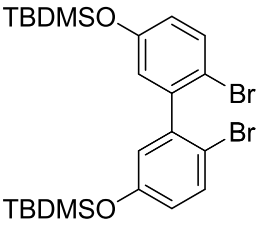

10

1,1‘-Biphenyl-2,2‘-dibromo-5,5‘-diol (1.00 g, 2.91 mmol), *tert*-butyldimethylsilyl chloride (1.31 g, 8.72 mmol) and imidazole (987 mg, 14.5 mmol) were placed in dichloromethane (50 mL) and stirred at room temperature for 13 h. Water (100 mL) was added to the reaction mixture and the phases were separated. The organic phase was washed once with saturated sodium chloride solution (100 mL), dried over magnesium sulfate, filtered off, and the solvent was removed in vacuo. The crude product was purified by column chromatography on silica gel (cyclohexane/ethyl

acetate, 1:0 → 8:2). The product was obtained as a colorless wax, which precipitated as a colorless amorphous solid.

Yield: 1.65 g (2.88 mmol, 99 %).

^{1}H NMR (500 MHz, $CDCl_3$): δ = 7.48 – 7.46 (m, 2H), 6.76 – 6.72 (m, 4H), 0.98 (s, 18H), 0.21 (s, 6H), 0.20 (s, 6H) ppm.

^{13}C NMR (126 MHz, $CDCl_3$): δ = 154.9, 142.8, 133.3, 122.9, 121.6, 114.7, 25.8, 18.4, -4.26, -4.35 ppm.

HRMS (EI) *m/z*: $[M]^{\cdot+}$ Calcd for $^{12}C_{24}{}^{1}H_{36}{}^{79}Br_2{}^{16}O_2{}^{28}Si_2$ 570.0621; Found: 570.0621. Calcd for $^{12}C_{24}{}^{1}H_{36}{}^{79}Br_1{}^{81}Br_1{}^{16}O_2{}^{28}Si_2$ 572.0600; Found 570.0602. Calcd for $^{12}C_{24}{}^{1}H_{36}{}^{79}Br_2{}^{16}O_2{}^{29}Si_2$ 572.0612; Found 572.0602. Calcd for $^{12}C_{24}{}^{1}H_{36}{}^{79}Br_2{}^{16}O_2{}^{28}Si_1{}^{29}Si_1$ 572.0589; Found 572.0602. Calcd for $^{12}C_{24}{}^{1}H_{36}{}^{79}Br_1{}^{81}Br_1{}^{16}O_2{}^{29}Si_2$ 574.0592; Found 574.0588. Calcd for $^{12}C_{24}{}^{1}H_{36}{}^{81}Br_2{}^{16}O_2{}^{28}Si_2$ 574.0580; Found 574.0588. Calcd for $^{12}C_{24}{}^{1}H_{36}{}^{79}Br_1{}^{81}Br_1{}^{16}O_2{}^{28}Si_1{}^{29}Si_1$ 574.0569; Found 574.0588.

IR (ATR): $\tilde{\nu}$ = 2955 (m), 2886 (w), 1592 (w), 1480 (s), 1361 (m), 1285 (s), 1253 (s), 1178 (s), 1085 (w), 939 (m), 899 (s), 825 (s) cm^{-1}.

1,1'-Biphenyl-2,2'-dibromo-5,5'-divinyloxy (11)

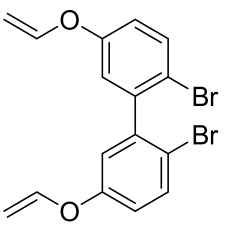

11

Under nitrogen atmosphere 1,1'-biphenyl-2,2'-dibromo-5,5'-diol (**9**, 500 mg, 1.45 mmol), vinyl acetate (500 mg, 5.81 mmol), bis(1,5-cyclooctadiene)diiridium(I) dichloride (19.5 mg, 29.1 µmol) and anhydrous sodium carbonate (185 mg, 1.74 mmol) were placed in dry toluene (20 mL) and stirred at 100 °C for 22 h. Diethyl ether (50 mL) was added to the reaction mixture and the solvent was removed in vacuo. The crude product was purified by column chromatography on silica gel (cyclohexane/ethyl acetate, 9:1 → 3:1). The product was obtained as a yellow oil, which precipitated as a beige amorphous solid.

Yield: 460 mg (1.16 mmol, 80 %).

^{1}H NMR (500 MHz, $CDCl_3$): δ = 7.59 (dd, *J* = 8.7 Hz, 0.4 Hz, 2H), 6.94 – 6.92 (m, 2H), 6.90 – 6.89 (m, 2H), 6.62 (dd, *J* = 13.7 Hz, 6.1 Hz, 2H), 4.82 (dd, *J* = 13.7 Hz, 1.8 Hz, 2H), 4.49 (dd, *J* = 6.1 Hz, 1.9 Hz, 2H) ppm.

^{13}C NMR (126 MHz, $CDCl_3$): δ = 155.9, 147.7, 142.7, 133.7, 119.5, 118.5, 116.6, 96.5 ppm.

HRMS (EI) *m/z*: $[M]^{\cdot+}$ Calcd for $^{12}C_{16}{}^{1}H_{12}{}^{79}Br_2{}^{16}O_2$ 393.9204; Found 393.9205. Calcd for $^{12}C_{16}{}^{1}H_{12}{}^{79}Br_1{}^{81}Br_1{}^{16}O_2$ 395.9184; Found 395.9185. Calcd for $^{12}C_{16}{}^{1}H_{12}{}^{81}Br_2{}^{16}O_2$ 397.9163; Found 397.9167.

IR (ATR): $\tilde{\nu}$ = 3070 (w), 2923 (w), 2853 (w), 1641 (s), 1586 (m), 1568 (s), 1481 (m), 1465 (s), 1139 (s), 1127 (m), 1038 (m), 986 (m), 908 (m), 869 (s), 830 (m), 818 (s) cm^{-1}.

1,1'-Biphenyl-2,2'-dibromo-5,5'-dioctyloxy (12)

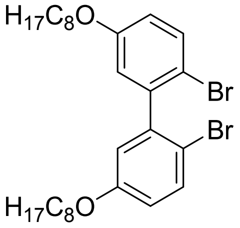

12

1,1'-Biphenyl-2,2'-dibromo-5,5'-diol (1.00 g, 2.91 mmol), 1-octyl-*p*-toluenesulfonate (2.48 g, 7.73 mmol) and cesium carbonate (5.70 g, 17.5 mmol) were placed in acetone (100 mL) and stirred under reflux for 13 h. The suspension was filtered, and the solvent was removed in vacuo. The crude product was purified by column chromatography on silica gel (cyclohexane/ethyl acetate, 1:0 → 8:2). The product was obtained as a colorless oil, which precipitated as a colorless amorphous solid.

Yield: 1.44 g (2.53 mmol, 87 %).

^{1}H NMR (600 MHz, $CDCl_3$): δ = 7.51 (d, J = 8.7 Hz, 2H), 6.82 – 6.78 (m, 4H), 3.93 (t, J = 6.6 Hz, 4H), 1.79 – 1.75 (m, 4H), 1.46 – 1.41 (m, 4H), 1.36 – 1.25 (m, 16H), 0.89 – 0.87 (m, 6H) ppm.

^{13}C NMR (151 MHz, $CDCl_3$): δ = 158.3, 142.9, 133.2, 116.9, 116.2, 113.6, 68.5, 32.0, 29.5, 29.4, 29.3, 26.1, 22.8, 14.3.

HRMS (EI) *m*/*z*: $[M]^{\cdot+}$ Calcd for $^{12}C_{28}{}^{1}H_{40}{}^{79}Br_2{}^{16}O_2$ 566.1395; Found 566.1395. Calcd for $^{12}C_{28}{}^{1}H_{40}{}^{79}Br_1{}^{81}Br_1{}^{16}O_2$ 568.1375; Found 568.1374. Calcd for $^{12}C_{28}{}^{1}H_{40}{}^{81}Br_2{}^{16}O_2$ 570.1354; Found 570.1359.

IR (ATR): $\tilde{\nu}$ = 2954 (m), 2924 (s), 2870 (m), 2852 (m), 1597 (m), 1585 (m), 1486 (m), 1464 (s), 1404 (m), 1392 (s), 1124 (m), 1095 (w), 1048 (m), 860 (m), 836 (m), 808 (s) cm^{-1}.

General procedure for the synthesis of 9,9-dimethyl-9-stannafluorenes 13 – 16

Caution: Organic stannates are highly toxic and require particularly careful handling.

Under nitrogen atmosphere *n*-butyllithium (1.6 M in *n*-hexane, 2.00 mmol, 2.00 eq.) was slowly added at -78 °C to corresponding 1,1'-biphenyl-2,2'-dibromides (**8**, **10** – **12**, 1.00 mmol, 1.00 eq.), dissolved in dry diethyl ether (35 mL). After complete addition the reaction mixture was allowed to warm up to room temperature and stirred for 3 h. Dimethyltin dichloride (1.00 mmol, 1.00 eq.) was dissolved in dry diethyl ether (9 mL), slowly added to the reaction mixture and stirred over night. The solvent was removed in vacuo and *n*-hexane (100 mL) was added to the residue. The mixture was sonicated for 15 min, the resulting suspension was filtered through celite® and the solvent was removed in vacuo. The waxy residue was carefully overlaid with a small amount of *n*-hexane and stored in the freezer over night. The sticky solid obtained was filtered off

and reprecipitated from *n*-hexane under careful heating and subsequent cooling. The product was obtained as a colorless sticky and amorphous solid.

3,6-Di(*tert*-butyldimethylsilyloxy)-9,9-dimethyl-9-stannafluorene (13)

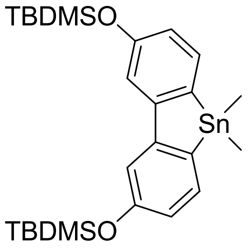

13

n-Butyllithium (1.6 M in *n*-hexane, 448 mg, 6.99 mmol), 1,1'-biphenyl-2,2'-dibromo-5,5'-di(*tert*-butyldimethylsilyloxy) (**10**, 2.00 g, 3.49 mmol) dissolved in dry diethyl ether (122 mL), dimethyltin dichloride (767 mg, 3.49 mmol) dissolved in dry diethyl ether (31 mL).

Yield: 1.21 g (2.16 mmol, 62 %).

^{1}H NMR (600 MHz, $CDCl_3$): *δ* = 7.47 (d, *J* = 7.6 Hz, 2H), 7.33 (d, *J* = 2.3 Hz, 2H), 6.79 (dd, *J* = 7.7 Hz, 2.2 Hz, 2H), 1.02 (s, 18H), 0.49 (s, 6H), 0.25 (s, 12H) ppm.

^{13}C NMR (151 MHz, $CDCl_3$): *δ* = 157.2, 150.1, 137.0, 133.0, 119.8, 114.7, 25.9, 18.5, -4.11, -8.20 ppm.

HRMS (EI) *m*/*z*: $[M]^{\cdot +}$ Calcd for $^{12}C_{26}{}^{1}H_{42}{}^{16}O_{2}{}^{29}Si_{2}{}^{118}Sn_{1}$ 562.1731; Found 562.1730. Calcd for $^{12}C_{26}{}^{1}H_{42}{}^{16}O_{2}{}^{29}Si_{1}{}^{30}Si_{1}{}^{117}Sn_{1}$ 562.1717; Found 562.1730. Calcd for $^{12}C_{26}{}^{1}H_{42}{}^{16}O_{2}{}^{28}Si_{2}{}^{120}Sn_{1}$ 562.1745; Found 562.1730. Calcd for $^{12}C_{26}{}^{1}H_{42}{}^{16}O_{2}{}^{28}Si_{1}{}^{30}Si_{1}{}^{118}Sn_{1}$ 562.1708; Found 562.1730. Calcd for $^{12}C_{26}{}^{1}H_{42}{}^{16}O_{2}{}^{28}Si_{1}{}^{29}Si_{1}{}^{119}Sn_{1}$ 562.1752; Found 562.1730.

Due to toxicity and stickiness of the compound **13**, the IR spectrum could not be measured.

3,6-Divinyloxy-9,9-dimethyl-9-stannafluorene (14)

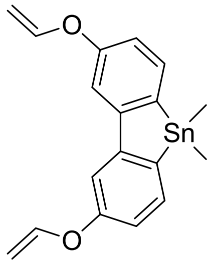

14

n-Butyllithium (1.6 M in *n*-hexane, 652 mg, 10.1 mmol), 1,1'-biphenyl-2,2'-dibromo-5,5'-divinyloxy (**11**, 2.00 g, 5.05 mmol) dissolved in dry diethyl ether (177 mL), dimethyltin dichloride (1.11 g, 5.05 mmol) dissolved in dry diethyl ether (45 mL). The

inseparable crude product was obtained as a colorless oil and used for the following reaction without further purification.

HRMS (EI) *m*/*z*: $[M]^{\cdot +}$ Calcd for $^{12}C_{18}{}^{1}H_{18}{}^{16}O_{2}{}^{120}Sn_{1}$ 386.0329; Found 386.0329.

3,6-Dioctyloxy-9,9-dimethyl-9-stannafluorene (15)

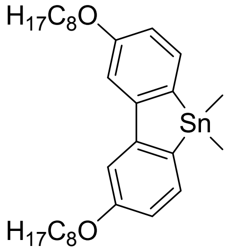

15

n-Butyllithium (1.6 M in *n*-hexane, 451 mg, 7.04 mmol), 1,1'-biphenyl-2,2'-dibromo-5,5'-dioctyloxy (**12**, 2.00 g, 3.52 mmol) dissolved in dry diethyl ether (123 mL), dimethyltin dichloride (773 mg, 3.52 mmol) dissolved in dry diethyl ether (32 mL). The inseparable crude product was obtained as a colorless oil and used for the following reaction without further purification.

HRMS (EI) *m*/*z*: $[M]^{\cdot +}$ Calcd for $^{12}C_{30}{}^{1}H_{46}{}^{16}O_{2}{}^{120}Sn_{1}$ 558.2520; Found 558.2521.

3,6-Dimethoxy-9,9-dimethyl-9-stannafluorene (16)

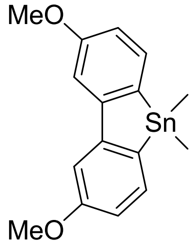

16

n-Butyllithium (1.6 M in *n*-hexane, 690 mg, 10.8 mmol), 1,1'-biphenyl-2,2'-dibromo-5,5'-dimethoxy (**8**, 2.00 g, 5.38 mmol) dissolved in dry diethyl ether (188 mL), dimethyltin dichloride (1.17 g, 5.38 mmol) dissolved in dry diethyl ether (48 mL).

Yield: 422 mg (1.17 mmol, 43 %).

^{1}H NMR (500 MHz, $CDCl_3$): δ = 7.56 (d, J = 7.8 Hz, 2H), 7.47 (d, J = 2.4 Hz, 2H), 6.88 (dd, J = 7.8 Hz, 2.4 Hz, 2H), 3.89 (s, 6H), 0.50 (s, 6H) ppm.

^{13}C NMR (126 MHz, $CDCl_3$): δ = 161.2, 150.0, 137.1, 132.4, 113.4, 109.3, 55.4, -8.27 ppm.

HRMS (EI) *m*/*z*: $[M]^{\cdot +}$ Calcd for $^{12}C_{16}{}^{1}H_{18}{}^{16}O_{2}{}^{120}Sn_{1}$ 362.0329; Found 362.0330.

Due to toxicity and stickiness of the compound **16**, the IR spectrum could not be measured.

S 2.4 Synthesis of triphenylene diazocines 18 – 31

General procedure for the synthesis of triphenylene diazocines 18 – 22 by multiple Stille cross-coupling reaction

Under nitrogen atmosphere 2,3,8,9-tetrabromo-11,12-dihydrodibenzo[*c,g*][1,2]-diazocine (**5**, 1.00 mmol, 1.00 eq.), corresponding 9,9-dimethyl-9-stannafluorenes (**13** – **17**, 3.00 mmol, 3.00 eq.) and bis(tri-*tert*-butylphosphine)palladium(0) (100 µmol, 0.1 eq.) were placed in in dry tetrahydrofuran (volume used see procedure of each compound) and stirred at 60 °C for 2 d.

Purification method A:

The reaction mixture was filtered through celite® and purified by flash column chromatography on silica gel (cyclohexane/ethyl acetate 1:0 → 8:2). The rack with the filled test tubes was placed in the refrigerator until a yellow precipitate formed, which was filtered off and dried in vacuo. The filtrate was combined together with the remaining product fraction and the solvent was removed in vacuo. The residue was reprecipitated from cyclohexane/ethyl acetate or dichloromethane/methanol until no more yellow solid was formed. The filtered solids were combined and dried in vacuo. The product was obtained as a yellow amorphous solid.

Purification method B:

The precipitate was filtered off, washed with tetrahydrofuran and dried in vacuo. The product was obtained as a yellow amorphous solid.

Tetrakis(*tert*-butyldimethylsilyloxy)triphenylene diazocine (18)

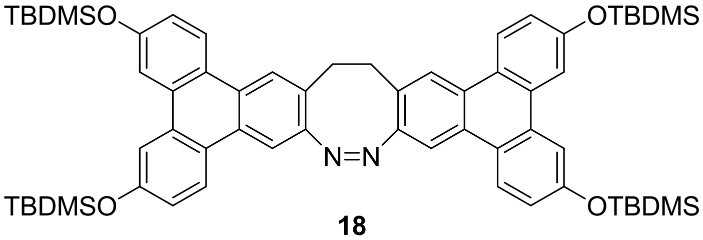

18

2,3,8,9-Tetrabromo-11,12-dihydrodibenzo[*c,g*][1,2]-diazocine (**5**, 4.11 g, 7.84 mmol), 3,6-di(*tert*-butyldimethylsilyloxy)-9,9-dimethyl-9-stannafluorene (**13**, 13.2 g, 23.5 mmol), bis(tri-*tert*-butylphosphine)palladium(0) (401 mg, 784 µmol), dry tetrahydrofuran (200 mL), purification method A (reprecipitation from cyclohexane/ethyl acetate).

Yield: 4.92 g (4.78 mmol, 61 %).

^{1}H NMR (500 MHz, $CDCl_3$): δ = 8.33 (d, *J* = 8.9 Hz, 2H), 8.31 (d, *J* = 8. Hz, 2H), 8.10 (s, 2H), 7.96 (s, 2H), 7.76 – 7.74 (m, 4H), 7.10 (dd, *J* = 8.9 Hz, 2.5 Hz, 2H), 7.08 (dd, *J* = 8.9 Hz, 2.5 Hz, 2H), 3.33 – 3.15 (m_c, 4H), 1.01 (s, 18H), 1.00 (s, 18H), 0.242 (s, 12H), 0.236 (s, 12H) ppm.

^{13}C NMR (126 MHz, $CDCl_3$): δ = 155.3, 155.2, 154.5, 131.0, 128.1, 128.0, 127.5, 125.1, 124.9, 124.1, 124.0, 123.9, 121.12, 121.06, 113.3, 113.2, 113.1, 32.4, 25.9, 18.5, -4.14 ppm.

The number of ^{13}C NMR signals does not equal the number of non-symmetry equivalent carbon atoms probably because of superposition of signals.

HRMS (ESI) *m/z*: $[M+H]^+$ Calcd for $C_{62}H_{81}O_4N_2Si_4$ 1029.5268; Found 1029.5269.

IR (ATR): $\tilde{\nu}$ = 2954 (m), 2929 (m), 2857 (m), 1738 (m), 1611 (s), 1575 (m), 1508 (s), 1486 (w), 1461 (m), 1424 (m), 1356 (m), 1299 (m), 1250 (w), 1234 (s), 1207 (s), 1117 (m), 1081 (w), 917 (s), 834 (s), 810 (s), 779 (s) cm^{-1}.

Tetrakis(vinyloxy)triphenylene diazocine (19)

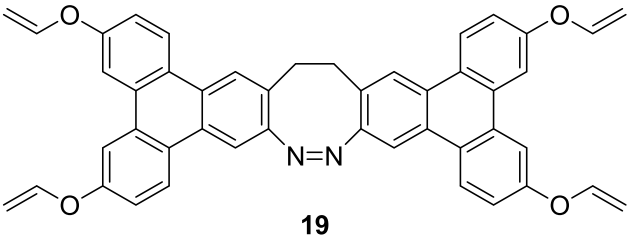

19

2,3,8,9-Tetrabromo-11,12-dihydrodibenzo[*c,g*][1,2]-diazocine (**5**, 230 mg, 440 µmol), 3,6-divinyloxy-9,9-dimethyl-9-stannafluorene (**14**, 508 mg, 1.32 mmol), bis(tri-*tert*-butylphosphine)palladium(0) (22.5 mg, 44.0 µmol), dry tetrahydrofuran (20 mL), purification method A (reprecipitation from cyclohexane/ethyl acetate).

Yield: 28.3 mg (41.8 µmol, 10 %).

^{1}H NMR (500 MHz, CD_2Cl_2): δ = 8.44 (d, *J* = 9.1 Hz, 2H), 8.41 (d, *J* = 9.1 Hz, 2H), 8.19 (s, 2H), 8.01 (s, 2H), 7.95 – 7.91 (m, 4H), 7.28 (t, *J* = 2.3 Hz, 2H), 7.27 (t, *J* = 2.4 Hz, 2H), 6.82 – 6.77 (m, 4H), 4.85 – 4.82 (m, 4H), 4.54 – 4.53 (m, 4H), 3.33 – 3.21 (m_c, 4H) ppm.

^{13}C NMR (126 MHz, $CDCl_3$): δ = 156.6, 156.5, 155.3, 148.4, 131.2, 131.1, 128.6, 128.1, 125.7, 125.6, 125.5, 125.4, 124.7, 118.2, 113.6, 110.5, 96.1, 32.5 ppm.

The number of ^{13}C NMR signals does not equal the number of non-symmetry equivalent carbon atoms probably because of superposition of signals.

HRMS (EI) *m/z*: $[M]^{\cdot+}$ Calcd for $^{12}C_{46}{}^{1}H_{32}{}^{14}N_2{}^{16}O_4$ 676.2362; Found 676.2359.

IR (ATR): $\tilde{\nu}$ = 3055 (w), 2943 (w), 2145 (w), 1644 (m), 1611 (s), 1576 (m), 1537 (m), 1506 (m), 1488 (m), 1463 (m), 1142 (s), 1039 (w), 1006 (m), 953 (m), 905 (w), 843 (s), 809 (s) cm^{-1}.

Tetrakis(octyloxy)triphenylene diazocine (20)

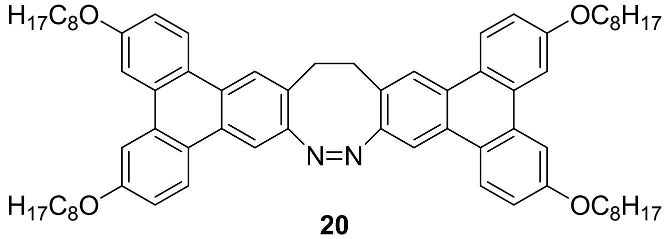

20

2,3,8,9-Tetrabromo-11,12-dihydrodibenzo[*c,g*][1,2]-diazocine (**5**, 361 mg, 689 µmol), 3,6-bis(octyloxy)-9,9-dimethyl-9-stannafluorene (**15**, 1.15 g, 2.07 mmol), bis(tri-*tert*-butylphosphine)palladium(0) (35.2 mg, 68.9 µmol), dry tetrahydrofuran (20 mL), purification method A (reprecipitation from dichloromethane/methanol).

Yield: 574 mg (561 µmol, 81 %).

^{1}H NMR (600 MHz, $CDCl_3$): δ = 8.37 (d, *J* = 7.9 Hz, 2H), 8.35 (d, *J* = 8.0 Hz, 2H), 8.11 (s, 2H), 7.97 (s, 2H), 7.81 – 7.80 (m, 4H), 7.17 (dd, *J* = 9.1 Hz, 2.4 Hz, 2H), 7.15 (dd, *J* = 9.1 Hz, 2.4 Hz, 2H), 4.11 – 4.09 (m, 8H), 3.32 – 3.15 (m_c, 4H), 1.86 – 1.81 (m, 8H), 1.52 – 1.47 (m, 8H), 1.40 – 1.35 (m, 8H), 1.348 – 1.25 (m, 24H), 0.89 – 0.87 (m, 12H) ppm.

^{13}C NMR (151 MHz, $CDCl_3$): δ = 158.6, 158.5, 154.4, 131.14, 131.12, 128.0, 127.9, 127.3, 125.1, 124.9, 123.9, 123.6, 123.3, 116.1, 116.0, 113.3, 107.2, 107.1, 68.43, 68.39, 32.4, 32.0, 29.6, 29.5, 29.4, 26.2, 22.8, 14.2 ppm.

The number of ^{13}C NMR signals does not equal the number of non-symmetry equivalent carbon atoms probably because of superposition of signals.

HRMS (EI) *m*/*z*: $[M]^{\cdot+}$ Calcd for $^{12}C_{70}{}^{1}H_{92}{}^{14}N_2{}^{16}O_4$ 1024.7057; Found 1024.7050.

IR (ATR): $\tilde{\nu}$ = 3032 (w), 2920 (s), 2852 (s), 2211 (w), 1739 (w), 1614 (s), 1575 (w), 1537 (w), 1511 (m), 1463 (m), 1428 (m), 1356 (m), 1147 (w), 1121 (w), 856 (w), 834 (m), 808 (s) cm^{-1}.

Tetrakis(methoxy)triphenylene diazocine (21)

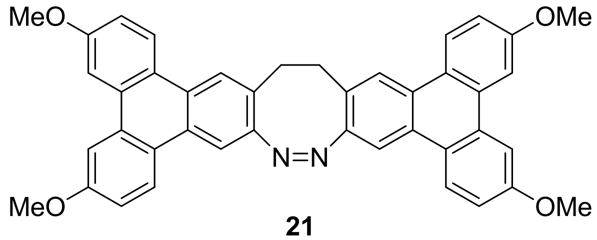

21

2,3,8,9-Tetrabromo-11,12-dihydrodibenzo[*c,g*][1,2]-diazocine (**5**, 113 mg, 216 µmol), 3,6-Bis(methoxy)-9,9-dimethyl-9-stannafluorene (**16**, 234 mg, 648 µmol), bis(tri-*tert*-butylphosphine)palladium(0) (11.0 mg, 21.6 µmol), dry tetrahydrofuran (20 mL), purification method B

Yield: 91.3 mg (145 µmol, 67 %).

^{1}**H NMR** (600 MHz, DMSO-*d*$_6$): δ = 8.58 (d, *J* = 9.3 Hz, 2H), 8.55 (d, *J* = 9.3 Hz, 2H), 8.41 (s, 2H), 8.11 (s, 2H), 8.02 – 8.01 (m, 4H), 7.25 – 7.23 (m, 4H), 3.94 (s, 12H), 3.30 – 3.14 (m, 4H)* ppm.

*The signal is partially superimposed by the signal of water (see Figure S35).

^{13}**C NMR** (151 MHz, DMSO-*d*$_6$): δ = 158.9, 158.8, 154.3, 130.6, 130.4, 127.6, 127.2, 127.0, 125.4, 125.3, 124.2, 122.7, 122.6, 116.3, 116.2, 112.5, 106.4, 106.3, 55.5, 31.1 ppm.

The number of ^{13}C NMR signals does not equal the number of non-symmetry equivalent carbon atoms probably because of superposition of signals.

HRMS (EI) *m/z*: $[M]^{\cdot+}$ Calcd for $^{12}C_{42}{}^{1}H_{32}{}^{14}N_2{}^{16}O_4$ 628.2362; Found 628.2364.

IR (ATR): $\tilde{\nu}$ = 2932 (w), 2828 (w), 1614 (s), 1579 (m), 1537 (w), 1510 (m), 1463 (s), 1417 (m), 1365 (m), 1139 (w), 878 (m), 836 (s), 812 (s) cm^{-1}.

Triphenylene diazocine (22)

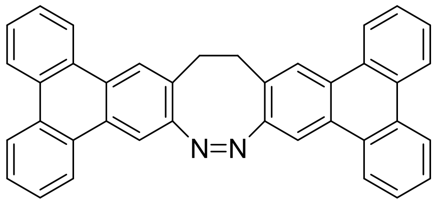

22

2,3,8,9-Tetrabromo-11,12-dihydrodibenzo[*c,g*][1,2]-diazocine (**5**, 55.0 mg, 105 µmol), 9,9-Dimethyl-9-stannafluorene (**17**, 95.0 mg, 316 µmol), Bis(tri-*tert*-butylphosphine)palladium(0) (5.37 mg, 10.5 µmol), dry tetrahydrofuran (20 mL), purification method B

Yield: 15.3 mg (30.1 µmol, 29 %).

^{1}**H NMR** (600 MHz, DMSO-*d*$_6$): δ = 8.73 – 8.60 (m, 8H), 8.58 (s, 2H), 8.28 (s, 2H), 7.68 – 7.56 (m, 8H), 3.37 – 3.21 (m, 4H)* ppm.

*The signal is partially superimposed by the signal of water (see Figure S37).

The ^{13}C NMR spectrum could not be analyzed due to low signal intensity as rapid precipitation of the compound occurred during the measurement in DMSO-*d*$_6$ at room temperature (see Figure S38).

HRMS (EI) *m/z*: $[M]^{\cdot+}$ Calcd for $^{12}C_{38}{}^{1}H_{24}{}^{14}N_2$ 508.1940; Found 508.1941.

IR (ATR): $\tilde{\nu}$ = 3032 (w), 2169 (w), 1644 (w), 1609 (m), 1578 (w), 1494 (m), 1432 (m), 905 (m), 877 (m), 851 (m), 810 (w) cm^{-1}.

General procedure for the synthesis of triphenylene diazocines 23 – 31 by one-pot deprotection and *in situ* reaction with electrophiles

Under nitrogen atmosphere tetrakis(*tert*-butyldimethylsilyloxy)triphenylene diazocine (**18**, 100 mg, 97.1 µmol, 1.00 eq.) was dissolved in dry tetrahydrofuran (20 mL) at room temperature. Tetrabutylammonium fluoride (1.0 M in THF, 152 mg, 583 µmol,

6.00 eq.) was added at room temperature and stirred for 30 s. Subsequently, the corresponding electrophile (583 µmol, 6.00 eq) was added and stirred either at 70 °C for 3 h (condition A) or at room temperature for 15 h (condition B).

Purification method C:

The solvent was removed in vacuo and the residue dissolved in a small amount of dichloromethane. The solution was carefully overlaid with a small amount of methanol and stored in the refrigerator over night. The precipitated solid was filtered off, washed with a small amount of ice-cold methanol and dried in vacuo. The product was obtained as a yellow amorphous solid.

Purification method D:

The precipitated solid was filtered off, washed with a small amount of ice-cold tetrahydrofuran and dried in vacuo. The product was obtained as a yellow amorphous solid.

Tetrakis(hexyloxy)triphenylene diazocine (23)

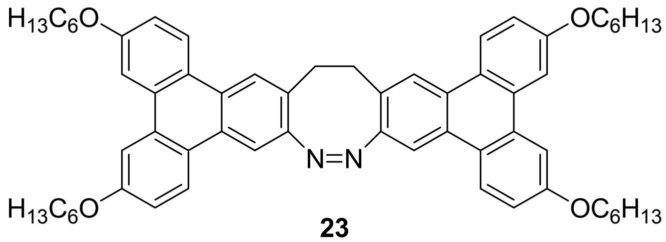

23

1-Iodohexane (124 mg, 583 µmol), condition A, purification method C.

Yield: 81.2 mg (89.3 µmol, 92 %).

^{1}H NMR (600 MHz, $CDCl_3$): δ = 8.34 (d, J = 5.4 Hz, 2H), 8.33 (d, J = 5.4 Hz, 2H), 8.09 (s, 2H), 7.96 (s, 2H), 7.79 – 7.78 (m, 4H), 7.16 (dd, J = 9.0 Hz, 2.4 Hz, 2H), 7.13 (dd, J = 9.1 Hz, 2.4 Hz, 2H), 4.10 – 4.07 (m, 8H), 3.31 – 3.14 (m_c, 4H), 1.86 – 1.80 (m, 8H), 1.52 – 1.47 (m, 8H), 1.39 – 1.32 (m, 16H), 0.92 – 0.89 (m, 12H) ppm.

^{13}C NMR (151 MHz, $CDCl_3$): δ = 158.6, 158.5, 154.4, 131.11, 131.09, 128.0, 127.9, 127.3, 125.1, 124.9, 123.9, 123.5, 123.3, 116.1, 115.9, 113.3, 107.2, 107.0, 68.40, 68.37, 32.4, 31.8, 29.5, 25.9, 22.8, 14.2 ppm.

The number of ^{13}C NMR signals does not equal the number of non-symmetry equivalent carbon atoms probably because of superposition of signals.

HRMS (ESI) m/z: $[M+H]^+$ Calcd for $C_{62}H_{73}O_4N_2$ 909.5565; Found 909.5547.

IR (ATR): $\tilde{\nu}$ = 2927 (m), 2855 (m), 2168 (w), 1737 (w), 1613 (s), 1575 (w), 1536 (w), 1511 (m), 1488 (w), 1462 (m), 1356 (m), 1147 (w), 1120 (m), 857 (m), 835 (m), 807 (s) cm^{-1}.

Tetrakis(dodecyloxy)triphenylene diazocine (24)

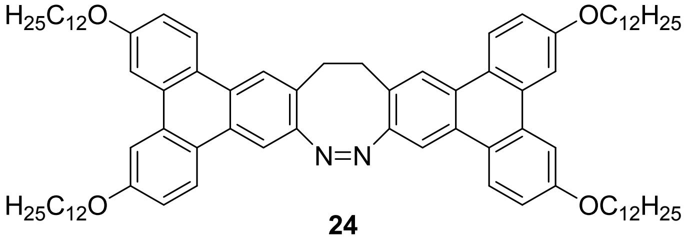

24

1-Iodododecane (173 mg, 583 µmol), condition A, purification method C.

Yield: 100 mg (80.3 µmol, 83 %).

^{1}H NMR (600 MHz, $CDCl_3$): δ = 8.37 (d, J = 8.1 Hz, 2H), 8.35 (d, J = 8.1 Hz, 2H), 8.11 (s, 2H), 7.97 (s, 2H), 7.81 – 7.80 (m, 4H), 7.17 (dd, J = 9.1 Hz, 2.4 Hz, 2H), 7.15 (dd, J = 9.1 Hz, 2.4 Hz, 2H), 4.11 – 4.09 (m, 8H), 3.33 – 3.15 (m_c, 4H), 1.86 – 1.81 (m, 8H), 1.52 – 1.47 (m, 8H), 1.39 – 1.34 (m, 8H), 1.33 – 1.23 (m, 56H), 0.88 – 0.85 (m, 12H) ppm.

^{13}C NMR (151 MHz, $CDCl_3$): δ = 158.6, 158.5, 154.4, 131.14, 131.12, 128.0, 127.9, 127.3, 125.1, 124.9, 123.9, 123.6, 123.3, 116.1, 116.0, 113.3, 107.2, 107.1, 68.43, 68.40, 32.4, 32.1, 29.81, 29.78, 29.75, 29.74, 29.6, 29.52, 29.49, 26.2, 22.8, 14.3 ppm.

The number of ^{13}C NMR signals does not equal the number of non-symmetry equivalent carbon atoms probably because of superposition of signals.

HRMS (ESI) *m/z*: $[M+H]^+$ Calcd for $C_{86}H_{121}O_4N_2$ 1245.9321; Found 1245.9294.

IR (ATR): $\tilde{\nu}$ = 2920 (s), 2851 (s), 2171 (w), 1745 (w), 1613 (s), 1575 (w), 1535 (w), 1511 (m), 1488 (w), 1463 (m), 1429 (m), 1357 (m), 1120 (w), 911 (w), 856 (m), 834 (m), 807 (s) cm^{-1}.

Tetrakis(octadecyloxy)triphenylene diazocine (25)

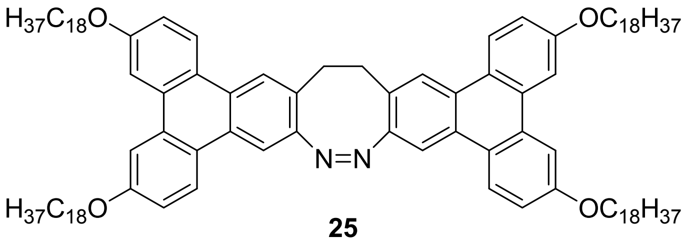

25

1-Iodooctadecane (222 mg, 583 µmol), condition A, purification method C.

Yield: 125 mg (787 µmol, 81 %).

^{1}H NMR (600 MHz, $CDCl_3$): δ = 8.36 (d, J = 7.5 Hz, 2H), 8.35 (d, J = 7.4 Hz, 2H), 8.11 (s, 2H), 7.97 (s, 2H), 7.80 – 7.79 (m, 4H), 7.17 (dd, J = 9.1 Hz, 2.3 Hz, 2H), 7.15 (dd, J = 9.2 Hz, 2.3 Hz, 2H), 4.11 – 4.08 (m, 8H), 3.32 – 3.15 (m_c, 4H), 1.86 – 1.81 (m, 8H), 1.52 – 1.47 (m, 8H), 1.39 – 1.34 (m, 8H), 1.33 – 1.24 (m, 112H), 0.88 – 0.86 (m, 12H) ppm.

^{13}C NMR (151 MHz, $CDCl_3$): δ = 158.6, 158.5, 154.4, 131.14, 131.11, 128.0, 127.9, 127.3, 125.1, 124.9, 123.9, 123.6, 123.3, 116.1, 116.0, 113.3, 107.2, 107.0, 68.42, 68.39, 32.4, 32.1, 29.9, 29.77, 29.75, 29.6, 29.5, 26.3, 22.8, 14.3 ppm.

The number of ^{13}C NMR signals does not equal the number of non-symmetry equivalent carbon atoms probably because of superposition of signals.

LRMS (MALDI) *m/z*: $[M+H]^+$ Calcd for $C_{110}H_{169}N_2O_4$ 1582.3; Found 1582.3.

IR (ATR): $\tilde{\nu}$ = 3031 (w), 2916 (s), 2849 (s), 1738 (w), 1613 (s), 1575 (m), 1536 (w), 1510 (m), 1489 (m), 1463 (m), 1427 (m), 1363 (m), 1117 (w), 1072 (w), 881 (w), 834 (m), 813 (m) cm^{-1}.

EA (for $C_{110}H_{168}N_2O_4$): Calcd for C, 83.49; H, 10.70; N, 1.77. Found C, 82.22; H, 10.44; N, 1.80.

Tetrakis(pent-4-en-1-yloxy)triphenylene diazocine (26)

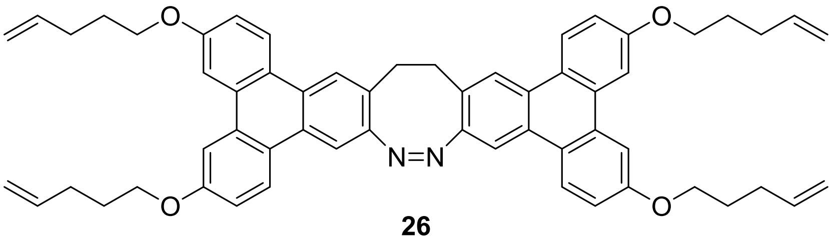

26

5-Bromo-1-pentene (86.9 mg, 583 µmol), condition A, purification method C.

Yield: 61.5 mg (72.8 µmol, 75 %).

^{1}H NMR (600 MHz, $CDCl_3$): δ = 8.34 (d, *J* = 5.5 Hz, 2H), 8.33 (d, *J* = 5.3 Hz, 2H), 8.09 (s, 2H), 7.96 (s, 2H), 7.78 – 7.77 (m, 4H), 7.16 (dd, *J* = 9.1 Hz, 2.5 Hz, 2H), 7.13 (dd, *J* = 9.0 Hz, 2.5 Hz, 2H), 5.90 – 5.84 (m_c, 4H), 5.10 – 5.06 (m, 4H), 5.02 – 5.00 (m, 4H), 4.12 – 4.09 (m_c, 8H), 3.31 – 3.14 (m_c, 4H), 2.30 – 2.26 (m, 8H), 1.96 – 1.91 (m, 8H) ppm.

^{13}C NMR (151 MHz, $CDCl_3$): δ = 158.5, 158.4, 154.4, 137.9, 131.08, 131.05, 128.0, 127.9, 127.3, 125.1, 124.9, 123.9, 123.6, 123.4, 116.0, 116.0, 115.5, 113.3, 107.2, 107.0, 67.53, 67.50, 32.4, 30.3, 28.6 ppm.

The number of ^{13}C NMR signals does not equal the number of non-symmetry equivalent carbon atoms probably because of superposition of signals.

HRMS (EI) *m/z*: $[M]^{\cdot+}$ Calcd for $^{12}C_{58}{}^{1}H_{56}{}^{14}N_2{}^{16}O_4$ 844.4240; Found 844.4231.

IR (ATR): $\tilde{\nu}$ = 3070 (w), 2935 (w), 2871 (w), 1640 (w), 1612 (s), 1577 (w), 1509 (m), 1489 (w), 1460 (m), 1426 (m), 1356 (m), 1117 (m), 994 (m), 910 (s), 853 (m), 833 (m), 821 (m), 809 (s) cm^{-1}.

Tetrakis(benzyloxy)triphenylene diazocine (27)

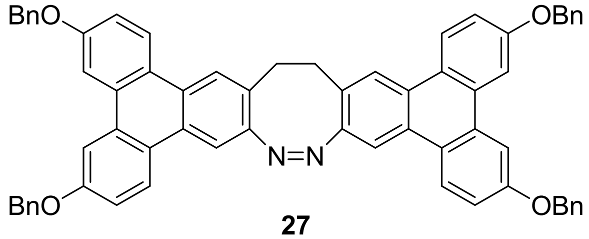

27

Benzyl bromide (99.7 mg, 583 µmol), condition A, purification method D.

Yield: 60.3 mg (64.6 µmol, 67 %).

^{1}H NMR (600 MHz, DMSO-*d*$_6$): δ = 8.59 (d, *J* = 9.3 Hz, 2H), 8.56 (d, *J* = 9.2 Hz, 2H), 8.43 (s, 2H), 8.15 – 8.14 (m, 4H), 8.12 (s, 2H), 7.55 – 7.53 (m, 8H), 7.42 – 7.39 (m, 8H), 7.35 – 7.31 (m, 8H), 5.33 (s, 4H), 5.32 (s, 4H), 3.31 – 3.15 (m, 4H)* ppm.

*The signal is partially superimposed by the signal of water (see Figure S47).

^{13}C NMR (151 MHz, DMSO-*d*$_6$): δ = 158.0, 157.9, 154.3, 137.0, 130.6, 130.4, 128.5, 128.0, 128.0, 127.9, 127.7, 127.2, 127.1, 125.44, 125.36, 124.3, 122.9, 122.7, 116.89, 116.85, 112.6, 107.5, 107.3, 69.6, 31.2.

The number of ^{13}C NMR signals does not equal the number of non-symmetry equivalent carbon atoms probably because of superposition of signals.

HRMS (ESI) *m/z*: [M+H]$^+$ Calcd for $C_{66}H_{49}O_4N_2$ 933.3642; Found 933.3636.

IR (ATR): $\tilde{\nu}$ = 3030 (w), 2887 (w), 2202 (w), 1613 (s), 1574 (m), 1537 (w), 1511 (m), 1489 (m), 1454 (m), 1121 (m), 1081 (w), 915 (w), 856 (m), 834 (m), 802 (s), 755 (m), 734 (s), 694 (s) cm^{-1}.

Tetrakis(acetoxy)triphenylene diazocine (28)

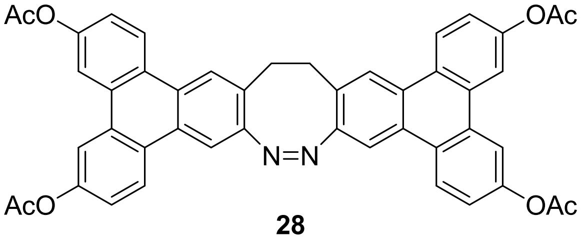

28

Acetyl chloride (45.8 mg, 583 µmol), condition B, purification method D. The crude product was additionally purified by flash column chromatography on silica gel (cyclohexane/ethyl acetate 9:1 → 1:1).

Yield: 33.1 mg (44.7 µmol, 46 %).

^{1}H NMR (600 MHz, $CDCl_3$): δ = 8.44 (d, *J* = 9.1 Hz, 2H), 8.42 (d, *J* = 9.1 Hz, 2H), 8.15 (s, 2H), 8.051 (s, 2H), 8.047 (s, 2H), 7.99 (s, 2H), 7.34 (dd, *J* = 8.0 Hz, 2.3 Hz, 2H), 7.32 (dd, *J* = 8.0 Hz, 2.3 Hz, 2H), 3.36 – 3.17 (m_c, 4H), 2.340 (s, 6H), 2.336 (s, 6H) ppm.

^{13}C NMR (151 MHz, $CDCl_3$): δ = 169.52 155.1, 150.2, 150.1, 130.59, 130.57, 128.5, 128.3, 127.3, 127.1, 125.1, 124.8, 124.7, 121.8, 121.7, 115.84, 115.80, 113.7, 32.3, 21.4 ppm.

The number of ^{13}C NMR signals does not equal the number of non-symmetry equivalent carbon atoms probably because of superposition of signals.

HRMS (ESI) *m/z*: [M+NH$_4$]$^+$ Calcd for $C_{46}H_{36}O_8N_3$ 758.2497; Found 758.2485.

IR (ATR): $\tilde{\nu}$ = 2918 (s), 2850 (s), 2171 (w), 1740 (s), 1615 (w), 1575 (w), 1507 (w), 1464 (m), 1428 (m), 1368 (m), 1179 (s), 1119 (m), 1016 (m), 891 (m), 812 (m) cm^{-1}.

Tetrakis(acrylate)triphenylene diazocine (29)

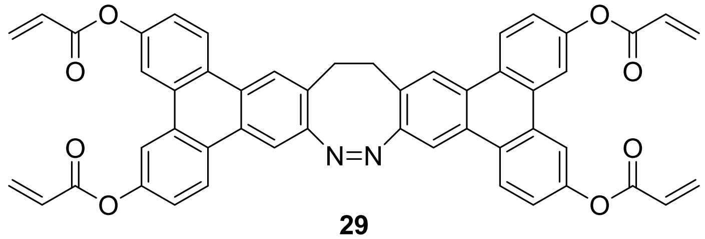

29

Acryloyl chloride (52.8 mg, 583 µmol), condition B, purification method D.

Yield: 39.8 mg (50.5 µmol, 52 %).

^{1}H-NMR (500 MHz, $CDCl_3$): δ = 8.47 (d, J = 9.1 Hz, 2H), 8.44 (d, J = 9.1 Hz, 2H), 8.18 (s, 2H), 8.12 (s, 2H), 8.11 (s, 2H), 8.02 (s, 2H), 7.39 (dd, J = 6.0 Hz, 2.4 Hz, 2H), 7.37 (dd, J = 6.0 Hz, 2.4 Hz, 2H), 6.66 – 6.62 (m, 4H), 6.38 – 6.32 (m, 4H), 6.06 – 6.03 (m, 4H), 3.37 – 3.20 (m, 4H) ppm.

^{13}C NMR (126 MHz, $CDCl_3$): δ = 164.6, 155.2, 150.14, 150.07, 133.0, 130.6, 128.5, 128.3, 128.0, 127.4, 127.2, 125.1, 124.83, 124.76, 121.8, 121.7, 115.84, 115.80, 113.7, 32.3 ppm.

The number of ^{13}C NMR signals does not equal the number of non-symmetry equivalent carbon atoms probably because of superposition of signals.

HRMS (ESI) *m/z*: $[M+NH_4]^+$ Calcd for $C_{50}H_{36}O_8N_3$ 806.2497; Found 806.2479.

IR (ATR): $\tilde{\nu}$ = 3069 (w), 2919 (w), 1735 (s), 1612 (w), 1578 (w), 1508 (w), 1490 (w), 1459 (w), 1426 (w), 1400 (m), 1293 (w), 1243 (m), 1139 (s), 1070 (m), 1019 (m), 977 (m), 907 (m), 866 (m), 798 (s) cm^{-1}.

Tetrakis(methyl-2-phenoxyacetate)triphenylene diazocine (30)

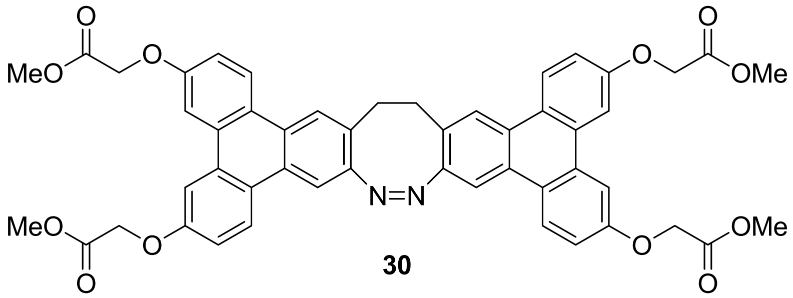

30

Methyl bromoacetate (89.1 mg, 583 µmol), condition B, purification method D.

Yield: 62.7 mg (72.8 µmol, 75 %).

^{1}H NMR (600 MHz, DMSO-d_6): δ = 8.57 (d, J = 9.3 Hz, 2H), 8.54 (d, J = 9.3 Hz, 2H), 8.41 (s, 2H), 8.12 (s, 2H), 8.06 (d, J = 2.6 Hz, 2H), 8.05 (d, J = 2.7 Hz, 2H), 7.26 (t, J = 2.6 Hz, 2H), 7.25 (t, J = 2.6 Hz, 2H), 5.033 (s, 4H), 5.031 (s, 4H), 3.70 (s, 12H), 3.30 – 3.14 (m, 4H)* ppm.

*The signal is partially superimposed by the signal of water (see Figure S53).

^{13}C NMR (151 MHz, DMSO-d_6): δ = 169.2, 157.2, 157.1, 154.4, 130.5, 130.3, 127.8, 127.1, 127.0, 125.5, 125.4, 124.3, 123.2, 123.1, 116.5, 116.4, 112.7, 107.6, 107.4, 64.8, 51.8, 31.2 ppm.

The number of ^{13}C NMR signals does not equal the number of non-symmetry equivalent carbon atoms probably because of superposition of signals.

HRMS (ESI) *m*/*z*: $[M+NH_4]^+$ Calcd for $C_{50}H_{44}O_{12}N_3$ 878.2920; Found 878.2937.

IR (ATR): $\tilde{\nu}$ = 3031 (w), 2325 (w), 1747 (w), 1613 (s), 1575 (w), 1510 (m), 1489 (m), 1453 (m), 1428 (m), 1401 (w), 1360 (m), 1272 (w), 1203 (s), 1120 (m), 1081 (m), 1043 (m), 1017 (m), 880 (w), 834 (s), 809 (s) cm^{-1}.

Tetrakis(3,4,5-tris(dodecyloxy)benzyl-2-phenoxyacetate)triphenylene diazocine (31)

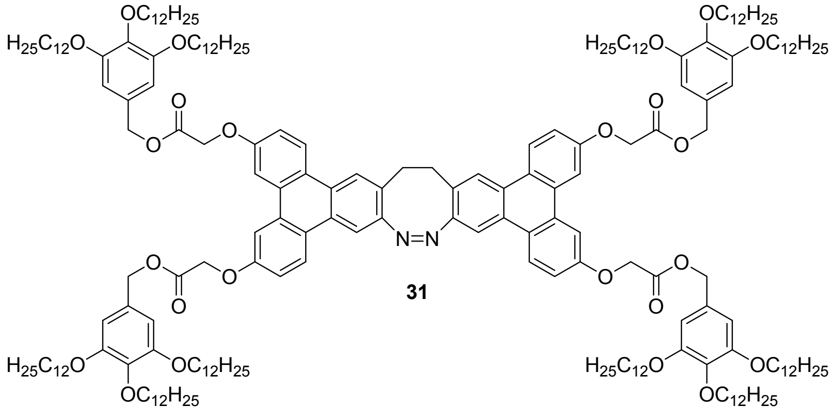

31

3,4,5-Tris(dodecyloxy)benzyl-2-bromoacetate (**32**, 456 mg, 583 µmol), condition B, purification method C.

Yield: 257 mg (76.1 µmol, 78 %).

^{1}H-NMR (500 MHz, $CDCl_3$): δ = 8.36 (d, *J* = 9.3 Hz, 4H), 8.11 (s, 2H), 7.97 (s, 2H), 7.80 – 7.79 (m, 4H), 7.17 (dd, *J* = 9.1 Hz, 2.5 Hz, 2H), 7.13 (dd, *J* = 9.1 Hz, 2.6 Hz, 2H), 6.502 (s, 4H), 6.500 (s, 4H), 5.13 (s, 8H), 4.77 (s, 4H), 4.76 (s, 4H), 3.89 (t, *J* = 6.5 Hz, 8H), 3.85 – 3.82 (m, 16H), 3.34 – 3.17 (m_c, 4H), 1.74 – 1.65 (m, 24H), 1.48 – 1.42 (m, 8H), 1.38 – 1.33 (m, 16H), 1.30 – 1.23 (m, 192H), 0.89 – 0.86 (m, 36H) ppm.

^{13}C NMR (126 MHz, $CDCl_3$): δ = 168.8, 157.24, 157.17, 154.6, 153.4, 138.5, 130.92, 130.89, 130.1, 130.0, 127.94, 127.88, 127.7, 125.4, 125.2, 124.5, 124.3, 124.1, 115.8, 113.5, 107.9, 107.8, 107.3, 73.5, 69.2, 67.7, 65.7, 32.1, 30.5, 29.92, 29.86, 29.8, 29.6, 29.5, 26.3, 26.2, 22.9, 14.3 ppm.

The number of ^{13}C NMR signals does not equal the number of non-symmetry equivalent carbon atoms probably because of superposition of signals.

LRMS (MALDI) *m*/*z*: $[M+H]^+$ Calcd for $C_{218}H_{345}N_2O_{24}$ 3375.6; Found 3375.3.

IR (ATR): $\tilde{\nu}$ = 2918 (s), 2850 (s), 1738 (m), 1614 (w), 1592 (w), 1508 (m), 1467 (m), 1439 (m), 1379 (w), 1336 (m), 1233 (m), 1210 (m), 1119 (s), 1075 (m), 878 (w), 832 (m), 808 (m) cm^{-1}.

EA (for $C_{218}H_{344}N_2O_{24}$): Calcd for C, 77.53; H, 10.27; N, 0.83. Found C, 77.49; H, 10.11; N, 0.86.

S 3 NMR spectra

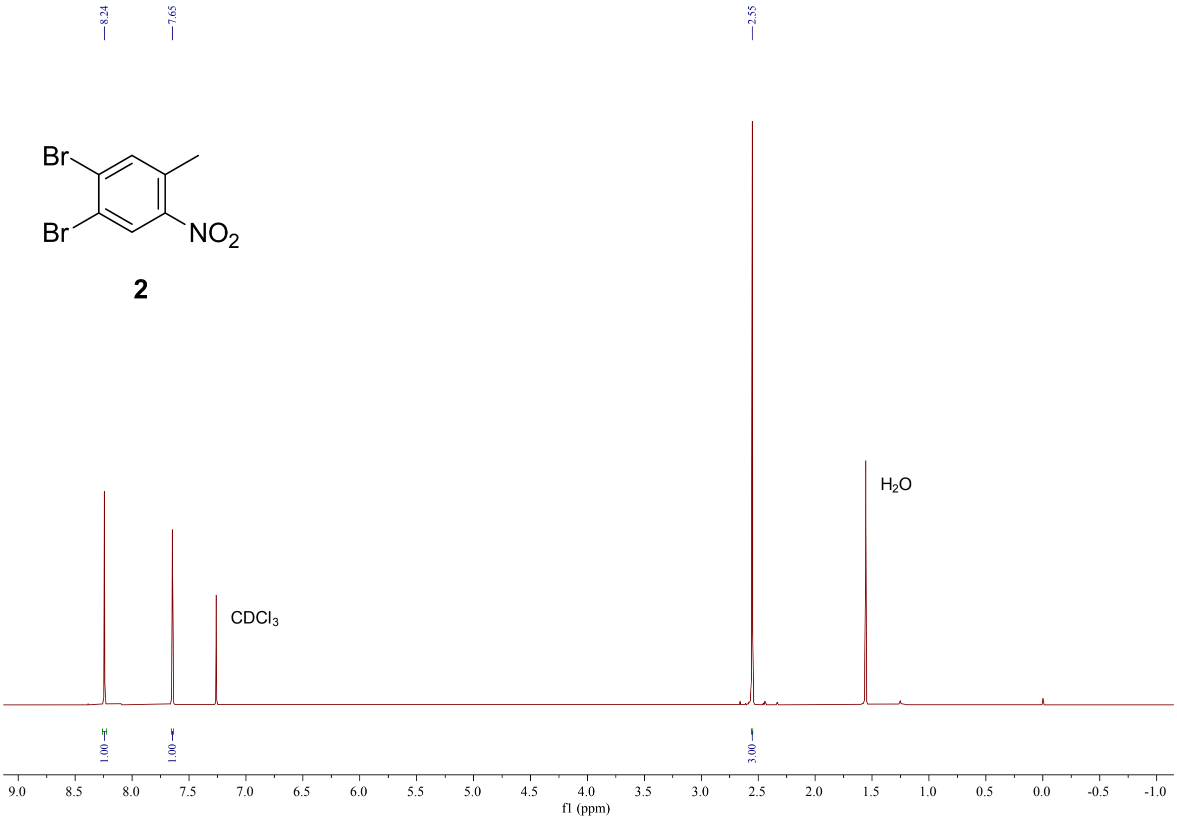

Figure S1: ^{1}H NMR spectrum (600 MHz, $CDCl_3$, 298 K) of compound **2**.

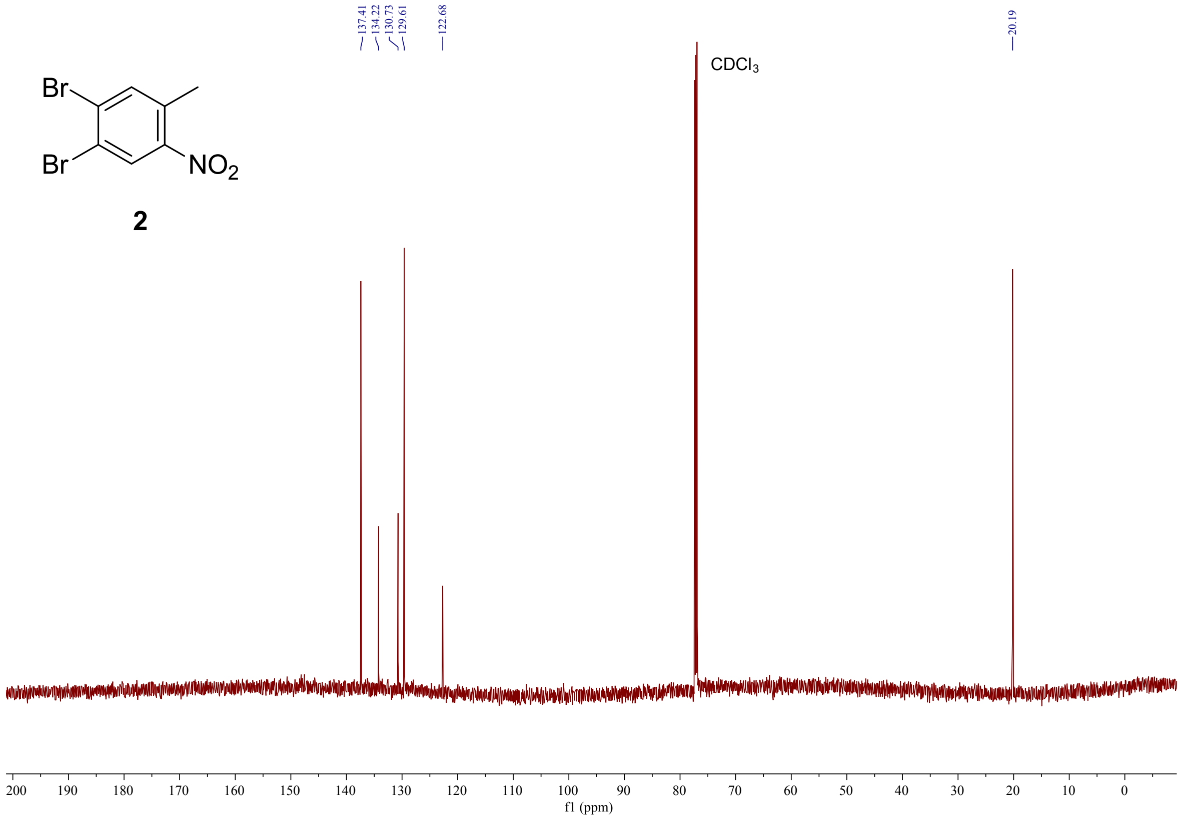

Figure S2: ^{13}C NMR spectrum (151 MHz, $CDCl_3$, 298 K) of compound **2**.

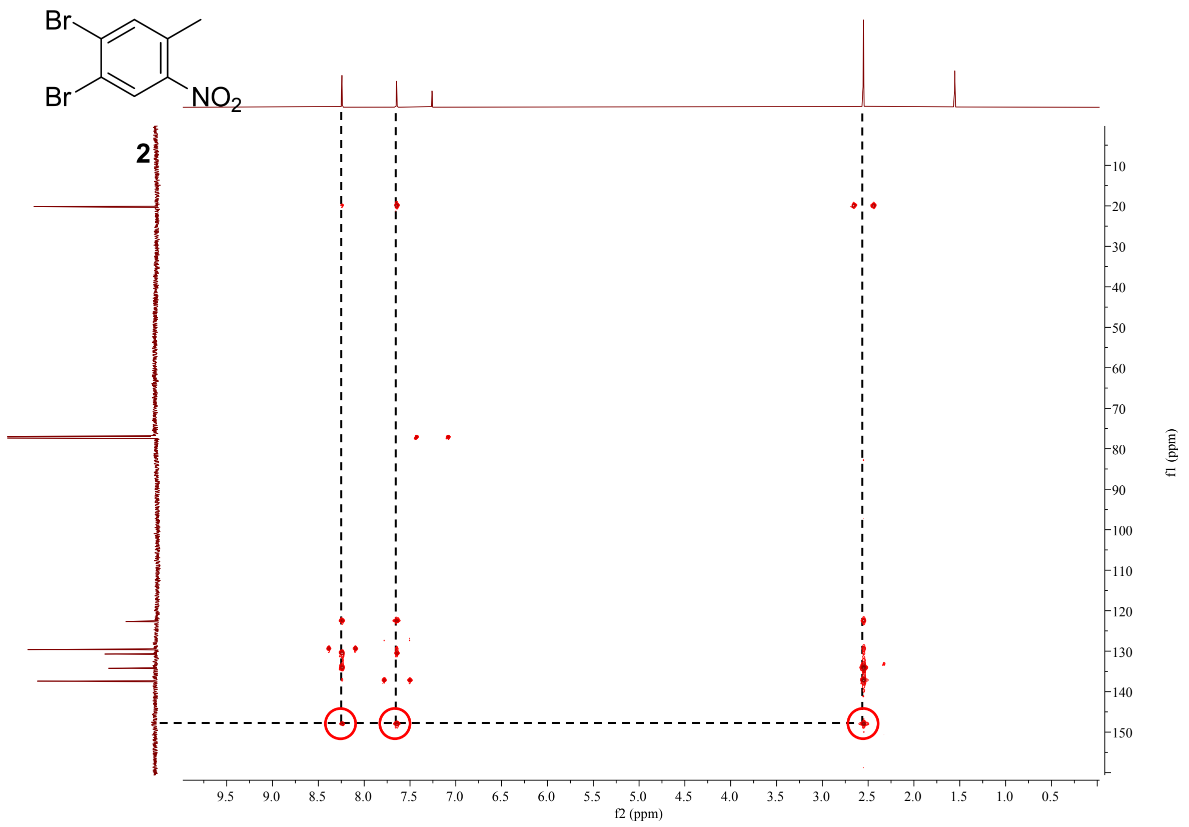

Figure S3: ^{1}H-^{13}C HMBC spectrum (600 MHz, 151 MHz, $CDCl_3$, 298 K) of compound **2**.

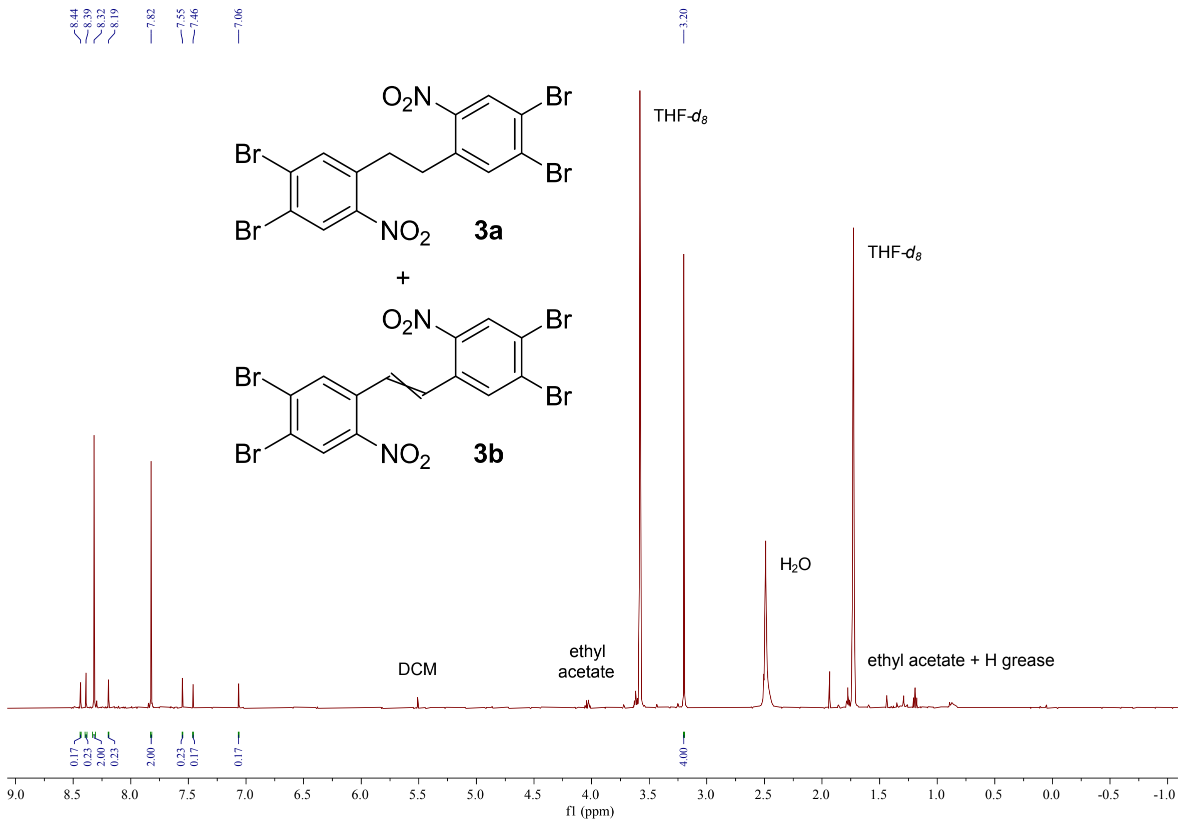

Figure S4: ^{1}H NMR spectrum (500 MHz, THF-d_8, 298 K) of compound **3a** and **3b**.

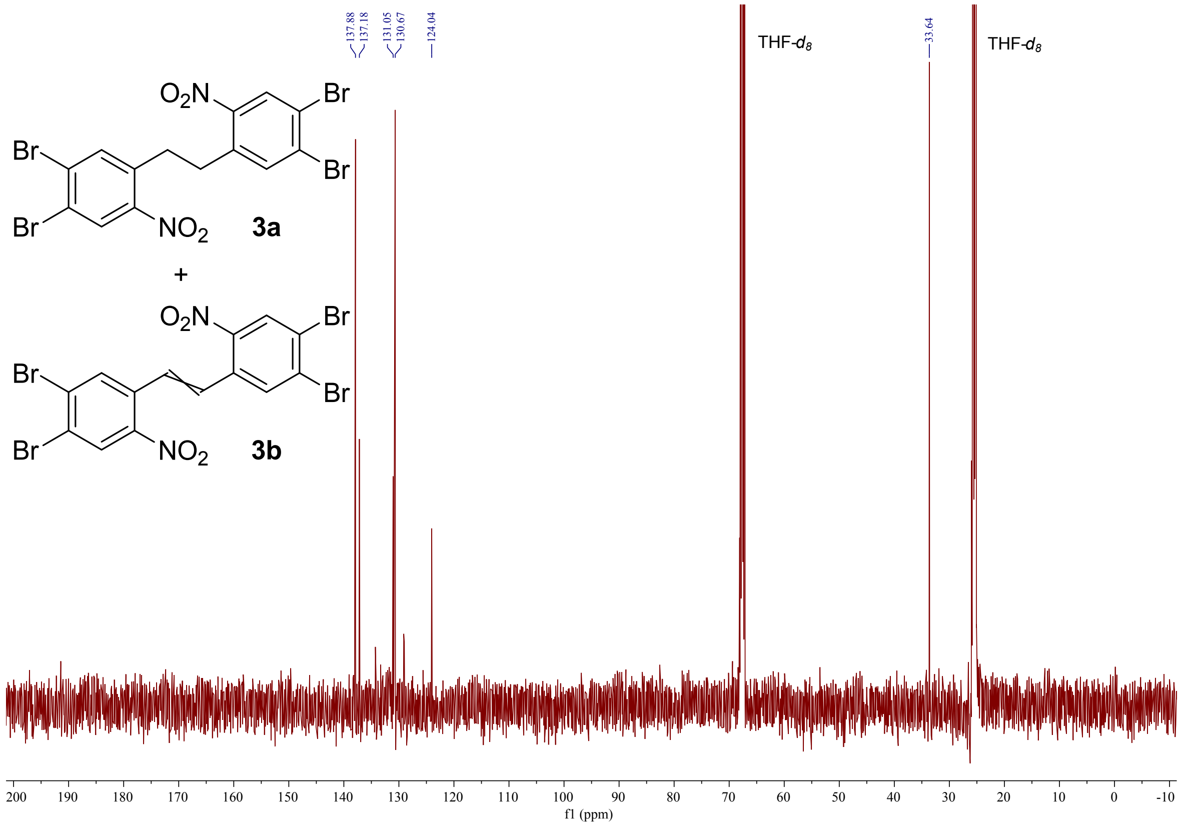

Figure S5: ^{13}C NMR spectrum (126 MHz, THF-d_8, 298 K) of compound **3a** and **3b**.

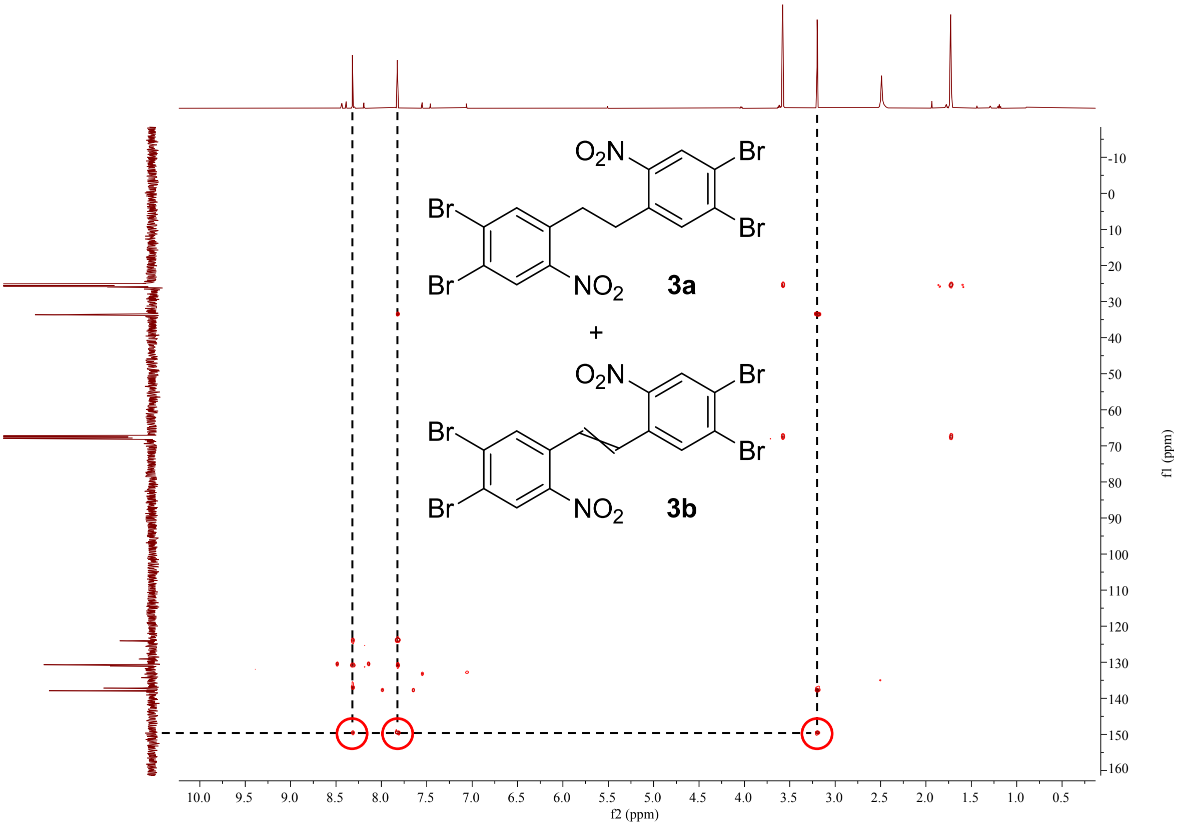

Figure S6: ^{1}H-^{13}C HMBC spectrum (500 MHz, 126 MHz, THF-d_8, 298 K) of compound **3a** and **3b**.

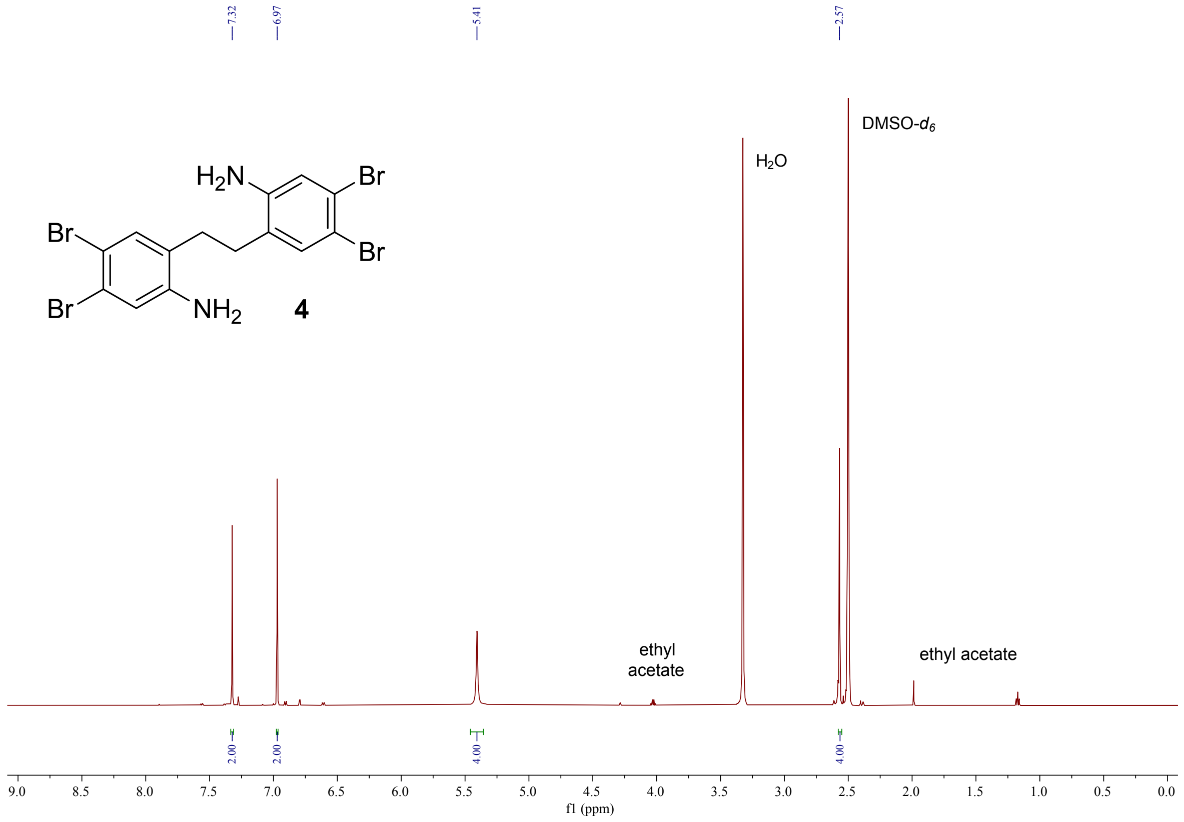

Figure S7: ^1H NMR spectrum (600 MHz, DMSO-d_8, 298 K) of compound **4**.

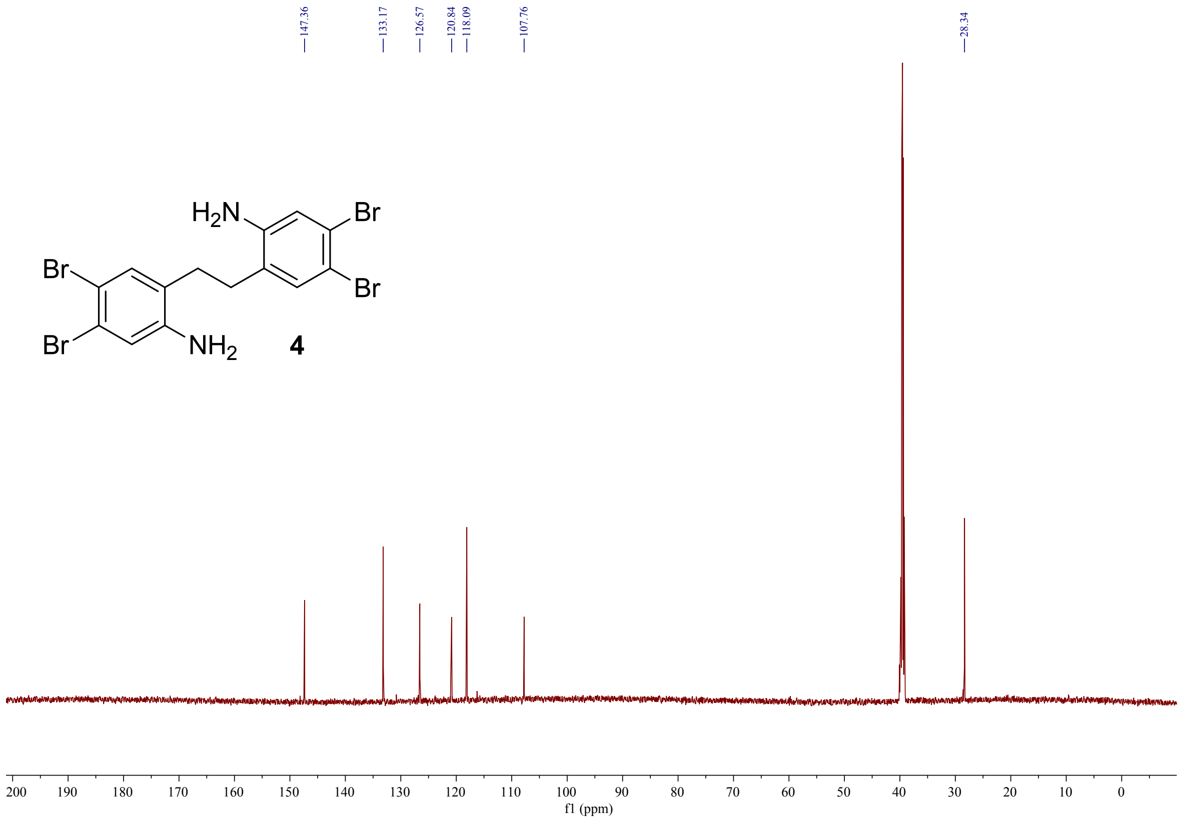

Figure S8: ^{13}C NMR spectrum (151 MHz, DMSO-d_8, 298 K) of compound **4**.

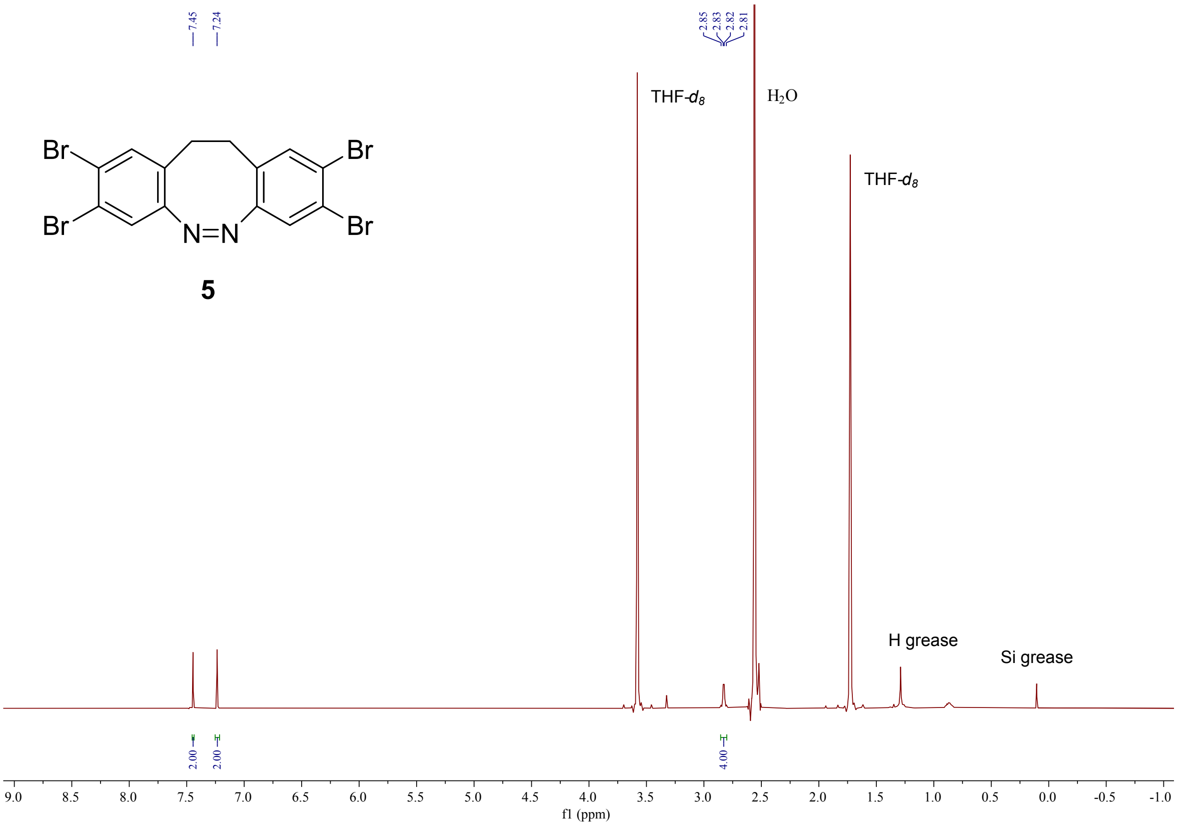

Figure S9: ^{1}H NMR spectrum (600 MHz, THF-d_8, 298 K) of compound **5**.

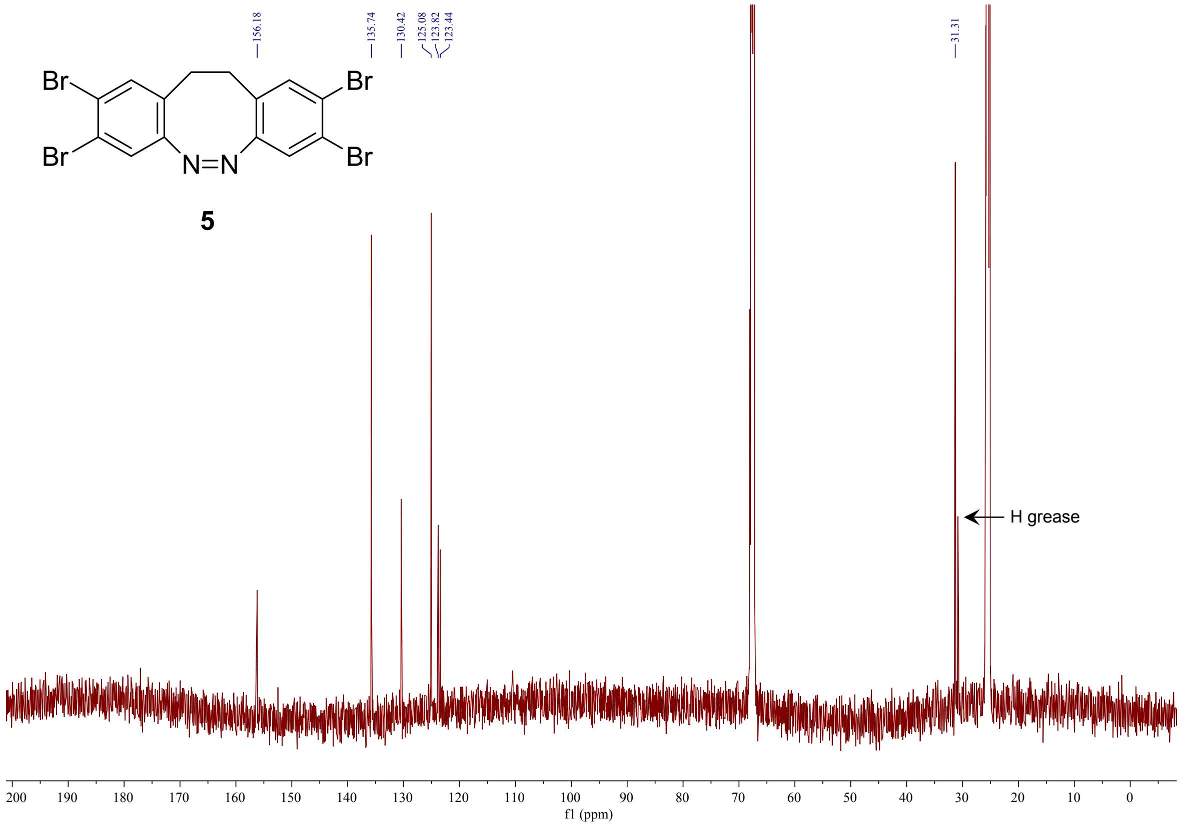

Figure S10: ^{13}C NMR spectrum (151 MHz, THF-d_8, 298 K) of compound **5**.

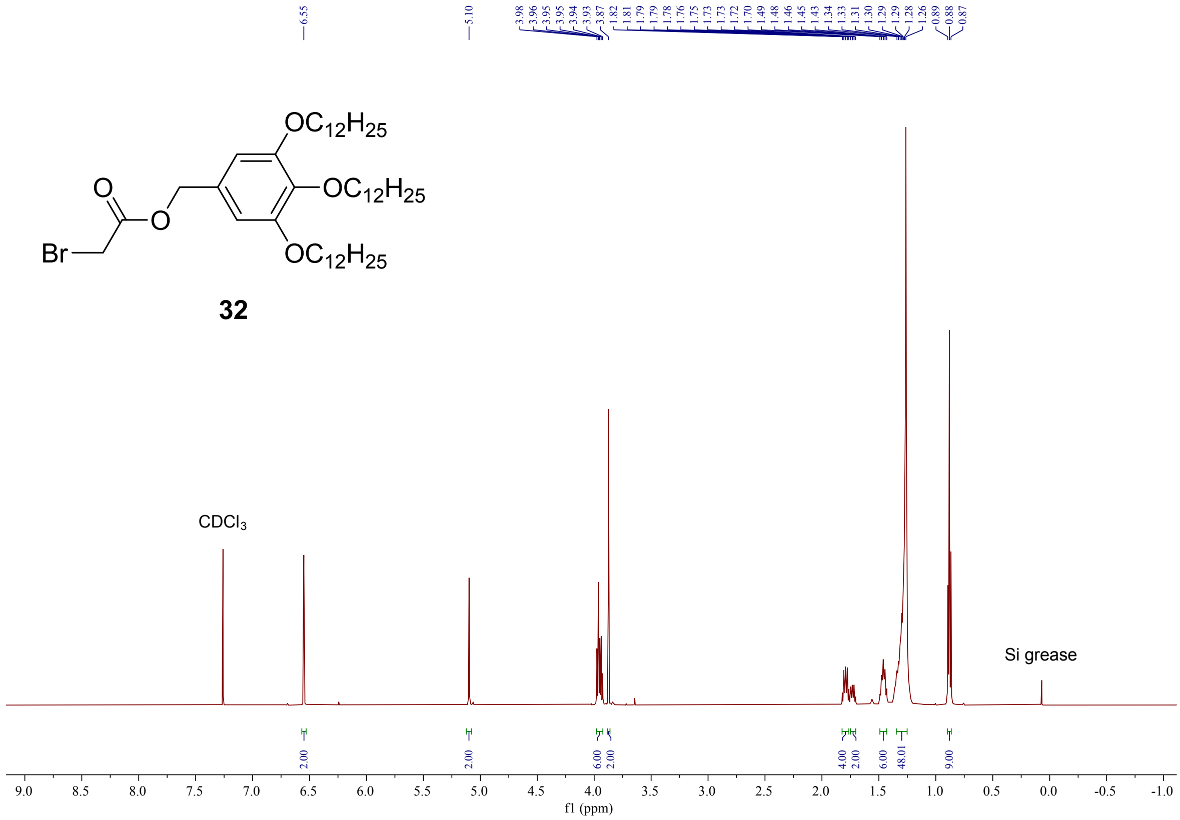

Figure S11: ^{1}H NMR spectrum (500 MHz, $CDCl_3$, 298 K) of compound **32**.

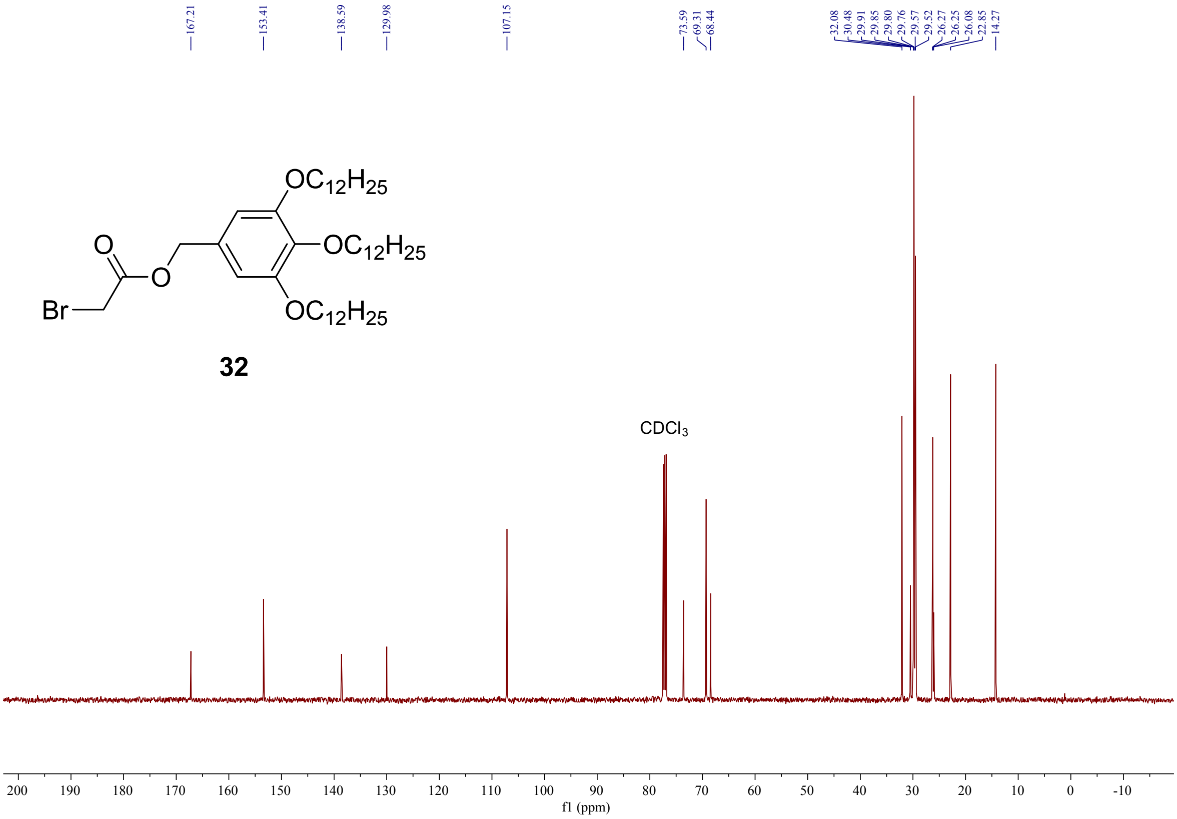

Figure S12: ^{13}C NMR spectrum (126 MHz, $CDCl_3$, 298 K) of compound **32**.

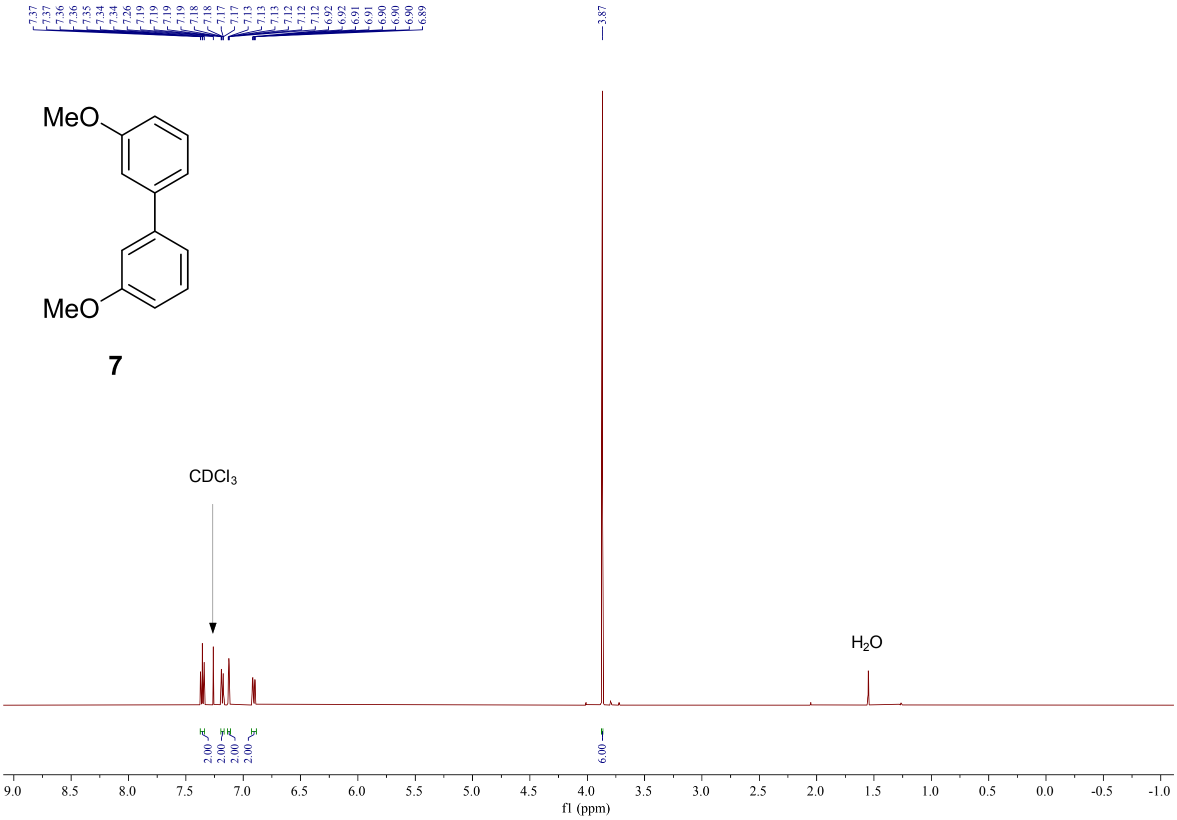

Figure S13: ^{1}H NMR spectrum (500 MHz, $CDCl_3$, 298 K) of compound **7**.

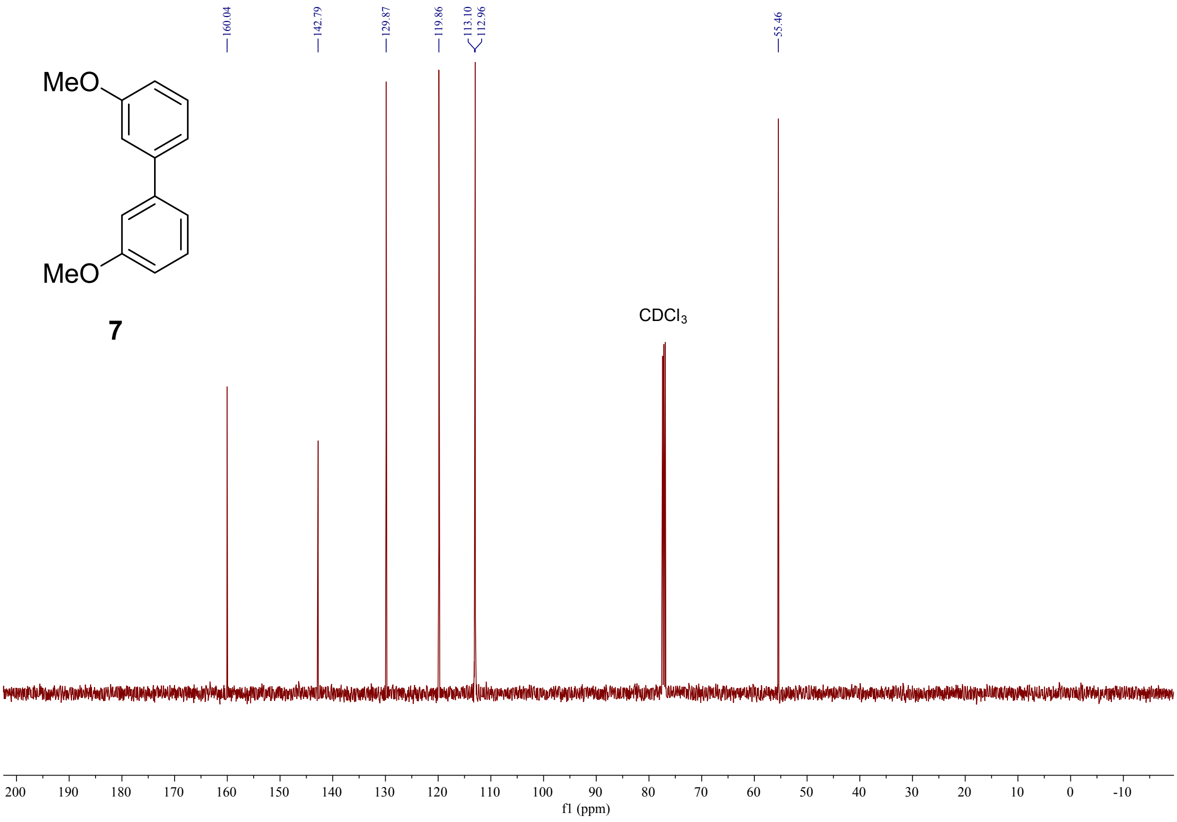

Figure S14: ^{13}C NMMR spectrum (126 MHz, $CDCl_3$, 298 K) of compound **7**.

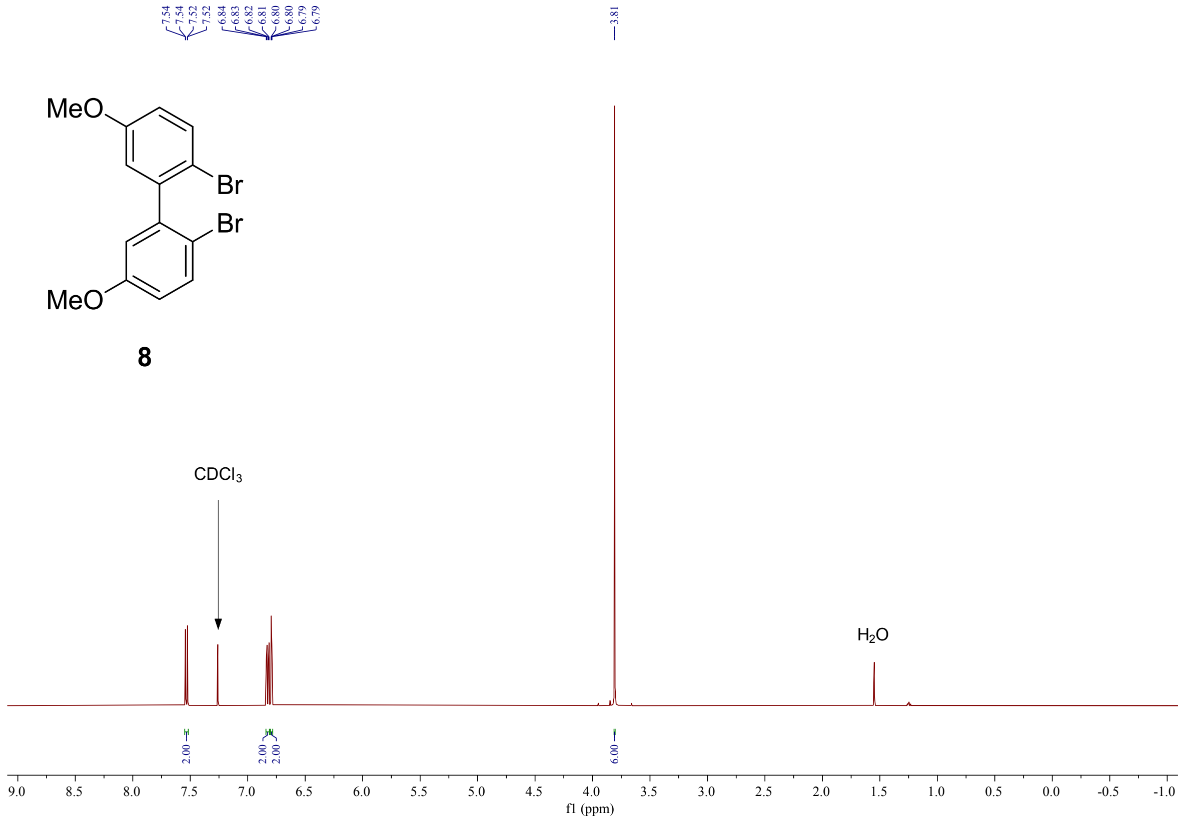

Figure S15: ^{1}H NMR spectrum (500 MHz, $CDCl_3$, 298 K) of compound **8**.

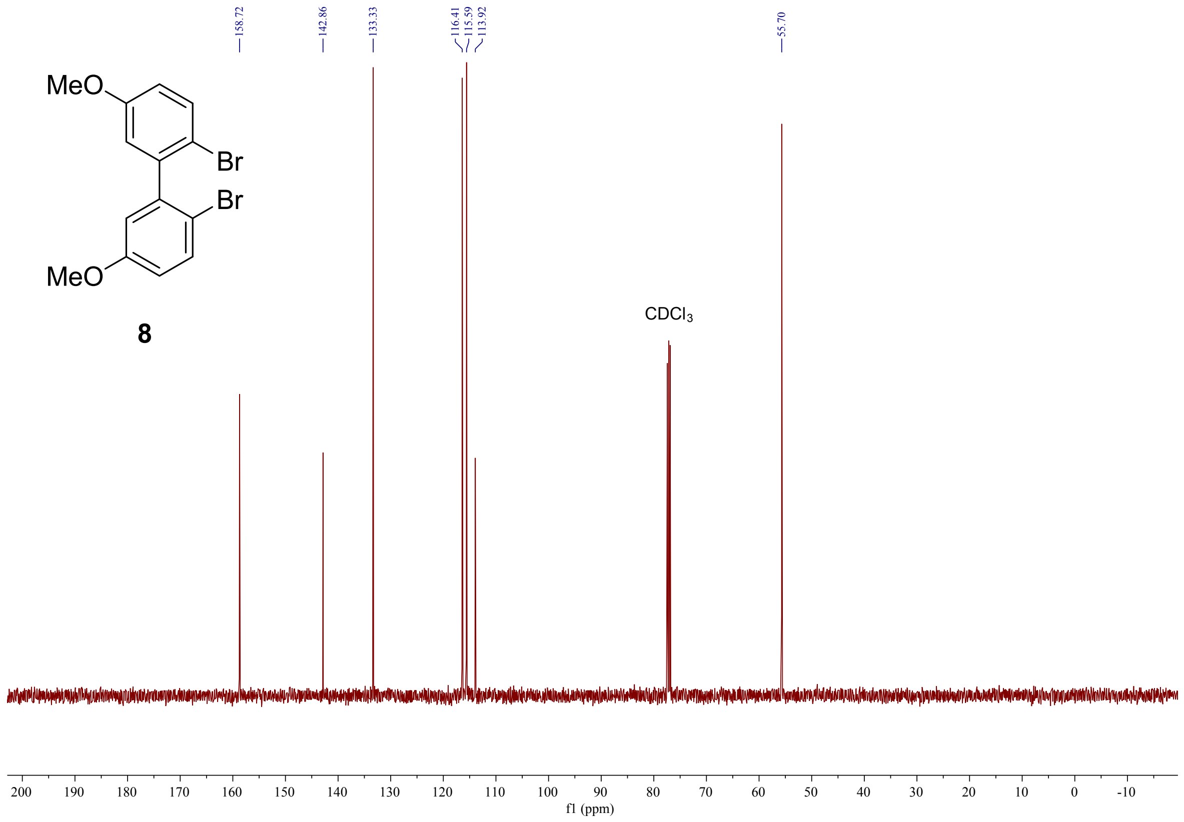

Figure S16: ^{13}C NMR spectrum (126 MHz, $CDCl_3$, 298 K) of compound **8**.

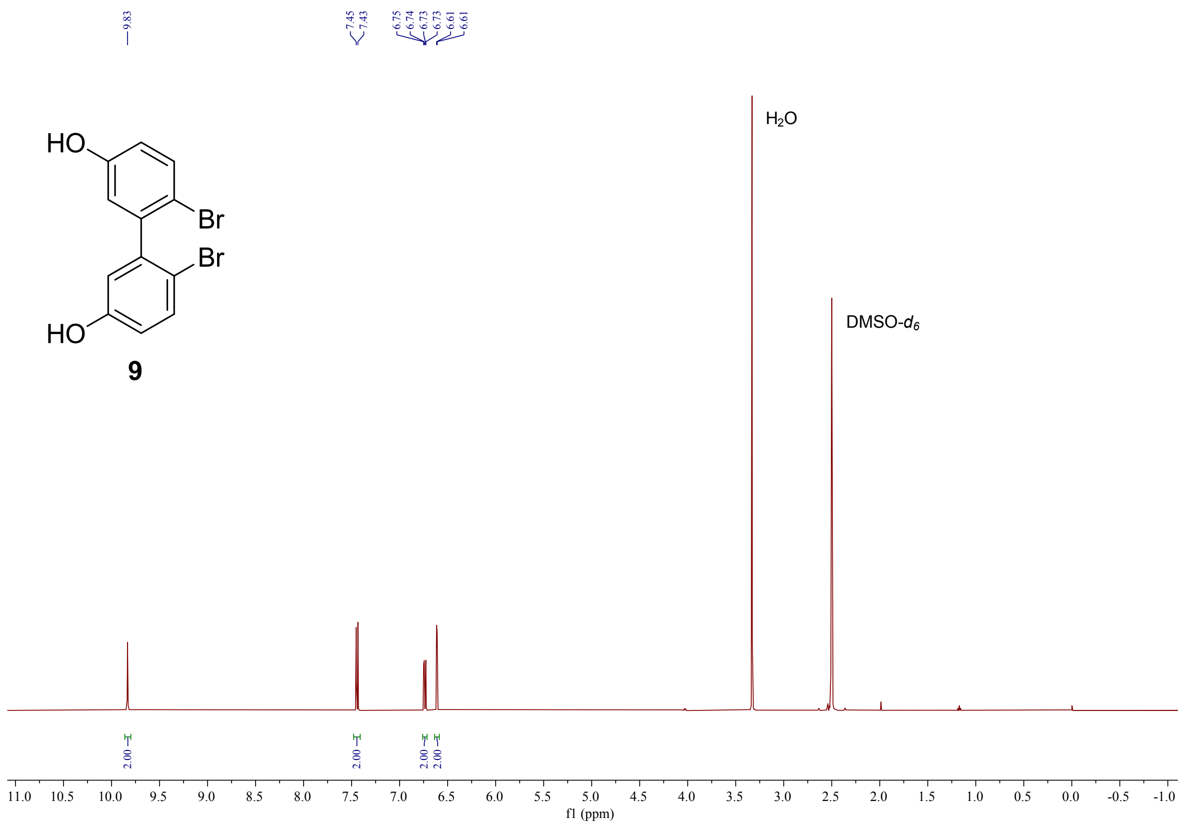

Figure S17: ^{1}H NMR spectrum (500 MHz, DMSO-d_6, 298 K) of compound **9**.

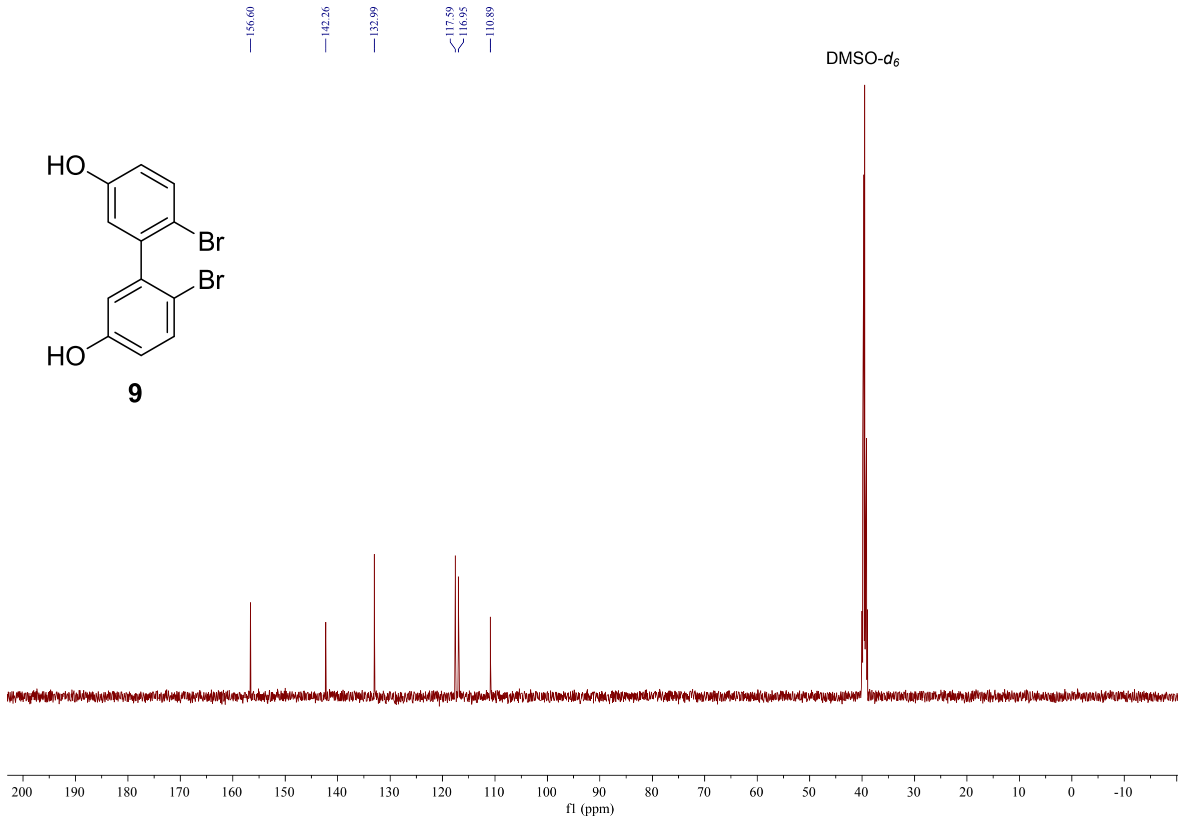

Figure S18: ^{13}C NMR spectrum (126 MHz, DMSO-d_6, 298 K) of compound **9**.

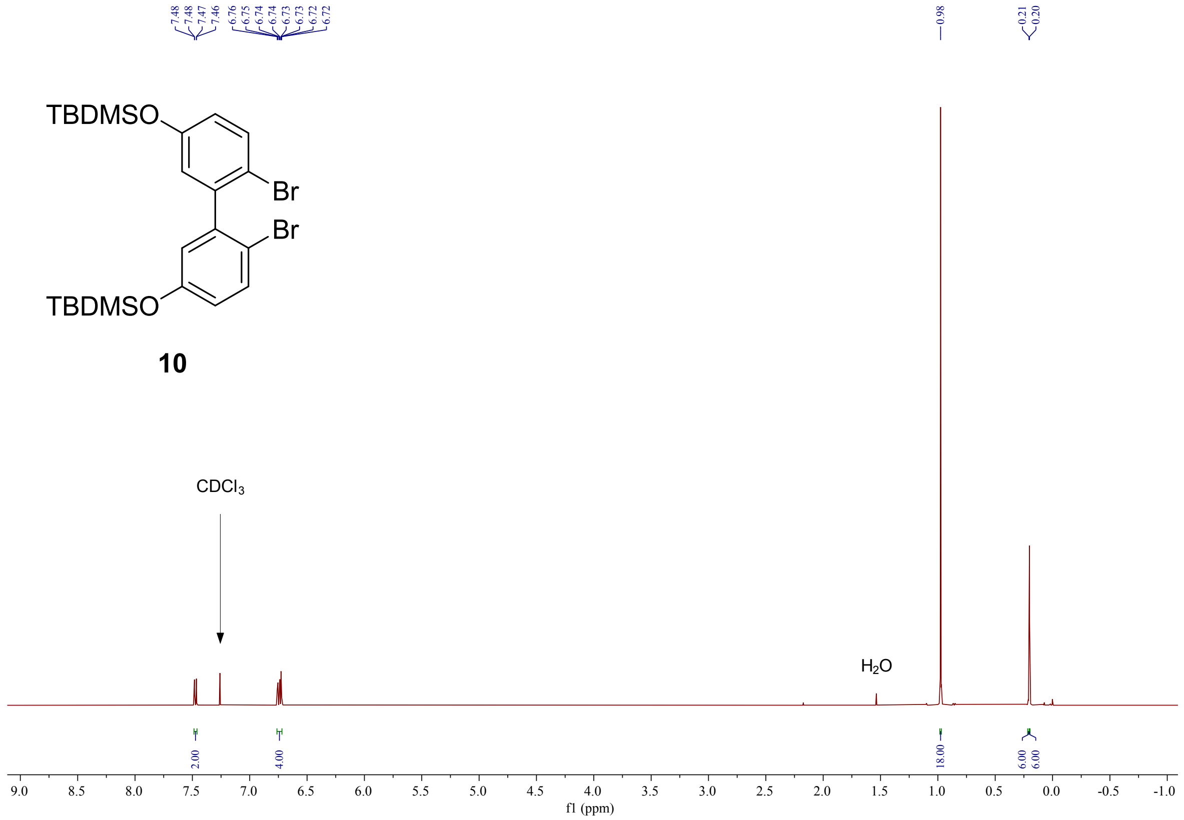

Figure S19: ^{1}H NMR spectrum (500 MHz, $CDCl_3$, 298 K) of compound **10**.

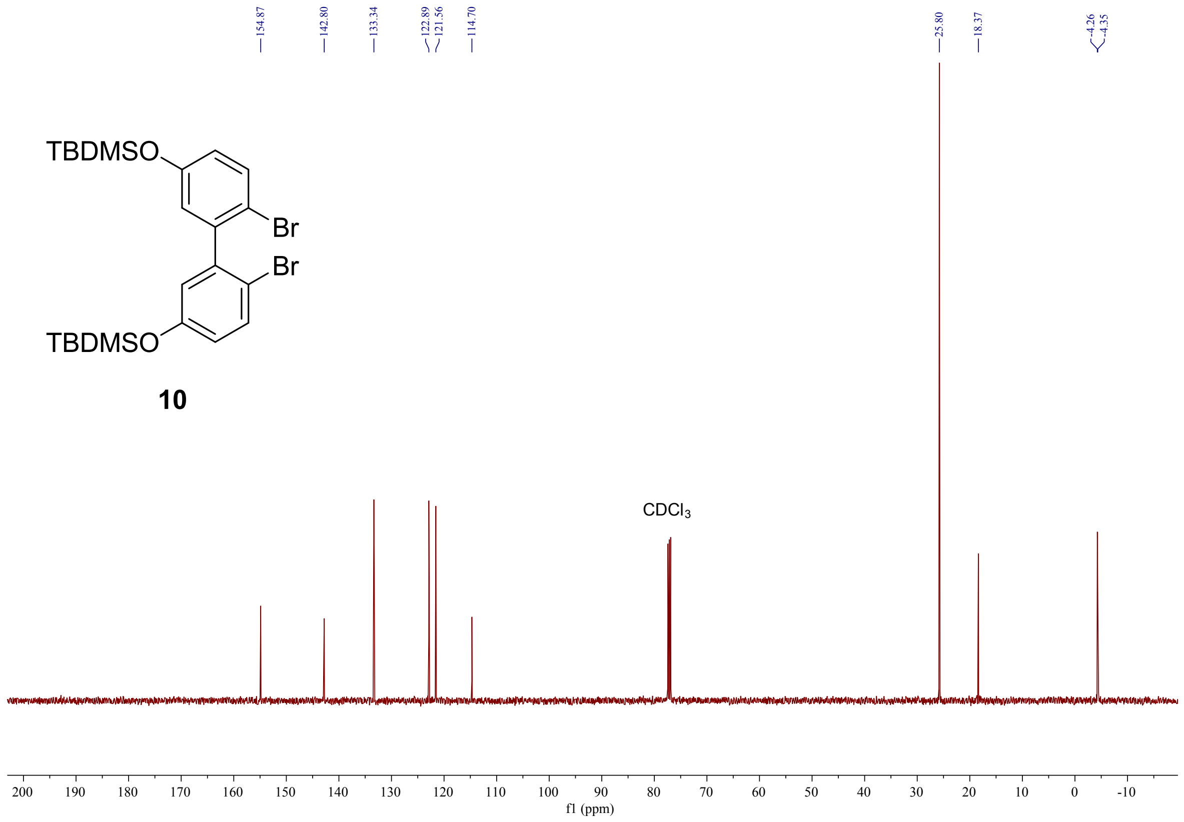

Figure S20: ^{13}C NMR spectrum (126 MHz, $CDCl_3$, 298 K) of compound **10**.

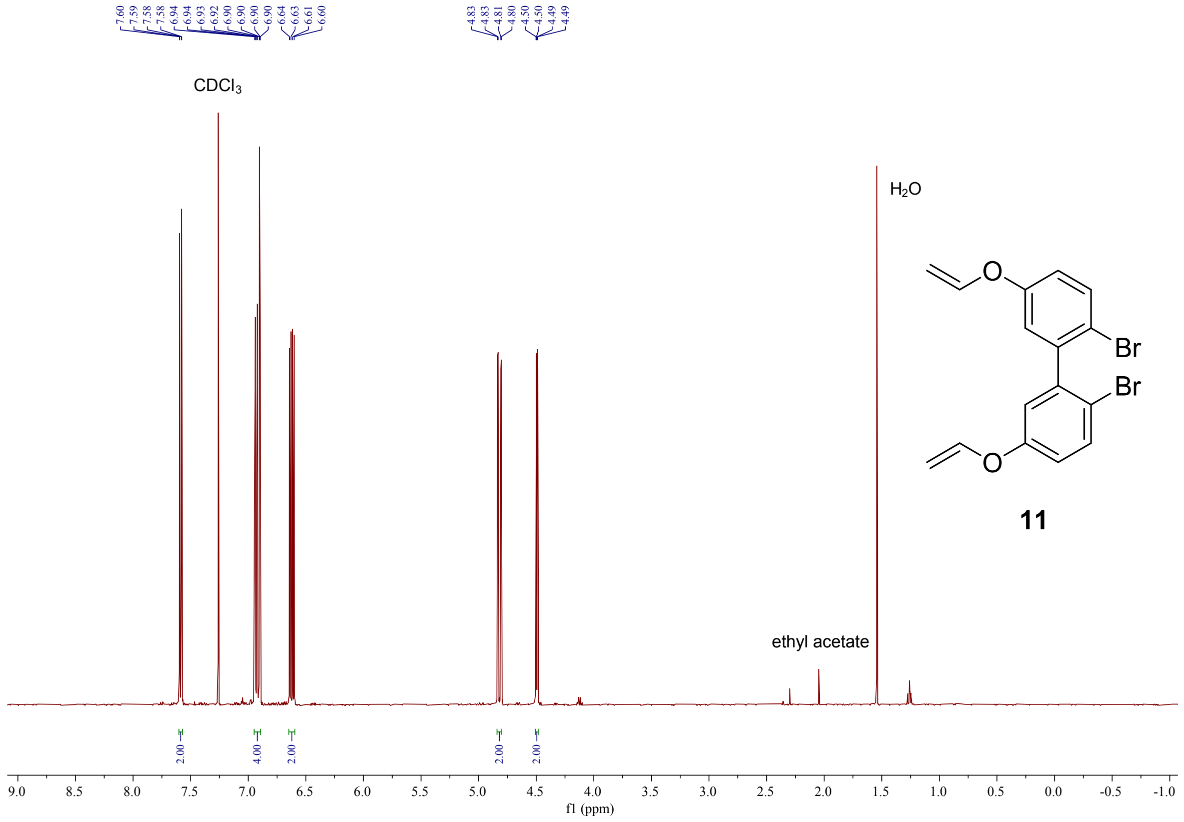

Figure S21: ^{1}H NMR spectrum (500 MHz, $CDCl_3$, 298 K) of compound **11**.

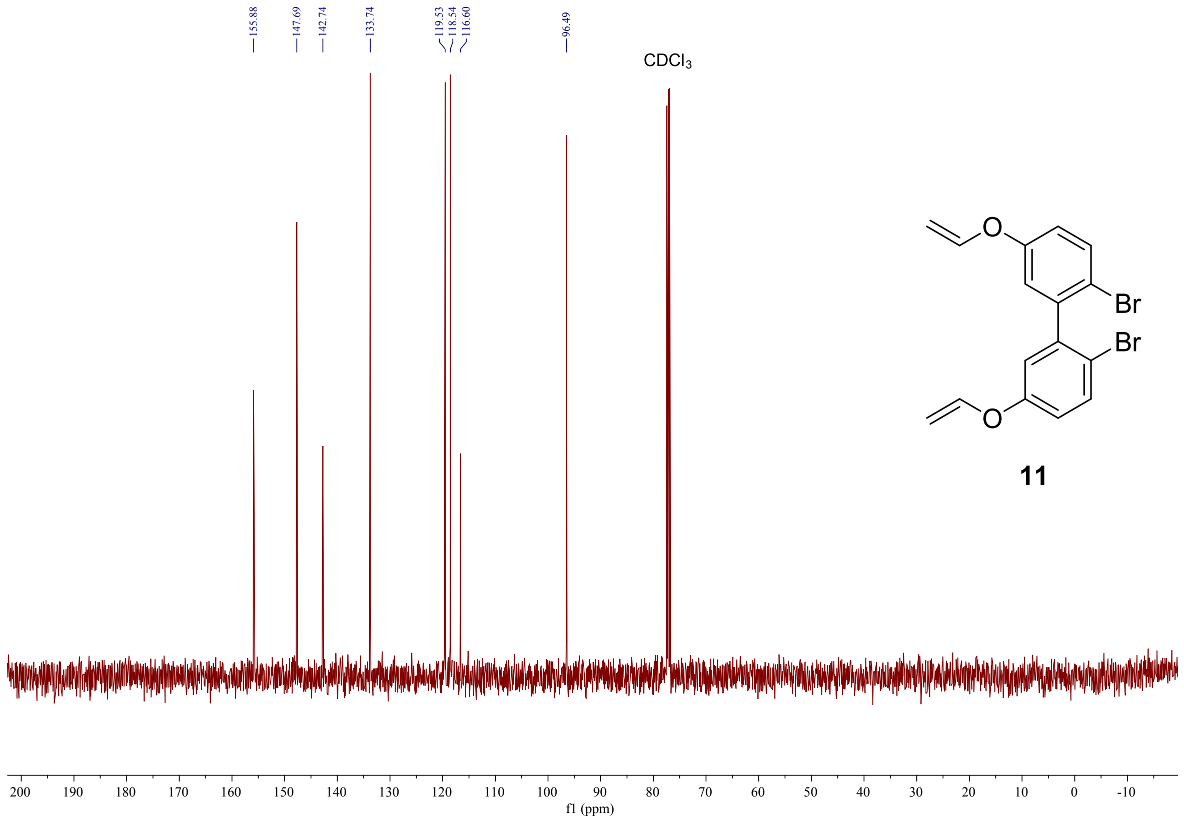

Figure S22: ^{13}C NMR spectrum (126 MHz, $CDCl_3$, 298 K) of compound **11**.

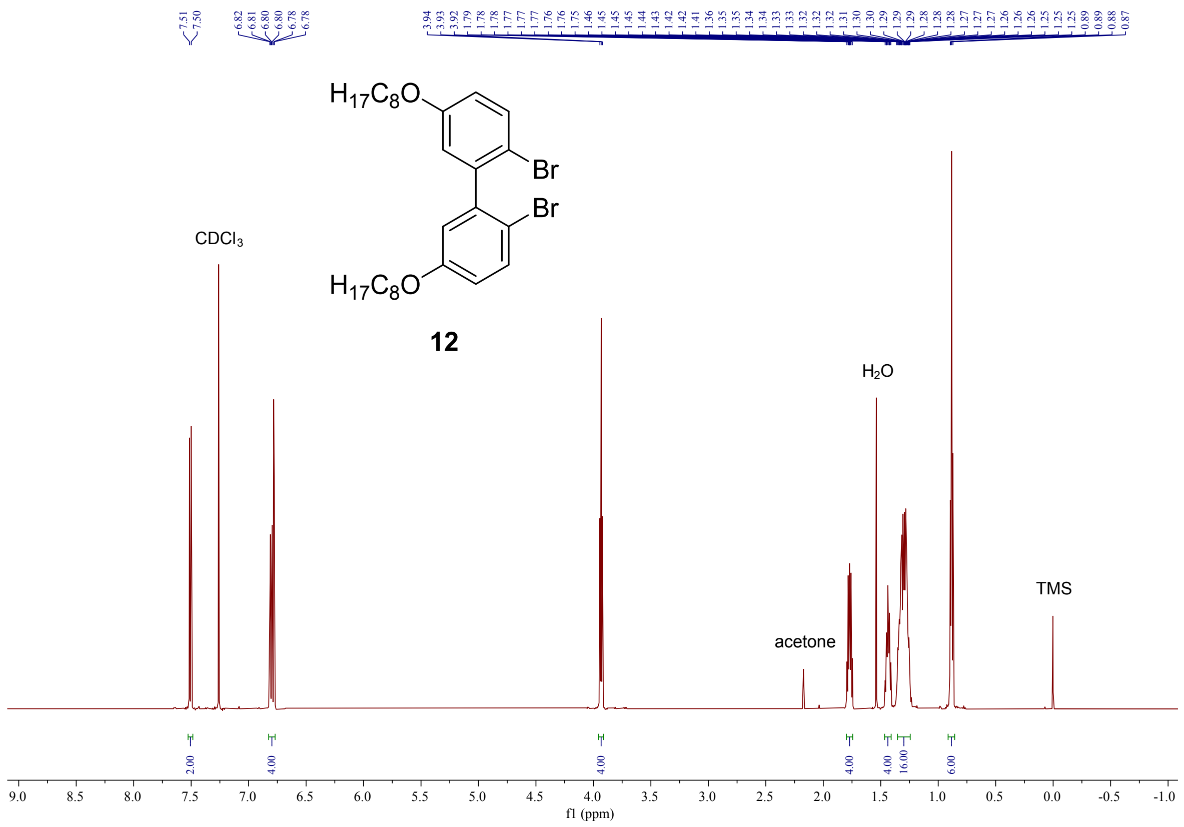

Figure S23: ^{1}H NMR spectrum (600 MHz, $CDCl_3$, 298 K) of compound **12**.

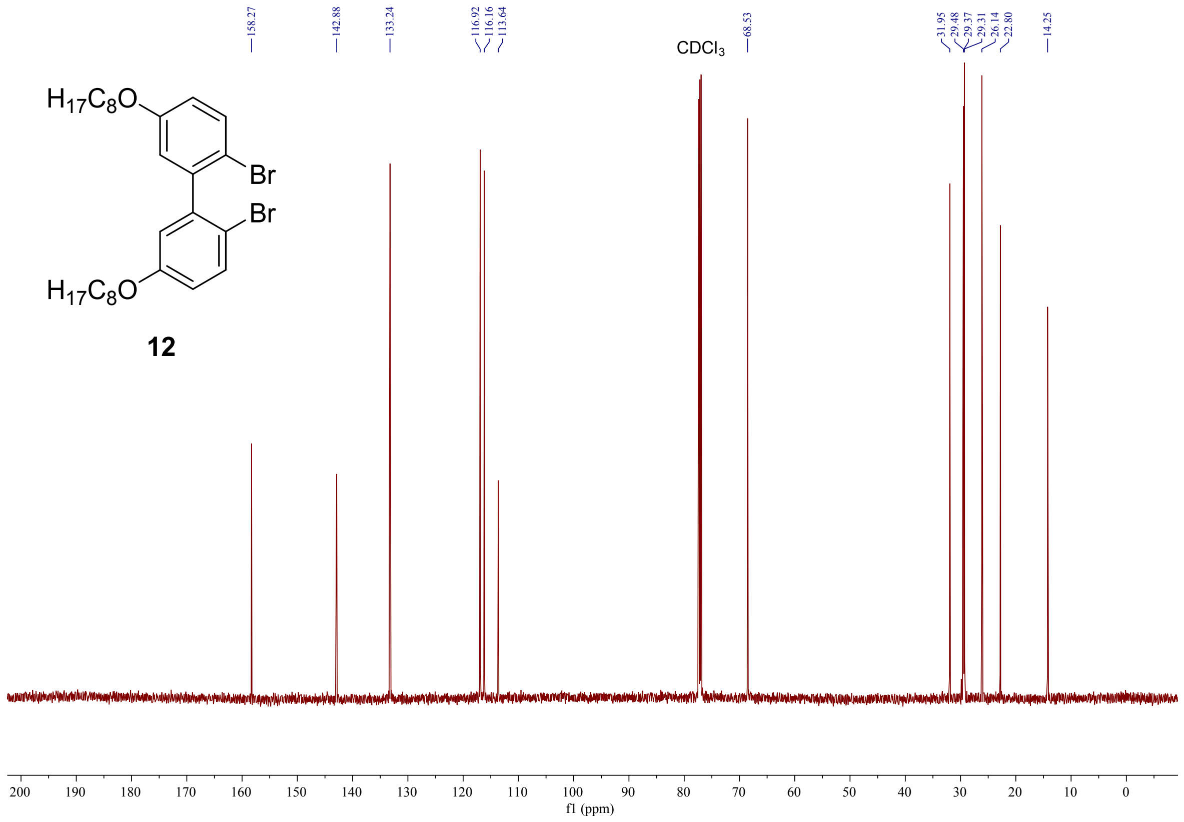

Figure S24: ^{13}C NMR spectrum (151 MHz, $CDCl_3$, 298 K) of compound **12**.

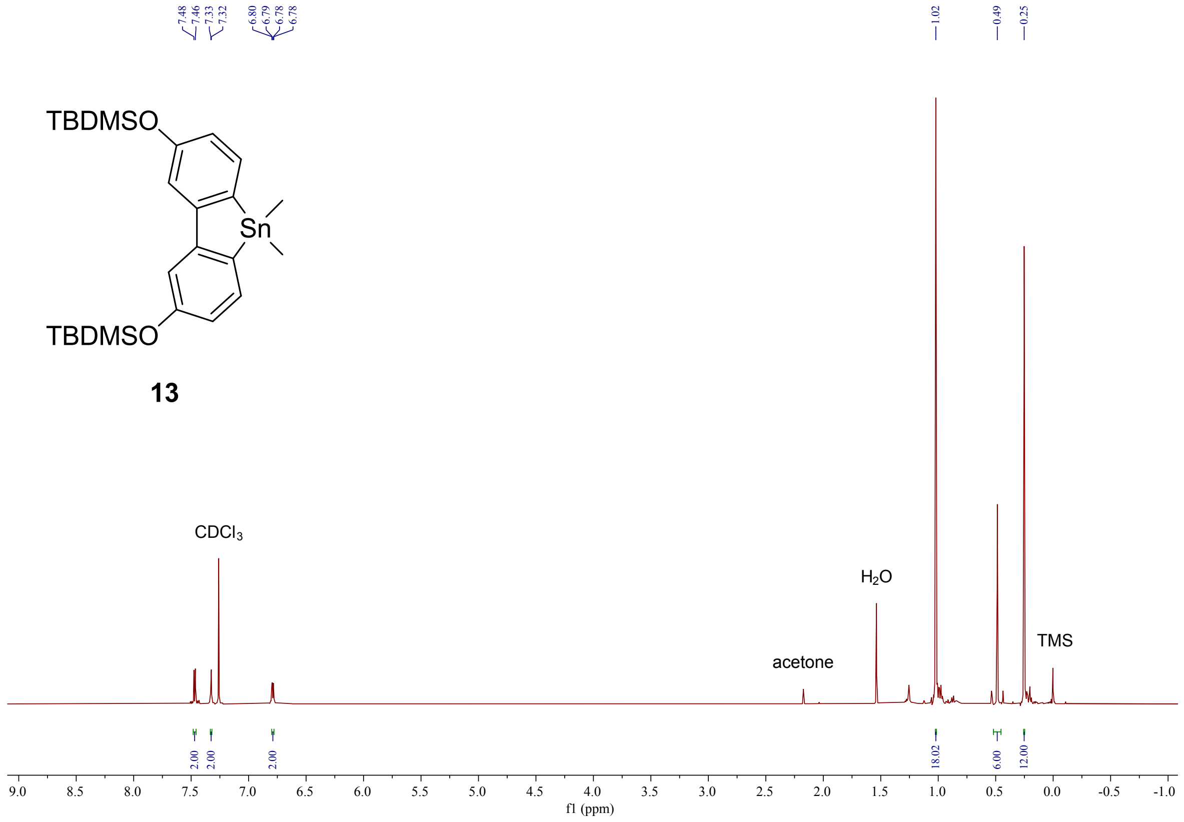

Figure S25: ^{1}H NMR spectrum (600 MHz, $CDCl_3$, 298 K) of compound **13**.

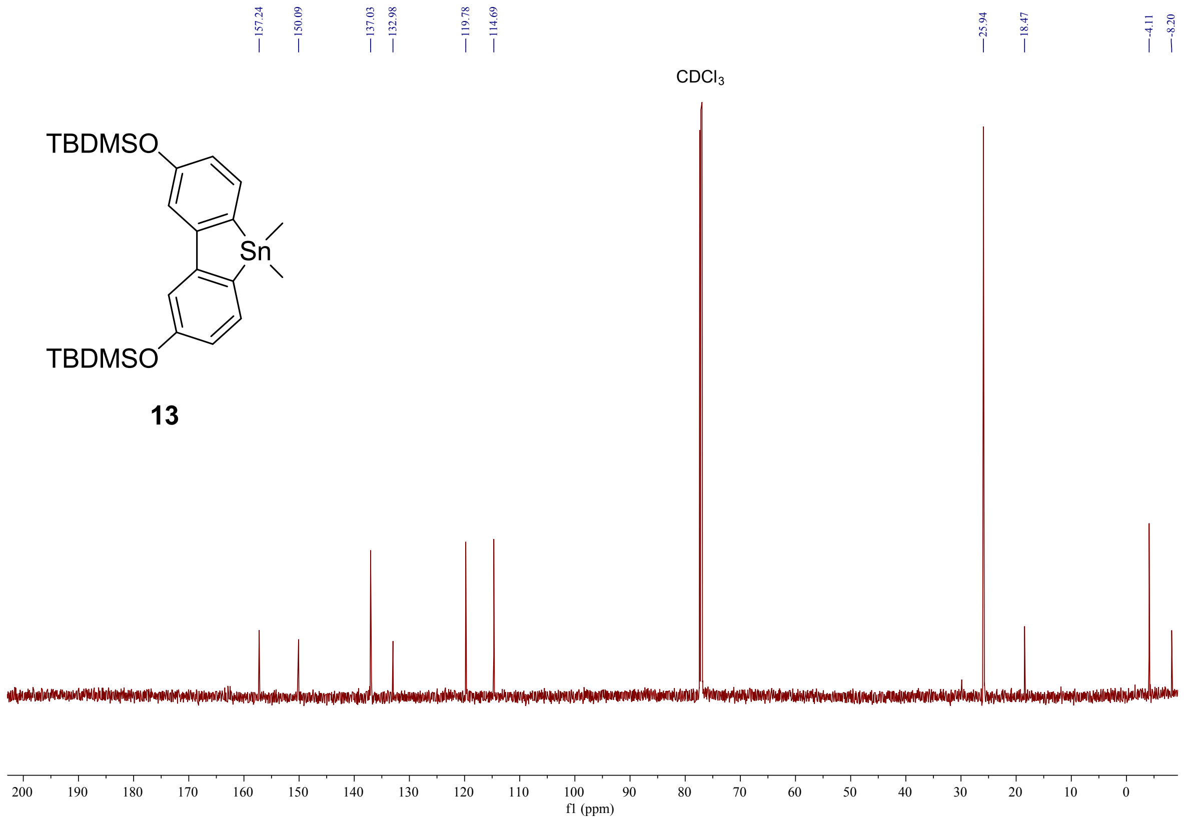

Figure S26: ^{13}C NMR spectrum (151 MHz, $CDCl_3$, 298 K) of compound **13**.

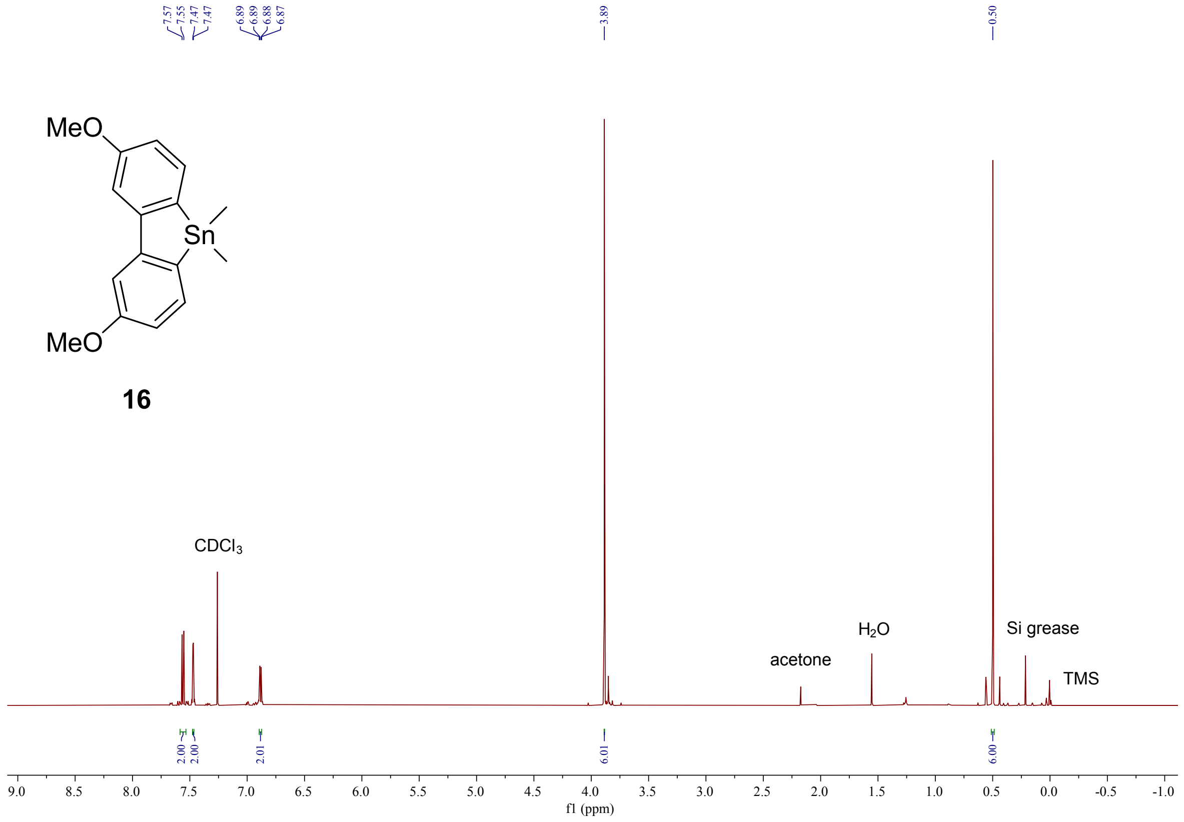

Figure S27: ^{1}H NMR spectrum (500 MHz, $CDCl_3$, 298 K) of compound **16**.

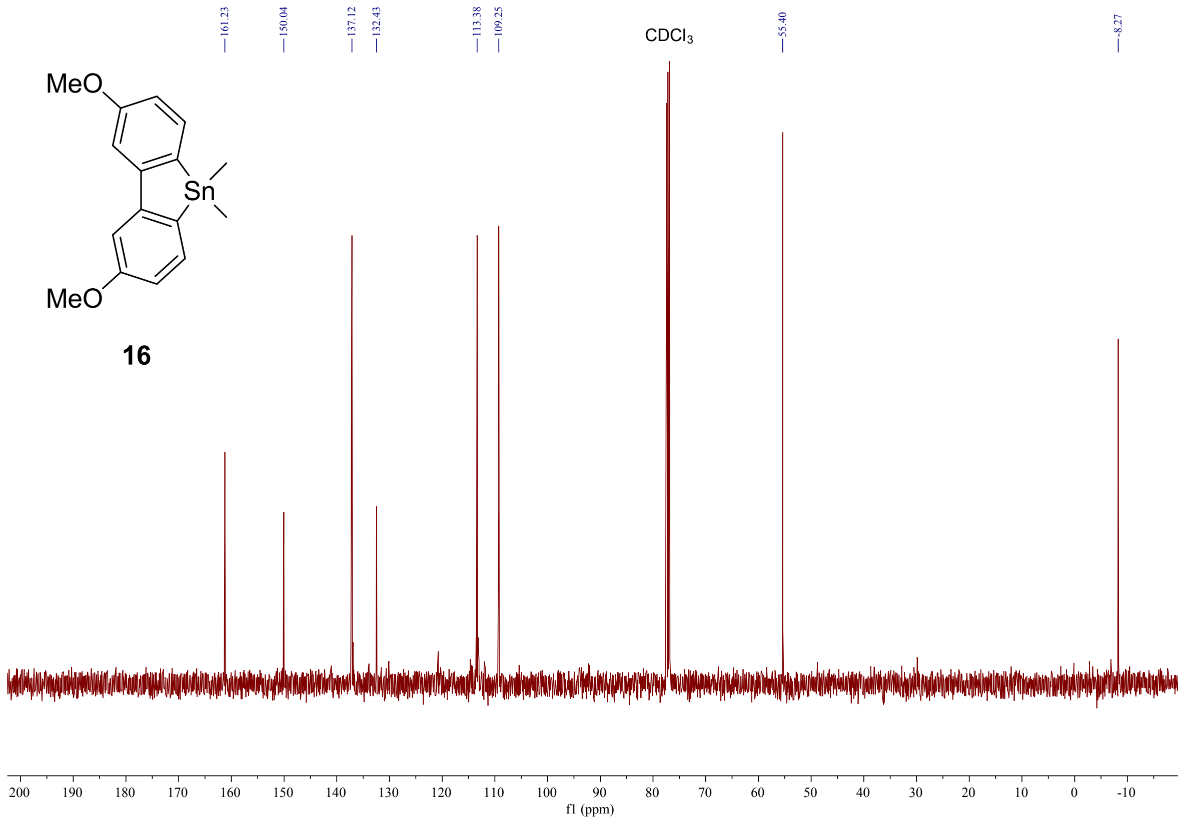

Figure S28: ^{13}C NMR spectrum (126 MHz, $CDCl_3$, 298 K) of compound **16**.

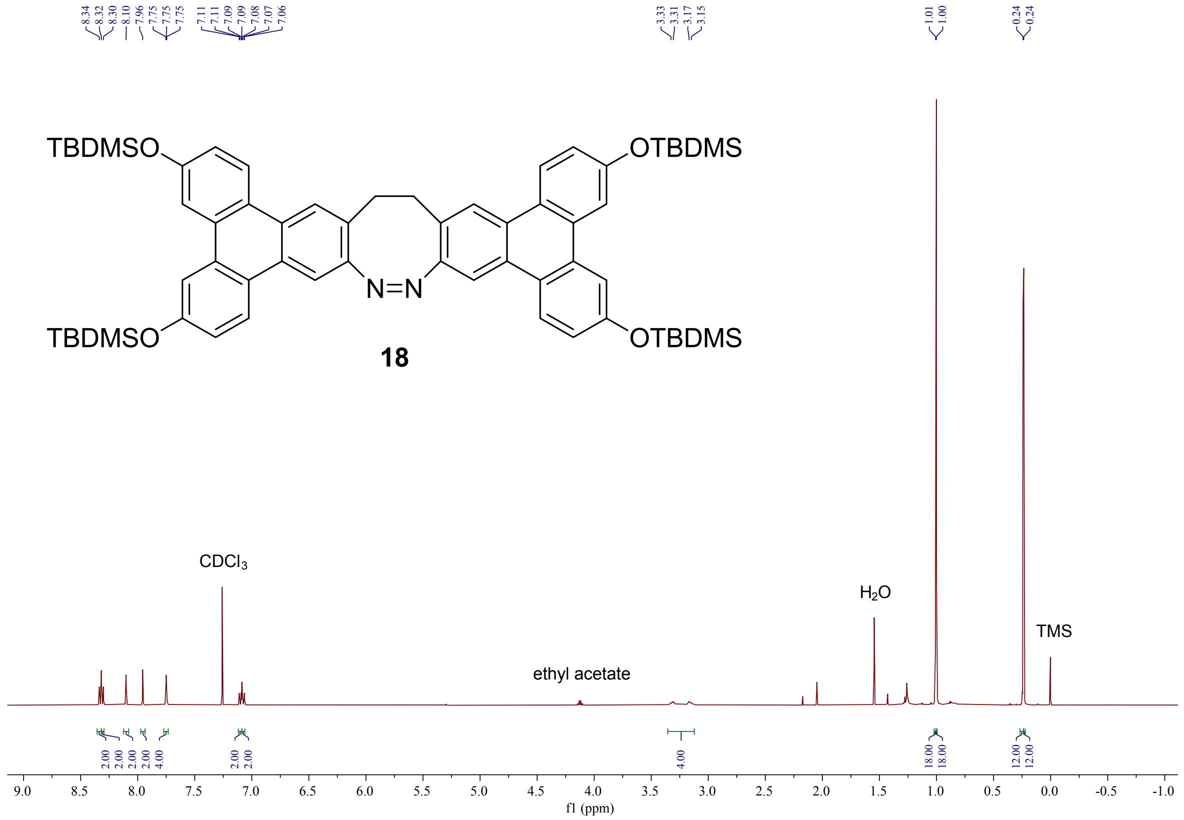

Figure S29: ^{1}H NMR spectrum (500 MHz, $CDCl_3$, 298 K) of compound **18**.

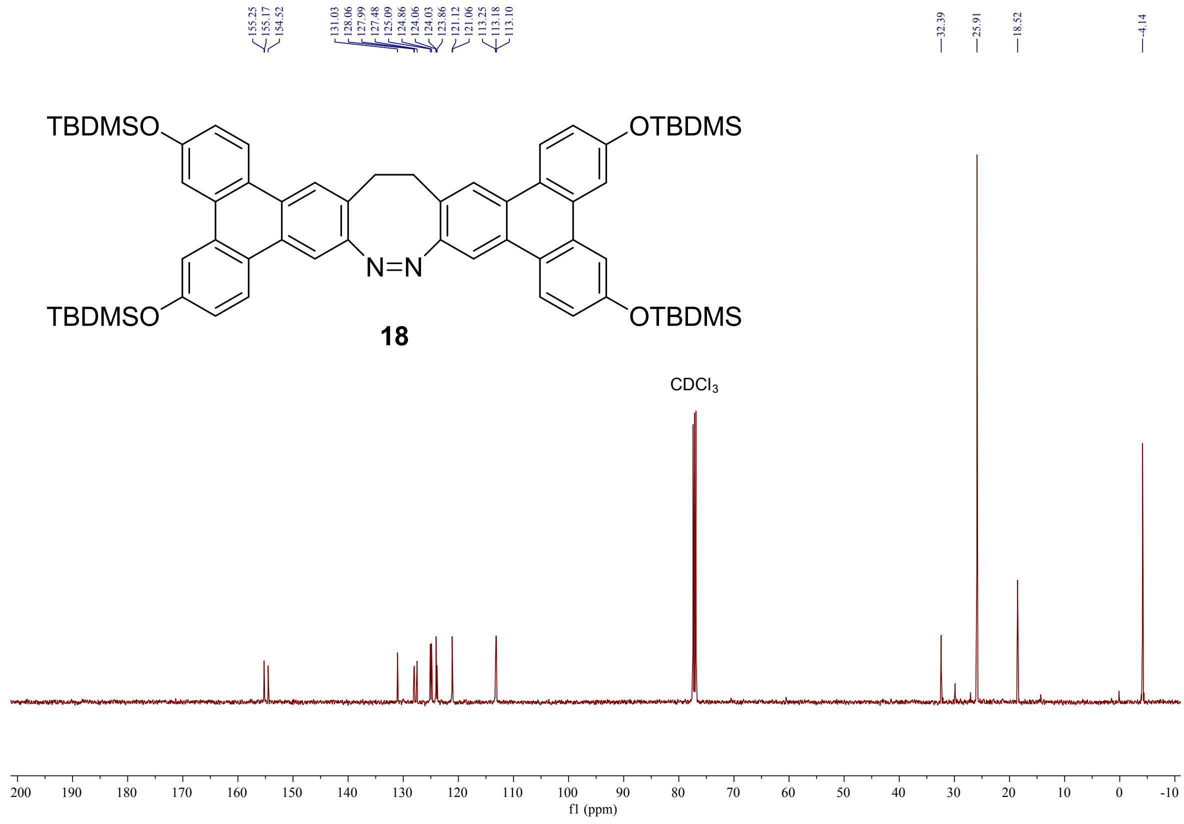

Figure S30: ^{13}C NMR spectrum (126 MHz, $CDCl_3$, 298 K) of compound **18**.

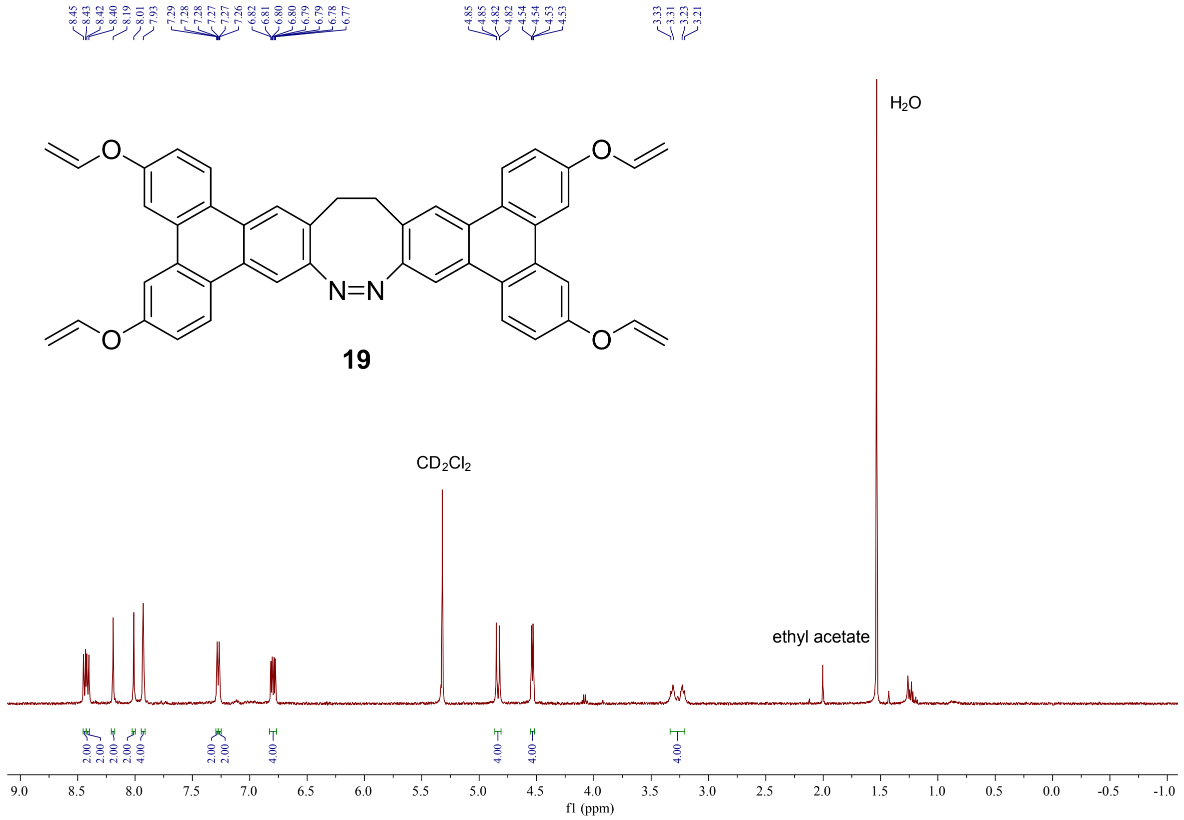

Figure S31: ^{1}H NMR spectrum (500 MHz, CD_2Cl_2, 298 K) of compound **19**.

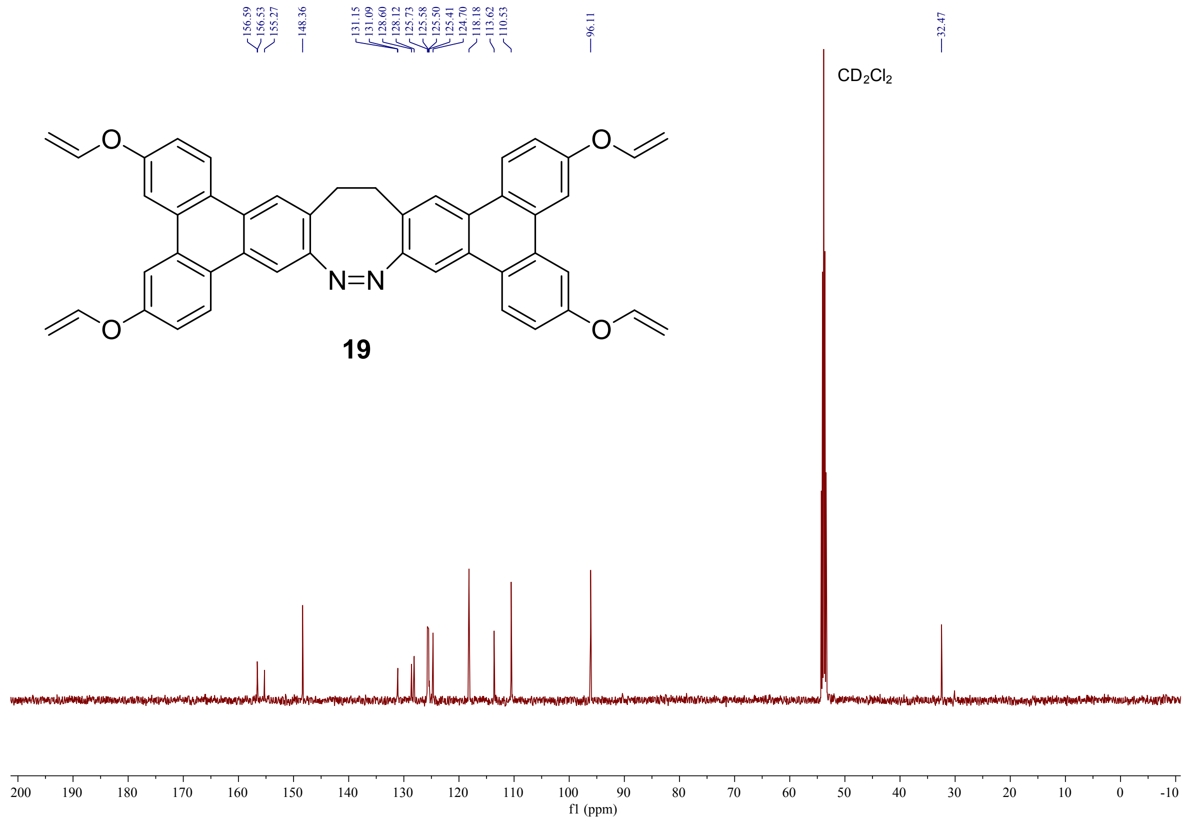

Figure S32: ^{13}C NMR spectrum (126 MHz, CD_2Cl_2, 298 K) of compound **19**.

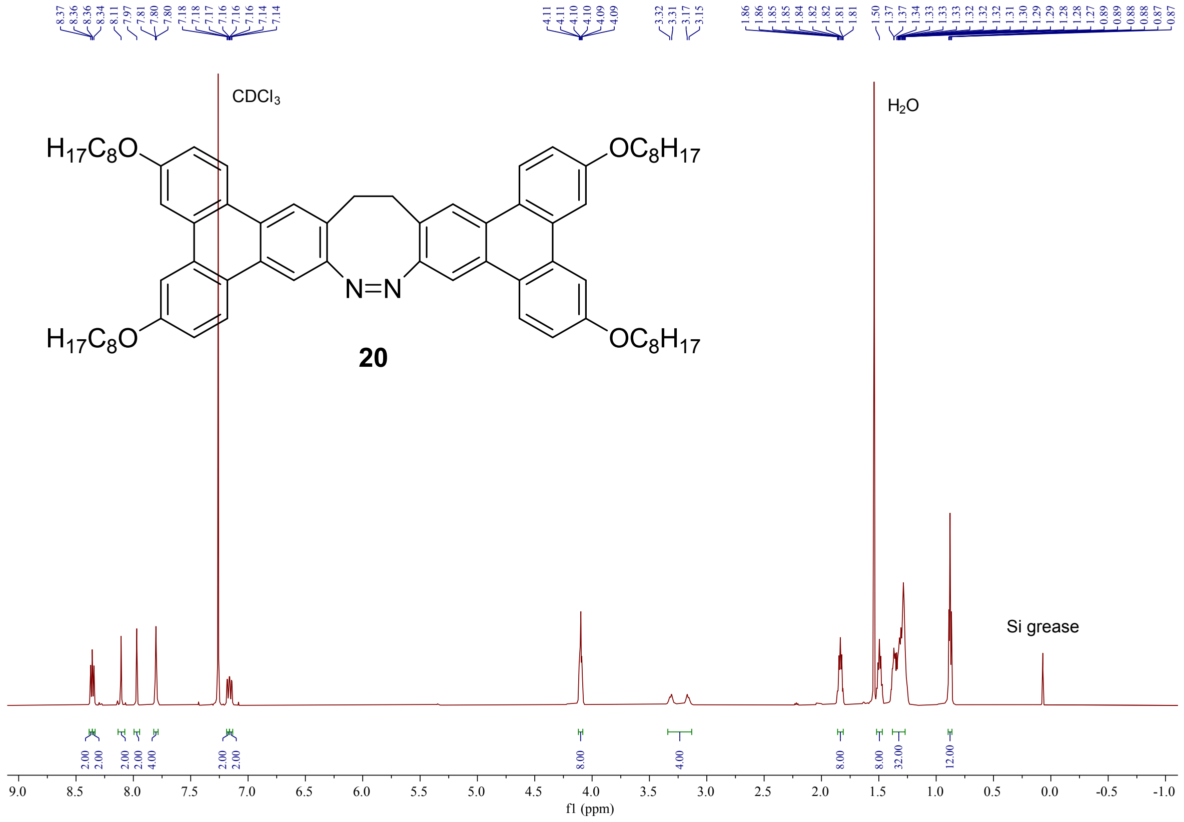

Figure S33: ^{1}H NMR spectrum (600 MHz, $CDCl_3$, 298 K) of compound **20**.

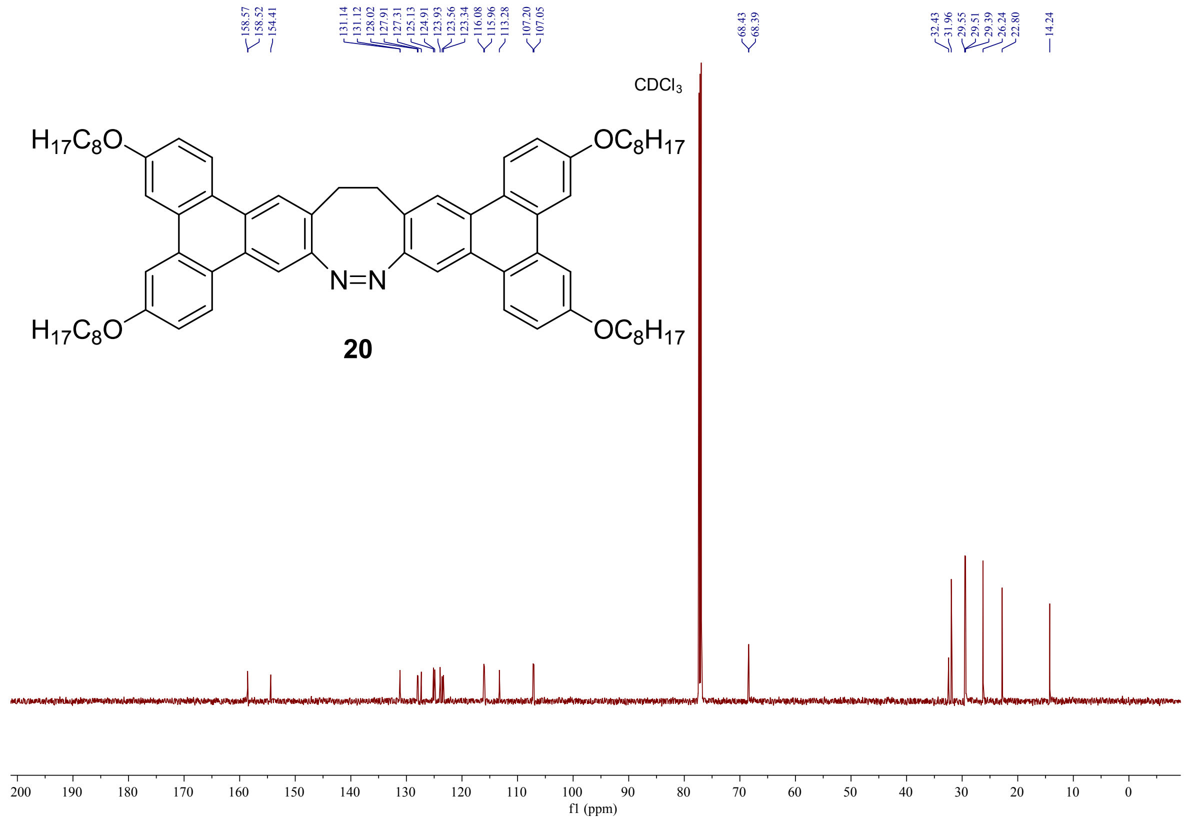

Figure S34: ^{13}C NMR spectrum (151 MHz, $CDCl_3$, 298 K) of compound **20**.

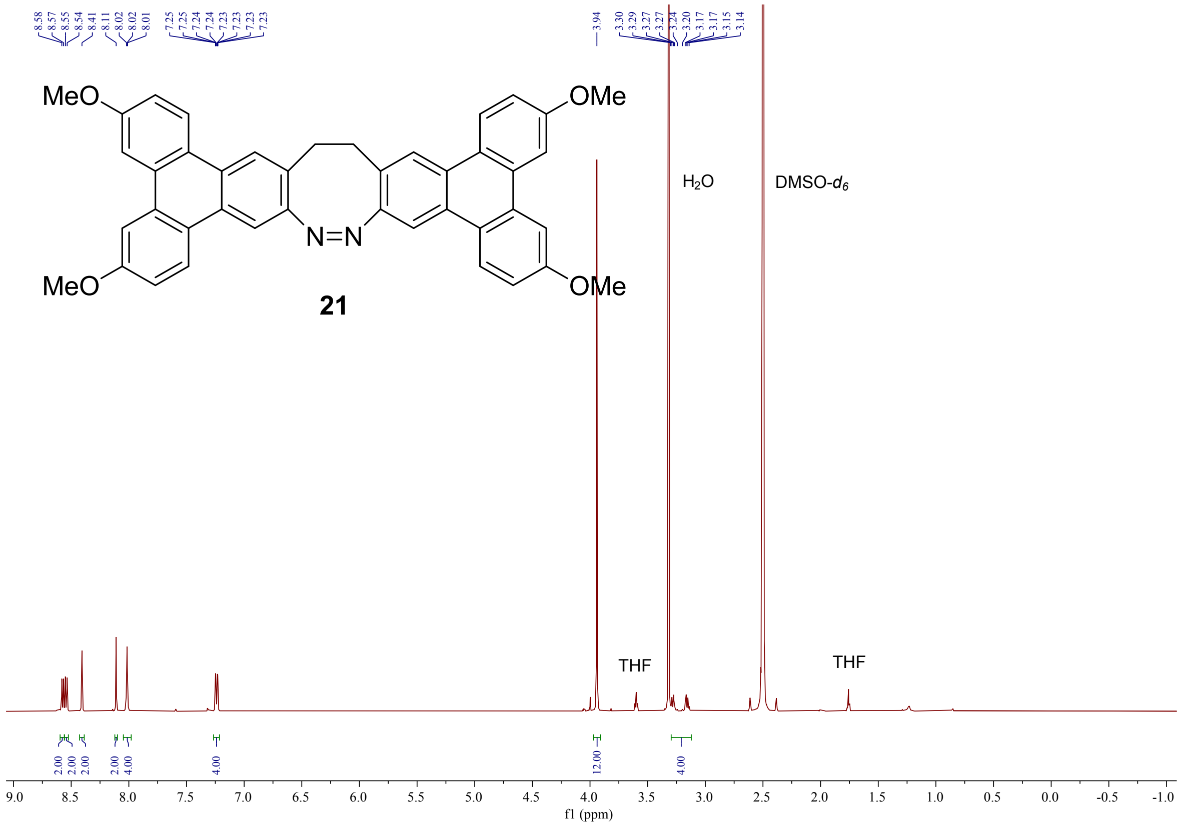

Figure S35: ^{1}H NMR spectrum (600 MHz, DMSO-d_6, 298 K) of compound **21**.

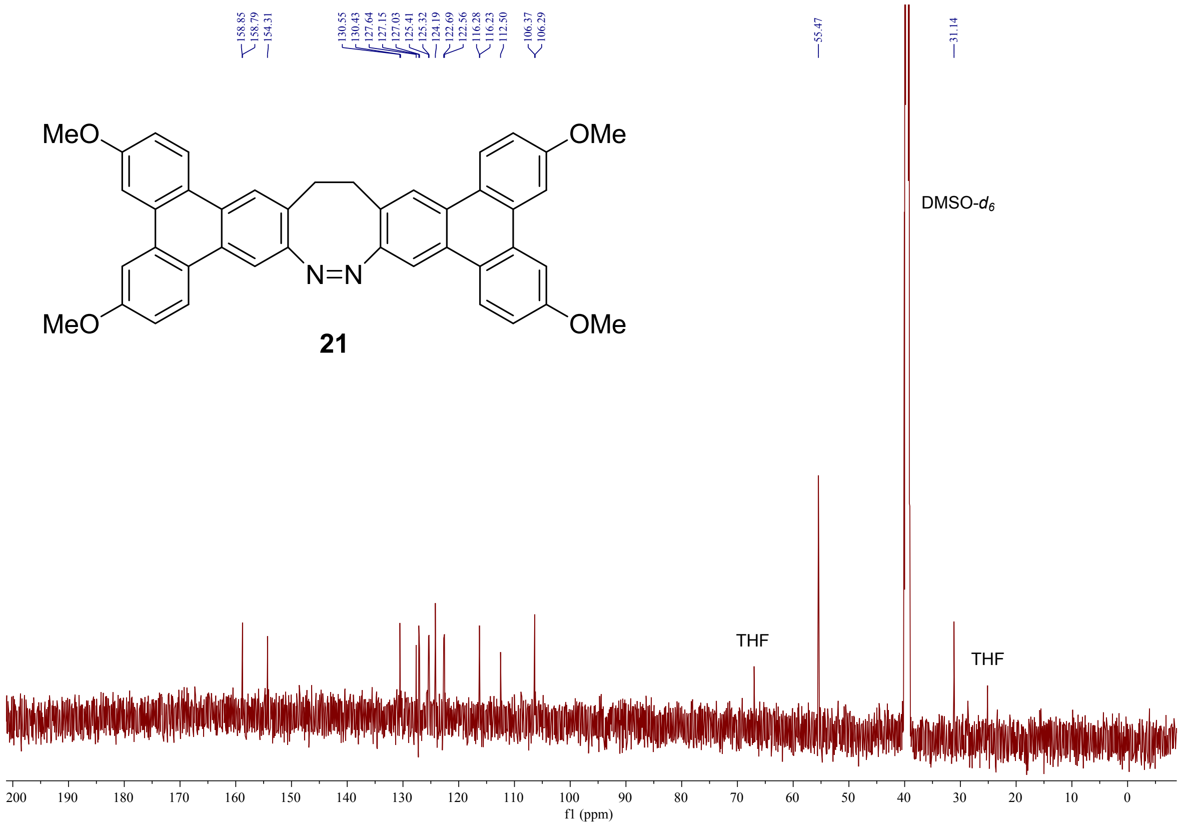

Figure S36: ^{13}C NMR spectrum (151 MHz, DMSO-d_6, 298 K) of compound **21**.

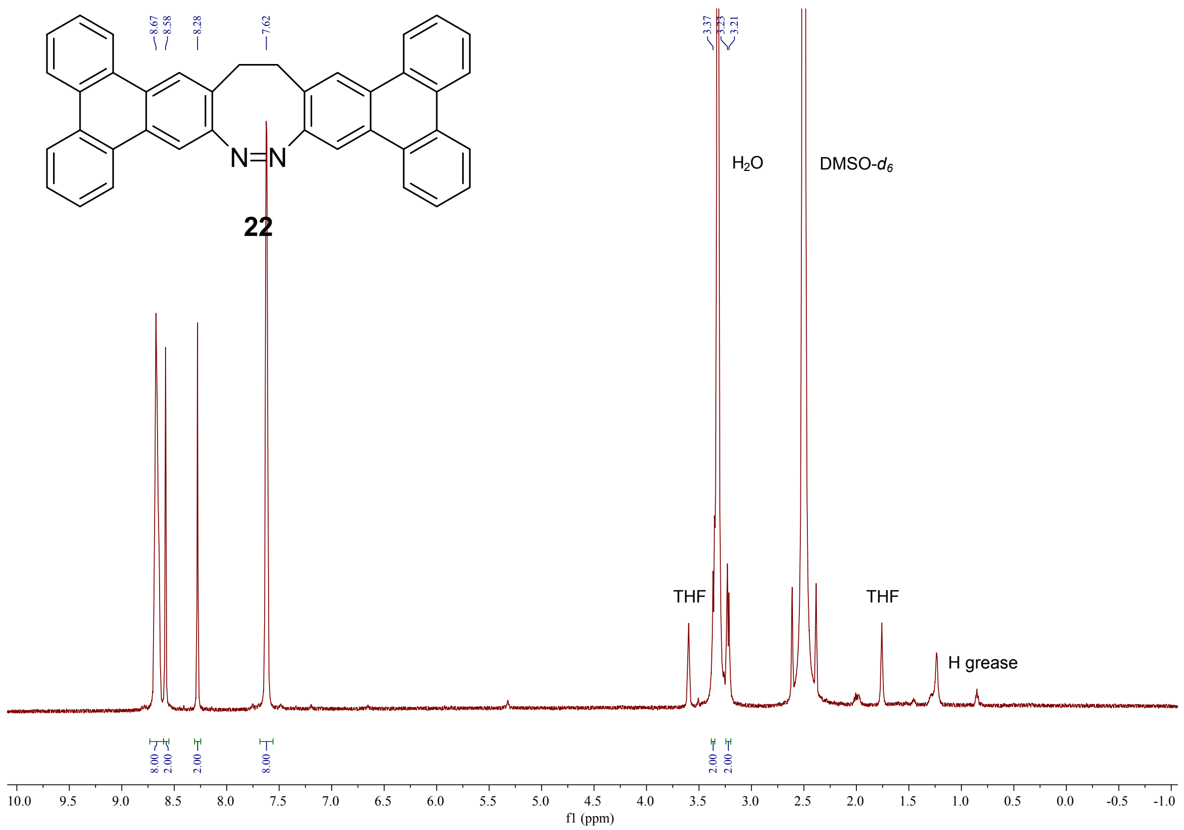

Figure S37: ^{1}H NMR spectrum (600 MHz, DMSO-d_6, 298 K) of compound **22**.

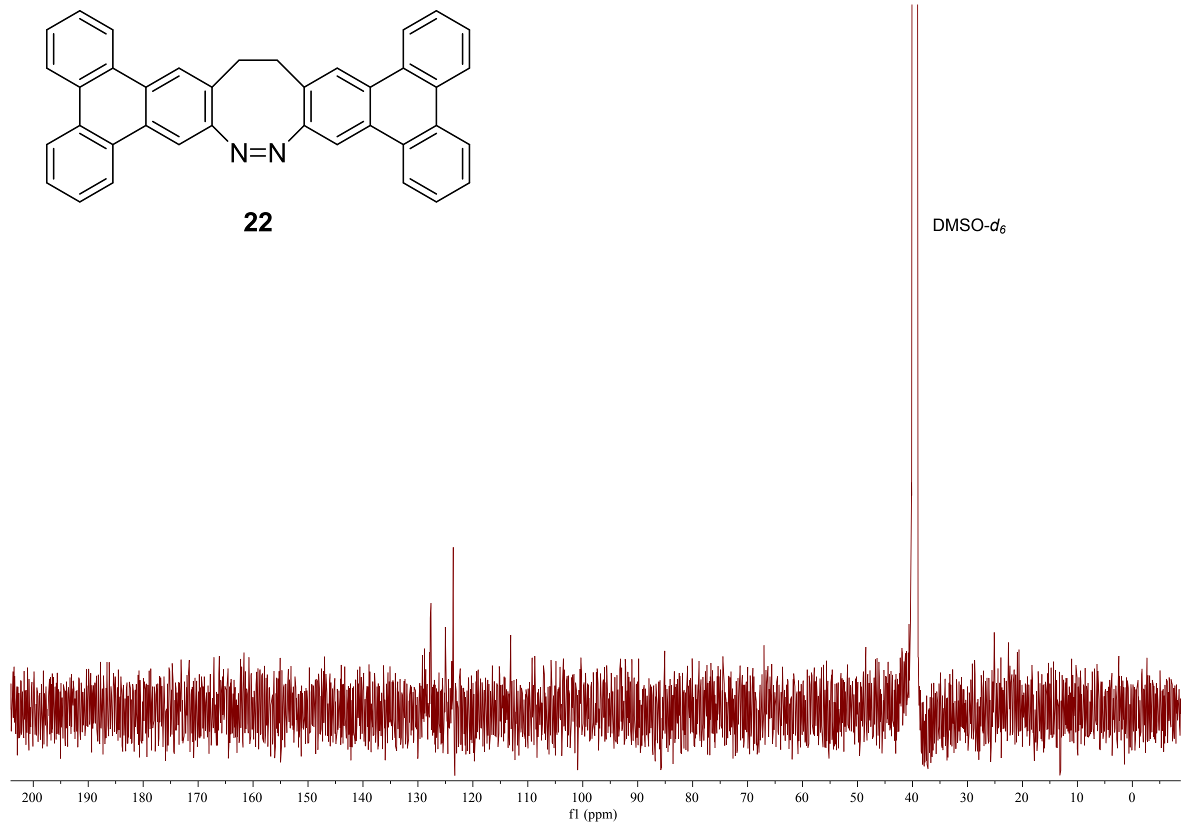

Figure S38: ^{13}C NMR spectrum (151 MHz, DMSO-d_6, 298 K) of compound **22**. The low signal intensity is due to the rapid precipitation of the compound **22**.

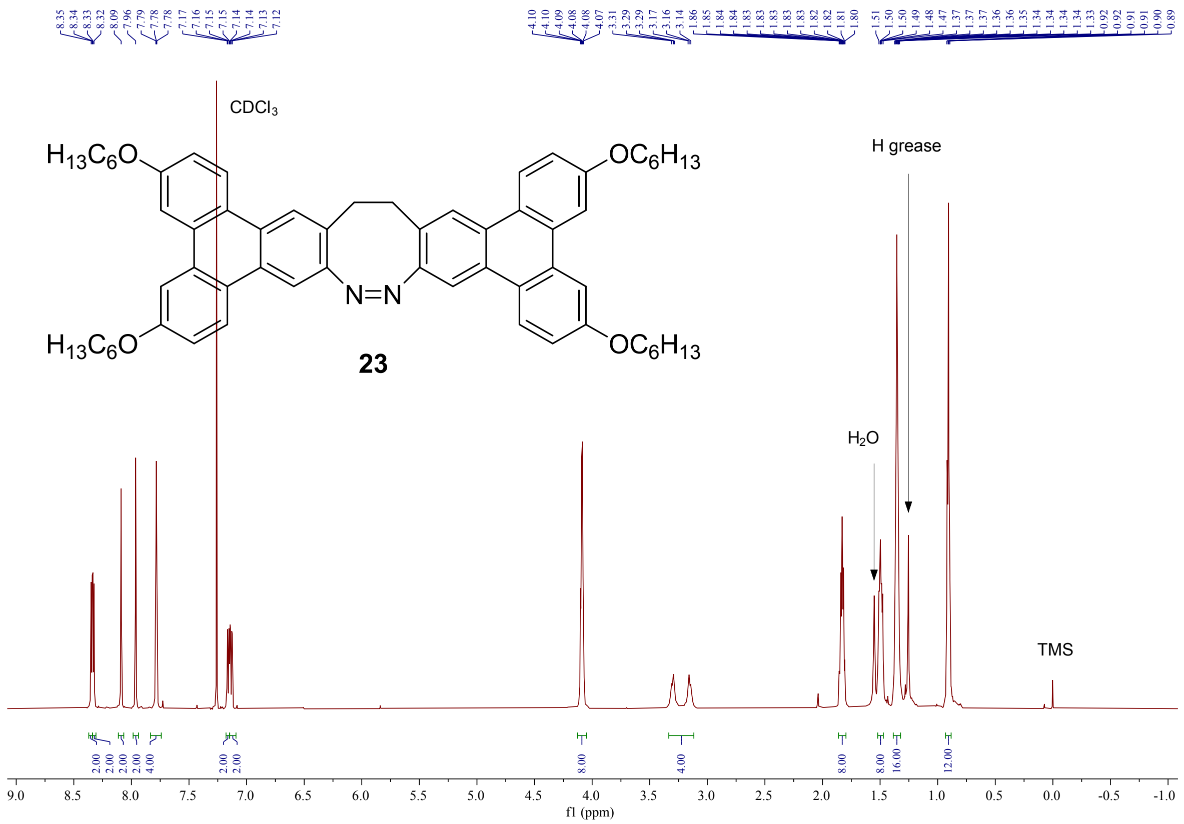

Figure S39: ^{1}H NMR spectrum (600 MHz, $CDCl_3$, 298 K) of compound **23**.

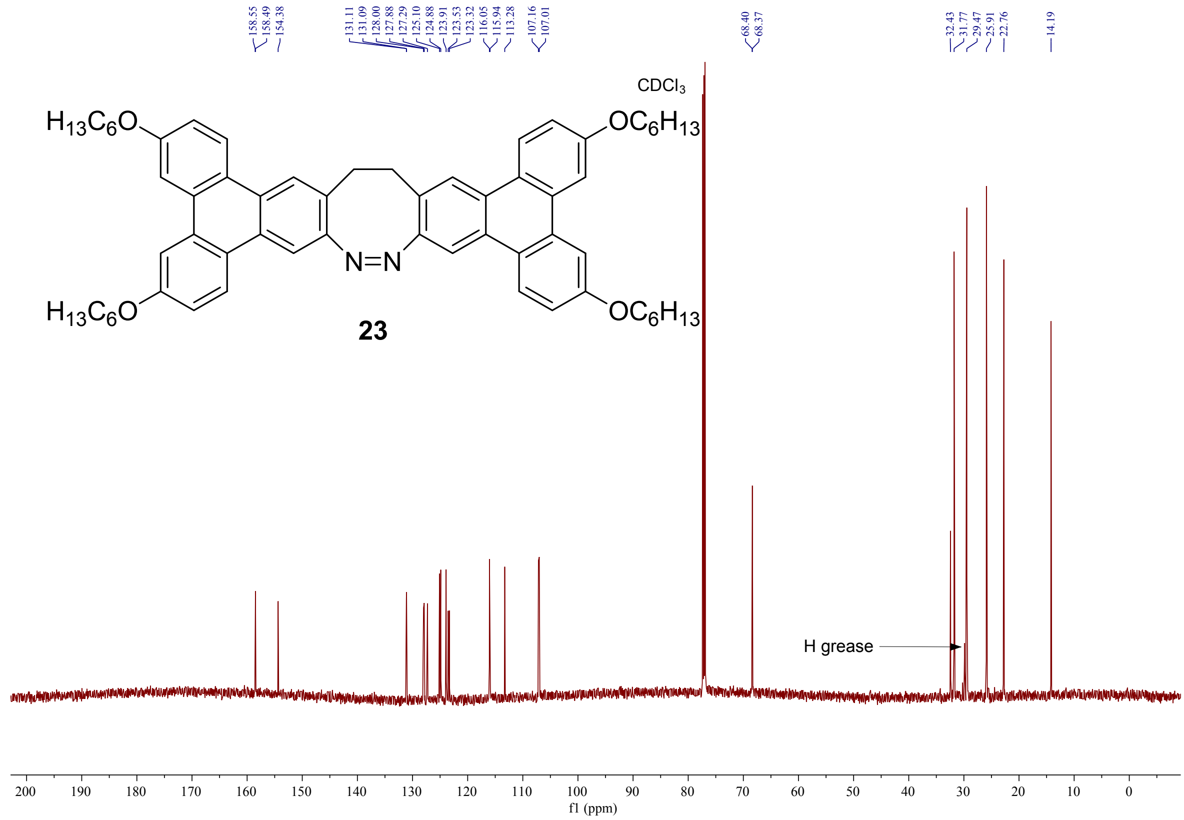

Figure S40: ^{13}C NMR spectrum (151 MHz, $CDCl_3$, 298 K) of compound **23**.

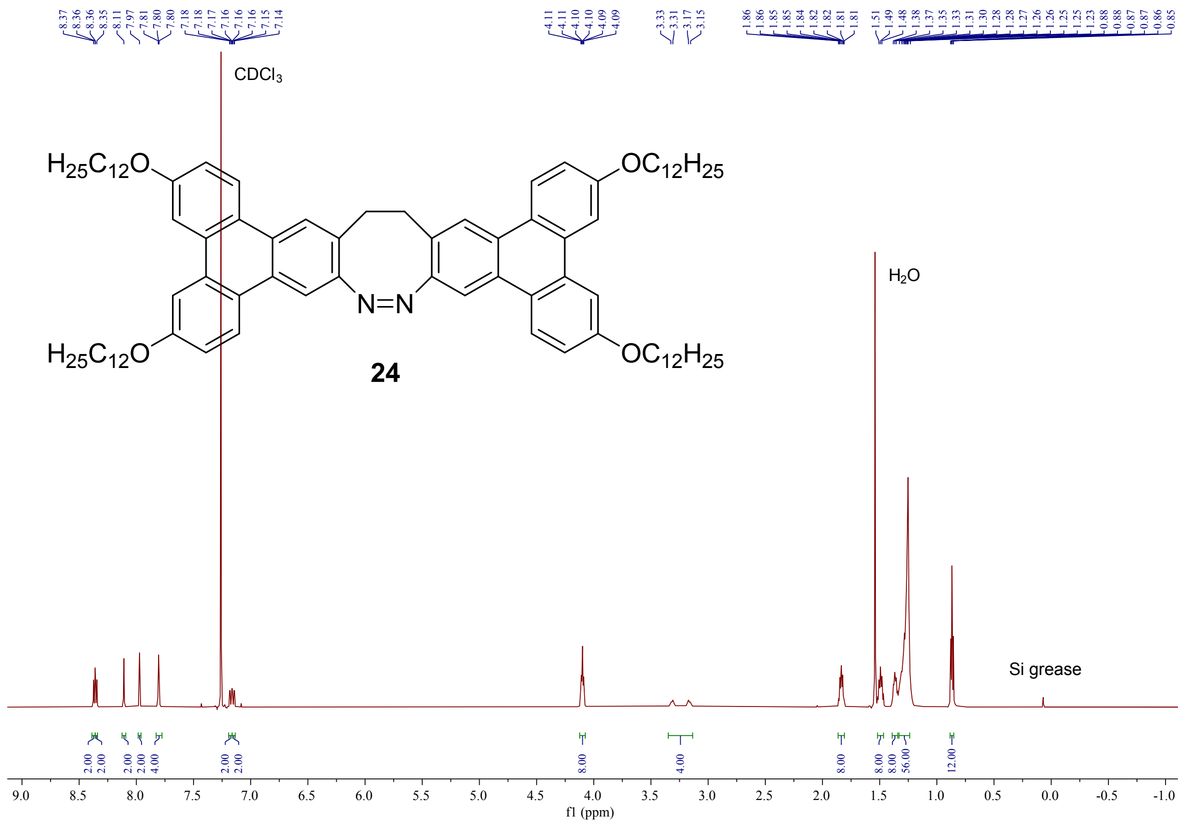

Figure S41: ^{1}H NMR spectrum (600 MHz, $CDCl_3$, 298 K) of compound **24**.

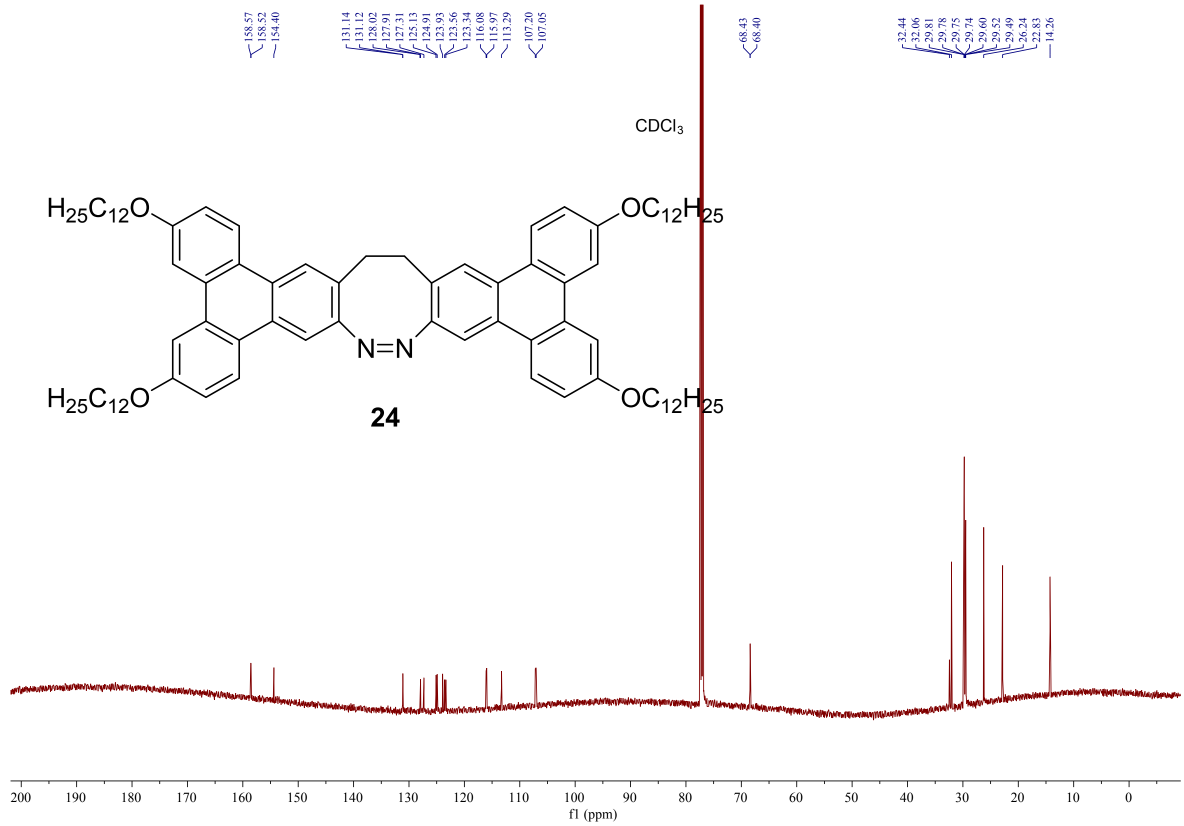

Figure S42: ^{13}C NMR spectrum (151 MHz, $CDCl_3$, 298 K) of compound **24**.

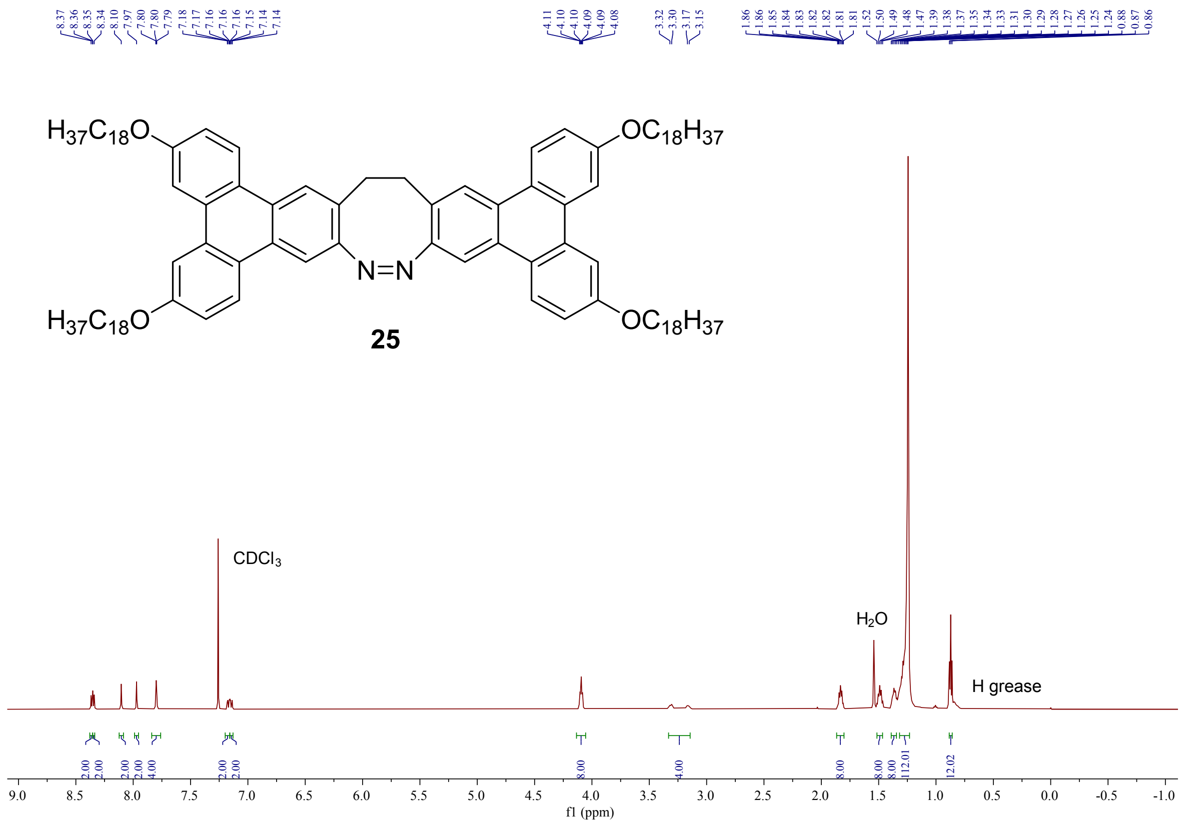

Figure S43: ^{1}H NMR spectrum (600 MHz, $CDCl_3$, 298 K) of compound **25**.

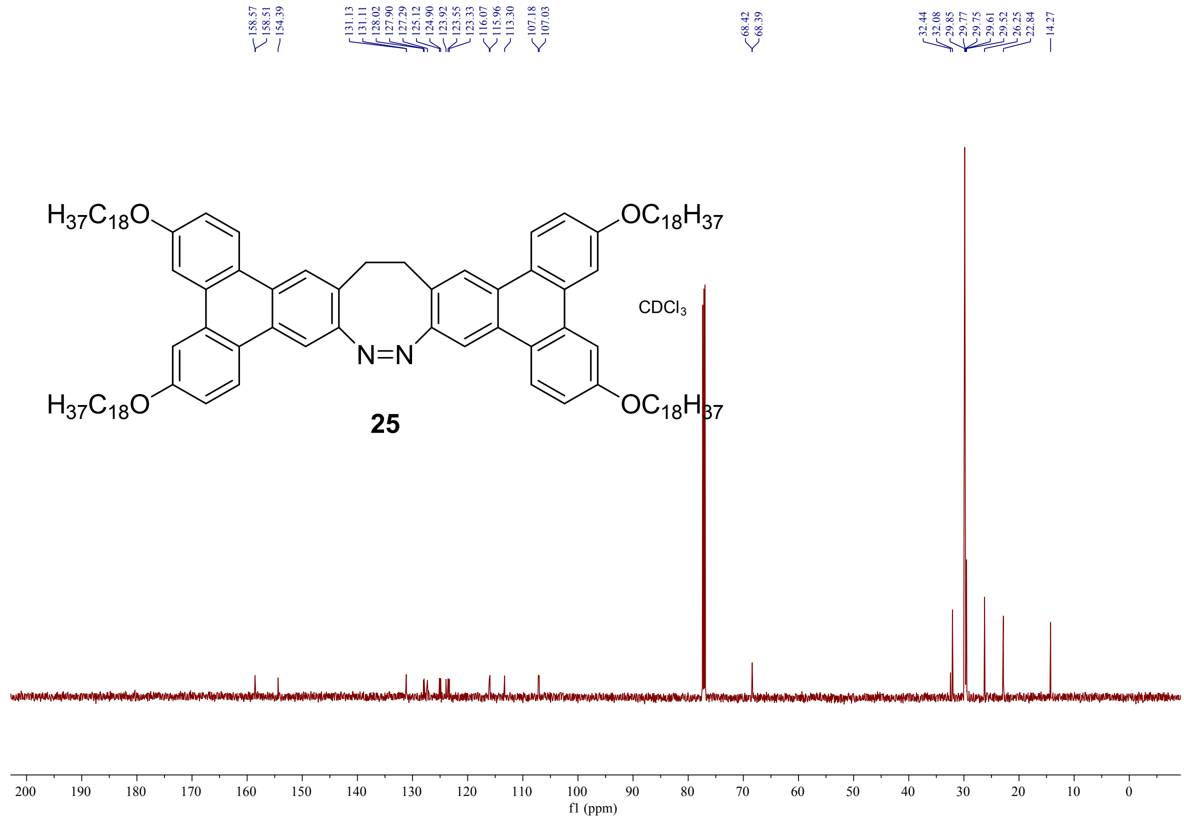

Figure S44: ^{13}C NMR spectrum (151 MHz, $CDCl_3$, 298 K) of compound **25**.

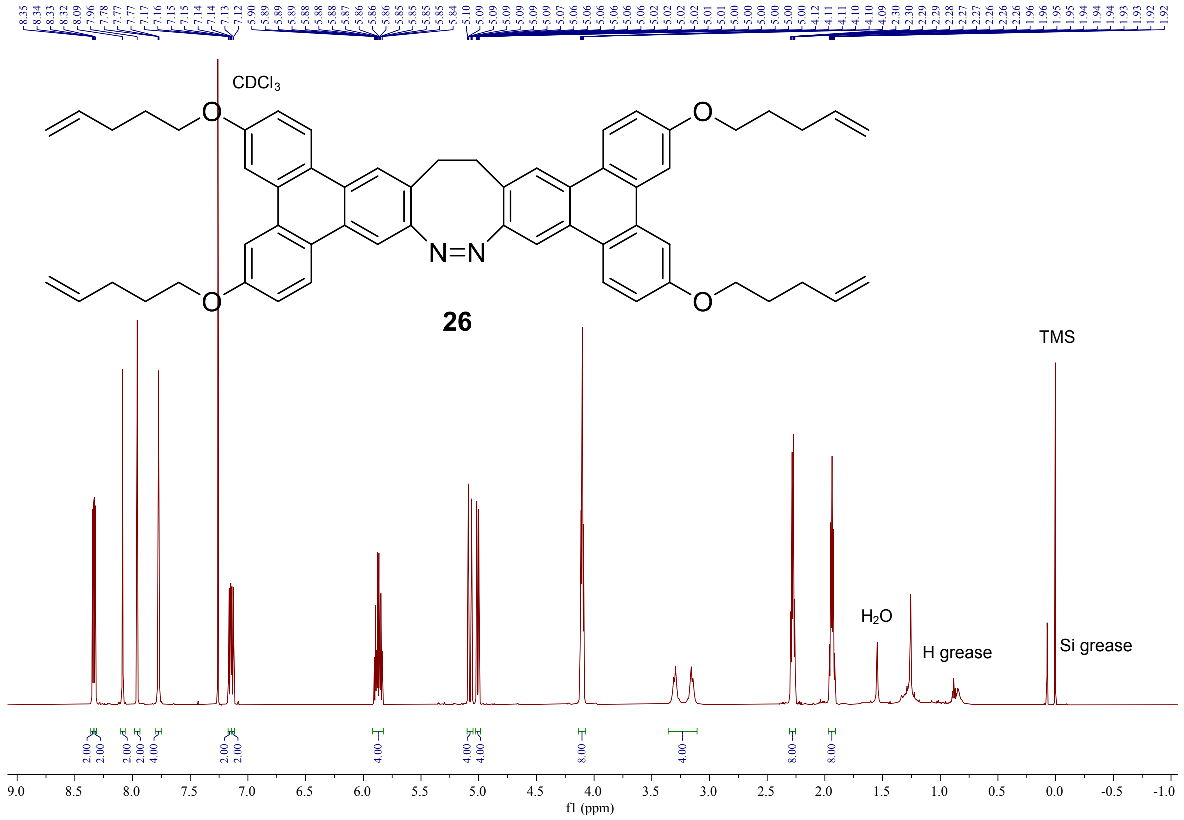

Figure S45: ^{1}H NMR spectrum (600 MHz, $CDCL_3$, 298 K) of compound **26**.

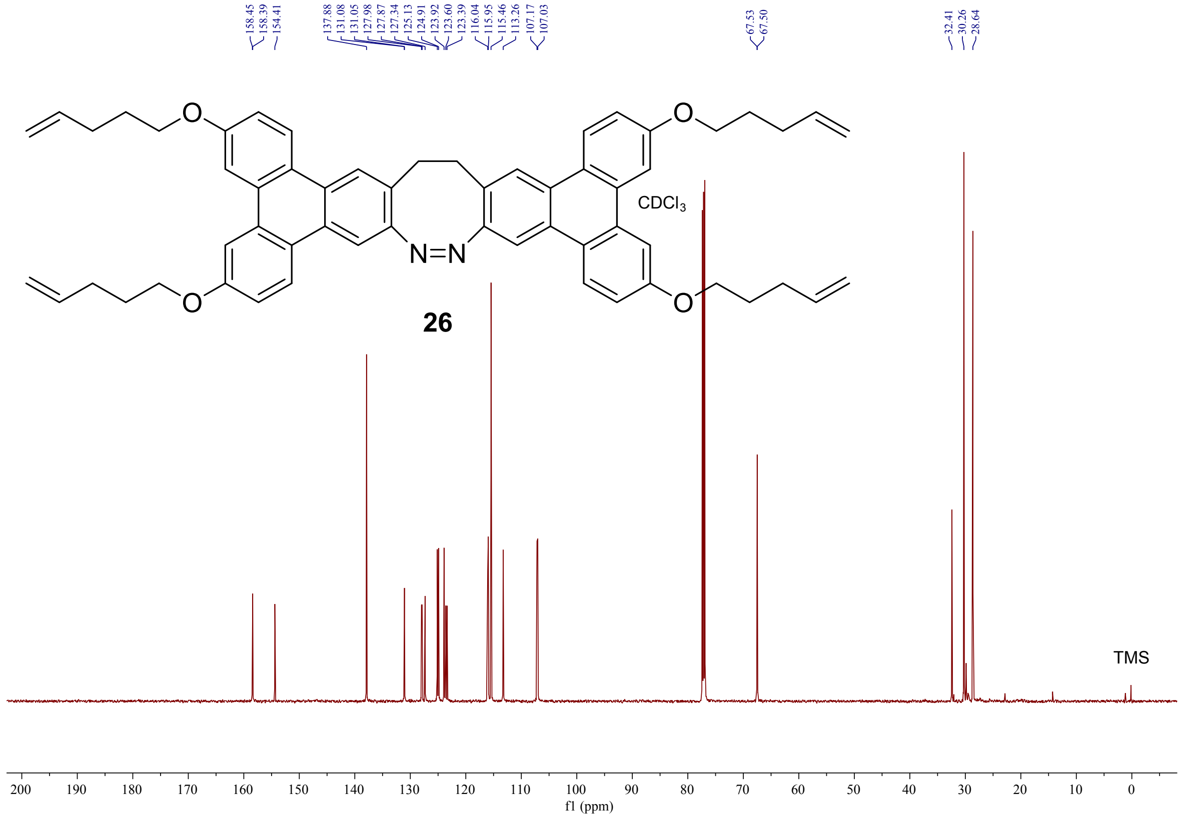

Figure S46: ^{13}C NMR spectrum (151 MHz, $CDCl_3$, 298 K) of compound **26**.

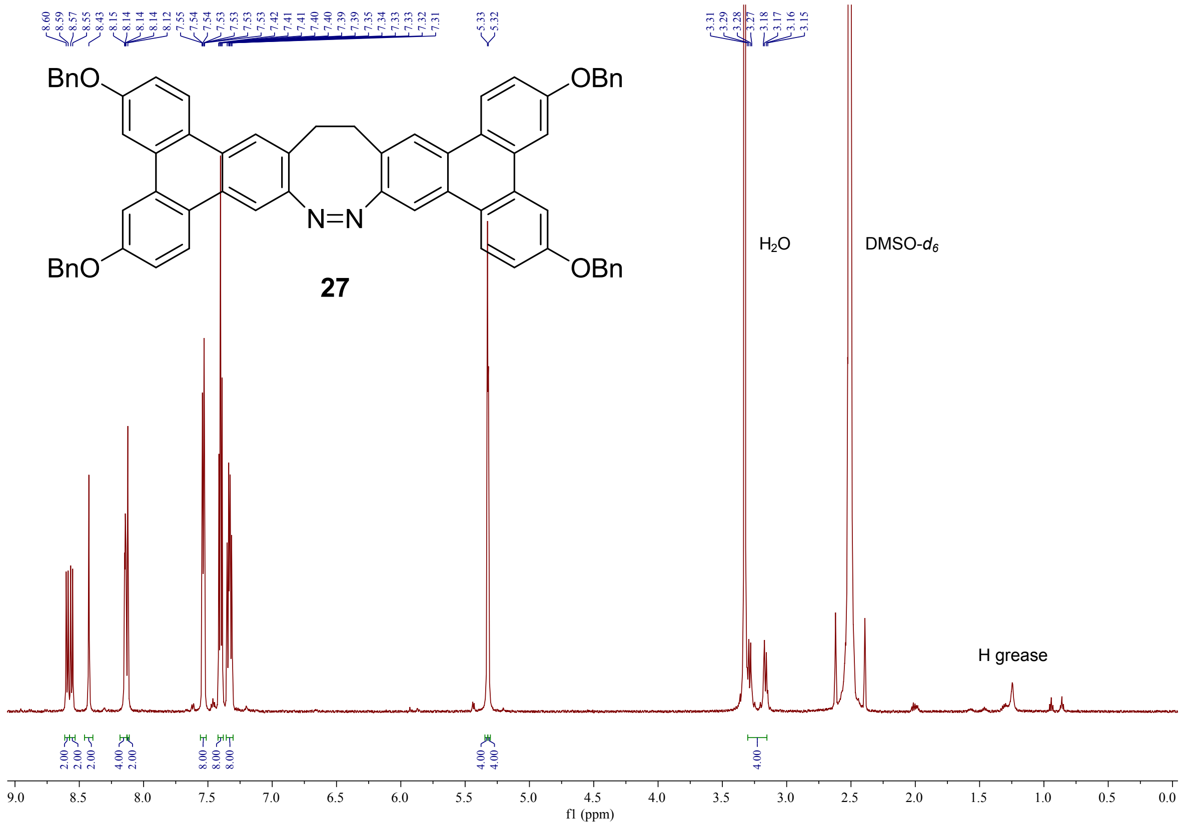

Figure S47: ^1H NMR spectrum (600 MHz, $CDCl_3$, 298 K) of compound **27**.

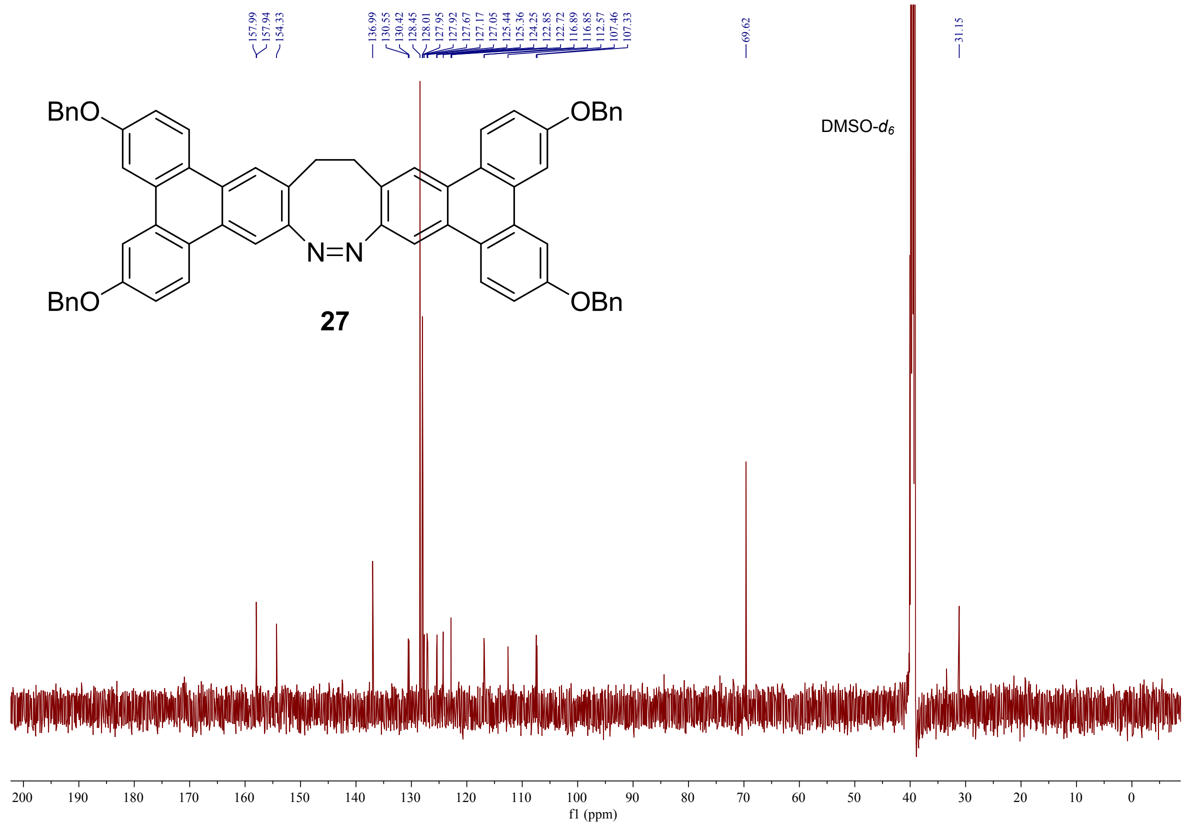

Figure S48: ^{13}C NMR spectrum (151 MHz, $CDCl_3$, 298 K) of compound **27**.

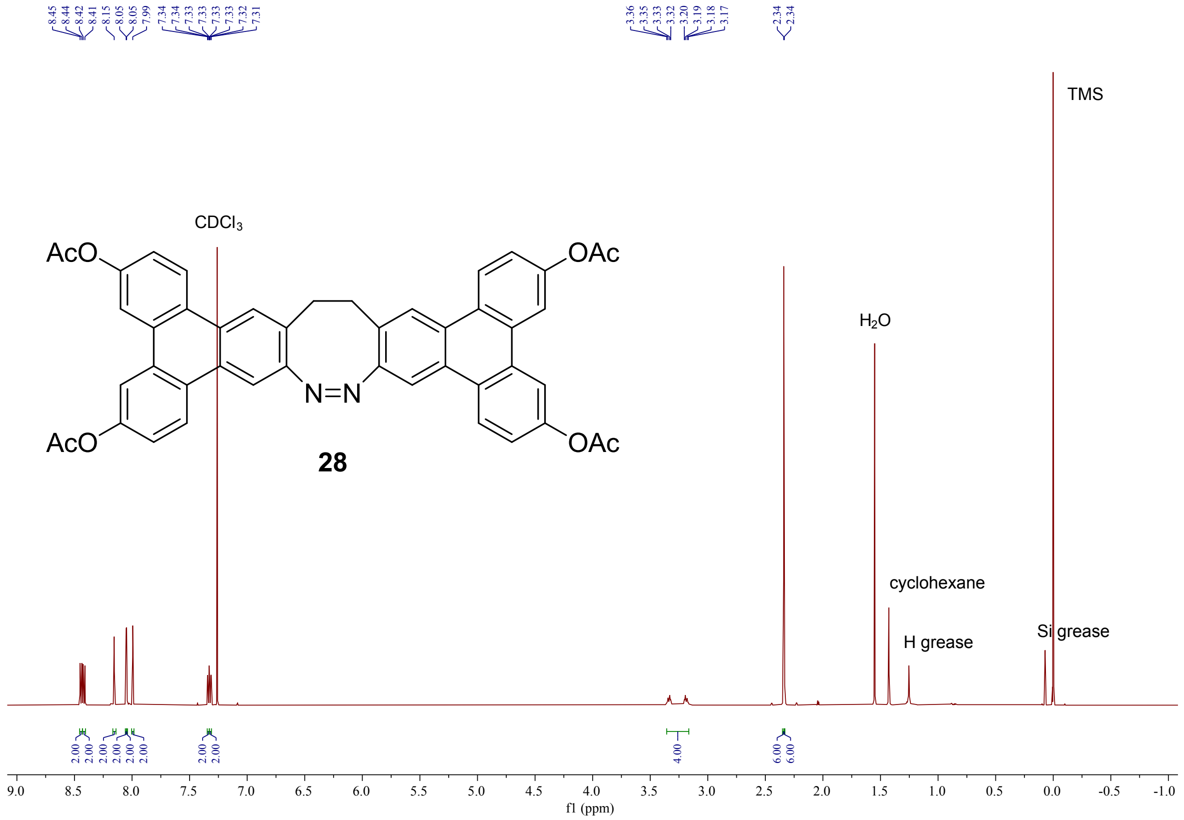

Figure S49: ^{1}H NMR spectrum (600 MHz, $CDCl_3$, 298 K) of compound **28**. After permanent drying *in vacuo*, additional cyclohexane could not be removed.

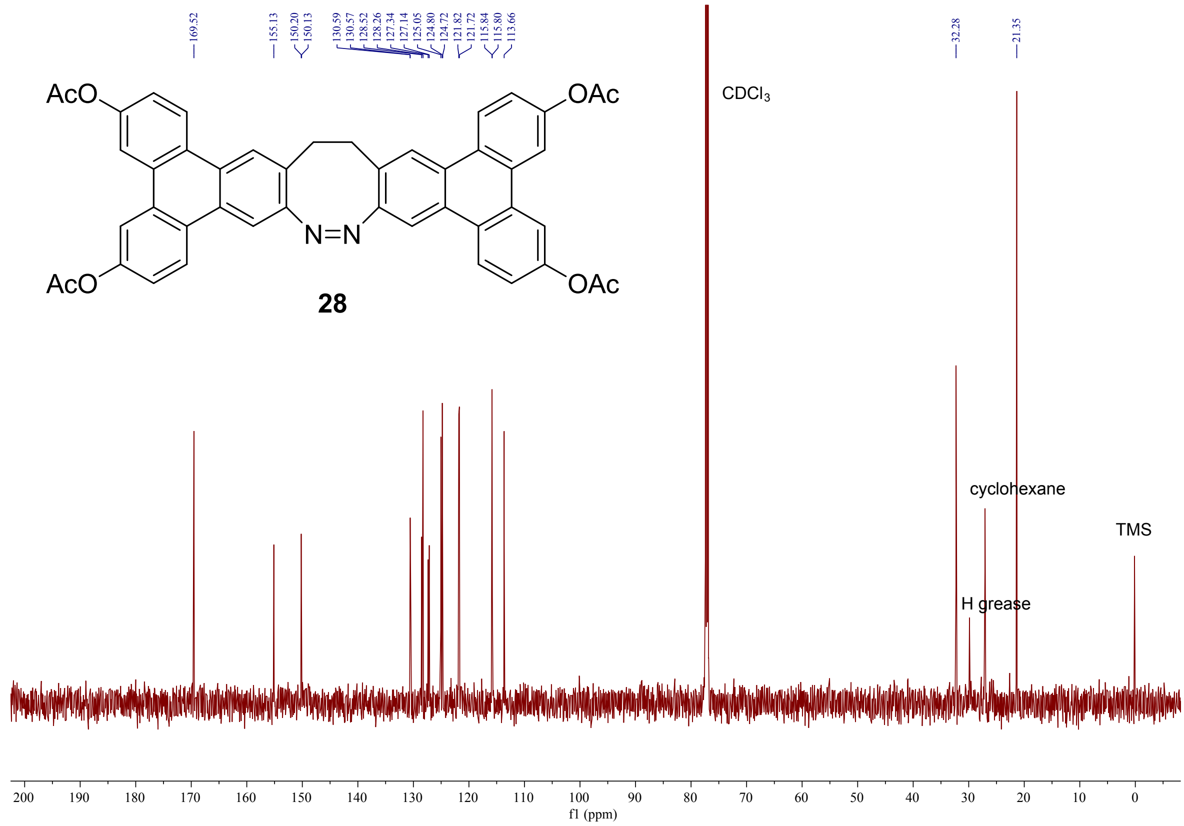

Figure S50: ^{13}C NMR spectrum (126 MHz, $CDCl_3$, 298 K) of compound **28**.

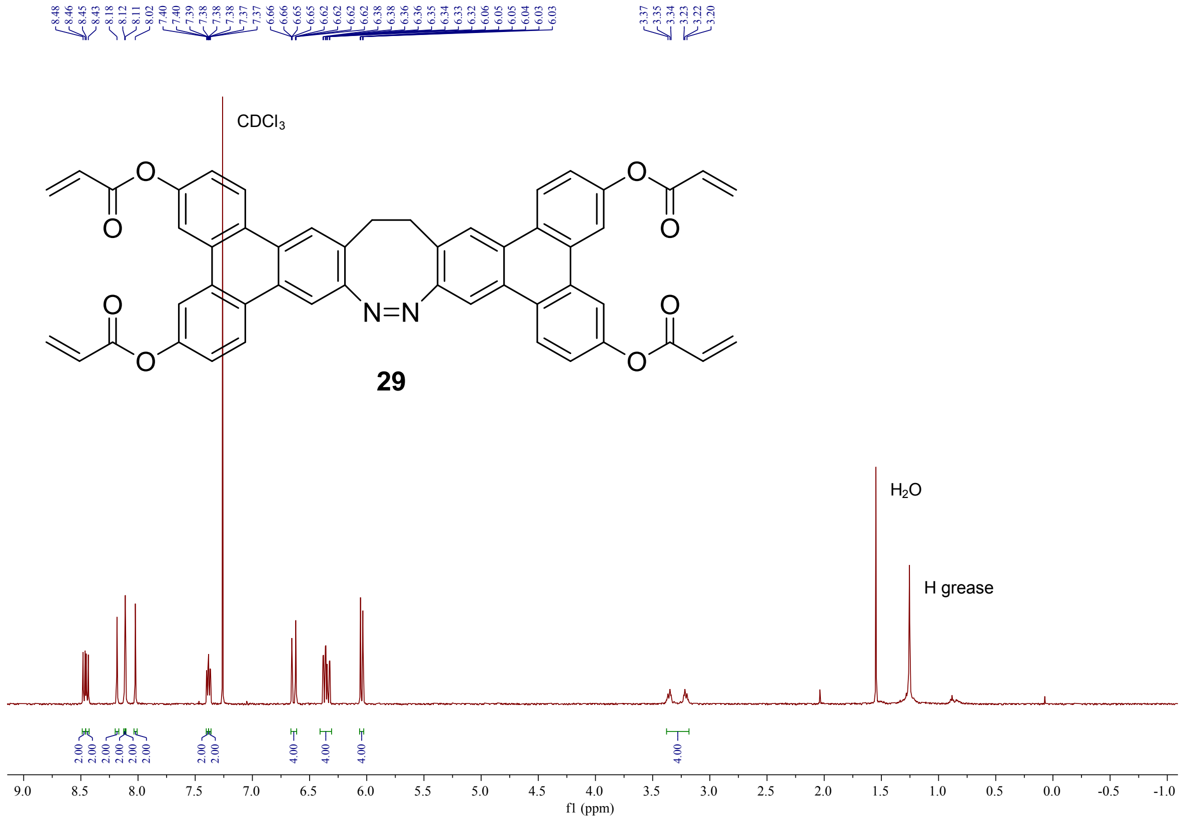

Figure S51: ^{1}H NMR spectrum (500 MHz, $CDCl_3$, 298 K) of compound **29**.

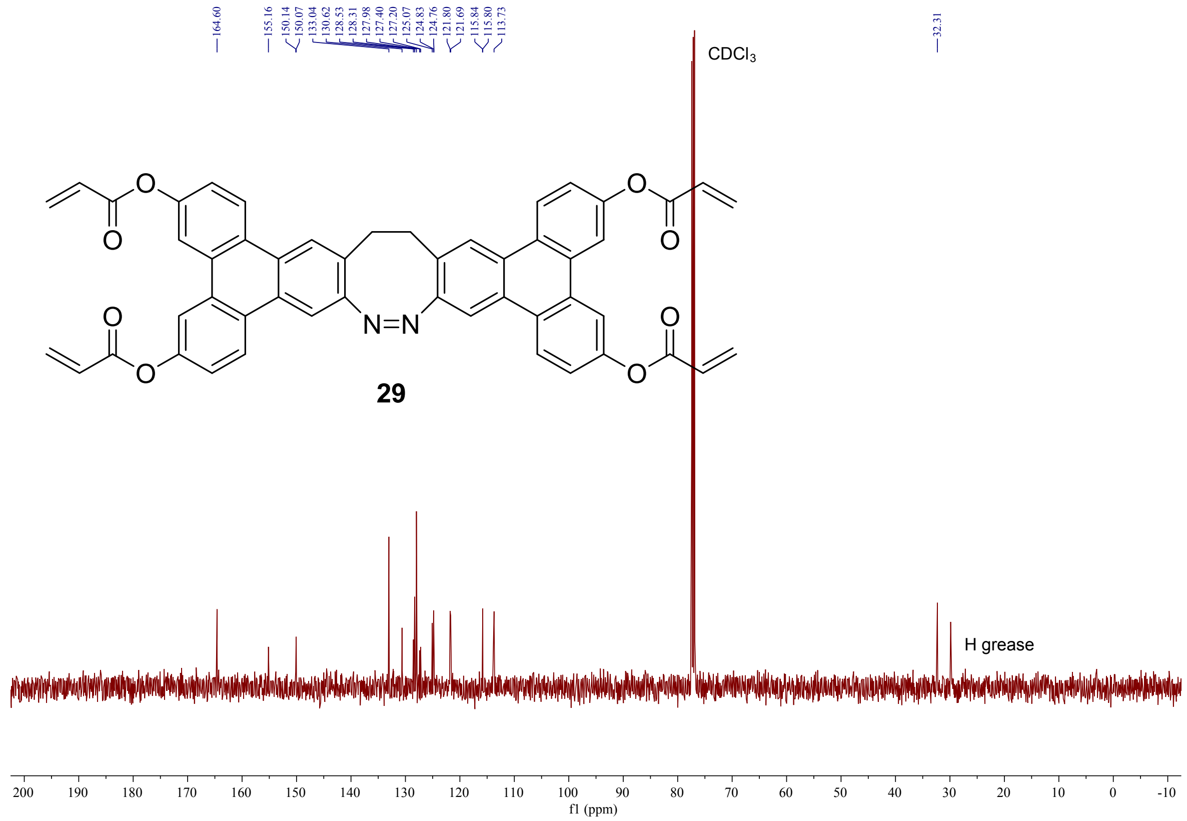

Figure S52: ^{13}C NMR spectrum (126 MHz, $CDCl_3$, 298 K) of compound **29**.

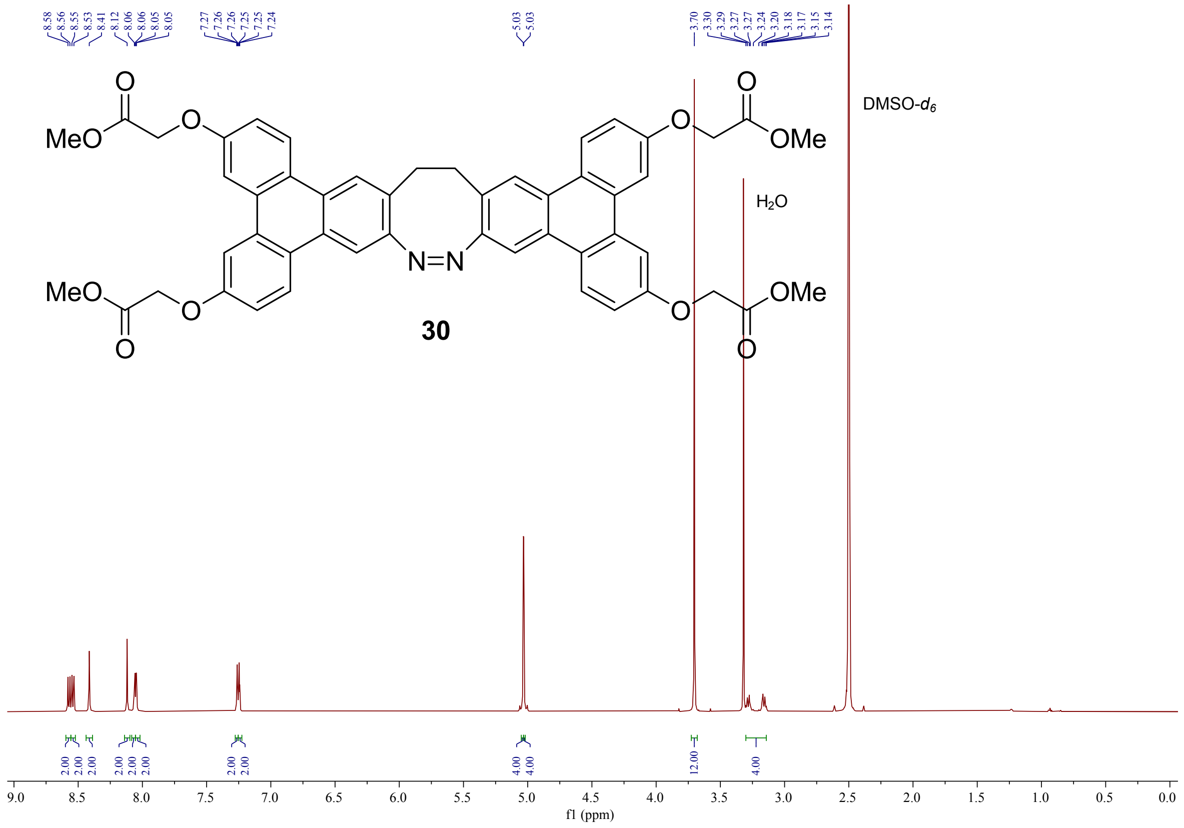

Figure S53: ^1H NMR spectrum (600 MHz, DMSO-d_6, 298 K) of compound **30**.

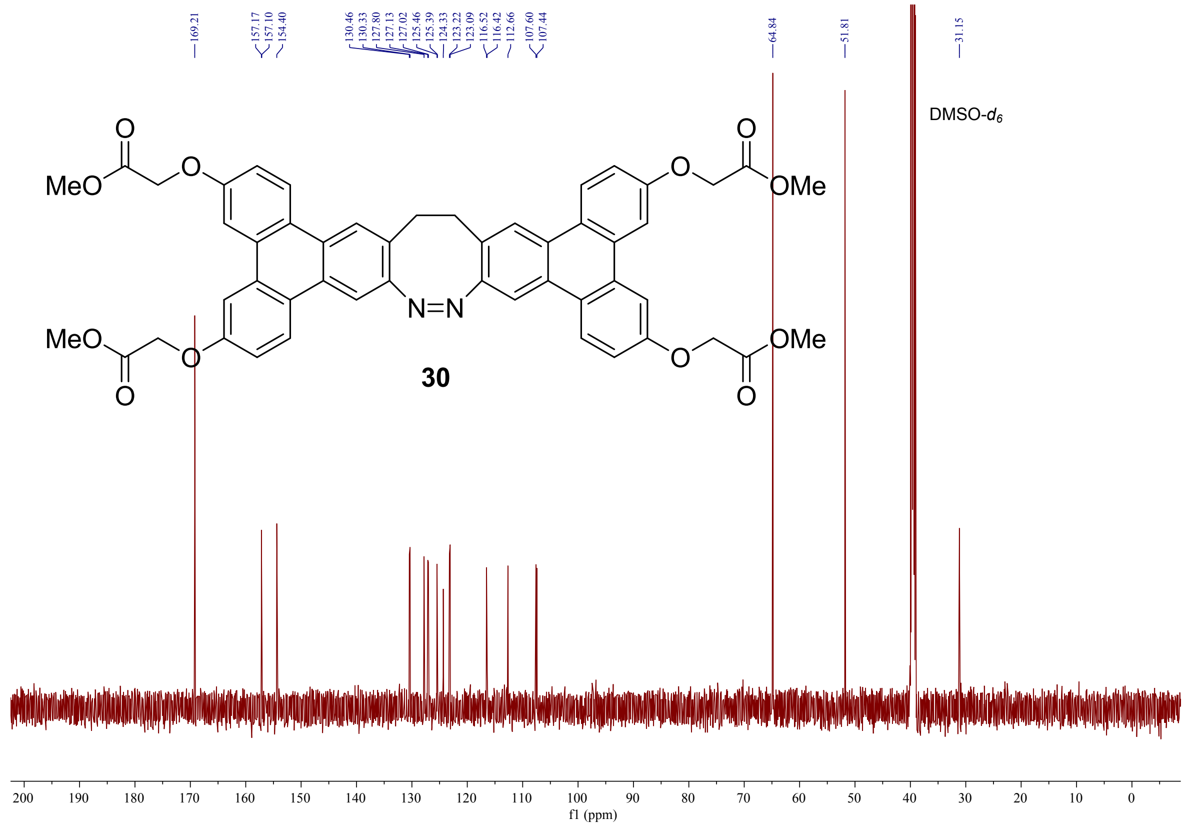

Figure S54: ^{13}C NMR spectrum (151 MHz, DMSO-d_6, 298 K) of compound **30**.

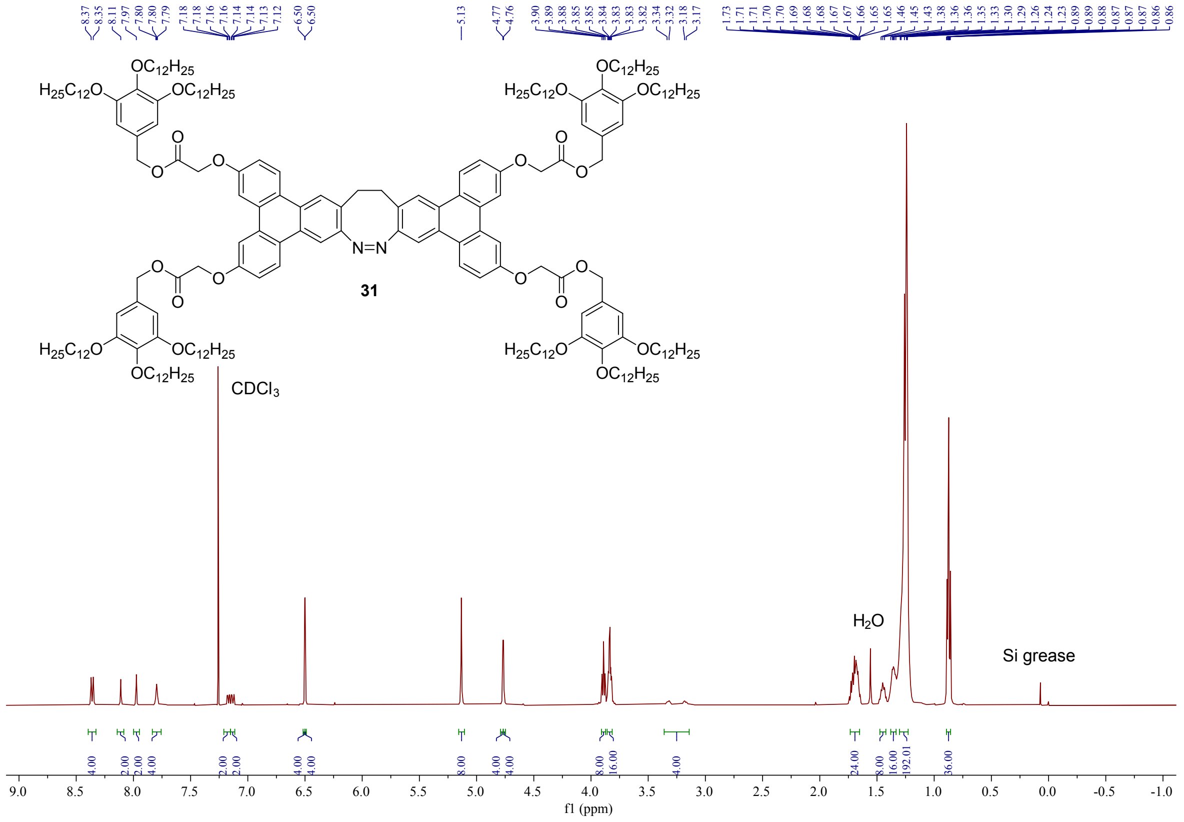

Figure S55: ^{1}H NMR spectrum (500 MHz, CDCl3, 298 K) of compound **31**.

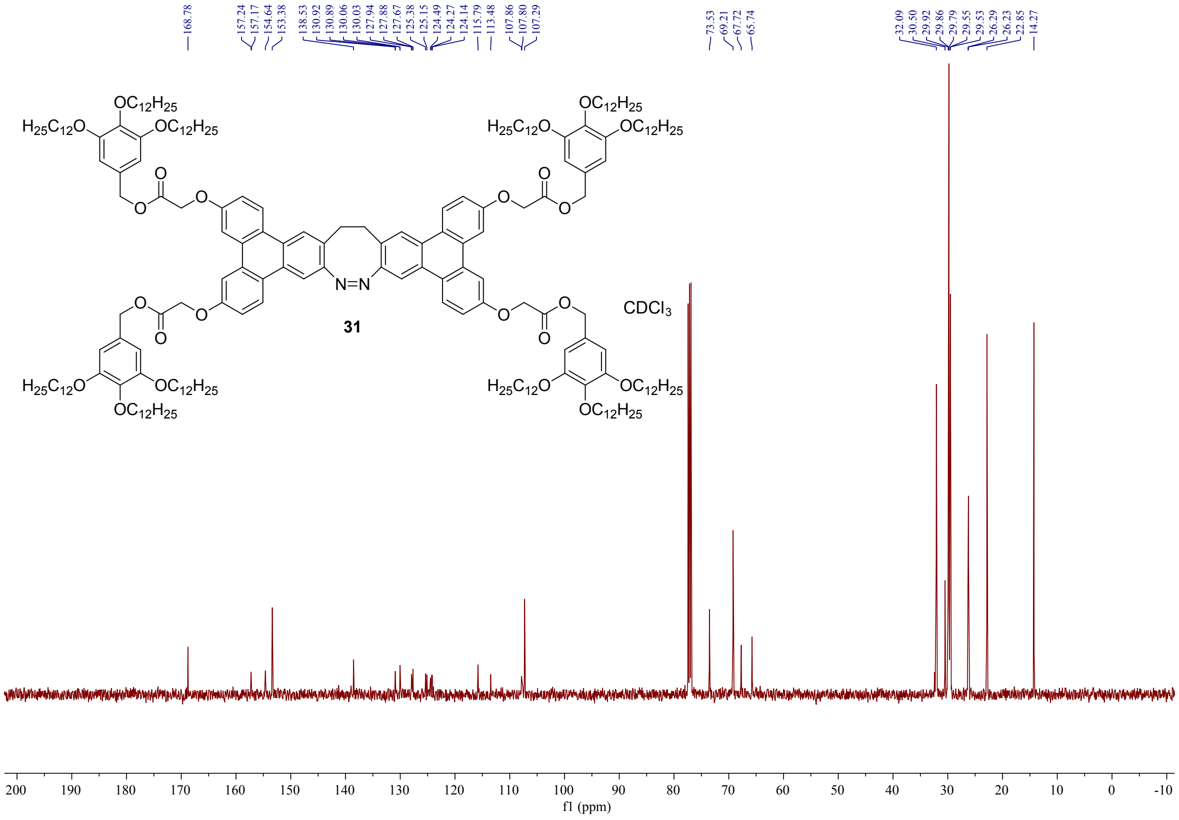

Figure S56: ^{13}C NMR spectrum (126 MHz, $CDCl_3$, 298 K) of compound **31**.

S 4 Photophysical characterization

S 4.1 UV/vis absorption spectra and thermal half-lives

To determine the optimum wavelengths for excitation of the photostationary state, 300 µM solutions in THF (compounds **5**, **18** – **20**, **23** – **26**, **28**, **29** and **31**), in DMSO (compounds **27** and **30**) and 50 µM solutions in DMSO (compounds **21** and **22**) were irradiated with 365 nm, 385 nm, 405 nm, 420 nm, 435 nm and 450 nm for 30 s at 298 K respectively. Re-isomerization to the thermodynamically stable *Z* isomer was carried out by irradiation with 530 nm for 30 s at 298 K. The UV/vis spectra after each irradiation were measured at 298 K within a measuring range of 250 nm – 700 nm. The compounds **21** and **22** are only soluble in DMSO at 298 K in very low concentrations, and only after the solutions have first been heated to almost the boiling point of DMSO. Although the compounds **21** and **22** dissolve in slightly higher concentrations in very hot DMSO, they precipitate as solids at 298 K within a very short time. Therefore, UV/vis spectroscopy had to be carried out at much lower concentrations of the solutions.

The half-lives $t_{1/2}$ of the thermal re-isomerization of the photoswitches **5** and **18** – **31** were determined by plotting $\ln(A_t/A_0)$ vs. time in assumption of first-order reactions. The slope of the linear fit gives the time constant *k*, which is used for the calculation of the half-life $t_{1/2}$ according to equation (1):

$$t_{1/2} = \frac{\ln(2)}{k} \tag{1}$$

A total of 60 data sets at intervals of 3 min at 298 K after excitation of the photostationary state for 30 s at 298 K with the previously determined optimum wavelengths were measured for each compound **5** and **18** – **31**. False excitation of the photostationary state in the measuring range of 250 nm – 750 nm by the light source of the UV/vis spectrometer used could not be ruled out. For this reason, only a small range of ± 5 nm around the respective λ_{max} of each photoswitch was recorded within each measurement. Consequently, 60 absorption data points at λ_{max} of each compound were used to determine the half-life $t_{1/2}$.

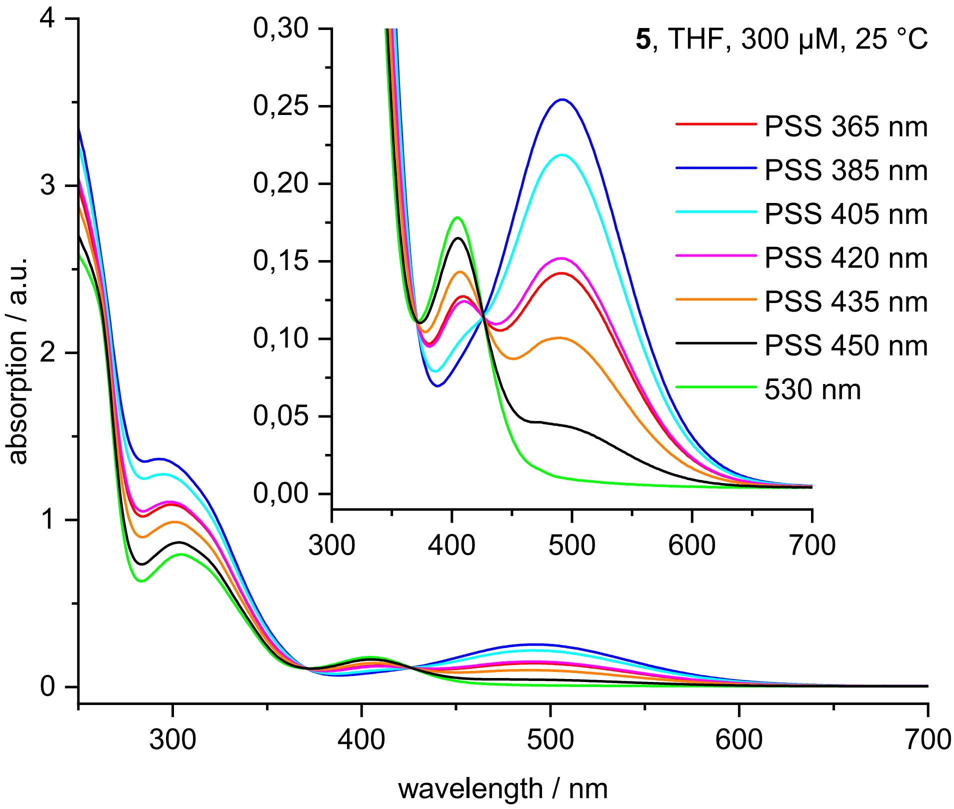

Figure S57: UV/vis spectra (THF, 300 µM, 298 K) of compound **5** after irradiation with 365 – 530 nm (λ_{max} (Z) = 405 nm, λ_{max} (E) = 492 nm).

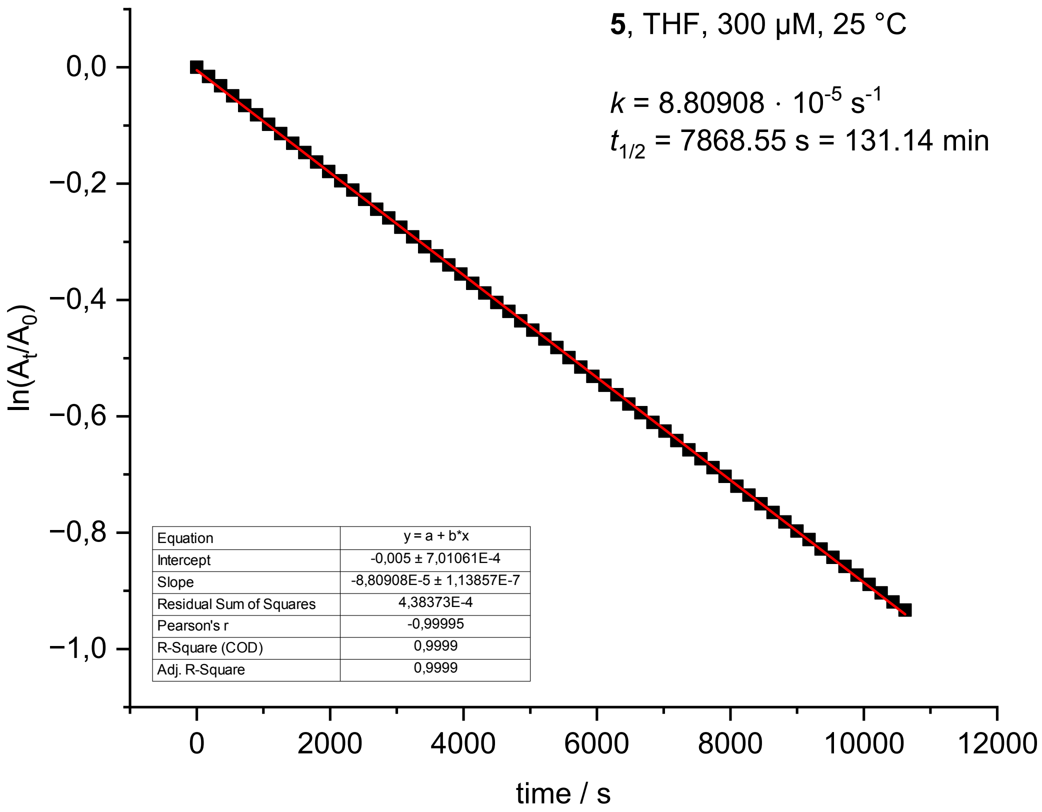

Figure S58: Determination of the thermal half-life $t_{1/2}$ of compound **5** (THF, 300 µM, 298 K) after irradiation with 385 nm (k = 8.80908 · 10^{-5} s^{-1}, $t_{1/2}$ = 131.14 min).

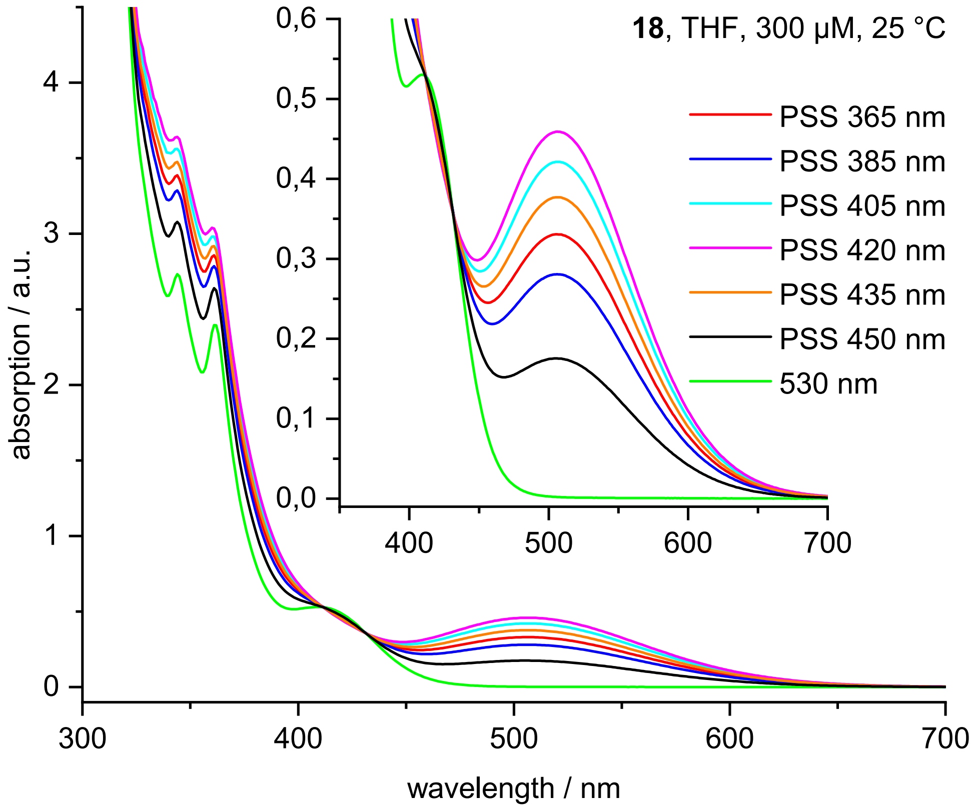

Figure S59: UV/vis spectra (THF, 300 µM, 298 K) of compound **18** after irradiation with 365 – 530 nm (λ_{max} (Z) = 410 nm, λ_{max} (E) = 507 nm).

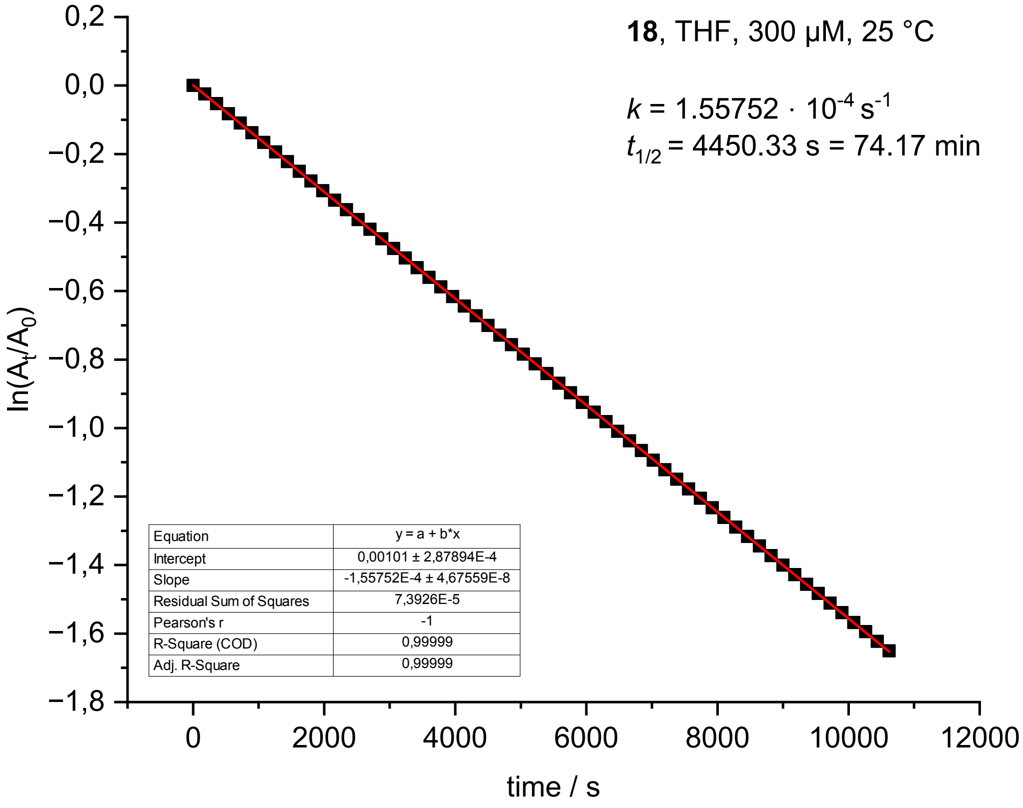

Figure S60: Determination of the thermal half-life $t_{1/2}$ of compound **18** (THF, 300 µM, 298 K) after irradiation with 420 nm (k = 1.55752 · 10^{-4} s^{-1}, $t_{1/2}$ = 74.17 min).

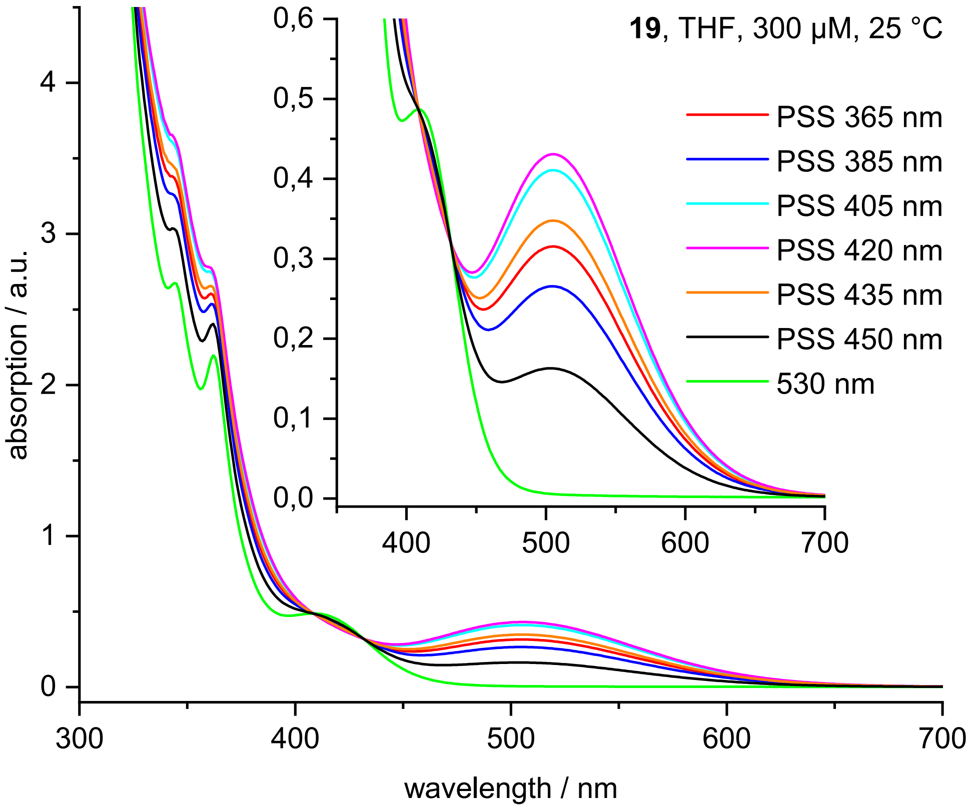

Figure S61: UV/vis spectra (THF, 300 μM, 298 K) of compound **19** after irradiation with 365 – 530 nm (λ_{max} (*Z*) = 409 nm, λ_{max} (*E*) = 505 nm).

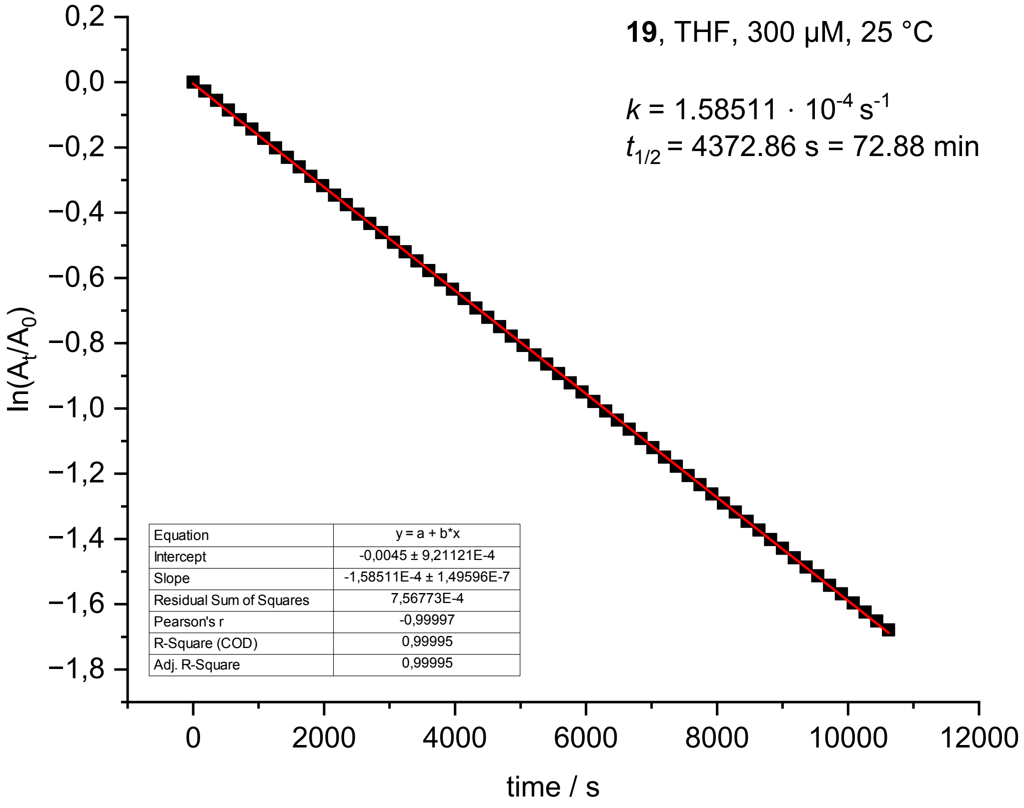

Figure S62: Determination of the thermal half-life $t_{1/2}$ of compound **19** (THF, 300 μM, 298 K) after irradiation with 420 nm (k = 1.58511 · 10^{-4} s^{-1}, $t_{1/2}$ = 72.88 min).

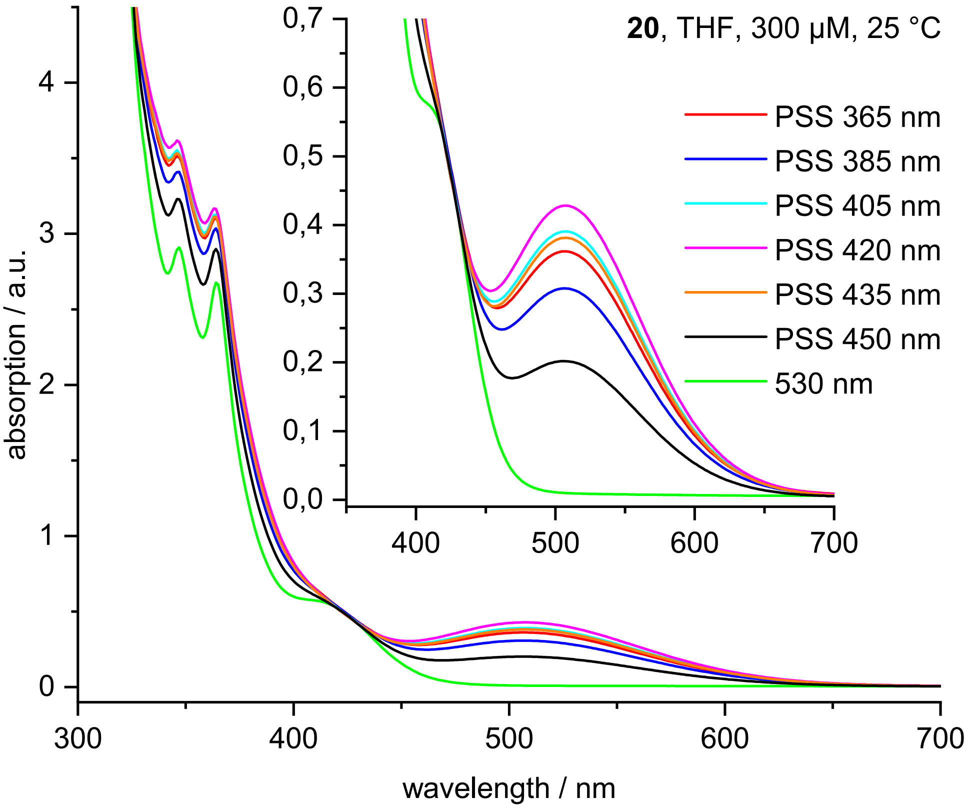

Figure S63: UV/vis spectra (THF, 300 µM, 298 K) of compound **20** after irradiation with 365 – 530 nm (λ_{max} (Z) = 410 nm, λ_{max} (E) = 507 nm).

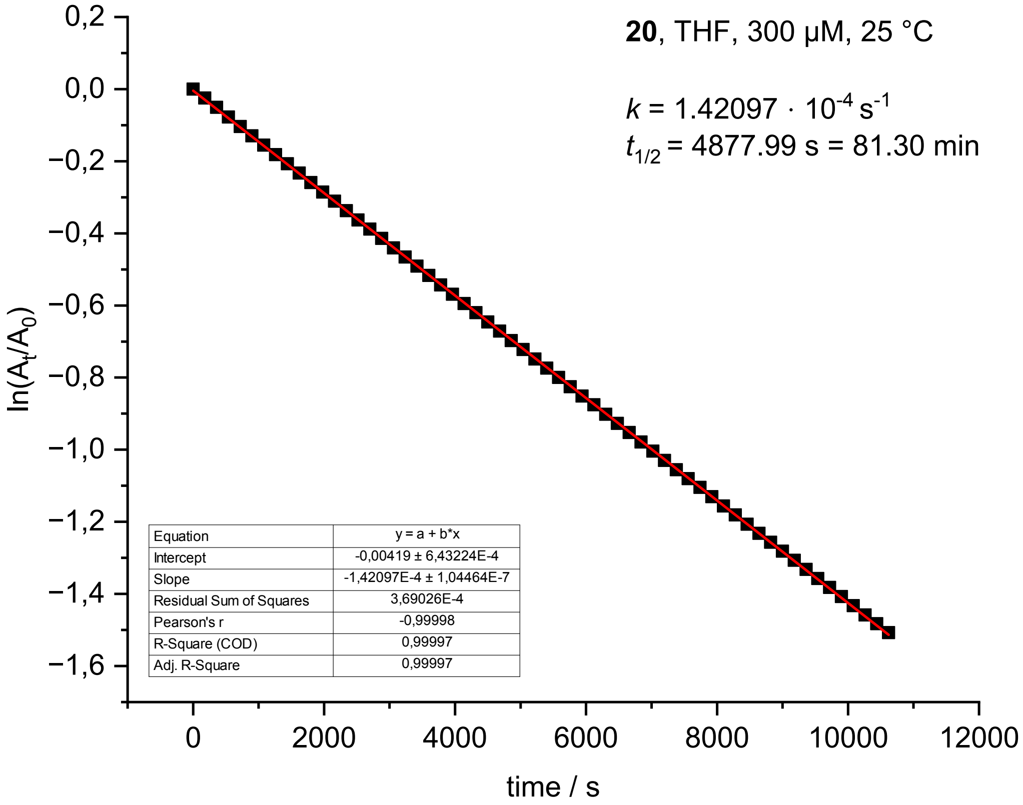

Figure S64: Determination of the thermal half-life $t_{1/2}$ of compound **20** (THF, 300 µM, 298 K) after irradiation with 420 nm (k = 1.42097 · 10^{-4} s^{-1}, $t_{1/2}$ = 81.30 min).

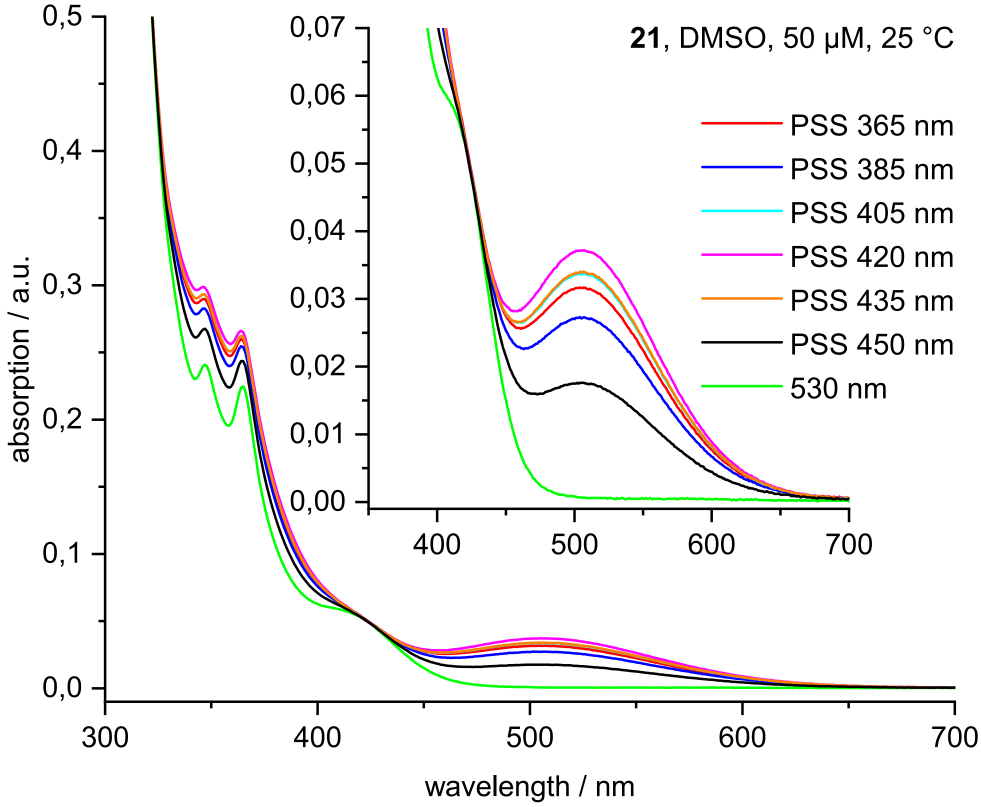

Figure S65: UV/vis spectra (DMSO, 50 µM, 298 K) of compound **21** after irradiation with 365 – 530 nm (λ_{max} (Z) = 410 nm, λ_{max} (E) = 505 nm).

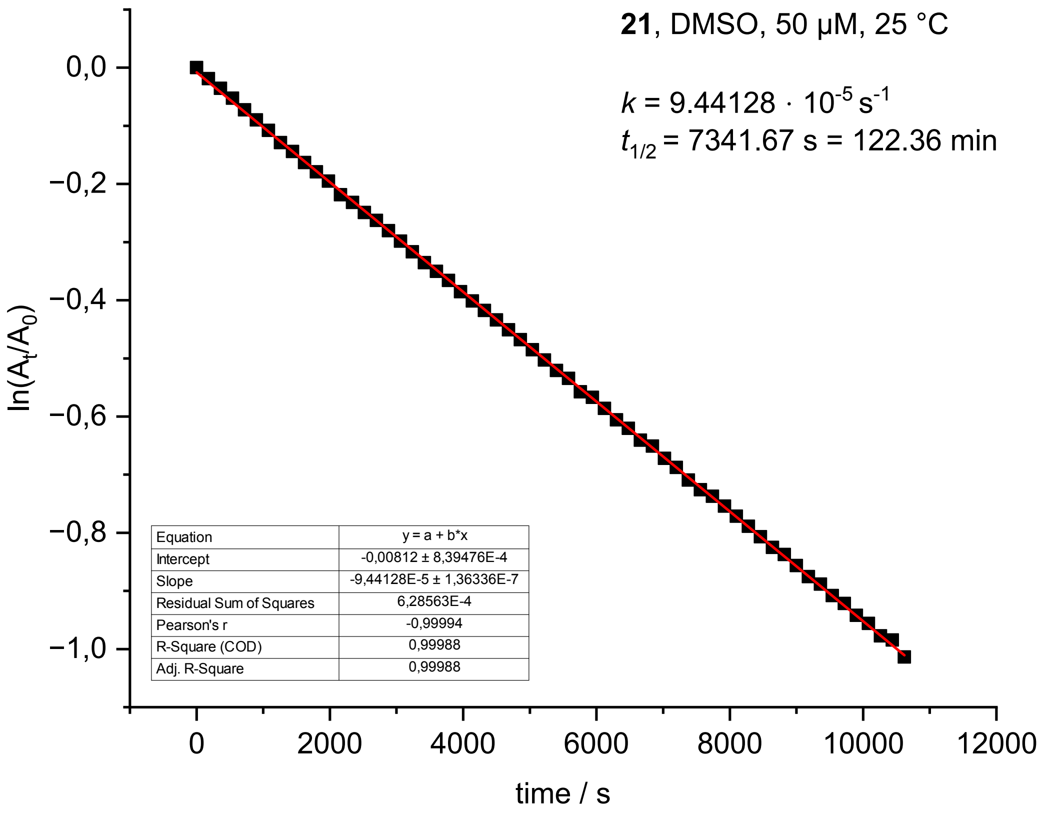

Figure S66: Determination of the thermal half-life $t_{1/2}$ of compound **21** (DMSO, 50 µM, 298 K) after irradiation with 420 nm (k = 9.44128 · 10^{-5} s^{-1}, $t_{1/2}$ = 122.36 min).

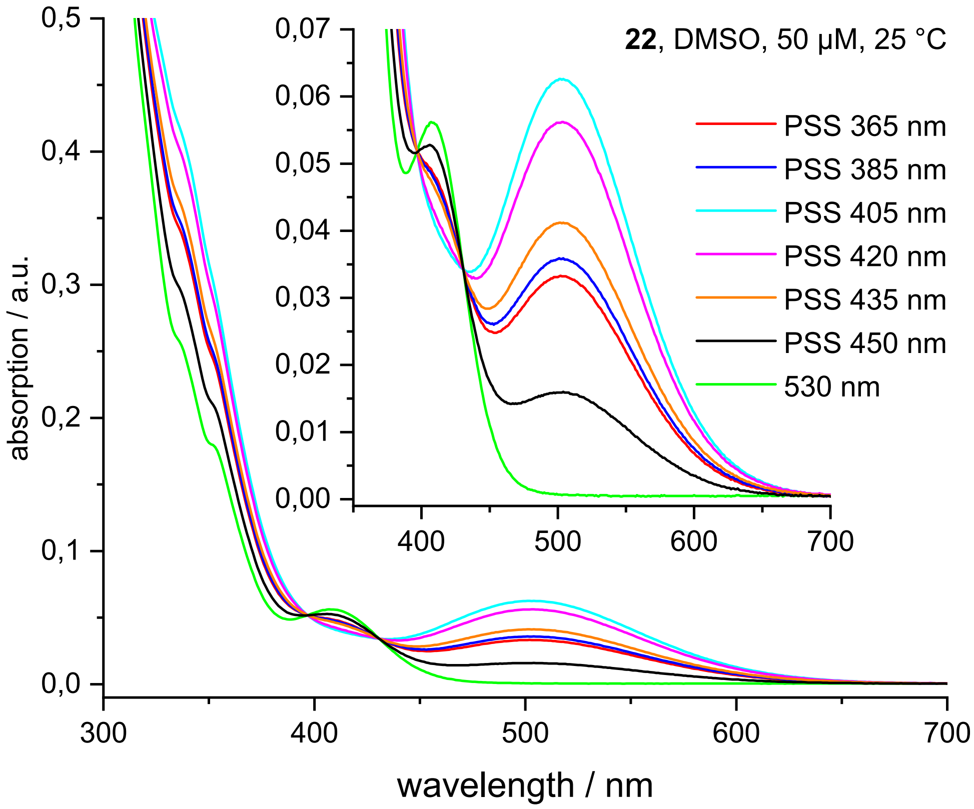

Figure S67: UV/vis spectra (DMSO, 50 µM, 298 K) of compound **22** after irradiation with 365 – 530 nm (λ_{max} (Z) = 405 nm, λ_{max} (E) = 504 nm).

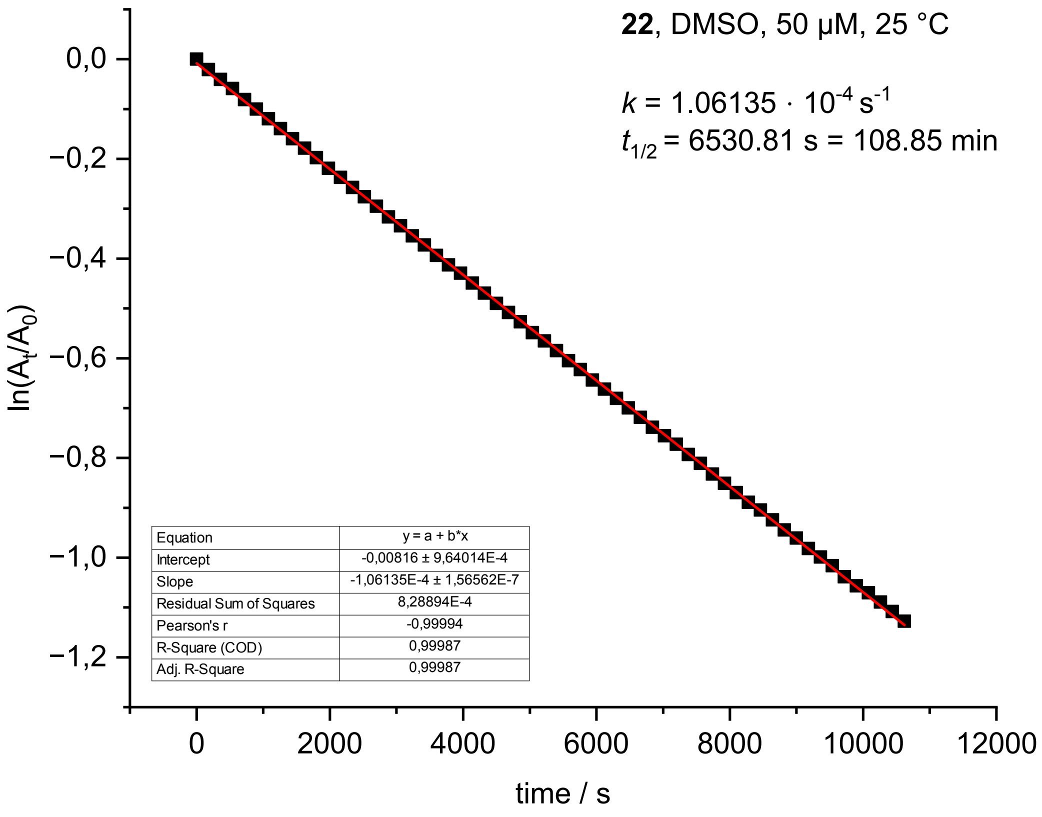

Figure S68: Determination of the thermal half-life $t_{1/2}$ of compound **22** (DMSO, 50 µM, 298 K) after irradiation with 405 nm (k = 1.06135 · 10^{-4} s^{-1}, $t_{1/2}$ = 108.85 min).

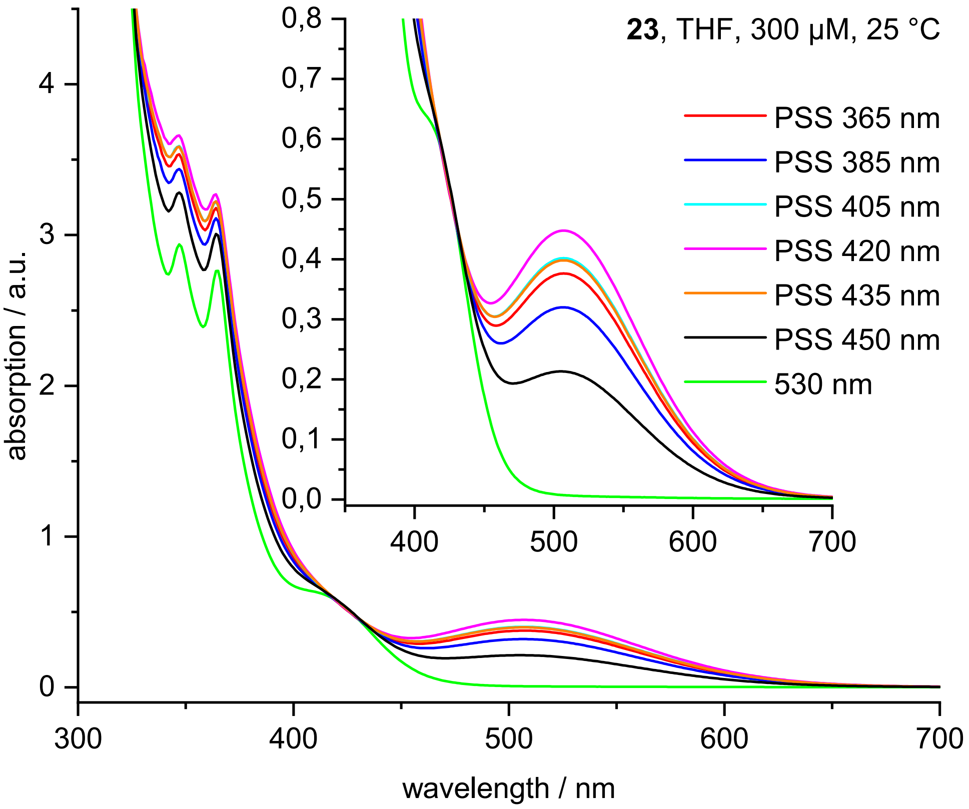

Figure S69: UV/vis spectra (THF, 300 μM, 298 K) of compound **23** after irradiation with 365 – 530 nm (λ_{max} (Z) = 410 nm, λ_{max} (E) = 507 nm).

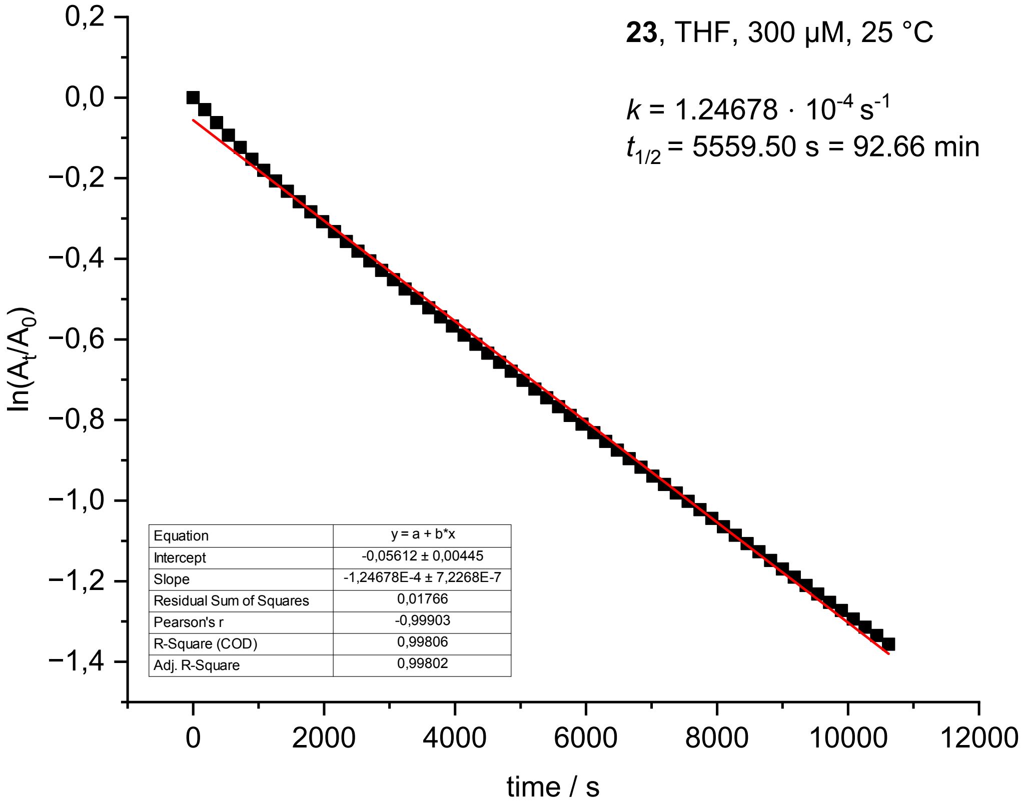

Figure S70: Determination of the thermal half-life $t_{1/2}$ of compound **23** (THF, 300 μM, 298 K) after irradiation with 420 nm (k = 1.24678 · 10^{-4} s^{-1}, $t_{1/2}$ = 92.66 min).

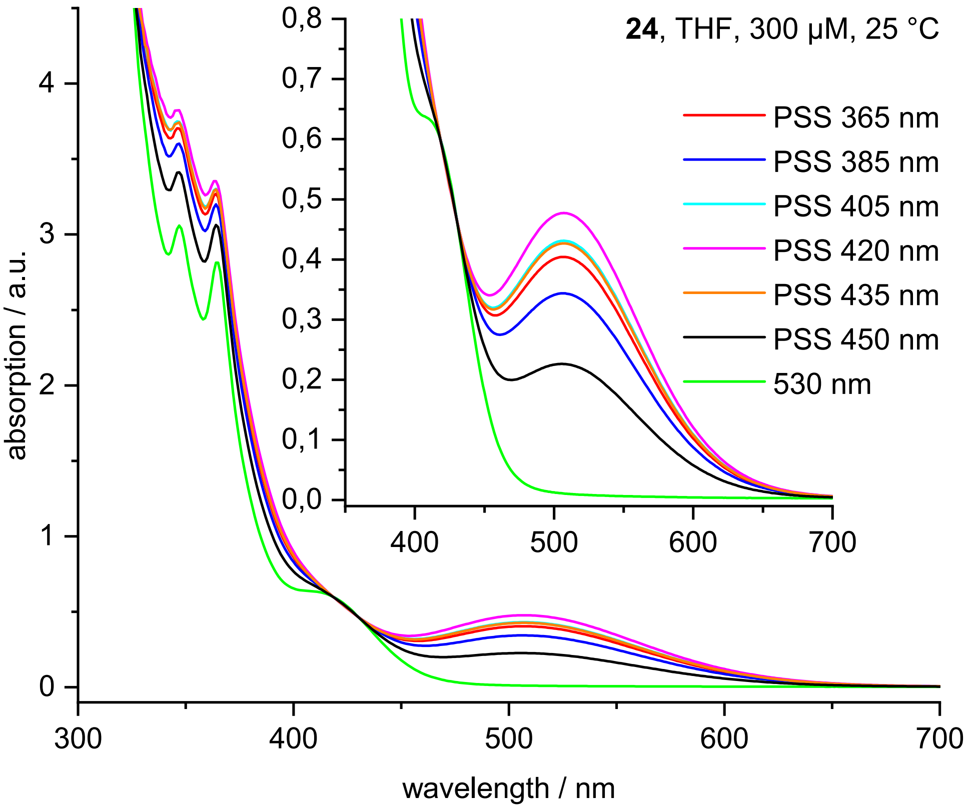

Figure S71: UV/vis spectra (THF, 300 µM, 298 K) of compound **24** after irradiation with 365 – 530 nm (λ_{max} (Z) = 410 nm, λ_{max} (E) = 507 nm).

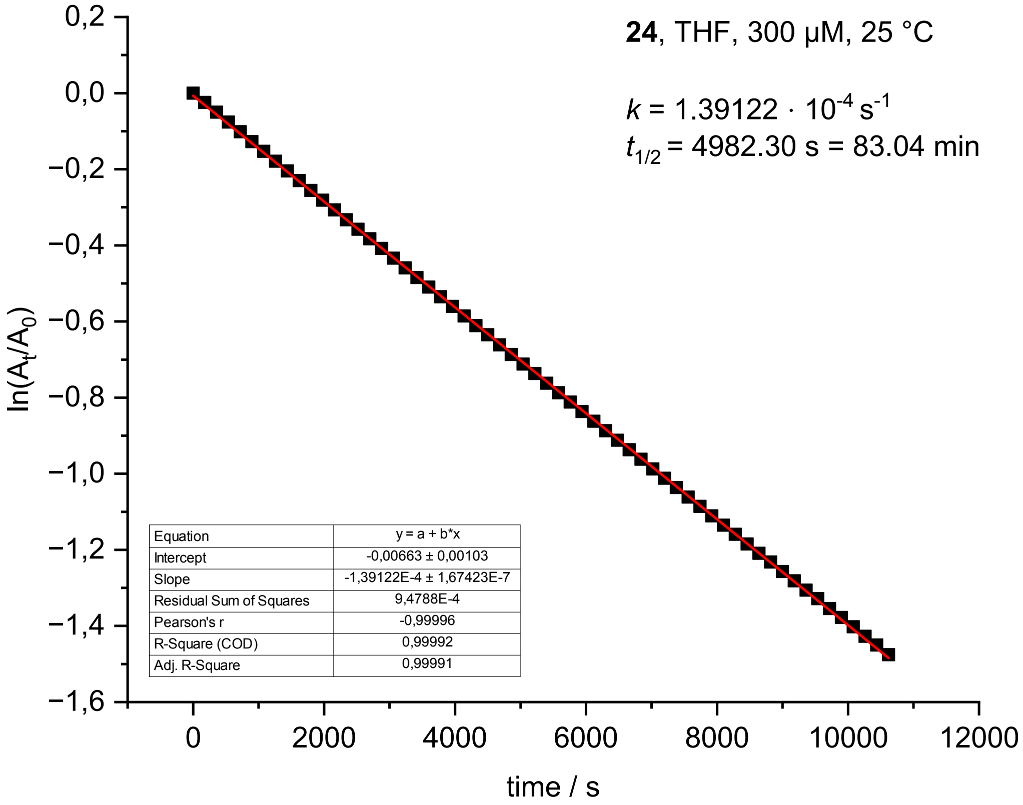

Figure S72: Determination of the thermal half-life $t_{1/2}$ of compound **24** (THF, 300 µM, 298 K) after irradiation with 420 nm (k = 1.39122 · 10^{-4} s^{-1}, $t_{1/2}$ = 83.04 min).

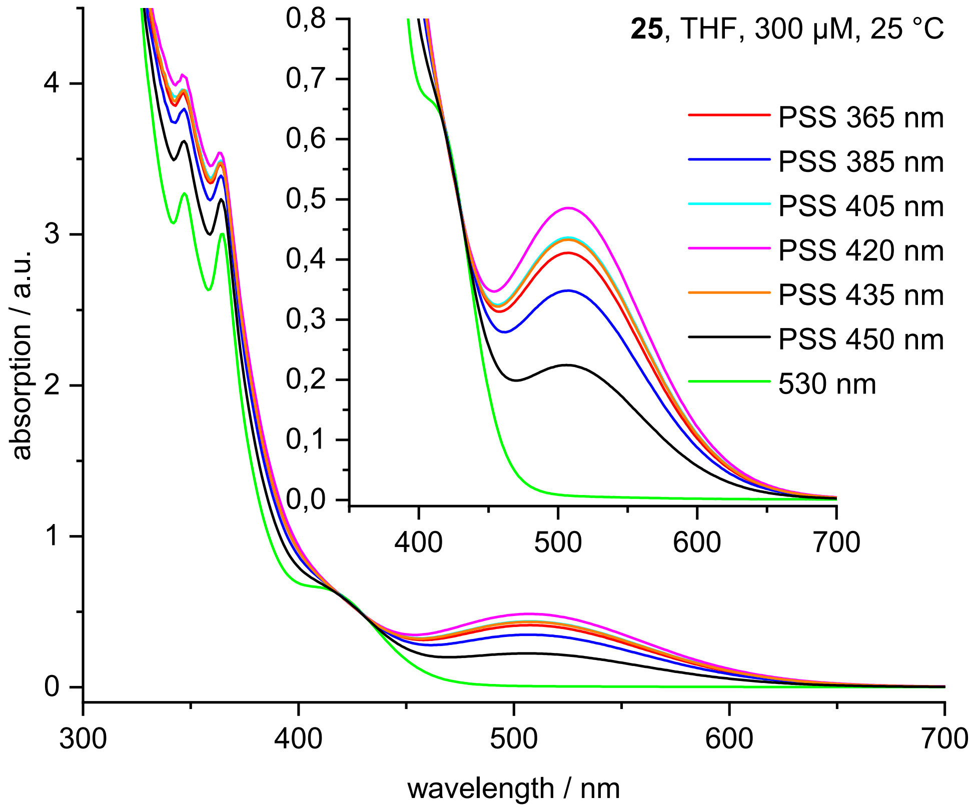

Figure S73: UV/vis spectra (THF, 300 µM, 298 K) of compound **25** after irradiation with 365 – 530 nm (λ_{max} (Z) = 410 nm, λ_{max} (E) = 507 nm).

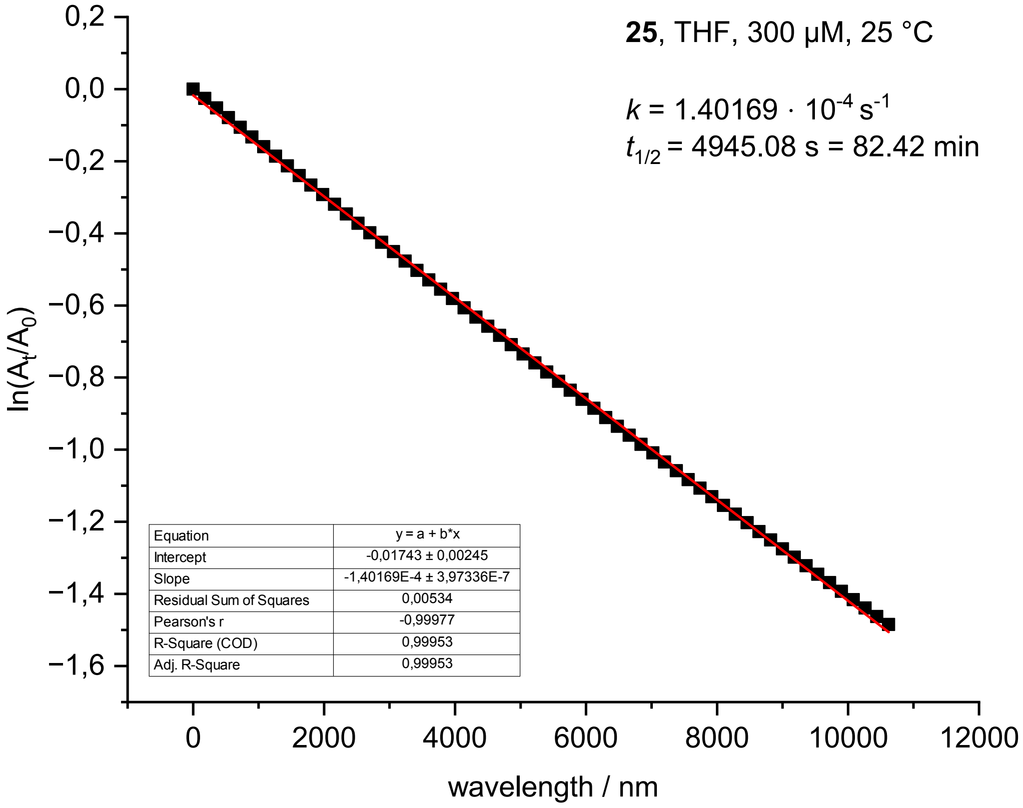

Figure S74: Determination of the thermal half-life $t_{1/2}$ of compound **25** (THF, 300 µM, 298 K) after irradiation with 420 nm (k = 1.40169 · 10^{-4} s^{-1}, $t_{1/2}$ = 82.42 min).

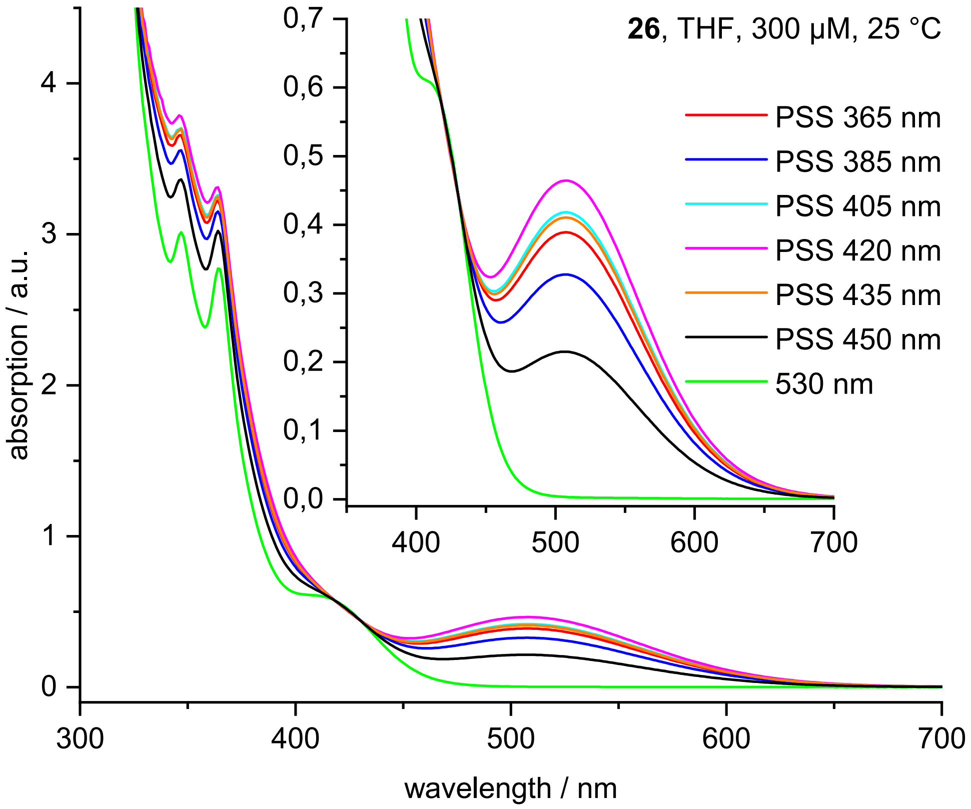

Figure S75: UV/vis spectra (THF, 300 µM, 298 K) of compound **26** after irradiation with 365 – 530 nm (λ_{max} (Z) = 410 nm, λ_{max} (E) = 507 nm).

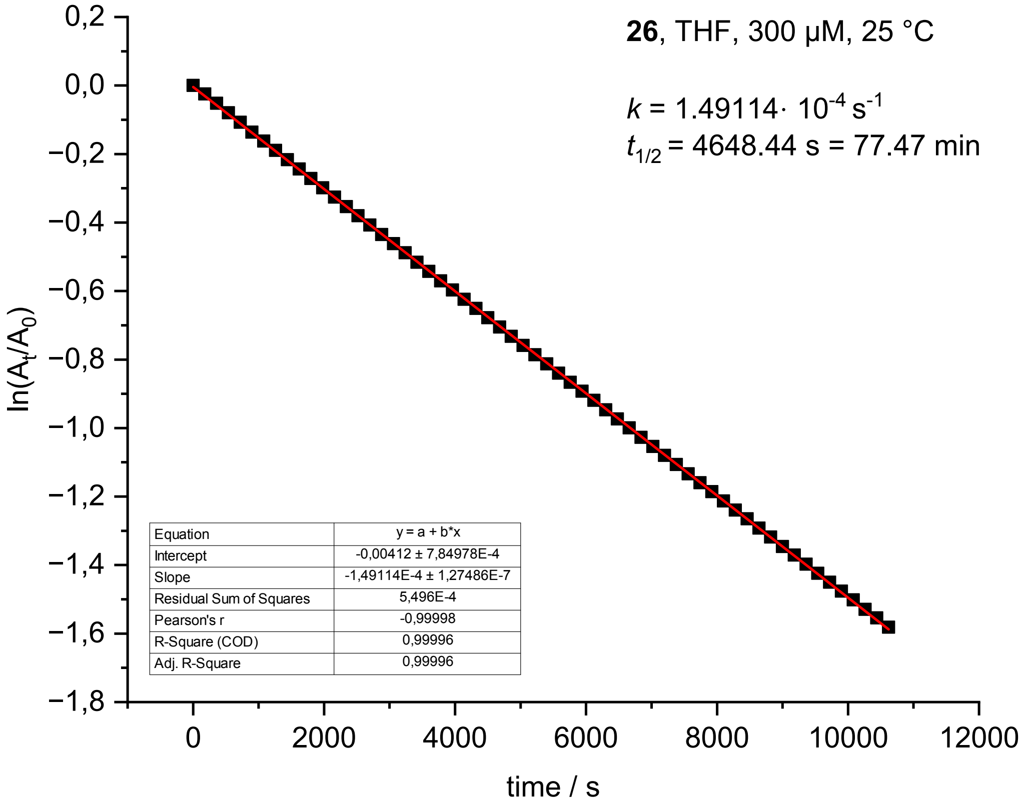

Figure S76: Determination of the thermal half-life $t_{1/2}$ of compound **26** (THF, 300 µM, 298 K) after irradiation with 420 nm (k = 1.49114 · 10^{-4} s^{-1}, $t_{1/2}$ = 77.47 min).

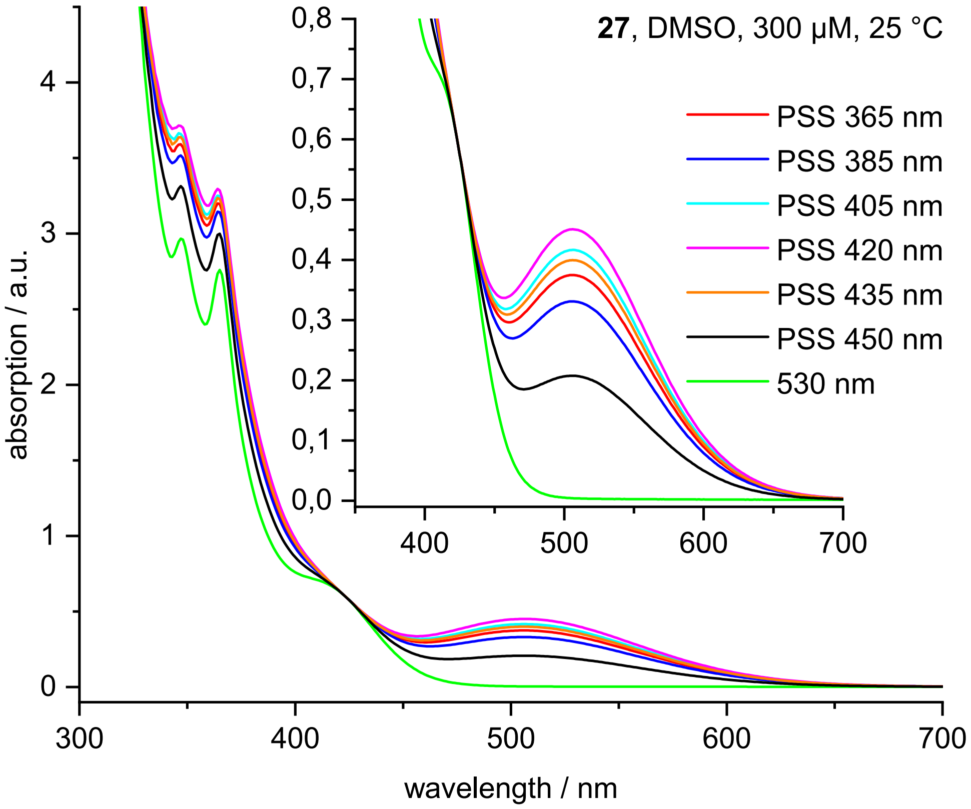

Figure S77: UV/vis spectra (DMSO, 300 µM, 298 K) of compound **27** after irradiation with 365 – 530 nm (λ_{max} (Z) = 410 nm, λ_{max} (E) = 506 nm).

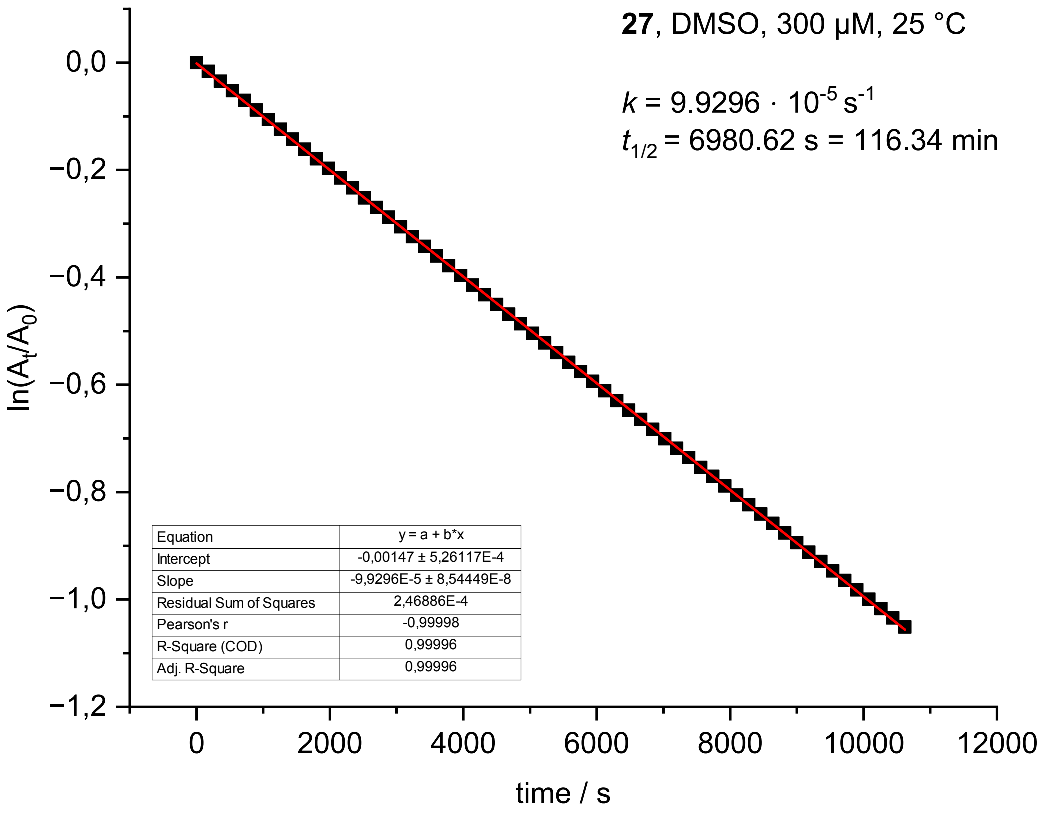

Figure S78: Determination of the thermal half-life $t_{1/2}$ of compound **27** (DMSO, 300 µM, 298 K) after irradiation with 420 nm (k = 9.9296 · 10^{-5} s^{-1}, $t_{1/2}$ = 116.34 min).

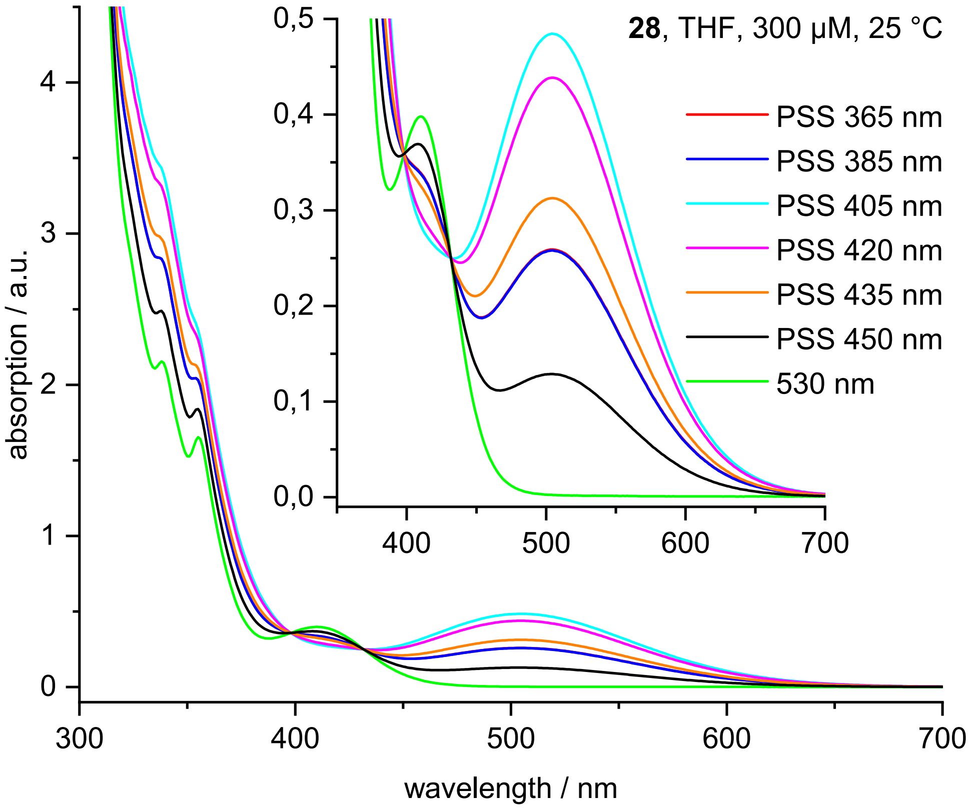

Figure S79: UV/vis spectra (THF, 300 µM, 298 K) of compound **28** after irradiation with 365 – 530 nm (λ_{max} (Z) = 410 nm, λ_{max} (E) = 505 nm).

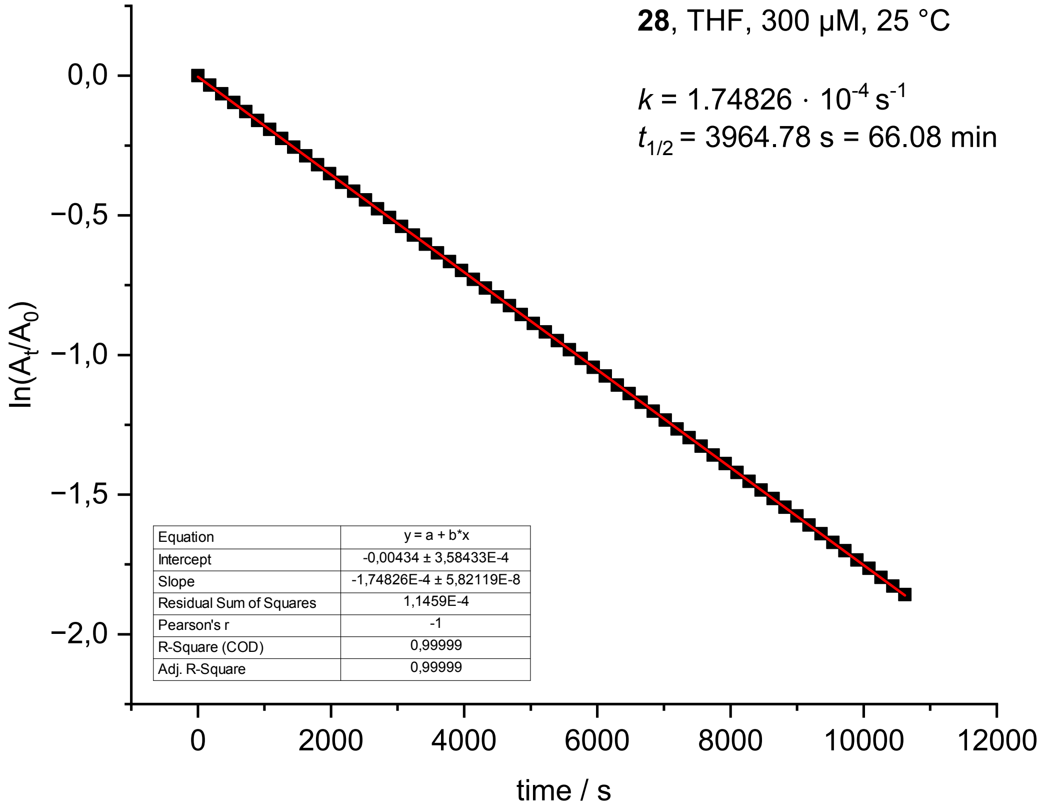

Figure S80: Determination of the thermal half-life $t_{1/2}$ of compound **28** (THF, 300 µM, 298 K) after irradiation with 405 nm (k = 1.74826 · 10^{-4} s^{-1}, $t_{1/2}$ = 66.08 min).

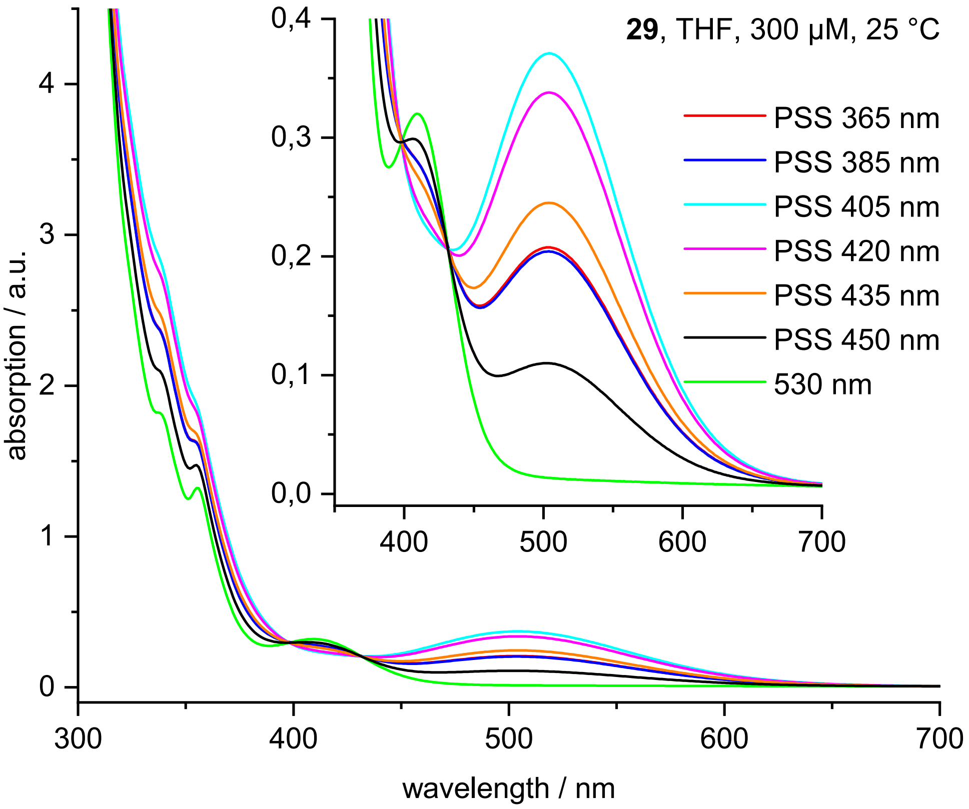

Figure S81: UV/vis spectra (THF, 300 µM, 298 K) of compound **29** after irradiation with 365 – 530 nm (λ_{max} (Z) = 410 nm, λ_{max} (E) = 504 nm).

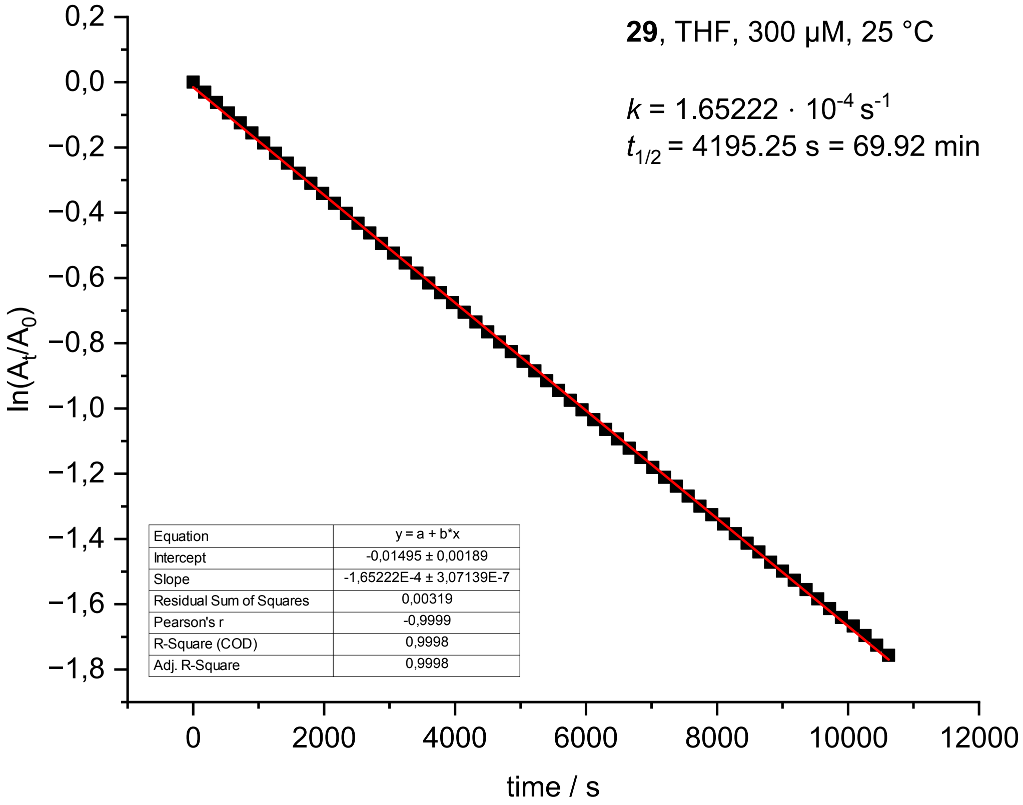

Figure S82: Determination of the thermal half-life $t_{1/2}$ of compound **29** (THF, 300 µM, 298 K) after irradiation with 405 nm (k = 1.65222 · 10^{-4} s^{-1}, $t_{1/2}$ = 69.92 min).

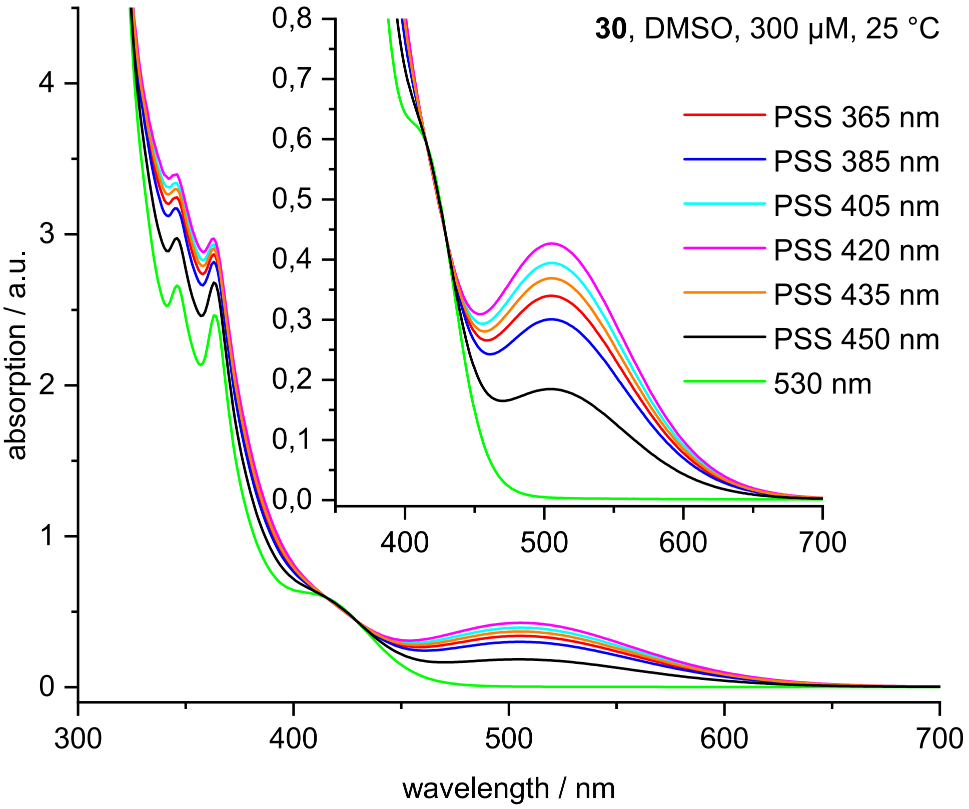

Figure S83: UV/vis spectra (DMSO, 300 µM, 298 K) of compound **30** after irradiation with 365 – 530 nm (λ_{max} (Z) = 410 nm, λ_{max} (E) = 506 nm).

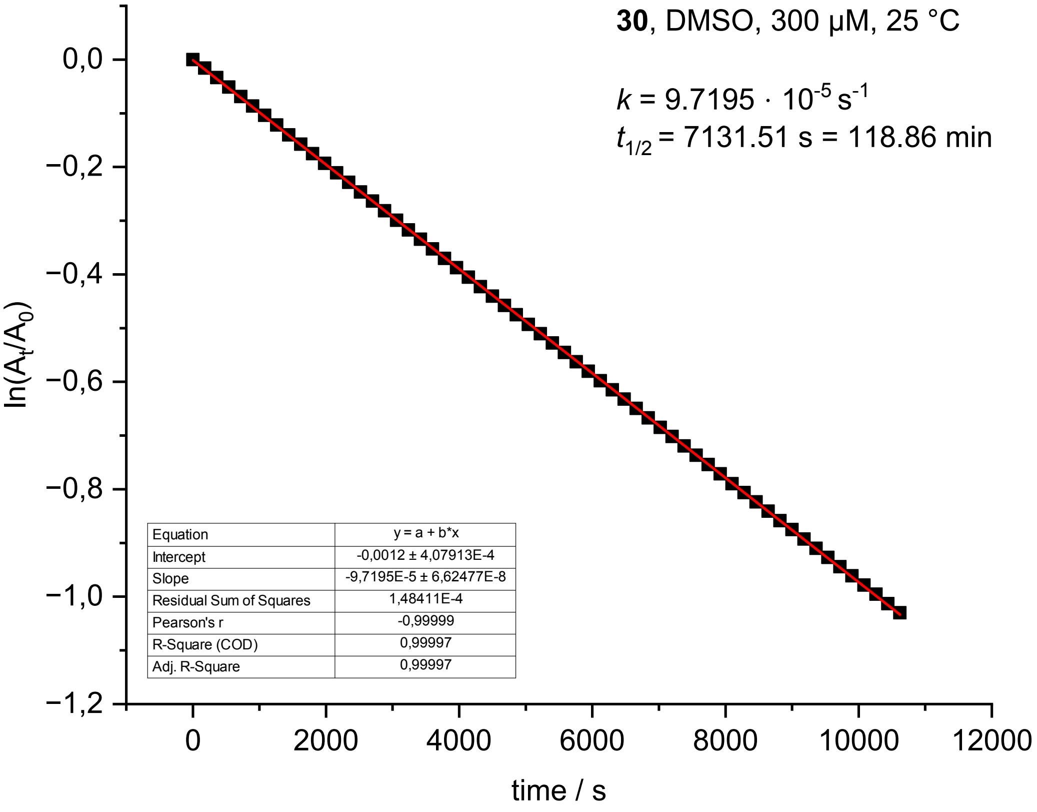

Figure S84: Determination of the thermal half-life $t_{1/2}$ of compound **30** (DMSO, 300 µM, 298 K) after irradiation with 420 nm (k = 9.7195 · 10^{-5} s^{-1}, $t_{1/2}$ = 118.86 min).

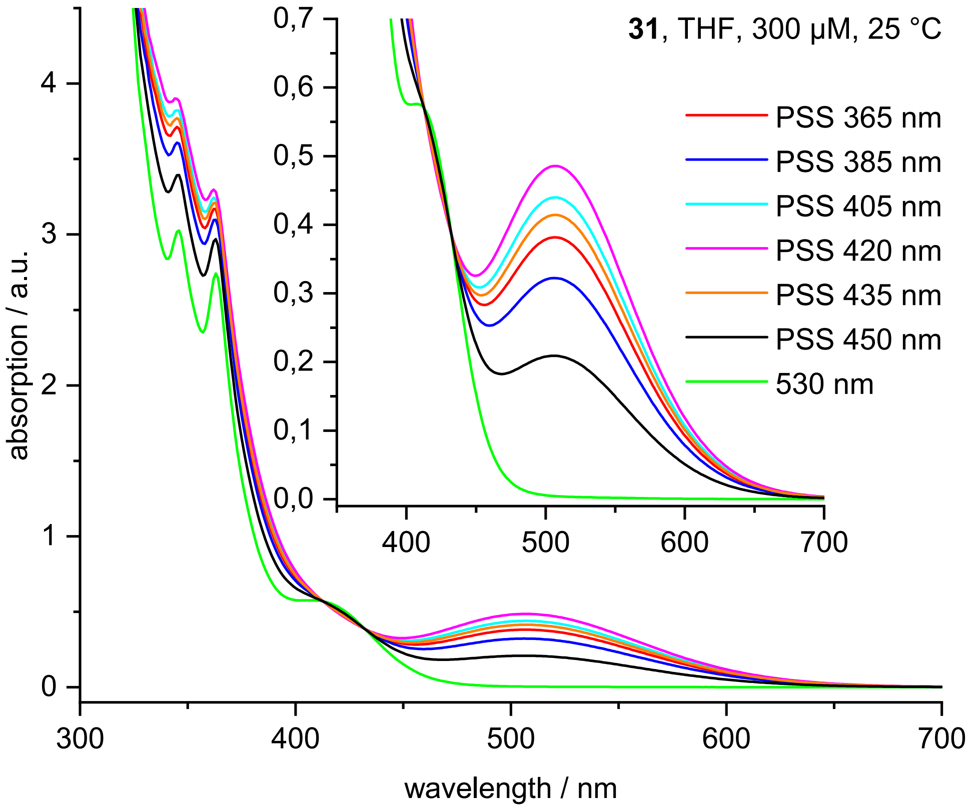

Figure S85: UV/vis spectra (THF, 300 µM, 298 K) of compound **31** after irradiation with 365 – 530 nm (λ_{max} (Z) = 407 nm, λ_{max} (E) = 504 nm).

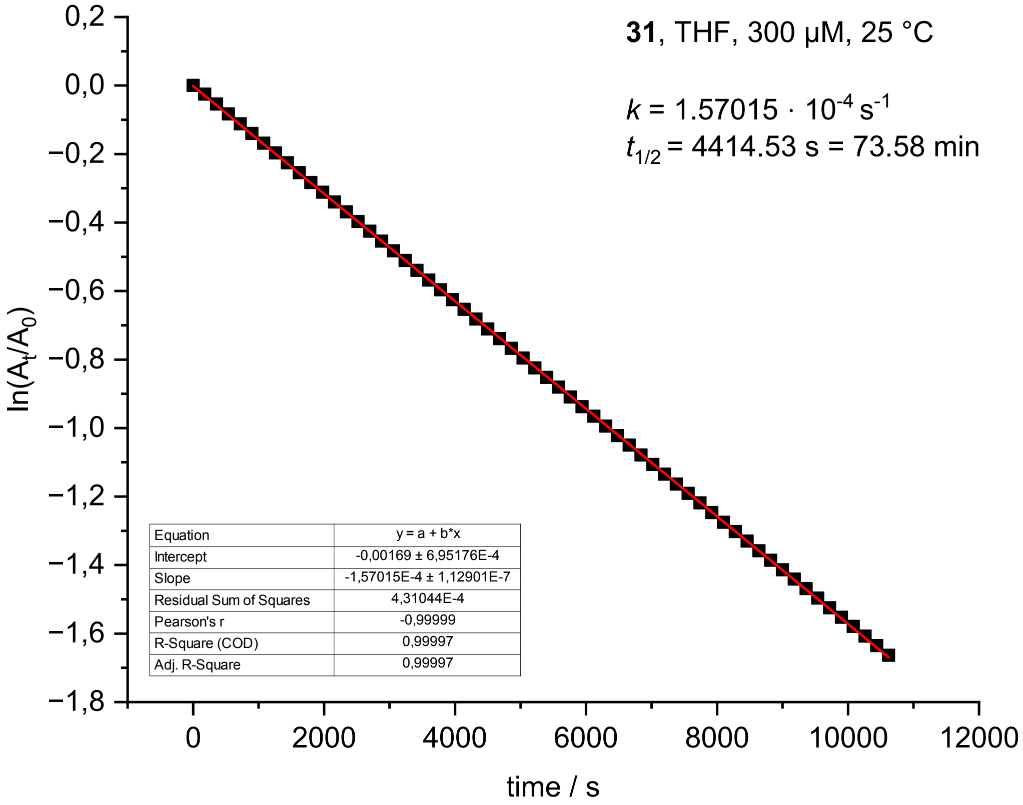

Figure S86: Determination of the thermal half-life $t_{1/2}$ of compound **31** (THF, 300 µM, 298 K) after irradiation with 420 nm (k = 1.57015 · 10^{-4} s^{-1}, $t_{1/2}$ = 73.58 min).

S 4.2 Photostationary states

To suppress thermal relaxation of the re-isomerization, 1.00 mg of each compounds **5**, **18** – **20**, **23** – **26**, **28**, **29** and **31** were dissolved in THF-d_8 and irradiated for 30 s at 273 K with the corresponding optimum wavelength for excitation of the photostationary state (see S 4.1). The following NMR spectra were also measured at 273 K. Due to their insolubility in THF-d_8 at this concentration, 1.00 mg of each compounds **21**, **22**, **27** and **30** were dissolved in DMSO-d_6 and exposed to light for 30 s at 298 K with the optimum wavelengths for excitation of the photostationary state (see S 4.1). The corresponding NMR spectra were measured at 298 K. At this concentration, compounds **21** and **22** first had to be dissolved in hot DMSO-d_6 before an NMR spectrum at 298 K could be measured.

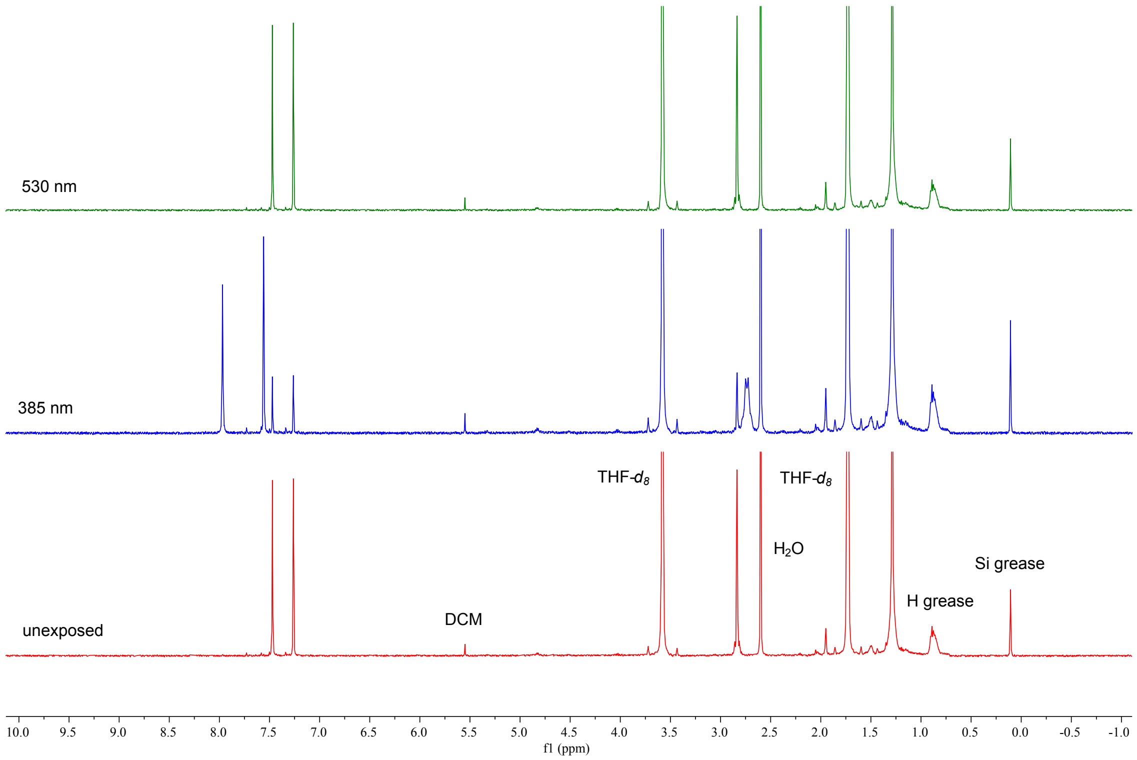

Figure S87: ^{1}H NMR spectra (500 MHz, THF-d_8, 273 K) of compound **5** (red), after irradiation for 30 s at 0 °C with 385 nm (blue) and 530 nm (green).

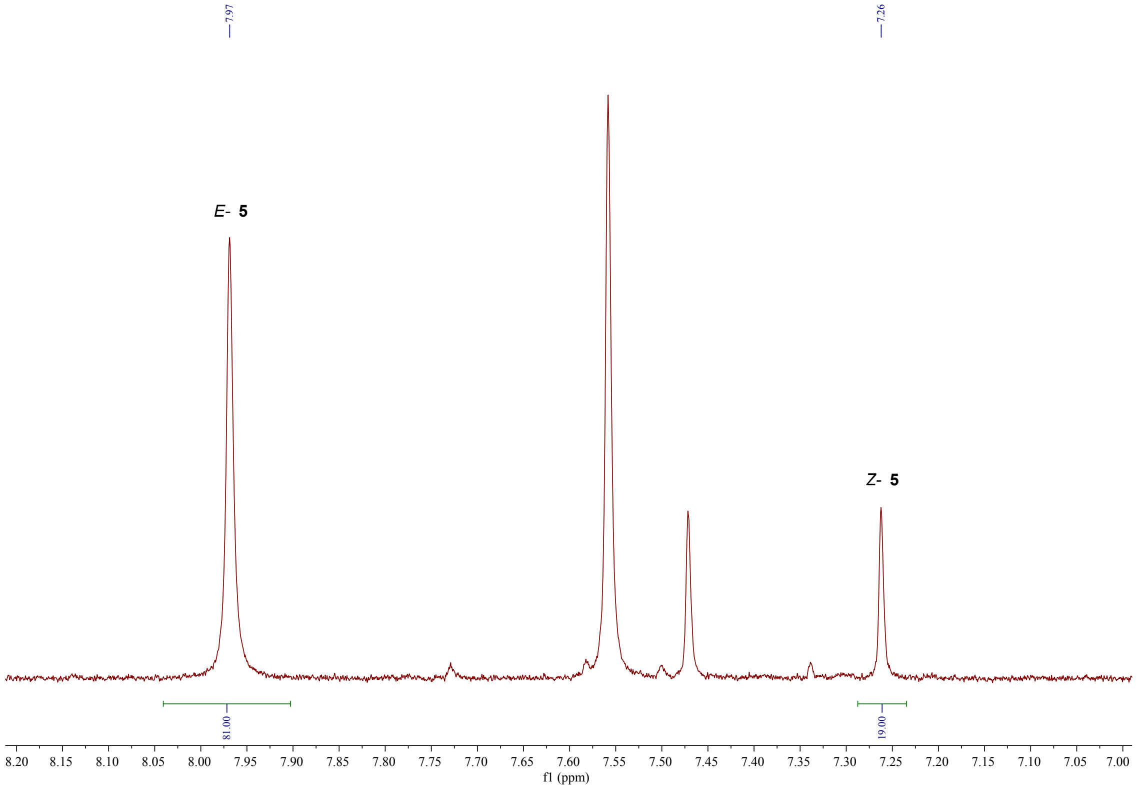

Figure S88: Excerpt of the ^{1}H NMR spectrum (500 MHz, THF-d_8, 273 K) of the photostationary state of compound **5** after irradiation with 385 nm and integration of the corresponding signals of the *Z* and *E* isomers.

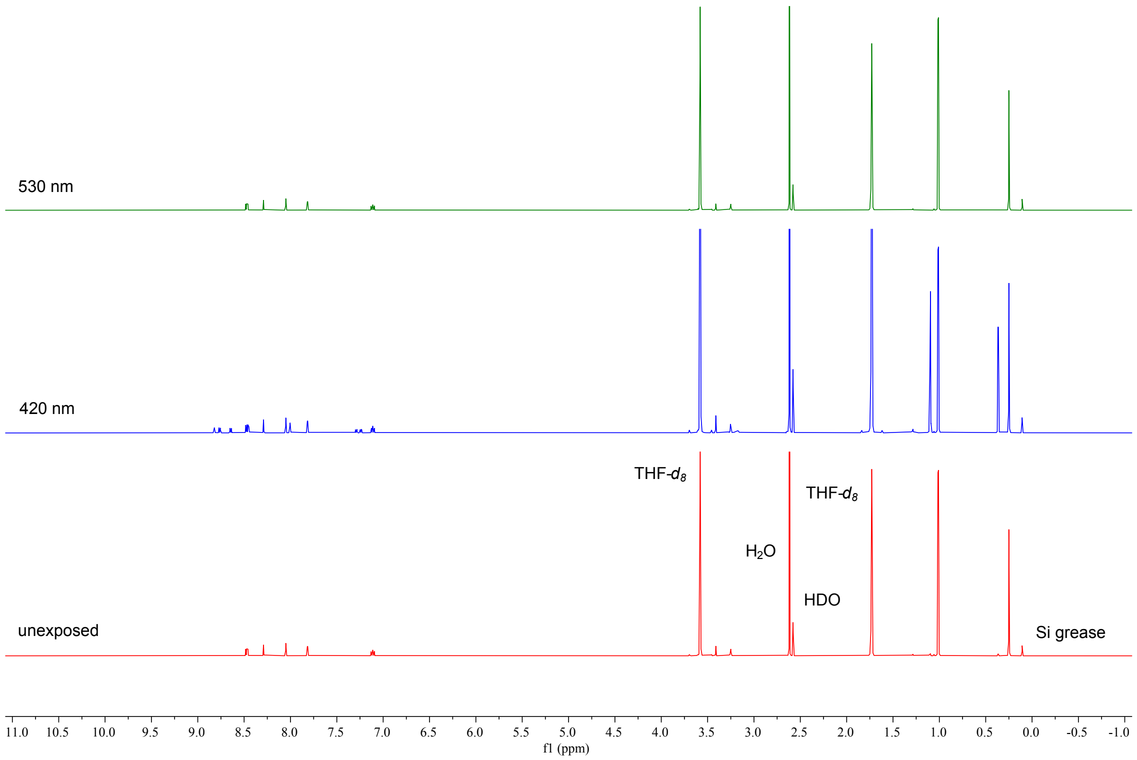

Figure S89: ^{1}H NMR spectra (600 MHz, THF-d_8, 273 K) of compound **18** (red), after irradiation for 30 s at 0 °C with 420 nm (blue) and 530 nm (green).

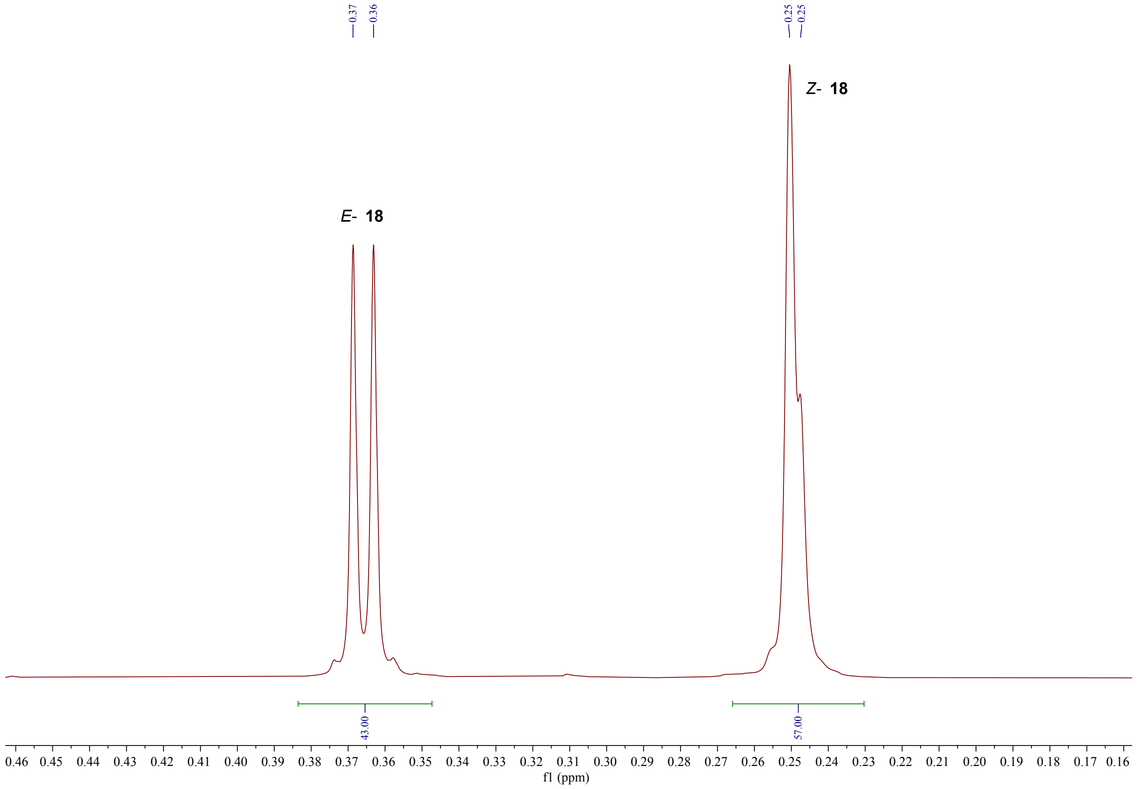

Figure S90: Excerpt of the ^{1}H NMR spectrum (600 MHz, THF-d_8, 273 K) of the photostationary state of compound **18** after irradiation with 420 nm and integration of the corresponding signals of the *Z* and *E* isomers.

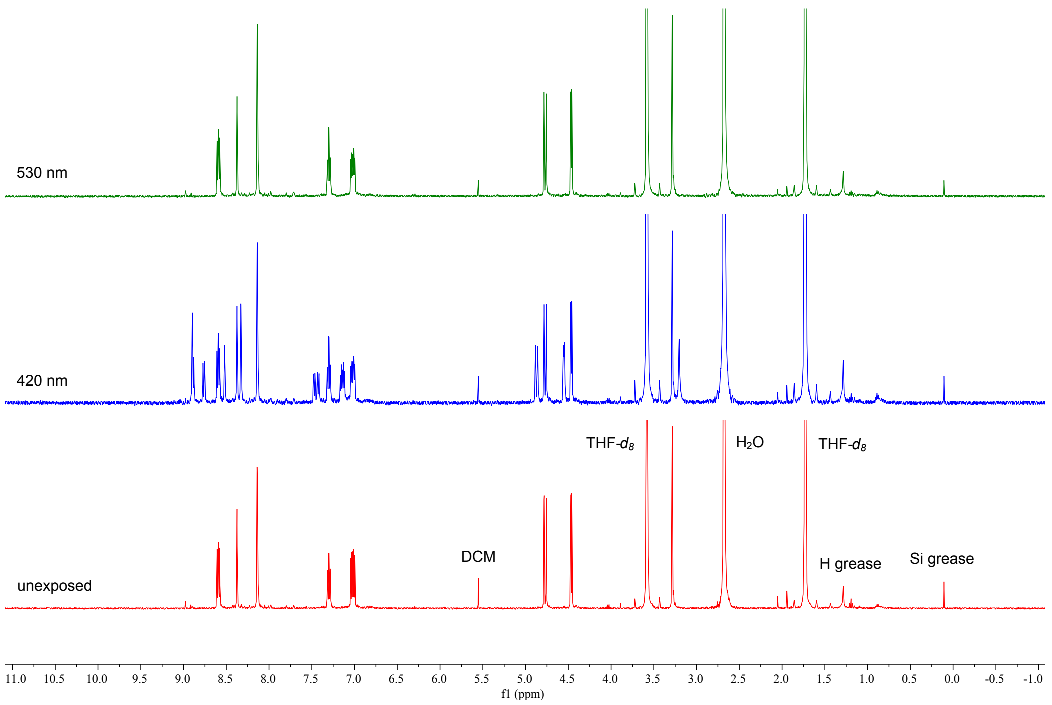

Figure S91: ^{1}H NMR spectra (500 MHz, THF-d_8, 273 K) of compound **19** (red), after irradiation for 30 s at 0 °C with 420 nm (blue) and 530 nm (green).

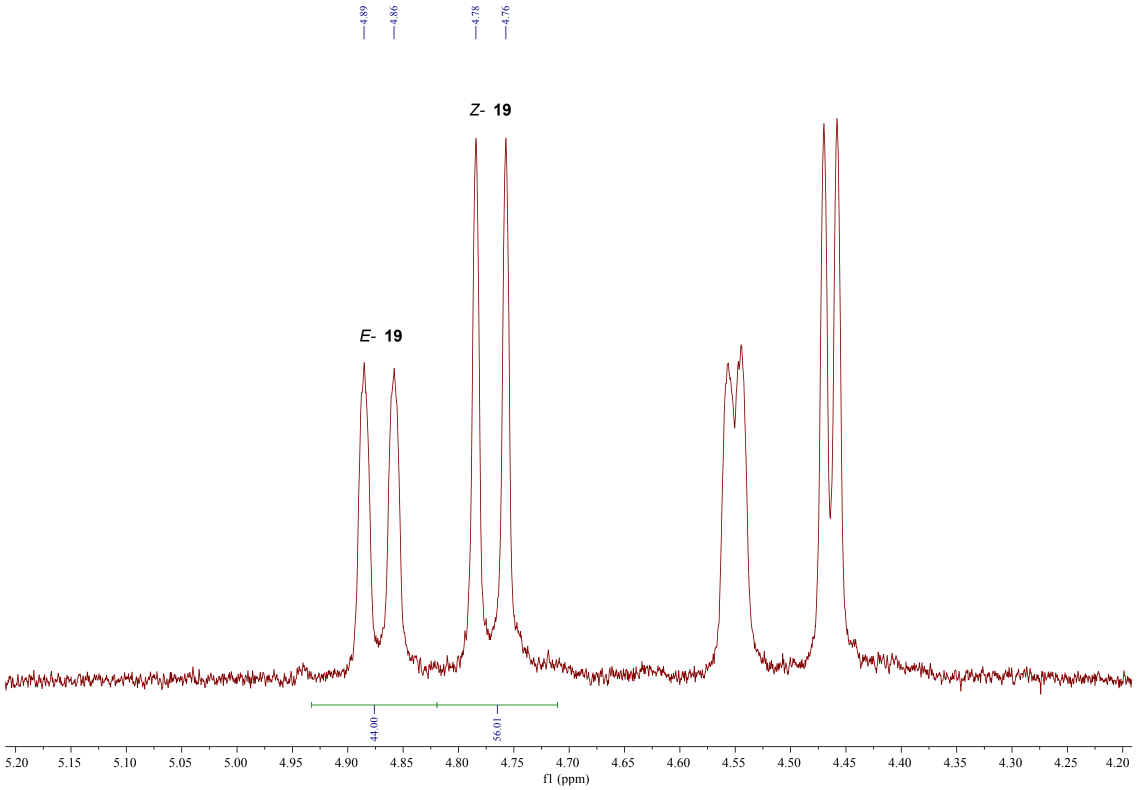

Figure S92: Excerpt of the ^{1}H NMR spectrum (500 MHz, THF-d_8, 273 K) of the photostationary state of compound **19** after irradiation with 420 nm and integration of the corresponding signals of the *Z* and *E* isomers.

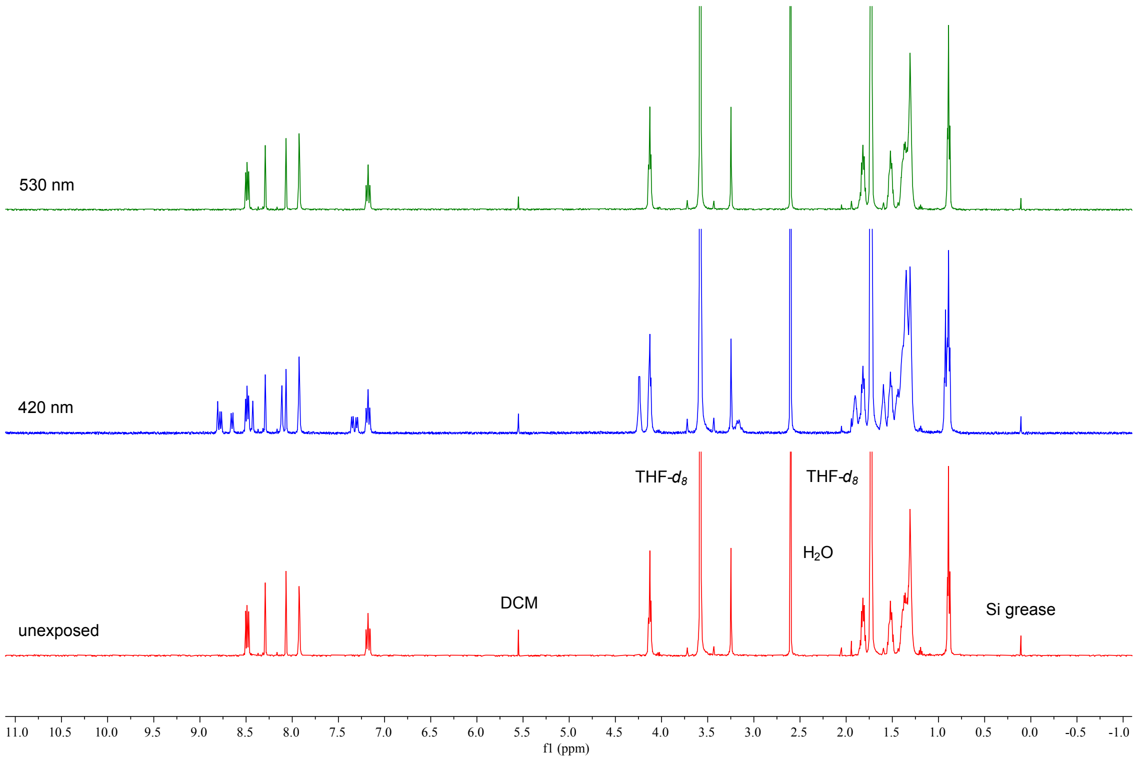

Figure S93: ^{1}H NMR spectra (500 MHz, THF-d_8, 273 K) of compound **20** (red), after irradiation for 30 s at 0 °C with 420 nm (blue) and 530 nm (green).

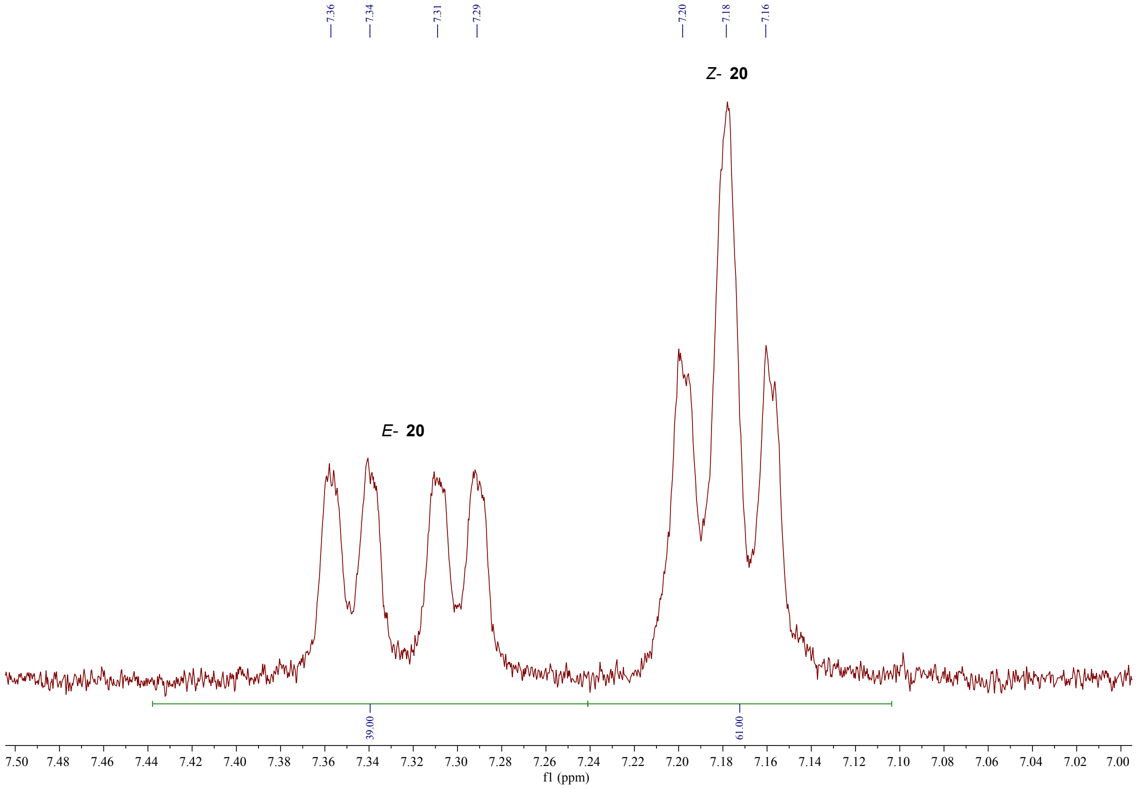

Figure S94: Excerpt of the ^{1}H NMR spectrum (500 MHz, THF-d_8, 273 K) of the photostationary state of compound **20** after irradiation with 420 nm and integration of the corresponding signals of the *Z* and *E* isomers.

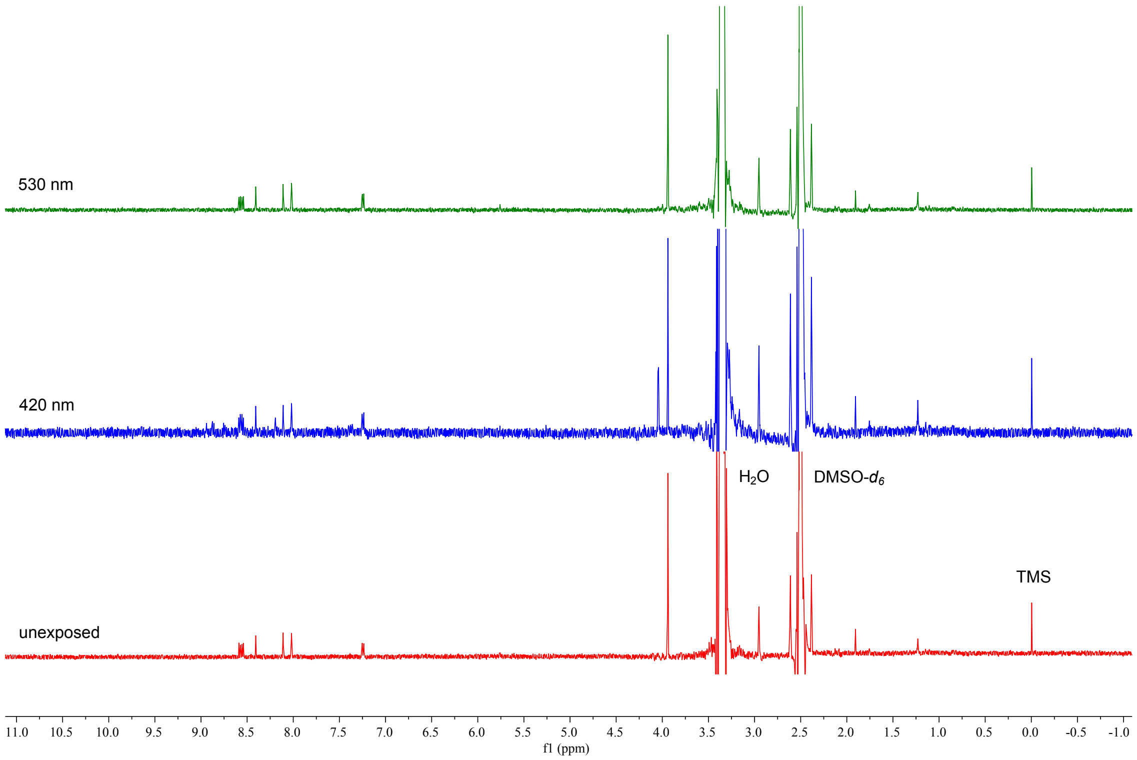

Figure S95: ^{1}H NMR spectra (500 MHz, DMSO-d_6, 298 K) of compound **21** (red), after irradiation for 30 s at 298 K with 420 nm (blue) and 530 nm (green).

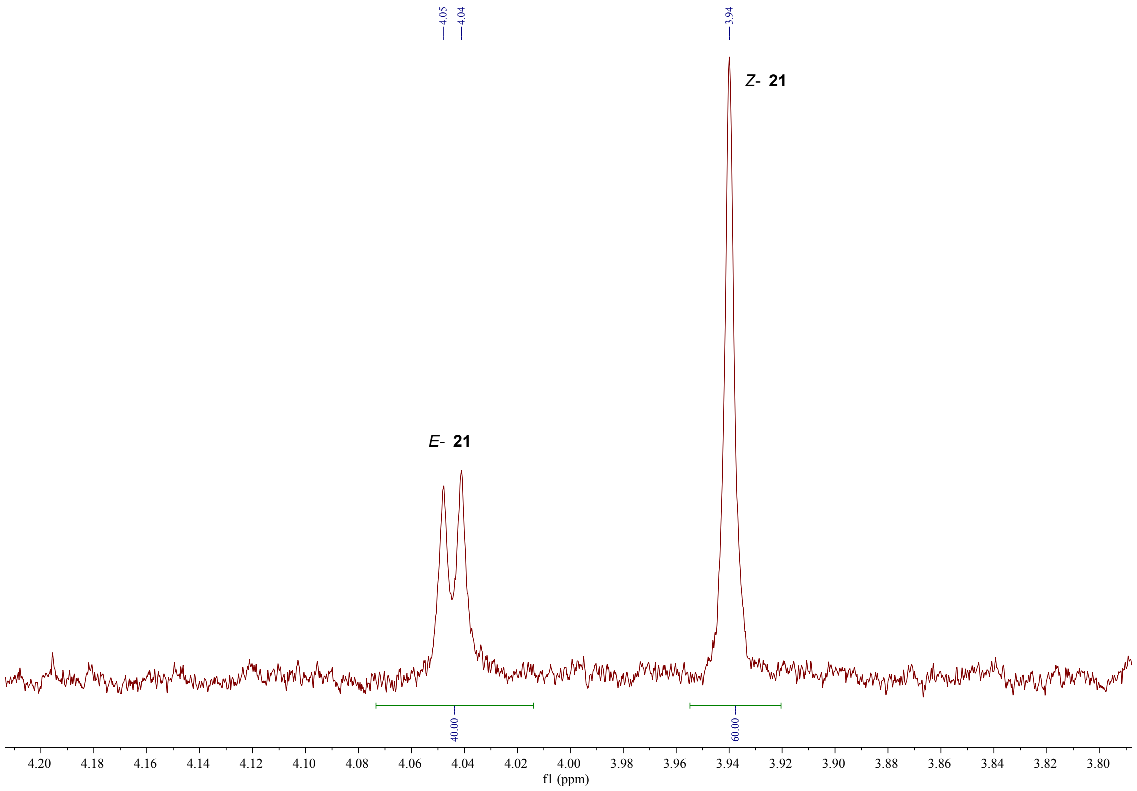

Figure S96: Excerpt of the ^{1}H NMR spectrum (500 MHz, DMSO-d_6, 298 K) of the photostationary state of compound **21** after irradiation with 420 nm and integration of the corresponding signals of the *Z* and *E* isomers.

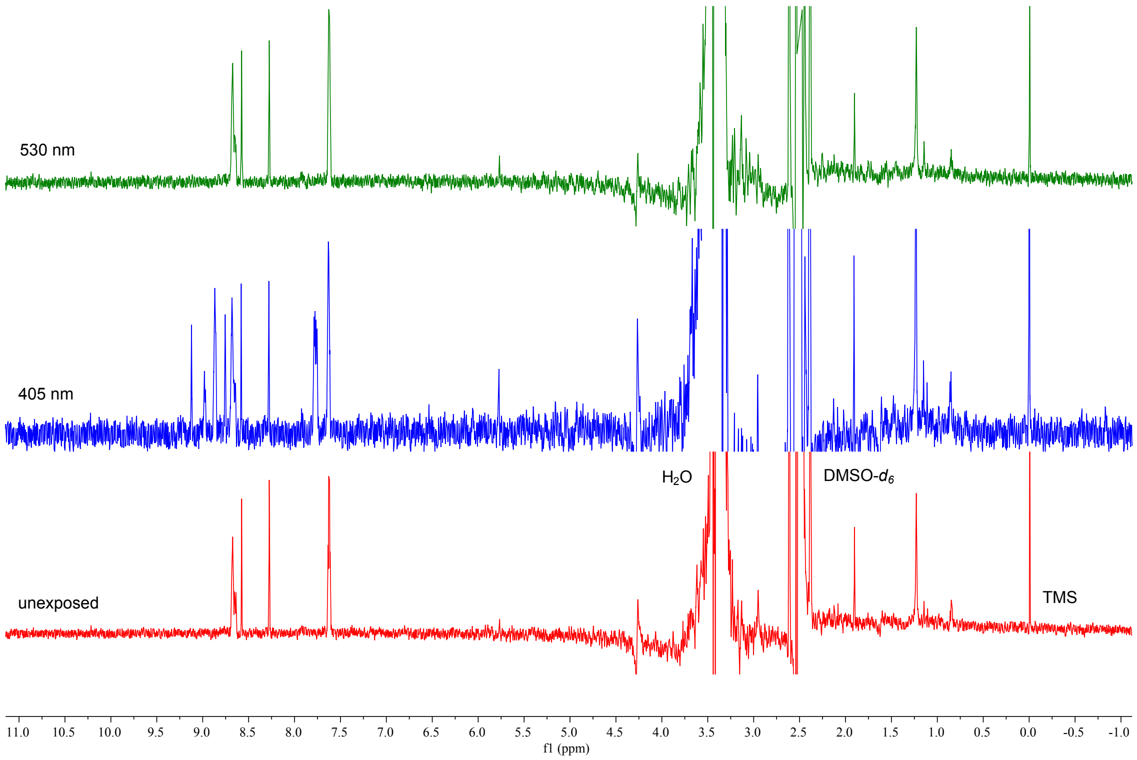

Figure S97: ^{1}H NMR spectra (500 MHz, DMSO-d_6, 298 K) of compound **22** (red), after irradiation for 30 s at 298 K with 405 nm (blue) and 530 nm (green).

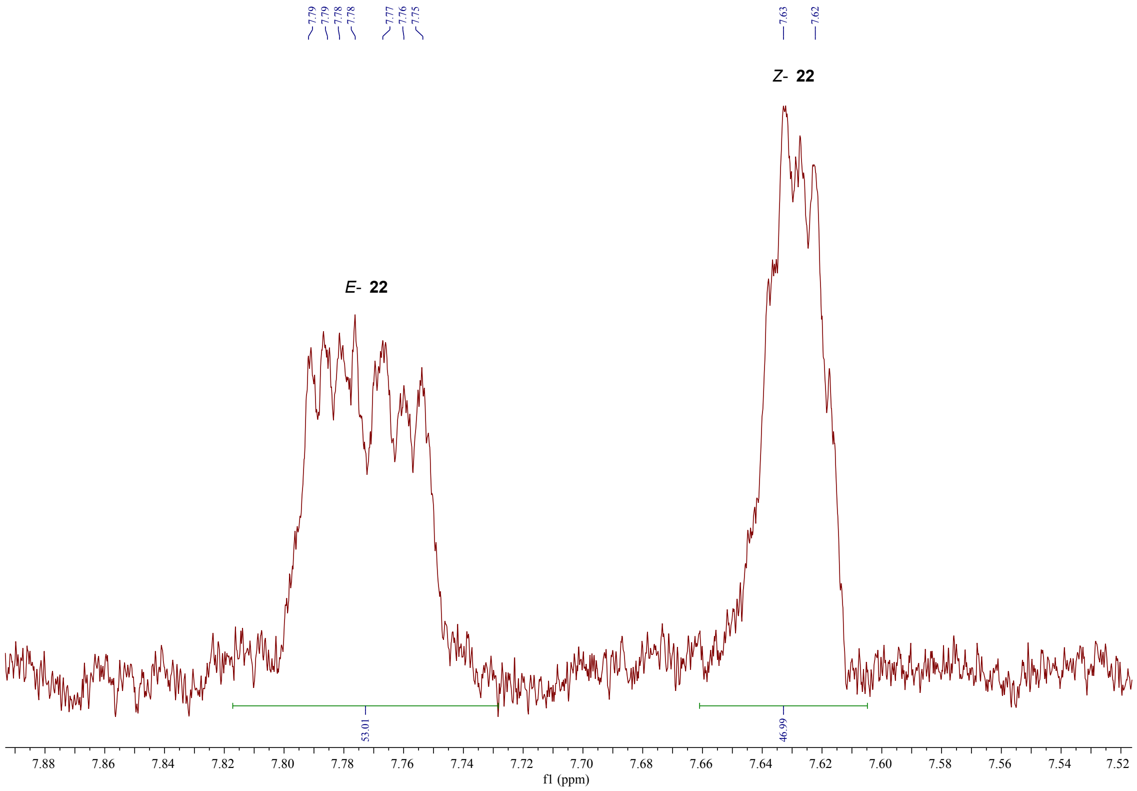

Figure S98: Excerpt of the ^{1}H NMR spectrum (500 MHz, DMSO-d_6, 298 K) of the photostationary state of compound **22** after irradiation with 405 nm and integration of the corresponding signals of the *Z* and *E* isomers.

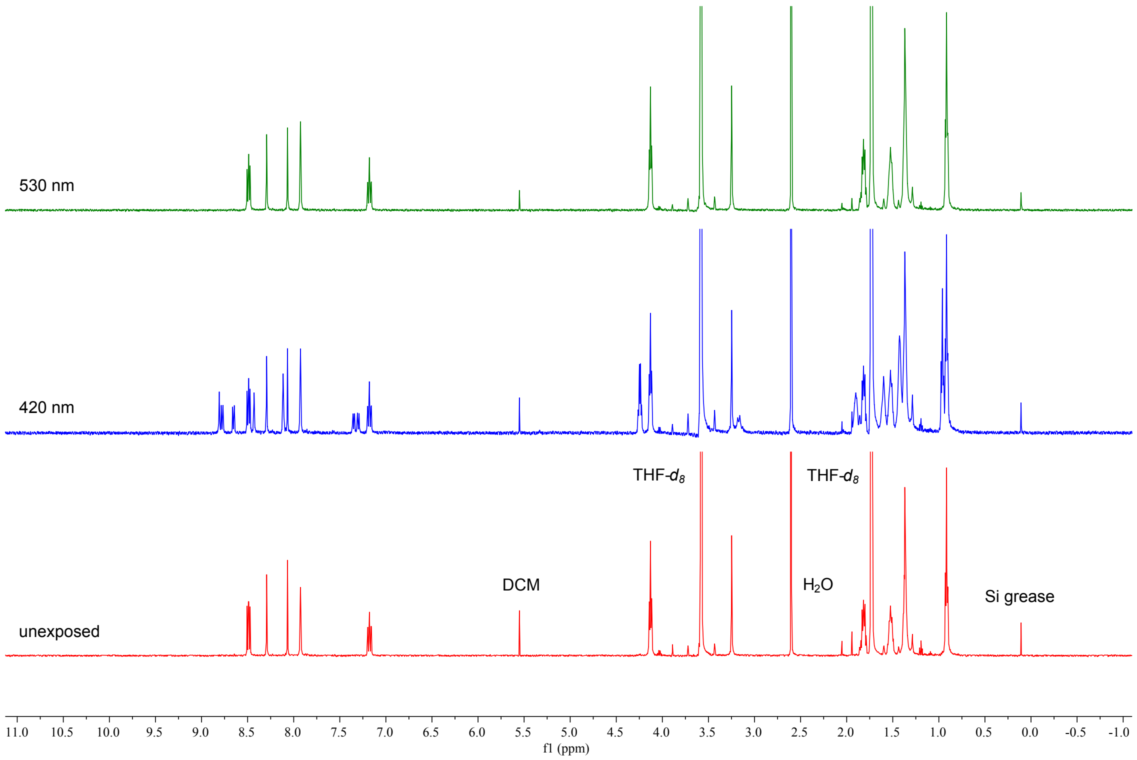

Figure S99: ^{1}H NMR spectra (500 MHz, THF-d_8, 273 K) of compound **23** (red), after irradiation for 30 s at 0 °C with 420 nm (blue) and 530 nm (green).

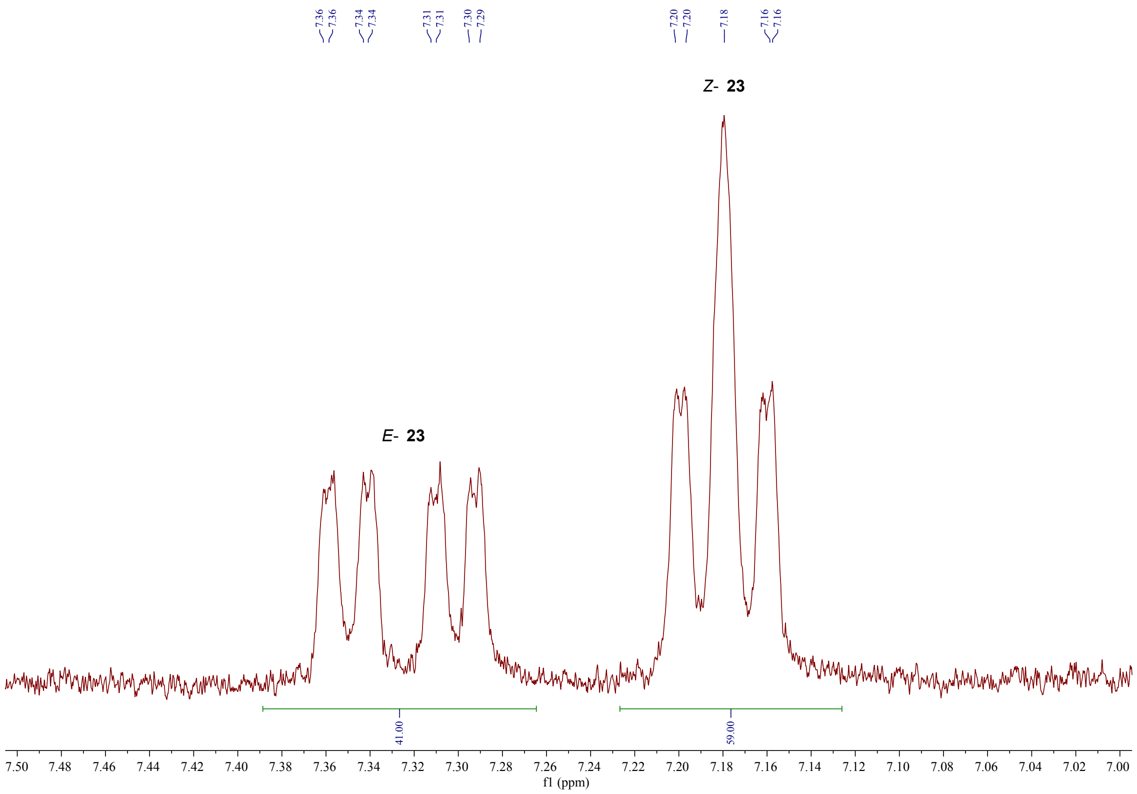

Figure S100: Excerpt of the ^{1}H NMR spectrum (500 MHz, THF-d_8, 273 K) of the photostationary state of compound **23** after irradiation with 420 nm and integration of the corresponding signals of the *Z* and *E* isomers.

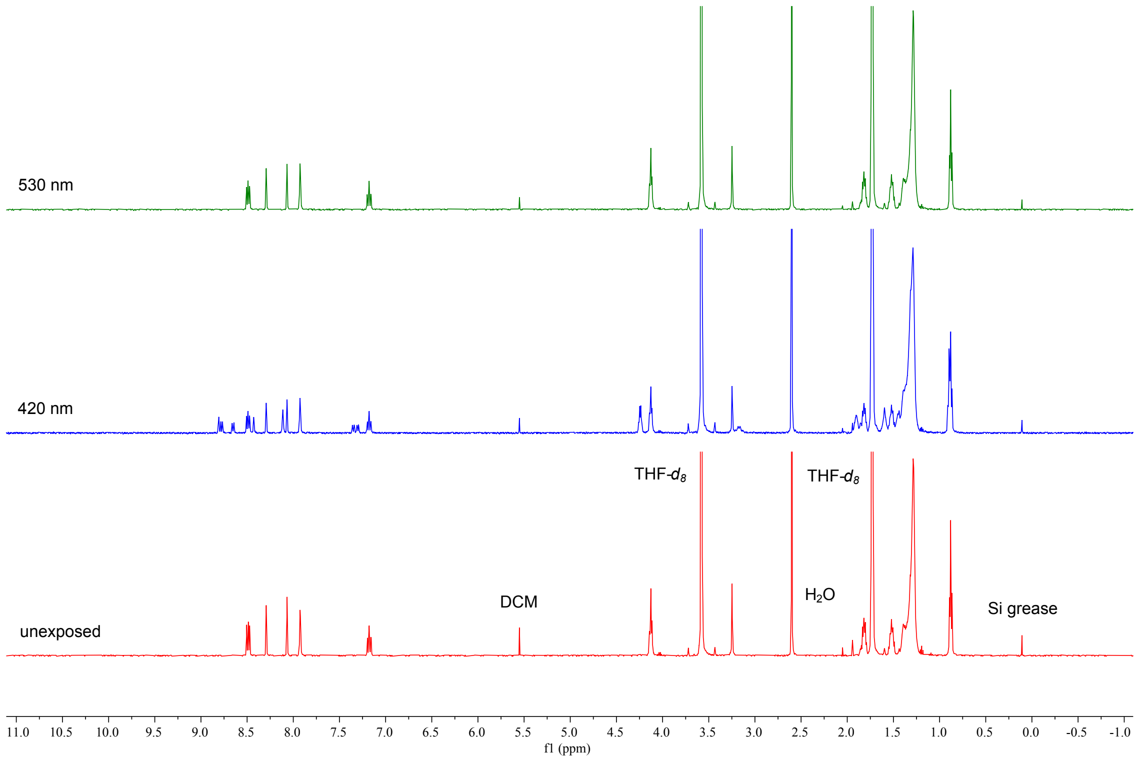

Figure S101: ^{1}H NMR spectra (500 MHz, THF-d_8, 273 K) of compound **24** (red), after irradiation for 30 s at 0 °C with 420 nm (blue) and 530 nm (green).

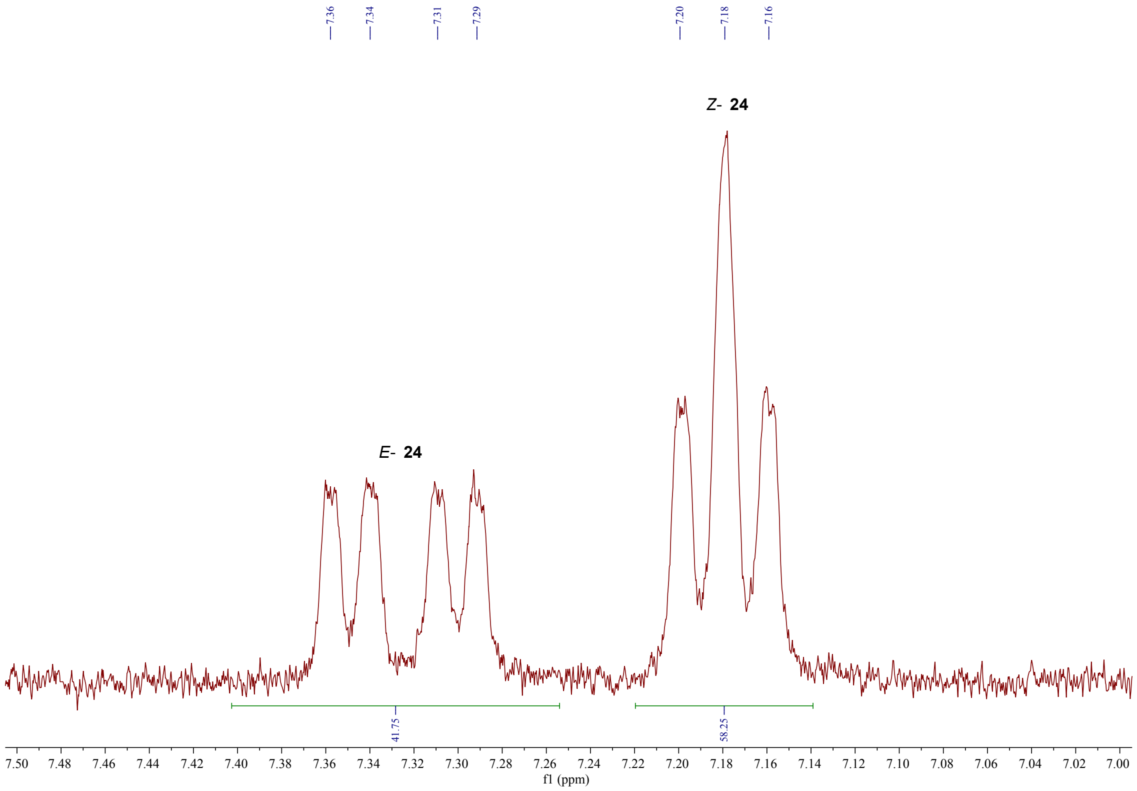

Figure S102: Excerpt of the ^{1}H NMR spectrum (500 MHz, THF-d_8, 273 K) of the photostationary state of compound **24** after irradiation with 420 nm and integration of the corresponding signals of the *Z* and *E* isomers.

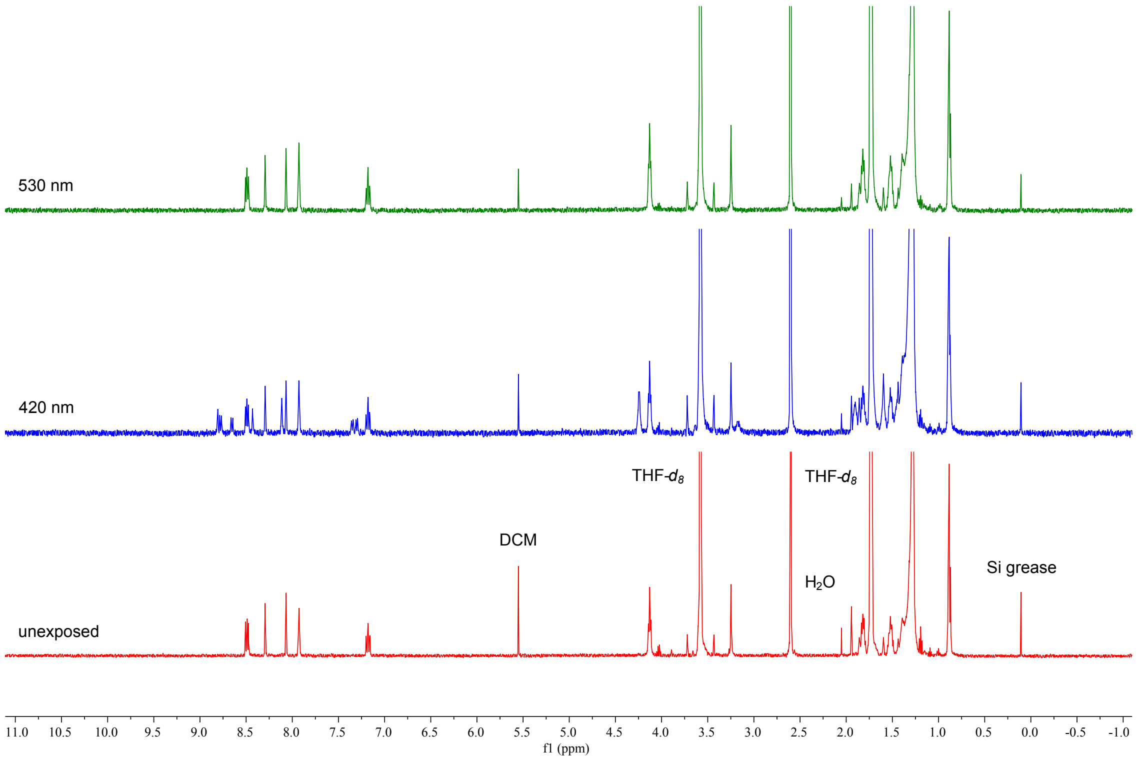

Figure S103: ^{1}H NMR spectra (500 MHz, THF-d_8, 273 K) of compound **25** (red), after irradiation for 30 s at 0 °C with 420 nm (blue) and 530 nm (green).

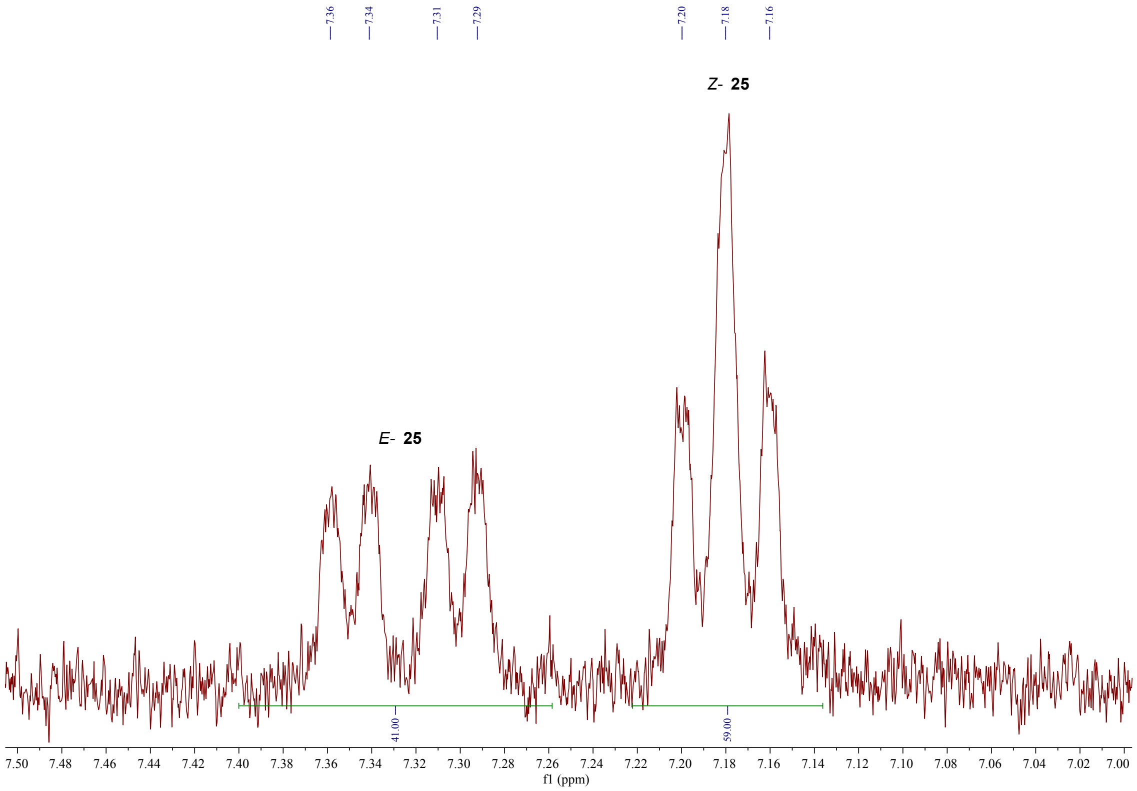

Figure S104: Excerpt of the ^{1}H NMR spectrum (500 MHz, THF-d_8, 273 K) of the photostationary state of compound **25** after irradiation with 420 nm and integration of the corresponding signals of the *Z* and *E* isomers.

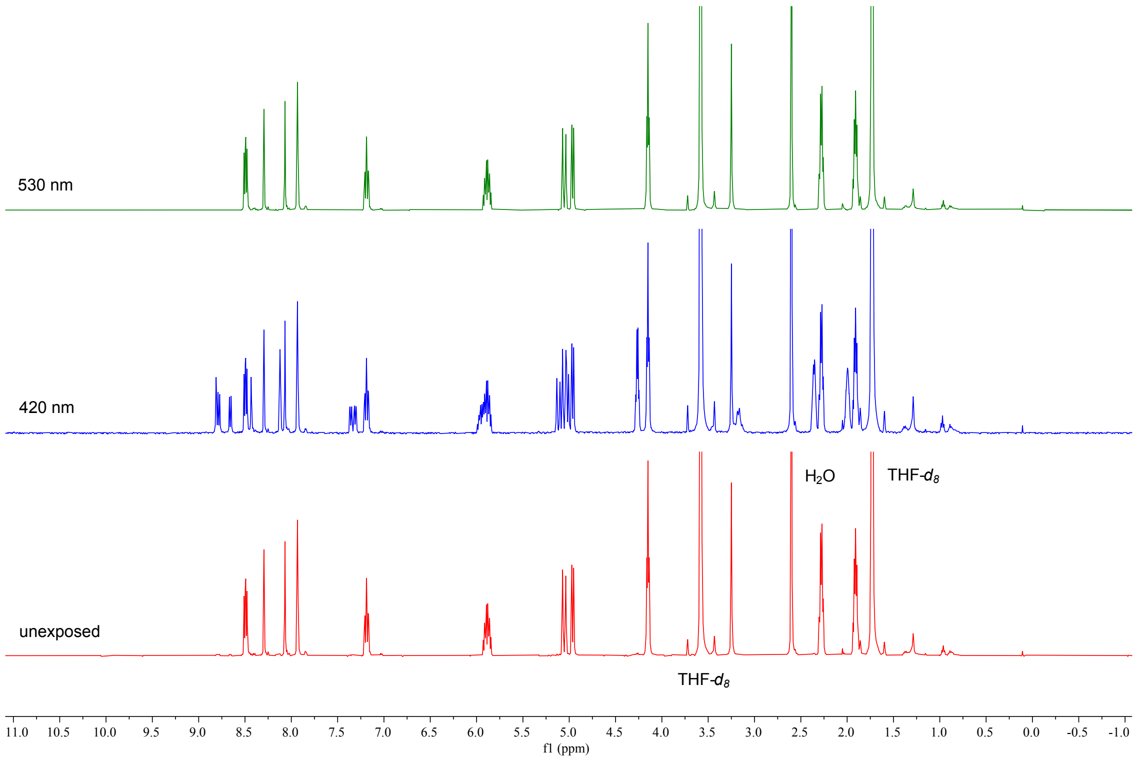

Figure S105: ^{1}H NMR spectra (500 MHz, THF-d_8, 273 K) of compound **26** (red), after irradiation for 30 s at 0 °C with 420 nm (blue) and 530 nm (green).

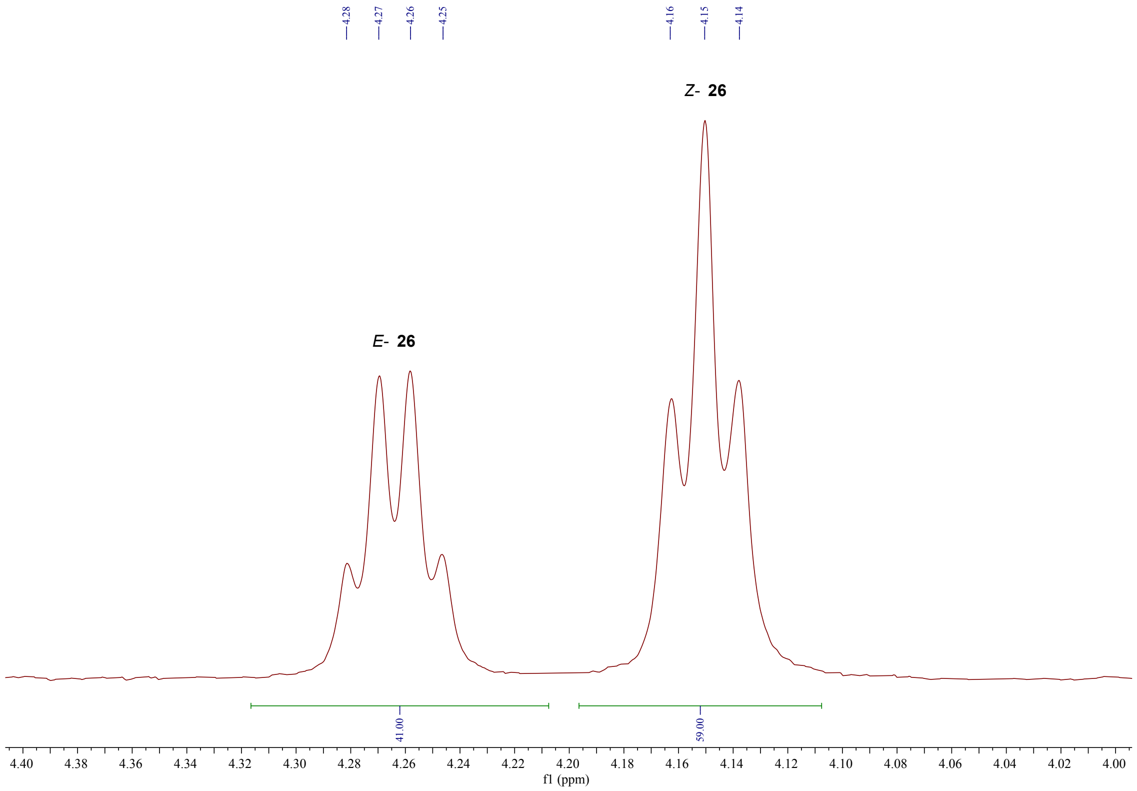

Figure S106: Excerpt of the ^{1}H NMR spectrum (500 MHz, THF-d_8, 273 K) of the photostationary state of compound **26** after irradiation with 420 nm and integration of the corresponding signals of the *Z* and *E* isomers.

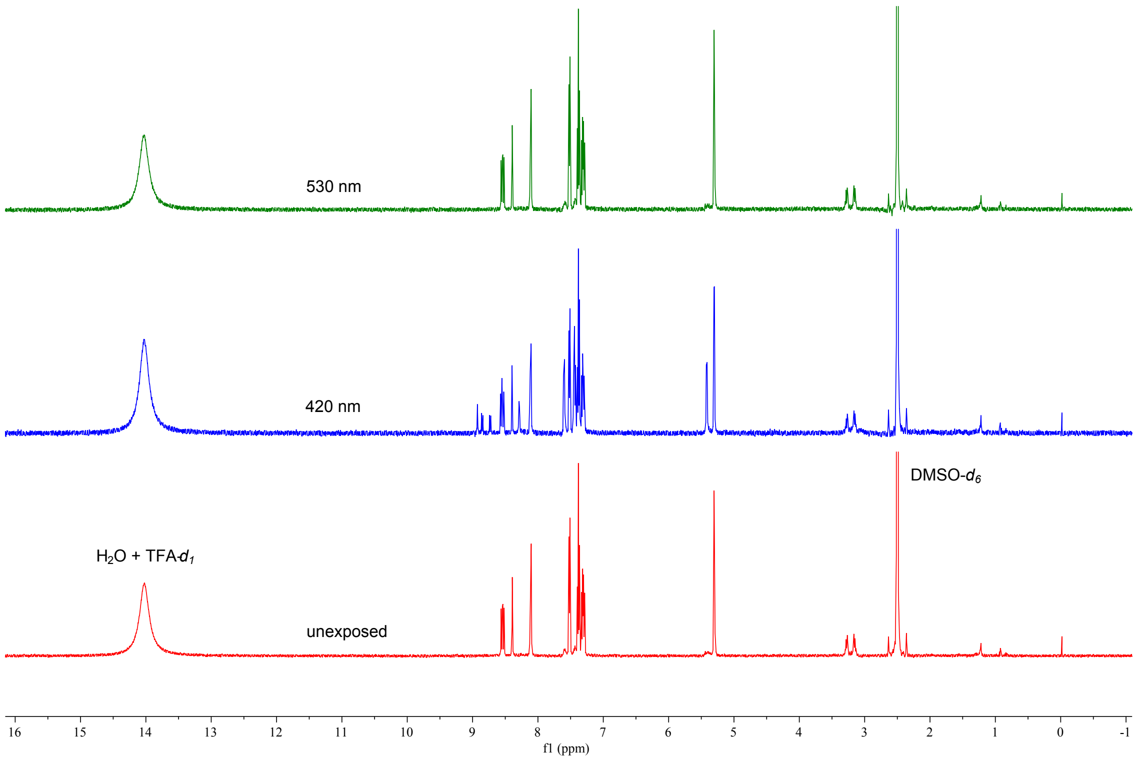

Figure S107: ^{1}H NMR spectra (500 MHz, DMSO-d_6 + TFA-d_1, 298 K) of compound **27** (red), after irradiation for 30 s at 298 K with 420 nm (blue) and 530 nm (green).

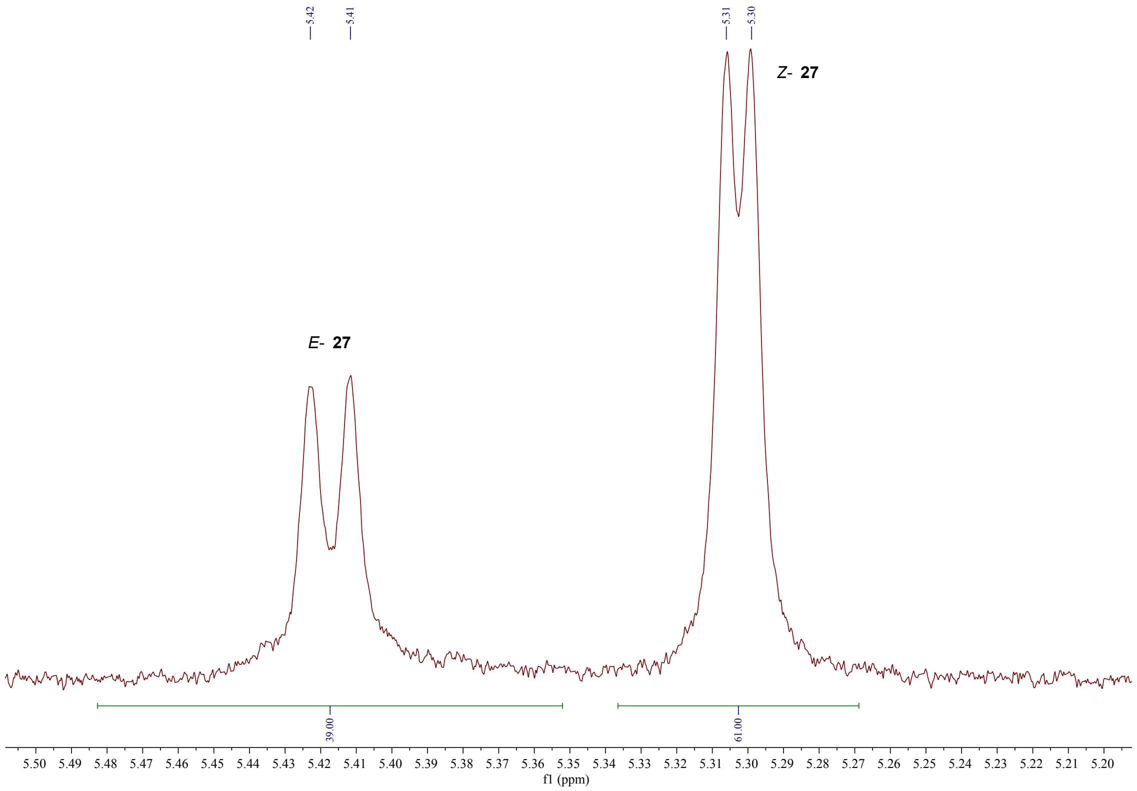

Figure S108: Excerpt of the ^{1}H NMR spectrum (500 MHz, DMSO-d_6 + TFA-d_1, 298 K) of the photostationary state of compound **27** after irradiation with 420 nm and integration of the corresponding signals of the *Z* and *E* isomers.

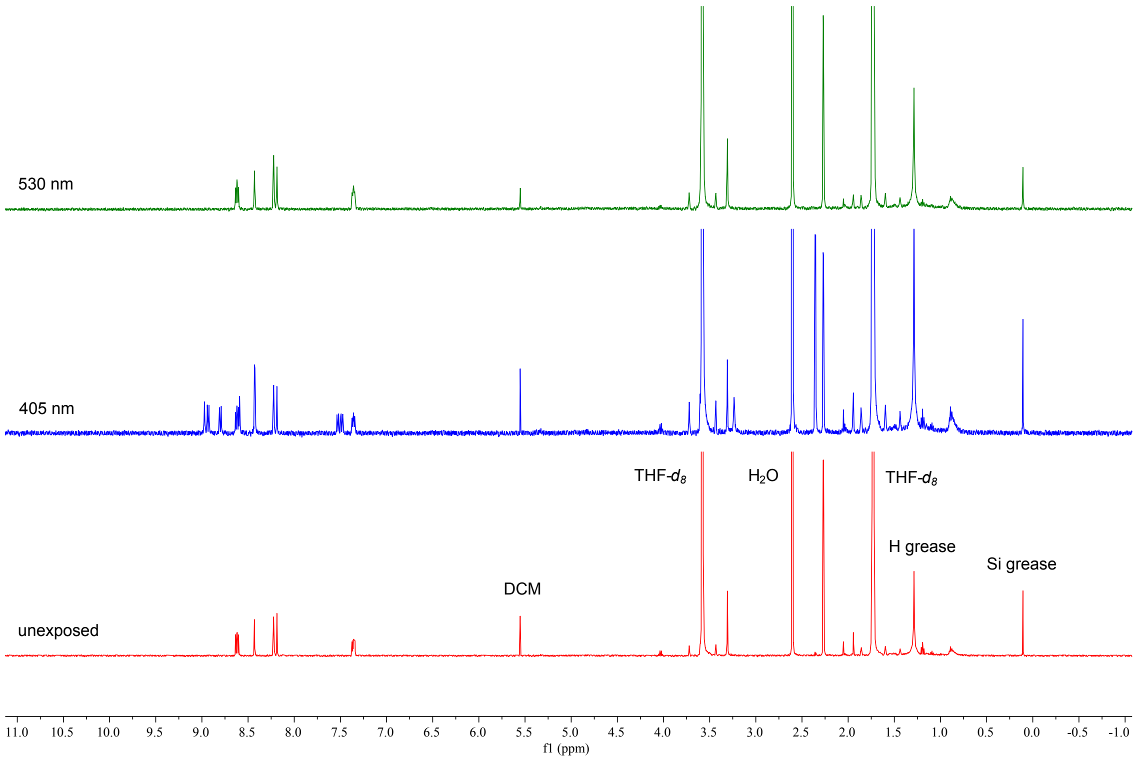

Figure S109: ^{1}H NMR spectra (500 MHz, THF-d_8, 273 K) of compound **28** (red), after irradiation for 30 s at 0 °C with 405 nm (blue) and 530 nm (green).

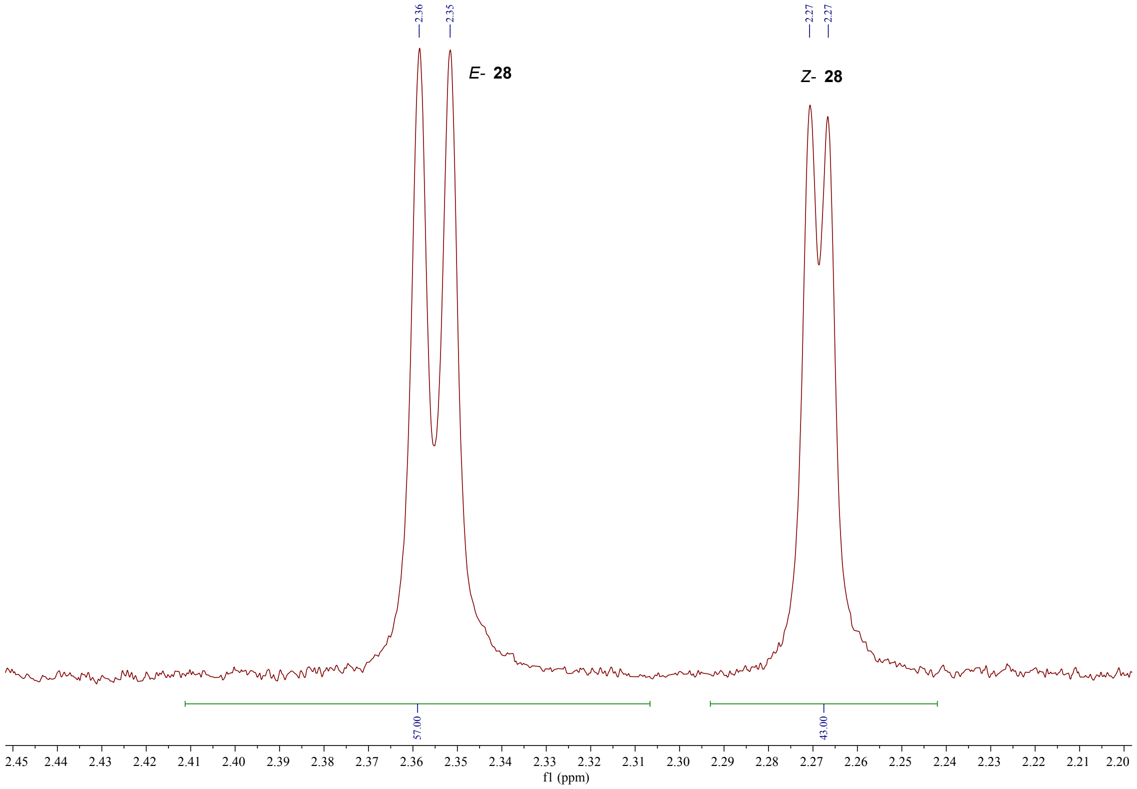

Figure S110: Excerpt of the ^{1}H NMR spectrum (500 MHz, THF-d_8, 273 K) of the photostationary state of compound **28** after irradiation with 405 nm and integration of the corresponding signals of the *Z* and *E* isomers.

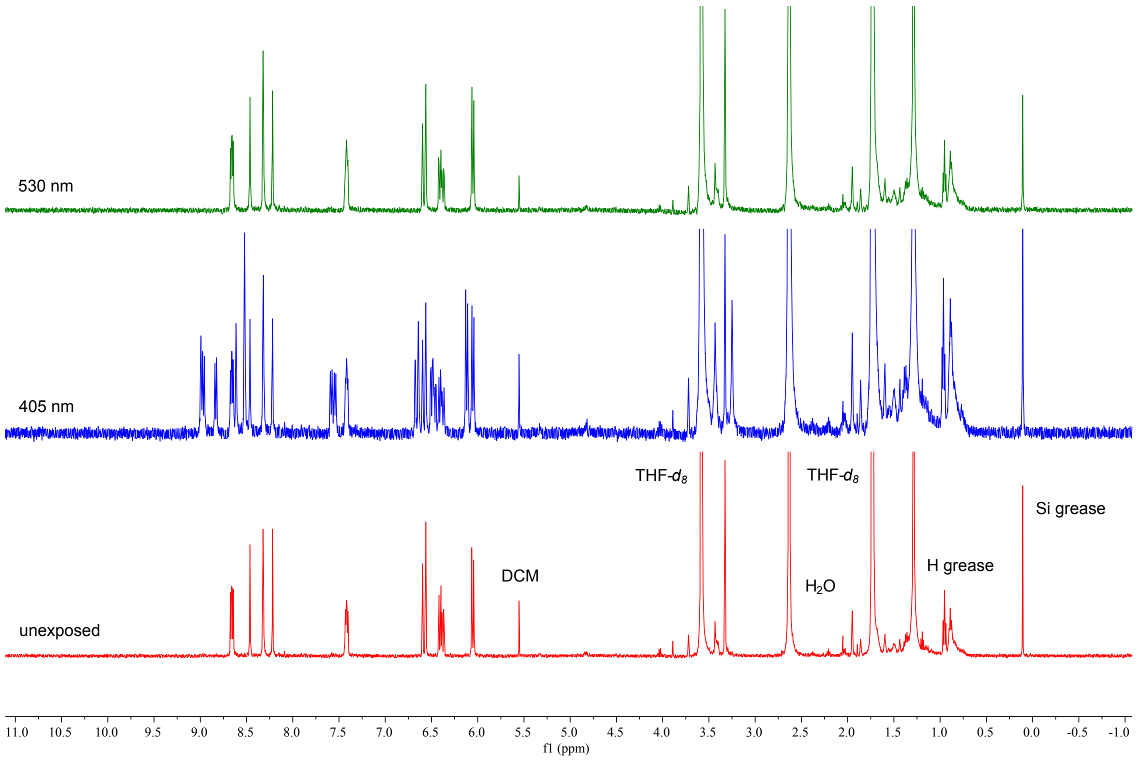

Figure S111: ^{1}H NMR spectra (500 MHz, THF-d_8, 273 K) of compound **29** (red), after irradiation for 30 s at 0 °C with 405 nm (blue) and 530 nm (green).

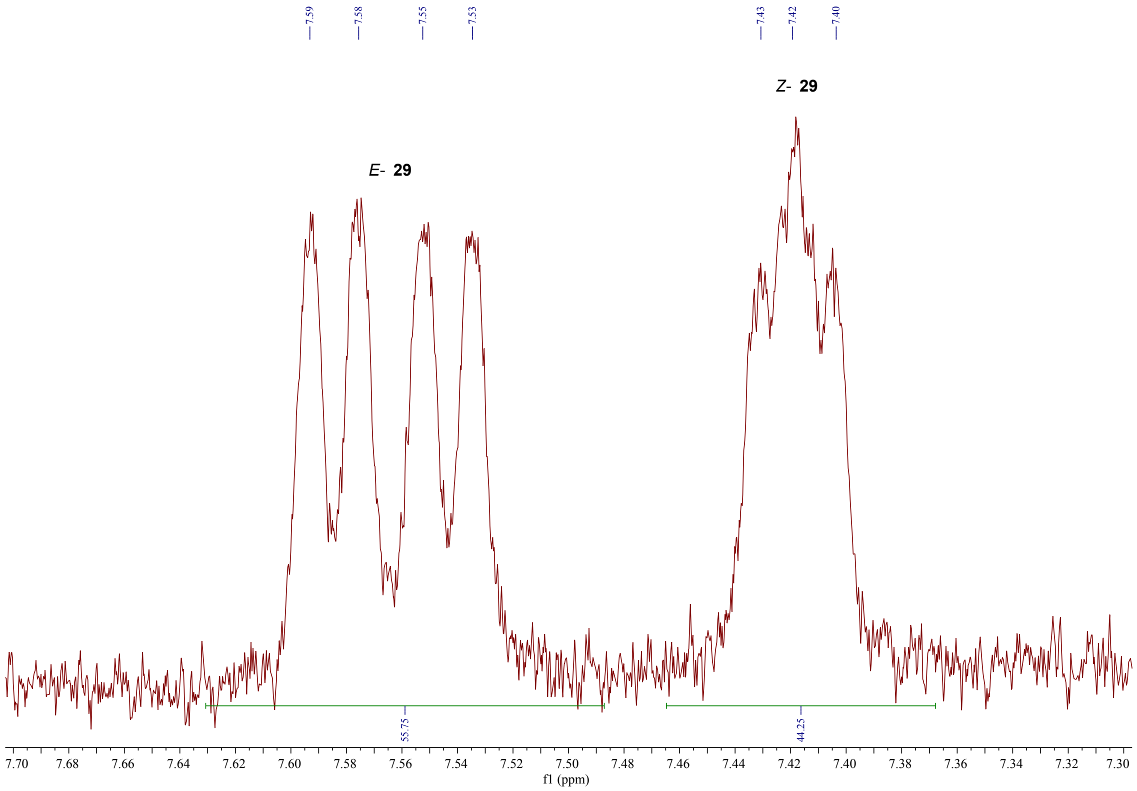

Figure S112: Excerpt of the ^{1}H NMR spectrum (500 MHz, THF-d_8, 273 K) of the photostationary state of compound **29** after irradiation with 405 nm and integration of the corresponding signals of the *Z* and *E* isomers.

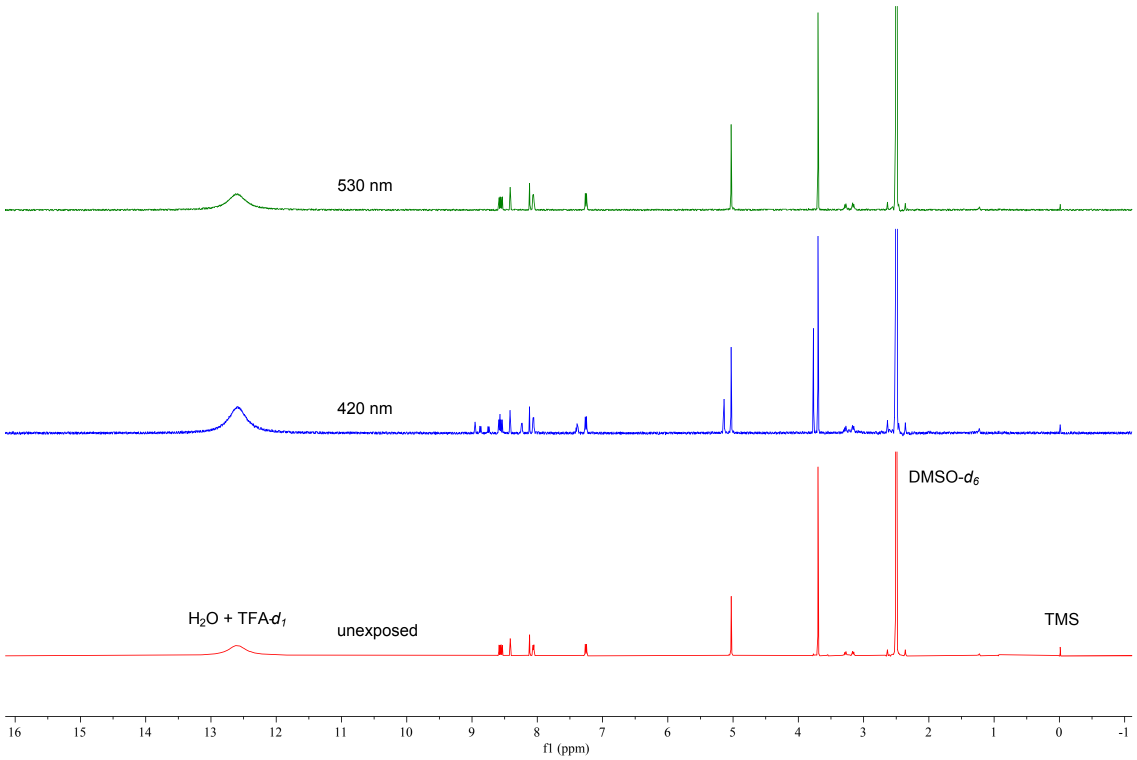

Figure S113: ^{1}H NMR spectra (500 MHz, DMSO-d_6 + TFA-d_1, 298 K) of compound **30** (red), after irradiation for 30 s at 298 K with 420 nm (blue) and 530 nm (green).

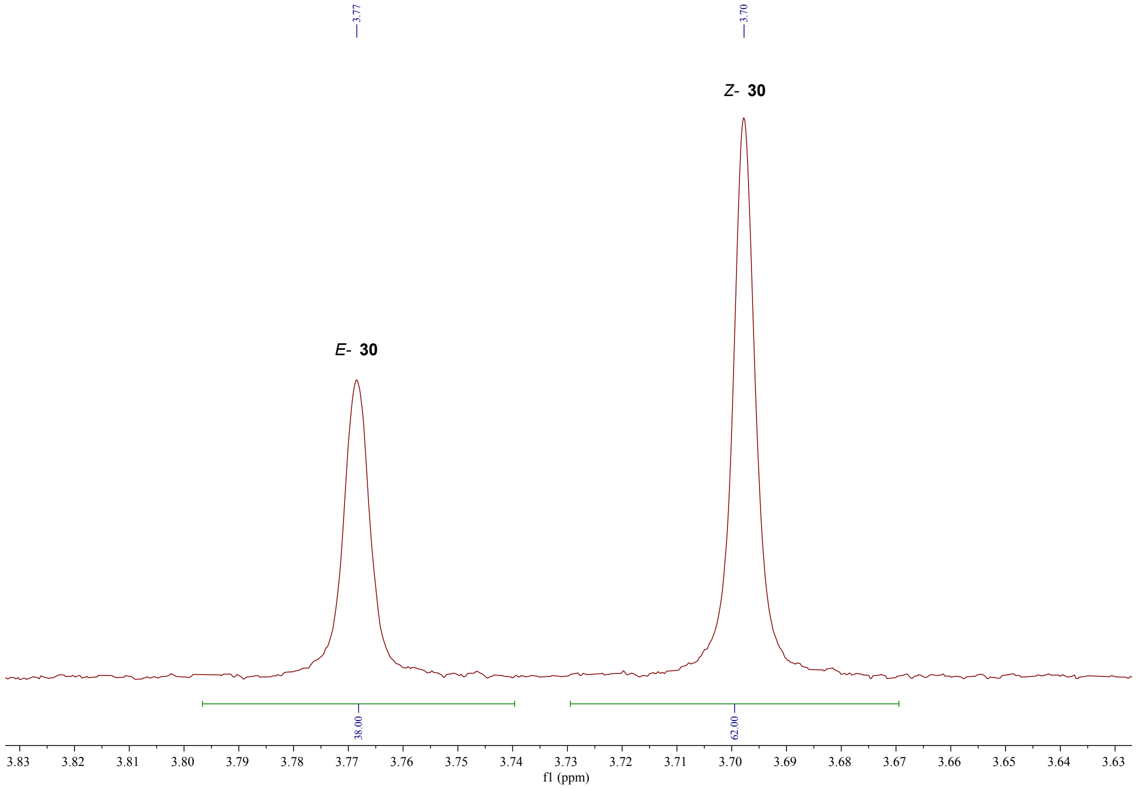

Figure S114: Excerpt of the ^{1}H NMR spectrum (500 MHz, DMSO-d_6 + TFA-d_1, 298 K) of the photostationary state of compound **30** after irradiation with 420 nm and integration of the corresponding signals of the *Z* and *E* isomers.

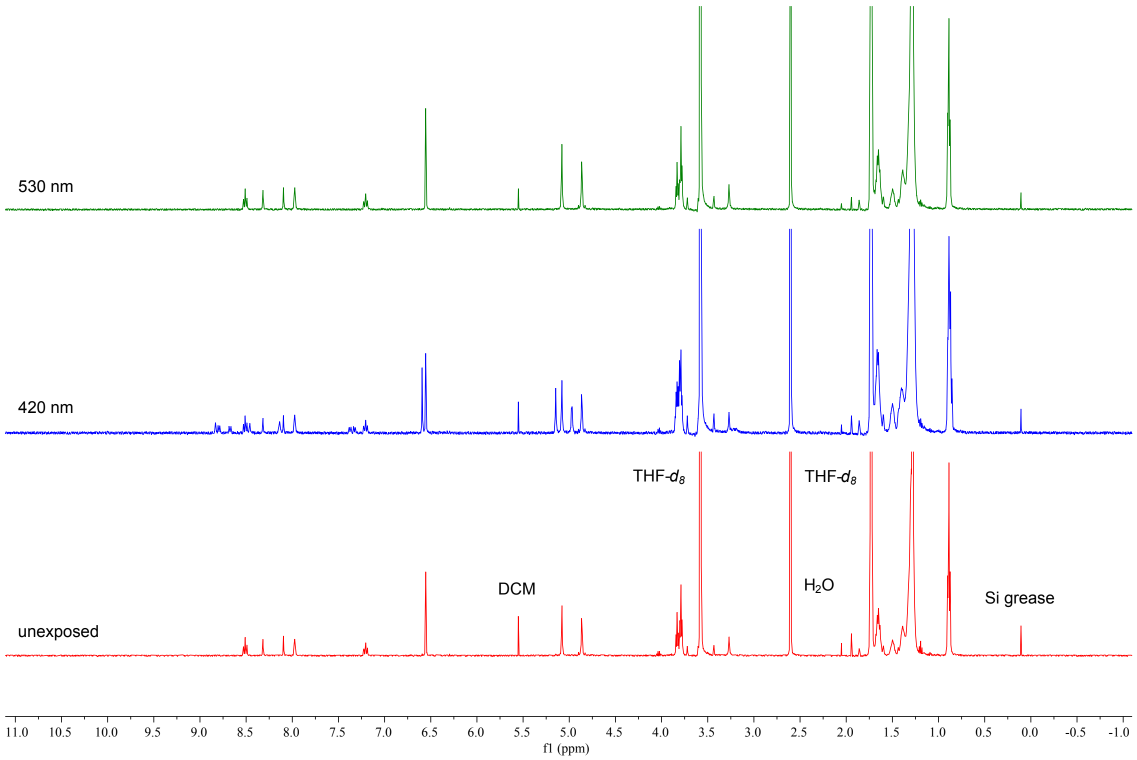

Figure S115: ^{1}H NMR spectra (500 MHz, THF-d_8, 273 K) of compound **31** (red), after irradiation for 30 s at 0 °C with 420 nm (blue) and 530 nm (green).

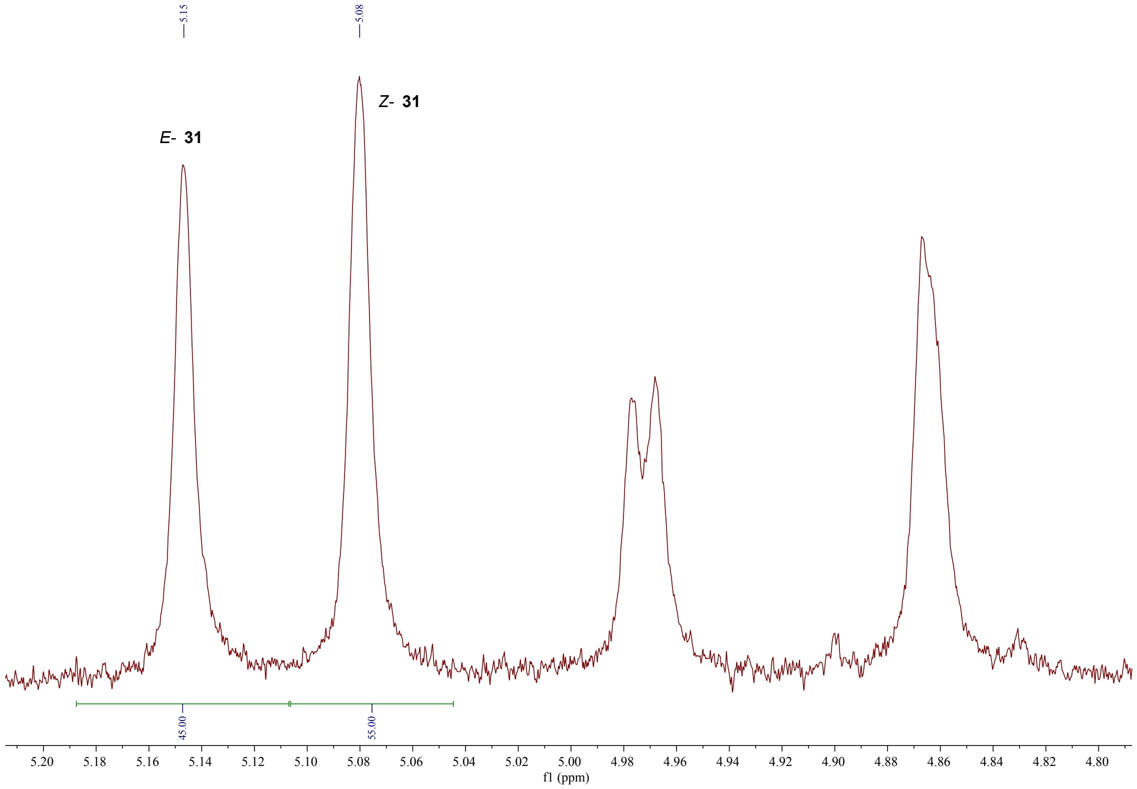

Figure S116: Excerpt of the ^{1}H NMR spectrum (500 MHz, THF-d_8, 273 K) of the photostationary state of compound **31** after irradiation with 420 nm and integration of the corresponding signals of the *Z* and *E* isomers.

S 5 X-ray single crystal structure analysis

Crystallization of TPD **18** was carried out through vapor diffusion experiments using chloroform as the solvent and methanol as the anti-solvent. Needle-like crystals of TPD **18** were obtained. A few milligrams of TPDs **21** and **22** were each added to DMSO in a NMR tube and heated until a clear solution was formed. The hot solutions were then cooled by 5 °C each day until crystallization of the compounds **21** and **22** was obtained.

Data collections were performed with a XtaLAB Synergy, Dualflex, HyPix diffractometer that is equipped with a micro focus tube. The structures were solved with SHELXT [1] and refined with SHELXL [2] using Least Squares minimization. In compound **21** and **22** all non-hydrogen atoms were refined anisotropic, whereas in compound **18** the disordered C atoms with lower occupancy (sof = 0.2) were refined isotropic. The C-H H atoms were positioned with idealized geometry (methyl H atoms allowed to rotate but not to tip) and were refined isotropic with $U_{iso}(H) = 1.2\ U_{eq}(C)$ (1.5 for methyl H atoms) using a riding model. Selected crystal data and details of the structure determinations can be found in Table S1 and ORTEP plots are shown in Figures S118 – S120.

CCDC-2330526 (**18**), CCDC-2330527 (**21**) and CCDC-2330525 (**22**) contain the supplementary crystallographic data for this paper. These data can be obtained free charge from the Cambridge Crystallographic Data Centre via http://www.ccdc.cam.ac.uk/data_request/cif.

1. Sheldrick, G.M. (2015). Acta Cryst. A71, 3-8.
2. Sheldrick, G.M. (2015). Acta Cryst. C71, 3-8.

The structure of TPDs **21** and **22** can also be refined in the centrosymmetric space group *I*2/*a* because but in this case the central C and N atoms are disordered and occupy nearly the same position, which is difficult to resolve. Both structures can be refined using a split model but this leads to significant higher reliability factors, unusual bond lengths and despite the use of restraints to unusual components of the anisotropic displacement parameters. For **22** structure Platon suggest a pseudo center of inversion and space group *I*2/*a* that fits to 91%.
In space group *Ia* there are no indication for disorder and the overall refinement leads to much better reliability factors (R1 = 6.9 for **21** and 7.96 for **22**) and more reasonable bond lengths.
In space group *Ia*, there are some correlations between some of the positional parameters indicating for higher symmetry, but the overall refinement is much better in the non-centrosymmetric space group and the models are more reasonable. Therefore, the non-centrosymmetric space group was selected. Because no strong anomalous scattering atoms are present, the absolute structure cannot be determined, because the Flack x-parameter do not refine to a reasonable value with extremely large uncertainty.
Finally, the non-centrosymmetric arrangement of the molecules is obvious from the packing in the crystal, which strongly indicate, that the space group is correctly selected (see below).
In compound **18** two of the *tert*-butylsilyl groups are disordered and were refined using a split model. Independent if the model is refined with disorder or not, extremely poor reliability factors are obtained, which can be traced back to the poor crystal quality.

The needle-like crystals are very soft and can easily deformed with a needle. If they are cooled down below 200 K, the crystals are split in two or more macroscopic individuals. It seems to be that the crystals consists of bundles of very small needles, that are shifted relative to each other and therefore, very poor diffraction pattern were obtained (Figure S117). This also leads to a very large anisotropic mosaic spread, which make a precise intensity measurement more difficult (see below). Because the crystals crystallize as very thin and very long needles, they must be cutted into smaller pieces, which increase the mosaic spread even more. It was also tried to perform twin refinements but too many individuals are involved.

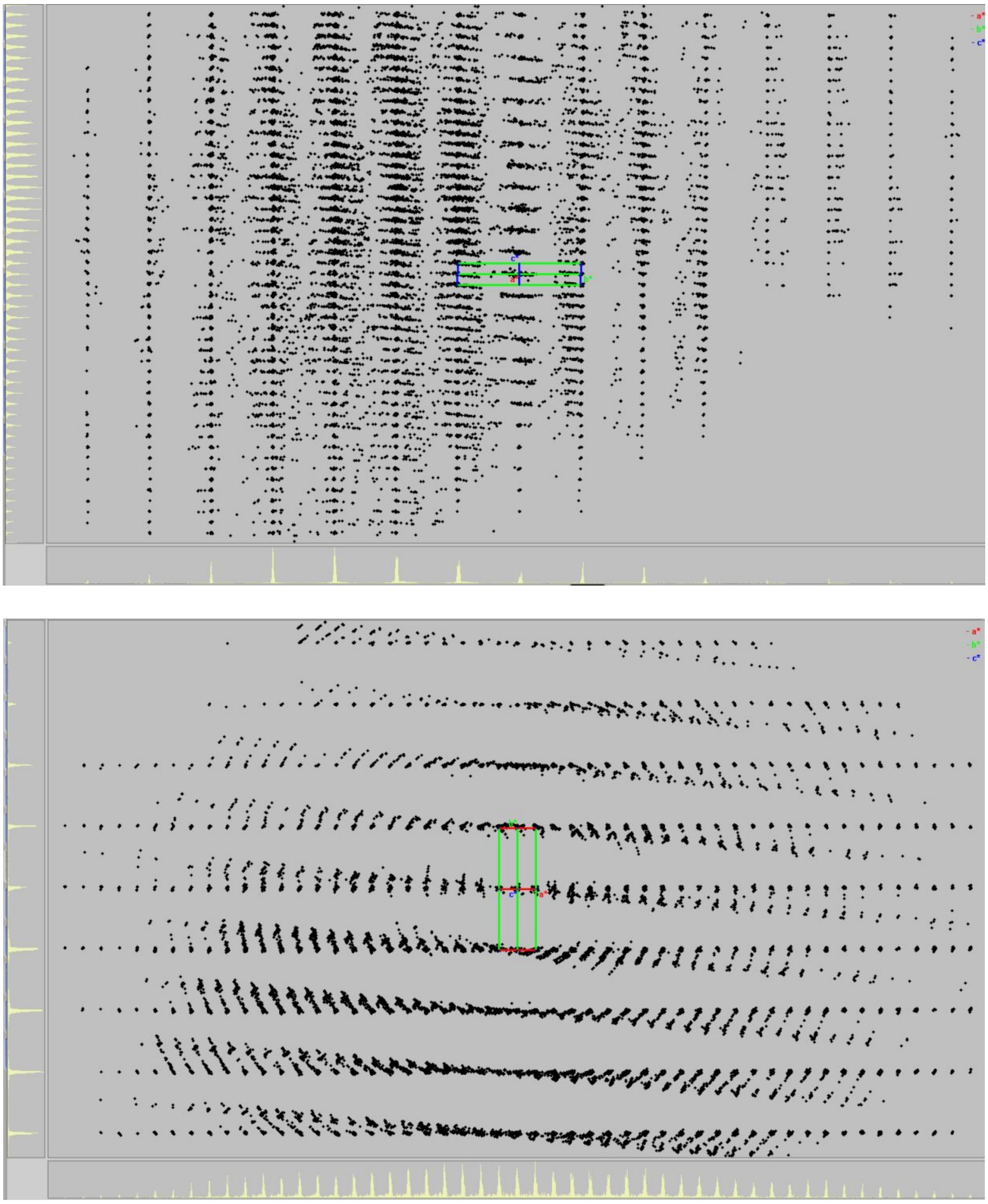

Figure S117: Diffraction pattern of compound **18** with view along a* (top) and c* (bottom).

Table S1: Selected crystal data and details of the structure determinations for compound **18**, **21** and **22**.

	**18**	**21**	**22**
Formula	$C_{62}H_{80}N_2O_4Si_4$	$C_{38}H_{24}N_2$	$C_{42}H_{32}N_2O_4$
MW / g mol^{-1}	11029.64	508.59	628.69
Crystal system	monoclinic	monoclinic	monoclinic
Space group	$P2_1/c$	*Ia*	*Ia*
a / Å	22.8774(3)	17.7852(2)	18.5655(3)
b / Å	6.7588(1)	5.12333(5)	5.12343(7)
c / Å	38.5210(4)	27.7647(4)	31.3711(5)
α / deg	90	90	90
β / deg	93.8609(12)	107.7741(13)	102.6214(17)
γ / deg	90	90	90
V / Å^3	5942.77(14)	2409.14(5)	2911.88(8)
T / K	200.0(2)	100.0(2)	100.0(2)
Z	4	4	4
D_{calcd} / g cm^{-3}	1.151	1.402	1.434
μ /mm^{-1}	1.283	0.627	0.736
2 θ_{max} / deg	136.492	160.766	160.23
Refl. collected	33957	41598	23995
Refl. unique	10754	4717	5901
R_{int}	0.0393	0.0237	0.0218
Refl. [$F_0 > 4\ \sigma(F_0)$]	9268	4651	5619
Parameters	729	361	438
R_1 [$F_0 > 4\ \sigma(F_0)$]	0.0744	0.0388	0.0502
wR_2 (all data)	0.2150	0.1115	0.1496
GOF	1.066	1.060	1.078
$\Delta\rho_{max;\ min}$ / e Å^{-3}	0.90; – 0.41	0.19; – 0.27	0.27; – 0.28

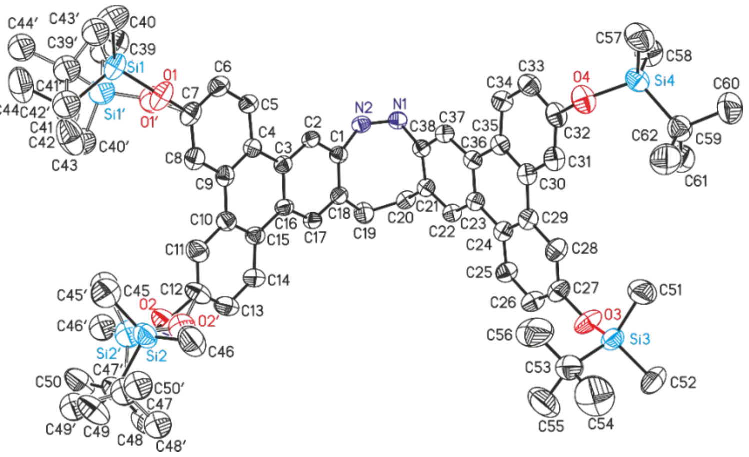

Figure 118: Crystal structure of TPD **18** with labeling and displacement ellipsoids drawn at the 50% probability level. The disordering of the *tert*-butylsilyl groups is indicated with full and open bonds. The H atoms are omitted for clarity.

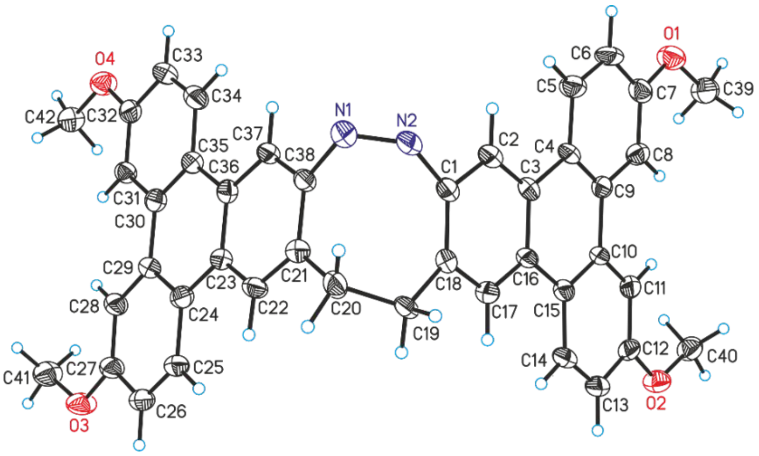

Figure S119: Crystal structure of TPD **21** with labeling and displacement ellipsoids drawn at the 50% probability level.

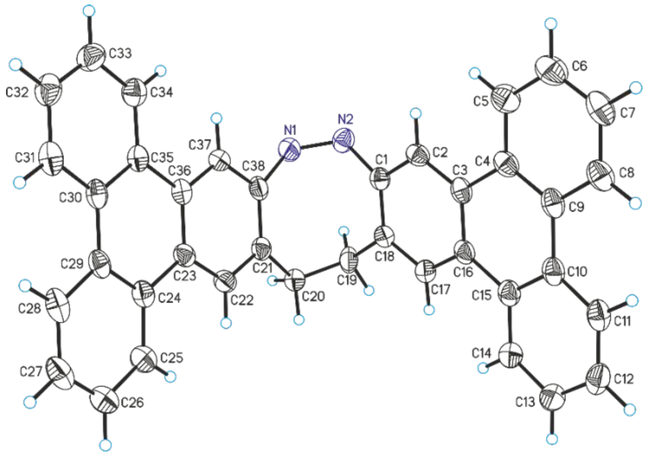

Figure S120: Crystal structure of TPD **22** with labeling and displacement ellipsoids drawn at the 50% probability level.

Structure description of TPDs 18, 21 and 22

In the crystal structure of TPD **18,** the molecules are arranged in columns, that are oriented parallel to the crystallographic *b*-axis and that are each surrounded by eight neighbored columns, leading to a closed packing arrangement (Figure S121).

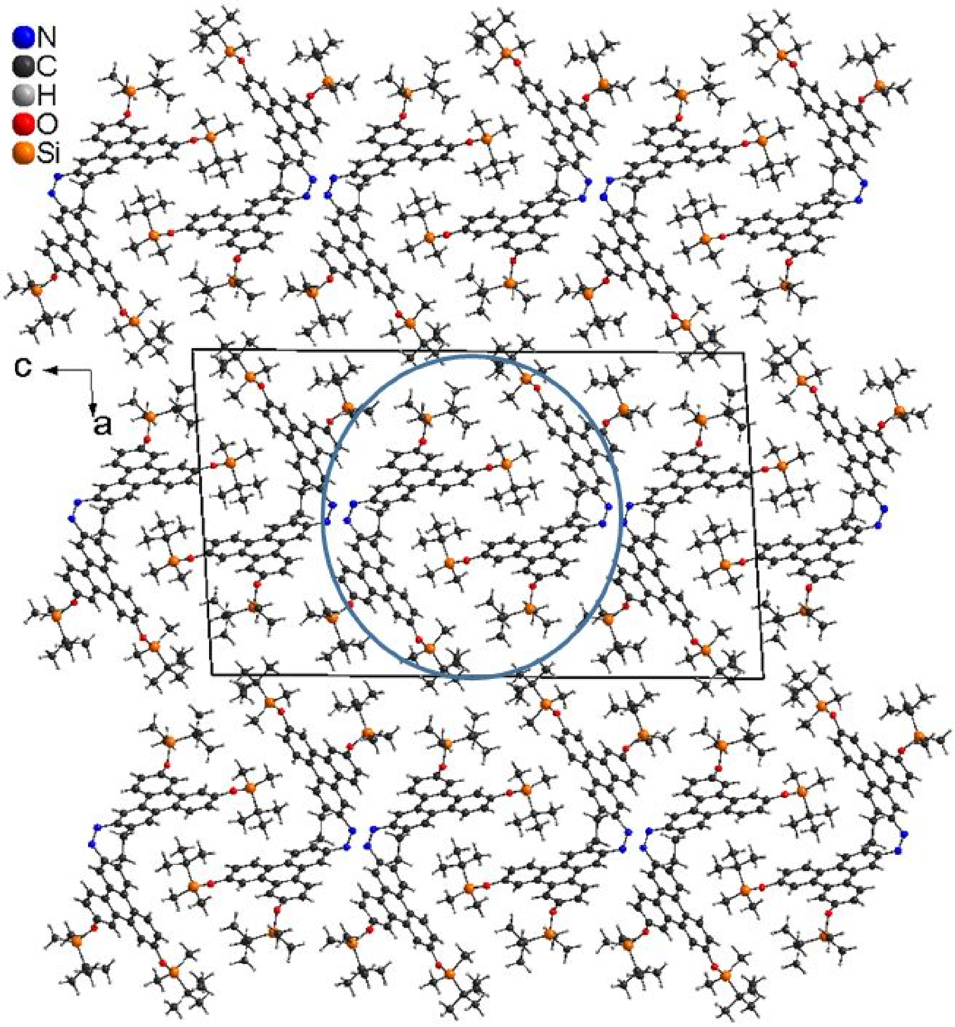

Figure S121: Crystal structure of TPD **18** with view along the crystallographic *b*-axis. The coloumns built up of pairs of molecules are indicated by a blue circle.

These columns consists of dime-like units, that are packed in a way, that each half of the molecules fits perfectly into the aperture of the neighboring molecule (Figure S122, left). Within these units the H atoms of the *tert*-butyl group points towards the 6-membered rings, which might be indicative for C-H$\cdots\pi$ interactions (Figure S122, right). In the whole structure there are no signs for $\pi\cdots\pi$ stacking interactions and between neighboring columns only weak van-der-Waals interactions seems to dominate.

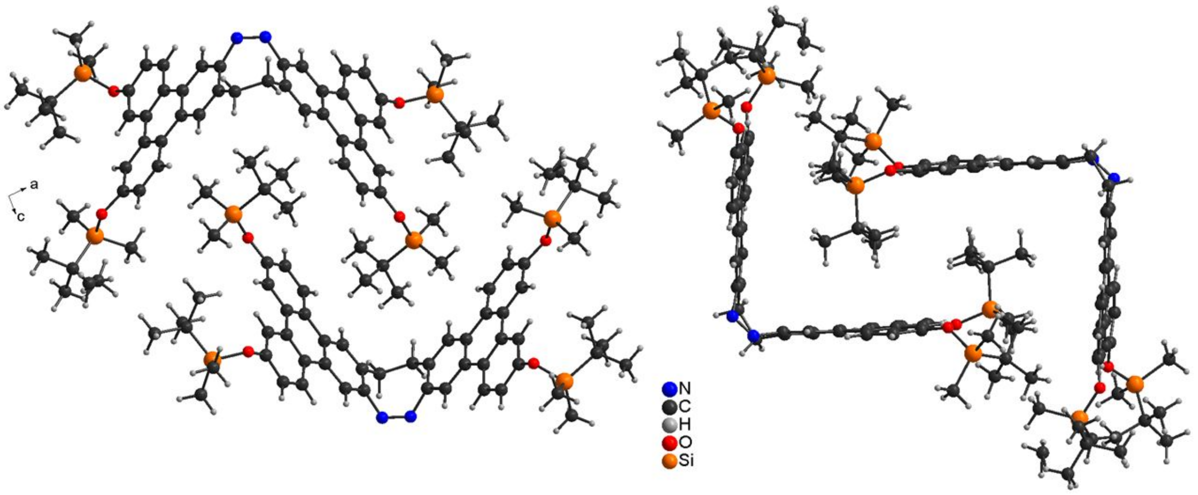

Figure S122: Crystal structure of TPD **18** with two different views of the dimeric units.

As mentioned above, the crystals form needles and to determine the orientation of the building blocks within these needles, the crystal faces were indexed (Figure S123). This prove that the needle axis coincidence with the crystallographic *b*-axis, which is the direction of the columns (Figure S123). Because these needles consist of bundles of needles, which are slightly misaligned relative to each other, this indicate that the intermolecular interactions between the columns is very weak. All this might be the reason for the very large anisotropic mosaic spread, which is expressed in the very poor diffraction patterns and the softness of the crystals.

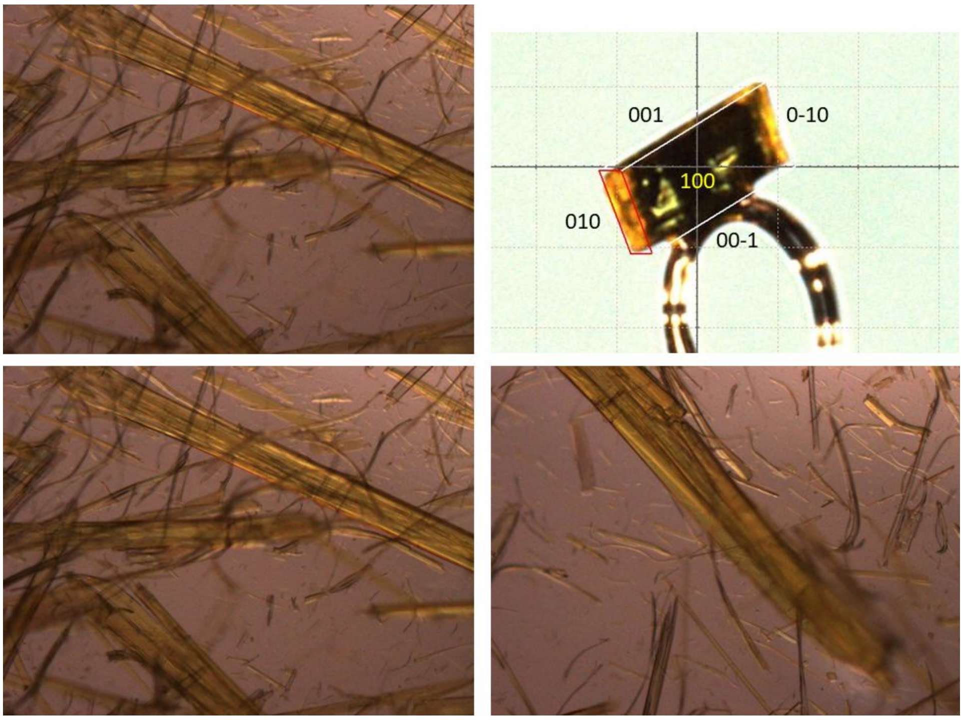

Figure S123: Microscopic images of the crystals of TPD **18**, together with an image of the crystal that was investigated with indices of the crystal faces. Please note, that this crystal was a part of a much longer needle, with the needle axis along the 010 direction.

The crystal structures of TPDs **21** and **22** are very similar or even isostructural, which is already obvious from their similar lattice parameters, same crystal symmetry and space group. As in TPD **18**, the molecules are arranged into columns that proceed along the crystallographic *b*-axis direction (Figure S124).

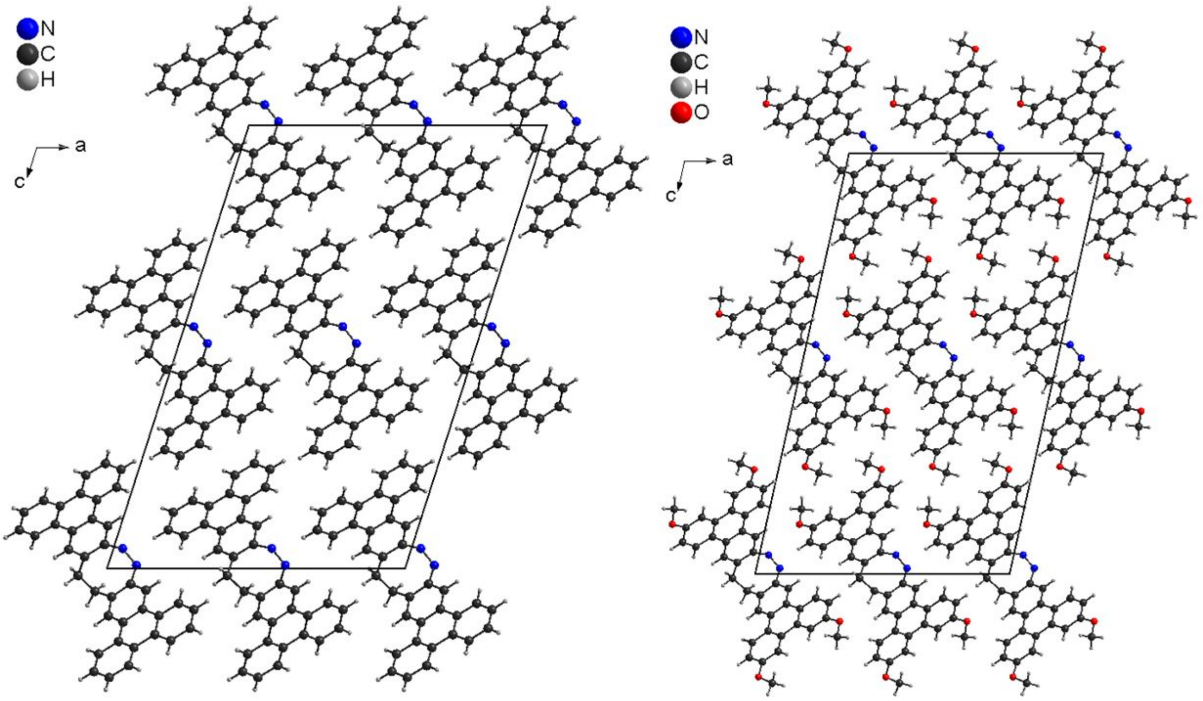

Figure S124: Crystal structure of TPDs **22** (left) and **21** (right) with view along the crystallographic *b*-axis.

In contrast to TPD **18**, no dimeric units are present and the molecule are stacked onto each into columns, where the aperture of each molecules is occupied with a neighboring molecule, leading to a dense structure (Figure S125). Within the columns the triphenylene rings are parallel, which is indicative for $\pi\cdots\pi$ interactions. Within the columns polar molecules always point in the same direction, which in fact is valid for the entire crystal structure, which prove the non-centrosymmetric arrangement of the building blocks (compare Figure S124 and S125). Finally, these columns are arranged in layers that are parallel to 10-1 (Figure S125), with neighboring layers shifted relative to each other.

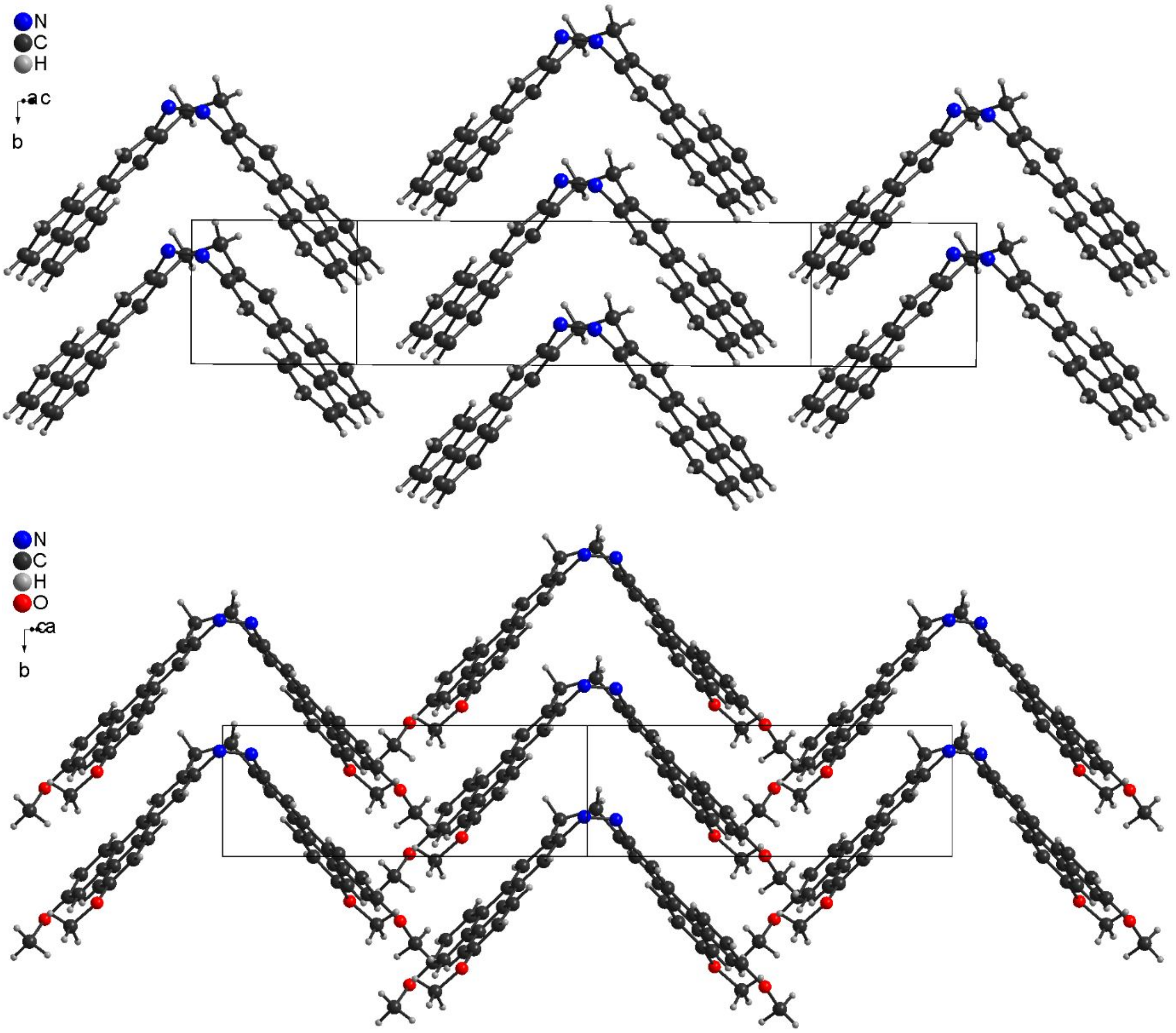

Figure S125: Crystal structure of TPDs **22** (top) and **21** (bottom) with view on 10-1.

S 6 References

[1] Fulmer, G. R.; Miller, A. J. M.; Sherden, N. H.; Gottlieb, H. E.; Nudelman, A.; Stoltz, B. M.; Bercaw, J. E.; Goldberg, K. I. NMR Chemical Shifts of Trace Impurities: Common Laboratory Solvents, Organics, and Gases in Deuterated Solvents Relevant to the Organometallic Chemist. *Organometallics* **2010**, *29* (9), 2176-2179.

[2] Fischer, G. M.; Daltrozzo, E.; Zumbusch, A. Selective NIR chromophores: Bis(Pyrrolopyrrole) Cyanines. *Angew. Chem. Int Ed.* **2011**, *50* (6), 1406-1409.

[3] Schmidt, B.; Riemer, M. Suzuki-Miyaura Coupling of Halophenols and Phenol Boronic Acids: Systematic Investigation of Positional Isomer Effects and Conclusions for the Synthesis of Phytoalexins from Pyrinae. *J. Org. Chem.* **2014**, *79* (9), 4104–4118.

[4] Romero, P. E.; Piers, W. E.; Decker, S. A.; Chau, D.; Woo, T. K.; Parvez, M. η^1 versus η^5 Bonding Modes in Cp*Al(I) Adducts of 9-Borafluorenes. *Organometallics* **2003**, *22* (6), 1266–1274.
